# MicroRNAs and essential components of the microRNA processing machinery are not encoded in the genome of the ctenophore *Mnemiopsis leidyi*

**DOI:** 10.1186/1471-2164-13-714

**Published:** 2012-12-20

**Authors:** Evan K Maxwell, Joseph F Ryan, Christine E Schnitzler, William E Browne, Andreas D Baxevanis

**Affiliations:** 1 Genome Technology Branch, National Human Genome Research Institute, National Institutes of Health, Bethesda, MD, 20892, USA; 2 Bioinformatics Program, Boston University, Boston, MA, 02215, USA; 3 Sars International Center for Marine Molecular Biology, University of Bergen, Bergen, 5008, Norway; 4 Department of Biology, University of Miami, Coral Gables, FL, 33146, USA

**Keywords:** *Mnemiopsis leidyi*, Ctenophore, Metazoa, microRNA, miRNA, Drosha, Pasha, Microprocessor complex, Ribonuclease III, RNase III

## Abstract

**Background:**

MicroRNAs play a vital role in the regulation of gene expression and have been identified in every animal with a sequenced genome examined thus far, except for the placozoan *Trichoplax*. The genomic repertoires of metazoan microRNAs have become increasingly endorsed as phylogenetic characters and drivers of biological complexity.

**Results:**

In this study, we report the first investigation of microRNAs in a species from the phylum Ctenophora. We use short RNA sequencing and the assembled genome of the lobate ctenophore *Mnemiopsis leidyi* to show that this species appears to lack any recognizable microRNAs, as well as the nuclear proteins Drosha and Pasha, which are critical to canonical microRNA biogenesis. This finding represents the first reported case of a metazoan lacking a Drosha protein.

**Conclusions:**

Recent phylogenomic analyses suggest that *Mnemiopsis* may be the earliest branching metazoan lineage. If this is true, then the origins of canonical microRNA biogenesis and microRNA-mediated gene regulation may postdate the last common metazoan ancestor. Alternatively, canonical microRNA functionality may have been lost independently in the lineages leading to both *Mnemiopsis* and the placozoan *Trichoplax*, suggesting that microRNA functionality was not critical until much later in metazoan evolution.

## Background

MicroRNAs (miRNAs) are a class of small RNA molecules derived from transcribed mRNA hairpin structures and spliced introns [1-3] that play a key role in mRNA targeting, leading to the degradation or translational repression of the target transcript. The regulatory functions of miRNAs are essential to many key biological processes in metazoans, including development, cell growth and death, stem cell maintenance, hematopoiesis, and neurogenesis. Aberrations in miRNA regulation have been linked to blood disorders, oncogenesis, and other malignancies in humans [4]. The hairpin structures in mRNA transcripts that give rise to primary microRNAs (pri-miRNAs) are not unique to miRNAs or metazoans; these hairpins can form much more frequently than functional pri-miRNAs [3,5] and can arise from inverted duplications, transposable elements, and genomic repeats [3,6,7]. Metazoans, however, possess a unique complement of cellular machinery for processing and transporting mature miRNAs to their targets that has not been identified in any non-metazoan species to date [8-11]. It has been observed that once novel miRNAs emerge in a metazoan lineage, they are very rarely lost. Thus, miRNAs are thought to represent strong phylogenetic markers and, through their ability to fine-tune gene expression, appear to be major drivers of biological complexity [8,12-14].

**Figure 1:**
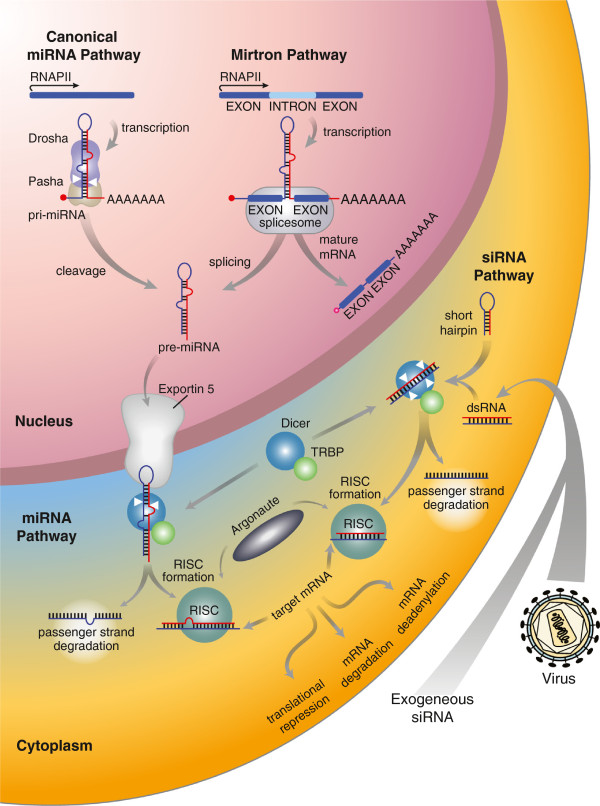
**Metazoan miRNA and siRNA pathways.** Representation of standard metazoan models for canonical miRNA biogenesis, mirtron biogenesis, and siRNA processing. The Drosha/Pasha protein complex is specific to canonical miRNA biogenesis and initiates cleavage of the primary miRNA (pri-miRNA) from transcribed mRNAs. Intronic miRNAs (mirtrons) bypass cleavage by Drosha/Pasha, generating precursor miRNAs (pre-miRNAs) via intron splicing of mRNAs. The Dicer and Argonaute proteins are responsible for further processing and transport of miRNAs, in addition to short-interfering RNAs (siRNAs) from exogenous sources, resulting in repression of mRNA targets.

The canonical miRNA biogenesis pathway in metazoans is part of the larger RNA interference (RNAi) pathway, which includes the closely related siRNA pathway (Figure 1). The miRNA pathway is distinct from the ancestral siRNA pathway in that it is initiated by the cleavage of hairpin structures (i.e., pri-miRNAs) from mRNAs in the nucleus by the Drosha/Pasha complex (also known as the Microprocessor complex), producing precursor-miRNAs (i.e., pre-miRNAs) that can be exported into the cytosol via the Exportin-5—Ran-GTP complex. After being transported into the cytosol, miRNAs and siRNAs undergo the same processing and targeting steps, initiated by Dicer cleavage and loading into the RNA-induced silencing complex (RISC) with Argonaute [15]. The siRNA pathway is an ancient biological defense mechanism used to ward off the integration of foreign nucleic acids, such as double stranded RNAs (dsRNAs) introduced by viruses, and is known to have existed in the oldest eukaryotes [7,10]. Thus, the emergence of the metazoan canonical miRNA biogenesis pathway most likely coincided with the evolution of the Drosha/Pasha complex found only in metazoans [10,11]. Functionally, the Drosha/Pasha complex enables cleavage of pri-miRNA hairpins that are subsequently exported out of the nucleus and processed by the pre-existing RNAi pathway.

Given the differences in molecular machinery, processing, and target recognition, miRNAs are thought to have evolved separately and exclusively in animals and plants [3,7,9,16]. However, a number of recent studies have reported identification of miRNAs in unicellular eukaryotes, including several thought to be homologs of miRNAs specific to animal and plant lineages [17-29]. These studies imply that miRNAs evolved once, early in eukaryotic evolution. Nevertheless, a recent report [30] reexamined these studies and found that, of the cumulative 232 reported miRNAs, none of the putative plant or animal homologs met established criteria for miRNA annotation; they were, instead, likely traces of other small RNAs (e.g., siRNAs, rRNAs, or snoRNAs) that happened to fit the length spectrum of mature miRNA sequences. Additionally, only 28 of the putative novel miRNAs passed the annotation criteria, and those were restricted to green and brown algae. In light of this evidence, it appears most likely that miRNAs evolved independently in multiple eukaryotic lineages, with the metazoan pathway being dependent upon the Drosha/Pasha protein complex.

Here, we describe an in-depth characterization of both the miRNA biogenesis pathway proteins and genomic regions that may correspond to pri-miRNA loci in the recently sequenced genome of *Mnemiopsis leidyi* (http://research.nhgri.nih.gov/mnemiopsis/). Recent phylogenomic analyses suggest that Ctenophora may be the earliest branching metazoan lineage [31,32], and genomic studies of a number of gene superclasses [33,34] and signaling pathways [35] in *Mnemiopsis* are consistent with this theory. If ctenophores are, indeed, the earliest metazoan branch, examining the genome of *Mnemiopsis* provides us a rare opportunity to better understand the origin of miRNA processing in metazoans. Alternatively, if ctenophores branched later in evolution and Porifera is the most basal metazoan lineage [36], *Mnemiopsis* still provides a valuable model from which to study the early evolution of this important small RNA processing pathway. Putative miRNAs (and the pathway proteins involved in their canonical biogenesis) have been studied in other non-bilaterian metazoans, including *Nematostella vectensis*, *Hydra magnipapillata*, *Trichoplax adhaerens*, and *Amphimedon queenslandica*[9,13,37]. The complete processing pathway was identified in all cases except *Trichoplax*, which lacks a Pasha homolog and recognizable miRNAs [6,9,38]. However, the presence of Drosha, Pasha, and miRNAs in *Amphimedon*, a metazoan lineage that branched prior to *Trichoplax*, suggests that *Trichoplax* must have lost miRNA functionality [9].

## Results and discussion

In order to understand the increasing complexity observed in the early evolution of animals, we have sequenced, annotated, and performed a preliminary analysis of the *Mnemiopsis* genome. During this process, we were able to map 99.4% of the 15,752 publicly available *Mnemiopsis* EST sequences to our genome assembly. These data are available through our *Mnemiopsis* Genome Project Web site (http://research.nhgri.nih.gov/mnemiopsis/). This Web site provides access to the assembled genome scaffolds, predicted protein models, transcriptome data, and EST data. The Web site also provides access to the *Mnemiopsis* Genome Browser, a BLAST utility, a gene-centric Wiki, protein domain annotations, and information on gene clusters mapped to human KEGG pathways via an intuitive and easy-to-use interface.

**Figure 2:**
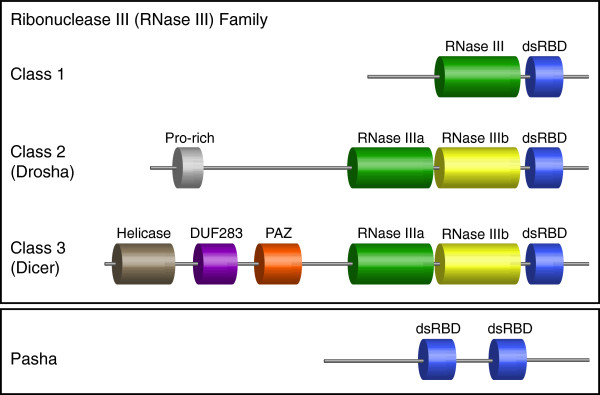
**Typical domain architectures of Ribonuclease III and Pasha proteins.** Members of the Ribonuclease III (RNase III) protein family all contain RNase III protein domains responsible for binding Mg^2+^ ions that cleave individual strands of dsRNA. The dsRNA binding domain (dsRBD) is common to most RNase III proteins and Pasha. Other common domains found in RNase III class 3 (Dicer) proteins include PAZ, a domain of unknown function (DUF), and a helicase. Pasha contains only tandem dsRBD domains, a domain architecture relatively common in other dsRNA binding proteins within metazoan proteomes.

Through our examination of the *Mnemiopsis* genome and its predicted proteome, we were able to identify multiple RNAi pathway proteins necessary for miRNA and siRNA processing, including Dicer, Argonaute, Ran, and exportin-5, but the miRNA-specific biogenesis pathway proteins Drosha and Pasha are strikingly absent. To our knowledge, this is the first reported case of a metazoan genome lacking a Drosha homolog. Since Dicer and Drosha are both members of the ribonuclease III (RNase III) protein family (Figure 2), we focused our analysis on the RNase III protein domain to better characterize the *Mnemiopsis* Dicer protein and to yield insight into how, through the evolution of this protein family in the Metazoa, the canonical miRNA biogenesis pathway may have emerged.

Drosha and Dicer belong to subclasses 2 (Drosha) and 3 (Dicer) of the RNase III protein family [39]. Both proteins are characterized by tandem RNase III domains that cleave dsRNA to a specific length, often producing cleavage products with a two-nucleotide 3^′^ overhang. However, distinct differences have been observed in the dsRNA-binding specificity and cellular localization of these two RNase III subclasses [39]. Class 3 RNase III enzymes have a PAZ domain that recognizes dsRNA ends with the distinctive two-nucleotide 3^′^ overhang indicative of prior RNase III cleavage. Class 2 RNase III enzymes do not appear to contain a domain with specific affinity for dsRNA and, instead, rely on complex formation in the nucleus with a co-factor (Pasha, or DGCR8 in vertebrates) that recognizes the ssRNA-dsRNA junctions characteristic of pri-miRNA hairpins [39]. RNase III class 3 Dicer-like proteins that lack a PAZ domain (and have a domain structure more similar to Drosha) have been identified in non-metazoans but function as part of an unrelated pathway [40]; they have also been identified in early branching metazoans, but their function has not been confirmed experimentally [40]. Since deletion of the PAZ domain in a functional Dicer has been shown to produce an RNase III enzyme without target specificity [41], there are likely functional binding domains other than PAZ within the RNase III class 3 subfamily.

To determine which class(es) of RNase III enzymes the *Mnemiopsis* Dicer protein is most closely related to, we performed a phylogenetic analysis on the RNase III domains of early-branching metazoan Dicer and Drosha proteins. We used HMMER [42] to search available non-bilaterian animal protein sequences (i.e., *Mnemiopsis*, *Nematostella*, *Hydra*, *Trichoplax*, and *Amphimedon*) to identify all candidate class 2 or class 3 RNase III proteins containing tandem RNase III domains. Our search yielded only one Dicer protein in *Mnemiopsis* and numbers of proteins consistent with other reports on the early-branching Metazoa [9,43]. We included a sample of bilaterian Dicer and Drosha sequences in our analysis to ensure each protein class was monophyletic across the Metazoa. We separated the RNase IIIa and RNase IIIb domains of each protein (Figure 2), aligned the domains, trimmed the poorly conserved and flanking regions, and used the resulting alignment as the basis for further phylogenetic analysis (see Additional file 1: Dataset 1a-b).

**Figure 3:**
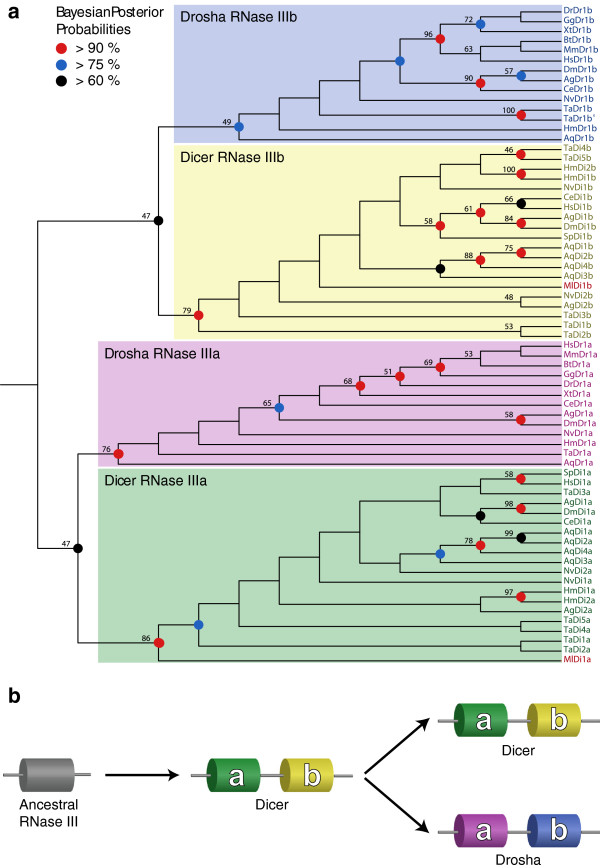
**Evolution of metazoan RNase III domains.a**, Cladogram of isolated RNase III domains from metazoan Dicer and Drosha proteins. *Mnemiopsis* Dicer protein RNase III domains are labeled in red. Bootstrap support values above 45, based on 1000 bootstrap replicates, are displayed on branches with Bayesian probabilities as indicated. See Additional file 7: Table S1 for information on sequence identifiers. **b**, Scenario for Drosha evolution. Dicer proteins evolved from a duplicated RNase III domain early in eukaryotic evolution. Drosha proteins evolved from a duplicated Dicer protein early in metazoan evolution. White ‘**a**’ and ‘**b**’ labels represent RNase IIIa and RNase IIIb domains of Dicer and Drosha proteins, respectively. Green, yellow, pink and blue domains correspond with the clades shown in **a**.

The tree generated from this alignment (Figure 3a) contains separate clades for each RNase III domain subgroup, confirming the characterization of the *Mnemiopsis* RNase III protein as a Dicer protein. Importantly, the topology unites the Drosha RNase IIIa and RNase IIIb domains with the respective Dicer RNase III domains. Given that RNase III class 2 (Drosha) proteins are restricted to the Metazoa [10,11], whereas RNase III class 3 (Dicer) proteins are found in the RNAi pathways of ancestral eukaryotes [7,10,43], this topology suggests that Drosha evolved from Dicer via a duplication event early in the evolution of the Metazoa, roughly coinciding with the emergence of miRNA functionality (Figure 3b). This observation contradicts the less parsimonious argument that these double RNase III domain-containing enzymes evolved independently from separate eubacterial RNase III domains [10] (Additional file 2: Figure S1).

It is possible that *Mnemiopsis* utilizes alternative methods for producing miRNAs for transcriptional regulation. Therefore, we searched for miRNAs using data from short RNA sequencing runs on two *Mnemiopsis* samples. We were unable to identify any known metazoan miRNAs that mapped to the *Mnemiopsis* genome. While we were able to predict several novel miRNA candidates using two methods, no predictions were reproducible across all samples and methods. In addition, ev en the highest-scoring predictions exhibited atypical read mapping signatures. Thus, we have classified all of these predictions as false positives, as they do not appear to be processed by the canonical miRNA machinery (see Methods).

Some spliced introns can correctly fold into pre-miRNAs, called mirtrons, independent of cleavage by Drosha and Pasha [1,2,6] (Figure 1). However, within the *Mnemiopsis* genome, only a handful of introns have predicted secondary structures suggestive of mirtron-coding potential, and none of these have read mapping signatures to indicate that they are functional mirtrons. The presence of exportin-5 and downstream RNAi pathway proteins Dicer and Argonaute in *Mnemiopsis* could indicate the existence of an alternative mechanism for miRNA production that predates the canonical miRNA pathway. The lack of recognizable miRNAs in our small RNA sequences, however, suggests that this scenario is unlikely. Recently, cases of functional exogenous miRNAs acquired via ingestion were identified in animals [44], suggesting a possible dietary mechanism by which *Mnemiopsis* could utilize miRNA regulatory functions in the absence of a functional endogenous canonical pathway. However, the mechanism for exogenous miRNA activity remains poorly understood.

It has been hypothesized that mirtrons may have predated the Drosha/Pasha-mediated pathway, based on the observation that the mechanistic requirements for their evolution may have been fairly simple [1,2]. The identification of mirtrons in rice [3,45] and the presence of the necessary machinery in *Mnemiopsis* (described above) are consistent with this hypothesis. However, given the absence of functional mirtrons in *Mnemiopsis*, it appears more likely that miRNA functionality evolved alongside the Drosha/Pasha-mediated pathway, independently of the mirtron pathway. Discerning the point in evolutionary time in which mirtrons became functional will require a thorough analysis of the genomes of additional species beyond nematodes, mammals, and avians [3,45].

## Conclusions

**Figure 4:**
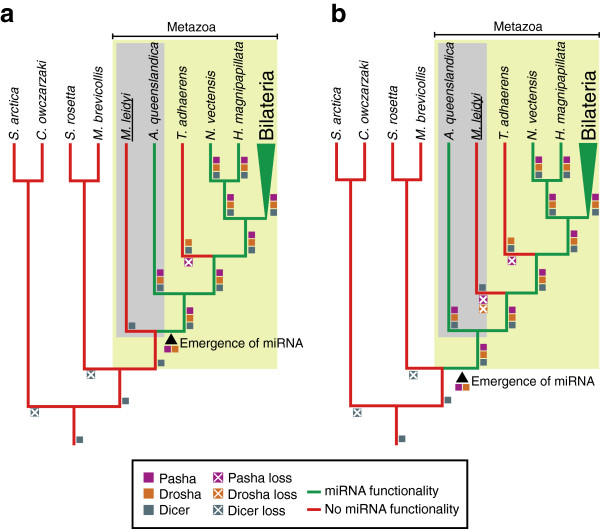
**Scenarios of the evolutionary implications of canonical miRNA functionality absence in*Mnemiopsis leidyi. *a**, Ctenophora (represented by *M*. *leidyi*) branching earlier than Porifera (represented by *A*. *queenslandica*). In this scenario, miRNA functionality likely emerged after the branching of Ctenophora. **b**, Porifera branching prior to Ctenophora. In this scenario, miRNA functionality coevolved with the Metazoa and was lost from *Mnemiopsis leidyi*, along with the biogenesis proteins Drosha and Pasha. Also shown are the closest outgroups to the Metazoa with sequenced genomes (i.e., *S*. *arctica*, *C*. *owczarzaki*, *S*. *rosetta*, and *M*. *brevicollis*); see Methods for details on the identification of miRNA pathway proteins in these species.

The implications of these results depend upon the phylogenetic position of Ctenophora. If ctenophores are the most basal metazoan clade, the most parsimonious explanation for our observations is that metazoan miRNA functionality originated after ctenophores diverged from the rest of animals (Figure 4a). Alternatively, if poriferans are the most basal metazoan clade, then Drosha, Pasha and canonical miRNA functionality must have been lost in the *Mnemiopsis* lineage (Figure 4b). If the latter were true, then canonical microRNAs and their machinery would have been independently lost in both Ctenophora and Placozoa. This, along with the large-scale losses of miRNAs described in acoelomorphs [46] and cnidarians [37], would contradict the premise that miRNAs are ultraconserved, canalized characters that are continuously added, but rarely lost – and, as such, would challenge their usefulness as phylogenetic markers [12,13].

Our data supports a scenario in which the role of miRNAs in fine-tuning gene expression was not solidified until more recently in metazoan evolution and thus indicates that miRNA regulatory functions were, perhaps, non-essential during early metazoan diversification. Given this, the lack of recognizable miRNA functionality in *Mnemiopsis* supports a scenario with Ctenophora branching at the base of the Metazoa, prior to the emergence of miRNA functionality (Figure 4a). It may also indicate that a novel RNA-based regulatory pathway evolved either within the ctenophore lineage or as a precursor to the canonical miRNA pathway recognizable in the rest of the Metazoa. In either case, ctenophores represent an intriguing model for better understanding the early evolution of small RNA-based regulatory functions, shedding light on a point in evolutionary time that may have predated the need for additional plasticity in key molecular systems inherent to animals. We expect that further exploration of the genomes of other ctenophores, early branching metazoans, and closely related non-metazoans will help determine the exact point in evolutionary history at which both canonical and mirtron-based miRNA pathways (and their components) emerged.

## Methods

### Sample preparation

Two RNA sources were used for sequencing miRNAs. Sample 1 was collected in Woods Hole, MA from mixed stage late embryos 15–30 hours post-fertilization. Total RNA was prepped with TRI-Reagent. Sample 2 was collected in Miami, FL from mixed stage embryos 0–30 hours post-fertilization. Total RNA was prepped with TRIzol Reagent and resuspended in 50 μl of THE RNA solution spiked with RNAsecure.

### Sequencing of short RNAs and genome mapping

Libraries of small RNAs were prepared from 5 μg total RNA using Illumina’s Small RNA Alternative v1.5 Sample Prep Protocol with the following modifications. Adapter ligation times were increased from 1 hour to 6 hours, a total of 15 PCR cycles were used, and a 10% acrylamide gel was used for better resolution of properly ligated sequences from unligated free adapters. Sequencing of adapter libraries was performed on an Illumina GAiix using version 5 chemistry and RTA version 1.8.70.0. Both runs were 36-cycle single read. Raw sequencing data was post-processed using CASAVA 1.7.0 and deposited in the NCBI Short Read Archive (http://www.ncbi.nlm.nih.gov/sra/), accession SRA057204.

The 3^′^ adapter sequence ATCTCGTATGCCGTCTTCTGCTTGT was trimmed from reads using Novocraft’s Novoalign v2.07.18. After filtering reads of low quality, we mapped the trimmed reads to the *Mnemiopsis* genome independently with both Novoalign and Bowtie v0.12. [47] (allowing up to two mismatches). Novoalign successfully mapped 65.9% of reads from sample 1 (out of 14,965,804 reads after removal of an overrepresented, unannotated rRNA transcript) and 58.5% of reads from sample 2 to the genome (out of 30,311,098 reads). Bowtie mapped 68.3% and 66.7% of reads from each sample, respectively. Rough estimates showed that ~94% of read mappings from sample 1 were represented in sample 2 and, conversely, ~91% of read mappings from sample 2 were represented in sample 1. This indicates that differences in samples and sequencing protocols did not significantly affect read sources.

### Canonical miRNA prediction

miRDeep2 [48] and miRanalyzer (version 0.2) [49] were used to predict miRNAs from our short RNA sequence data and the *Mnemiopsis* genome. Candidate predictions were restricted to those present in both samples in at least one read. Next, candidate miRNAs were ranked by the number of methods predicting them, where identification in both methods was considered most confident and predictions by miRDeep2-only were favored over miRanalyzer-only. This ranking is a result of noise filtering to reduce false positives in miRDeep2, producing fewer predictions (143 in sample 1 and 248 in sample 2 with miRDeep2, versus 4197 in sample 1 and 9056 in sample 2 with miRanalyzer).

For miRDeep2, we used all metazoan mature miRNA sequences in miRBase (http://mirbase.org/ftp.shtml) as the input set of known miRNAs. This is used to identify potentially conserved miRNAs, in addition to providing a template for estimating the false positive rate and signal-to-noise ratio at different score cutoffs [48]. No known metazoan miRNAs, including those of other early branching metazoans studied in this work, were identified in the *Mnemiopsis* samples based on strict sequence similarity having identical seed sequences (nucleotides 2–7) and a maximum of three mismatches in the remaining mature or mature-star arm [13]. The reported signal-to-noise distributions for each sample were notably dissimilar to those reported in other species with known miRNAs [48]. The signal-to-noise ratio is expected to be roughly monotonically increasing with respect to miRDeep2 scores and, in other species including *Nematostella*, should provide a true positive score cutoff at which signal-to-noise is 10:1, or in the worst case (sea squirt), at least 3.5:1. In our samples, the signal-to-noise ratio peaks at 1.6:1 and 1.3:1, respectively at a score cutoff of 4, and drops off at higher scores (Additional file 3: Dataset 2e & 2h). Although in those experiments the input set of known miRNAs was specific to a single species, opposed to all metazoans, the distributions of signal-to-noise ratio versus score cutoffs does not appear high enough to make any positive predictions in our experiments. Further, our top predictions were sample-specific.

For miRanalyzer, we used all Rfam sequences, provided automatically by the program, to identify known miRNAs and to filter short RNA sequences from other sources. In both samples, no known miRNA mature or mature-star sequences were identified. We did not use miRanalyzer predictions alone to identify novel miRNAs because of the immense number of predictions made. Manual analysis showed that the most highly expressed predictions corresponded to rRNA sequences. We therefore only used miRanalyzer predictions to support miRDeep2 predictions.

The best predictions over all samples and methods were made by miRDeep2 on sample 2. Thus, in addition to looking at the top predictions using the combinatorial criteria described above, we also looked at miRDeep2 predictions for each sample independently. No predicted miRNA had the ideal combination of read mapping signature and secondary structure to be considered a confident miRNA. Top miRDeep2 predictions for each sample are summarized in Additional file 3: Dataset 2a-b. Raw prediction outputs are provided in Additional file 3: Dataset 2c-h.

Finally, in the absence of confident miRNA predictions by the methods described above, we searched the *Mnemiopsis* genome specifically for miR-100 and miR-2022, as these miRNAs are the only known miRNAs (to our knowledge) thought to be conserved outside of the Bilateria; miR-100 appears to be conserved between *Nematostella* and bilaterians, while miR-2022 appears to be conserved between *Nematostella* and *Hydra*. Querying the *Mnemiopsis* genome with BLASTN using the conserved portions of the respective mature sequences (miR-100: ACCCGTAGATCCGAACTTGTG, miR-2022: TTTGCTAGTTGCTTTTGTCCC) yielded partial hits in both cases (14 and 16-nucleotide identity, respectively). However, only one hit (for miR-2022 on scaffold ML1502) covered the expected seed site, and no short RNA sequencing reads from either sample mapped to this region. In all, these results support the absence of miR-100 and miR-2022 in *Mnemiopsis* in addition to all other canonical miRNAs.

### Mirtron prediction

The basis of our mirtron prediction method was the combination of an absolute count of mapping reads from Bowtie [47] and predicted secondary structures by UNAFold [50] scored using an SVM approach trained on fly mirtrons [51]. All introns of length 50 to 120 nt in *Mnemiopsis* were considered candidate mirtrons (3953 total, Additional file 3: Dataset 2k) and scored by the SVM based on secondary structure alone. For every candidate mirtron, we independently counted the number of reads pooled from both samples mapping in the correct orientation to the 3^′^ or 5^′^ splice sites, with a three-nucleotide buffer in both directions. Our strict read mapping criteria was meant to identify the most likely candidates; while mirtron reads can be found further from the splice sites in other species, the majority of reads tend to fall in this range. We produced three rankings of candidate mirtrons based on the highest scored secondary structures, most correctly mapping reads, and finally by the intersection of the two. Our results did not uncover any high-confidence mirtron candidates. Scoring of the secondary structures resulted in noticeably fewer and lower quality predictions compared to scores reported on *Drosophila melanogaster* and *Caenorhabditis elegans* introns [51] (Additional file 4: Figure S2).

We analyzed introns up to length 150 nt (7324 additional introns from those length 50–120 nt) in the case that *Mnemiopsis* mirtrons, like *Amphimedon* miRNAs [9], were longer than those of flies. The intron length distribution can be seen in Additional file 5: Figure S3. We produced a ranked list based on read counts and manually analyzed the secondary structures of the most highly expressed. Again, no acceptable mirtron candidates were identified.

The best candidates had very low read counts and generally hit only one of the two splice sites; if they are truly functional mirtrons, they are not expressed at high enough levels to be concluded as functional. In addition, their secondary structure predictions were less than ideal relative to known mirtrons in other species. The best identified mirtron candidate (scaffold ML4098, from 40399–40490 on the ‘+’ strand) contains only seven reads total from a single sample (sample 2), six at the 5^′^ splice site and one at the 3^′^ splice site, and does not have a characteristic loop or 5^′^/3^′^ overhang structure. See Additional file 6: Figures S4-S8 for a summary of the best manually curated predictions, based on the combination of predicted secondary structure and read mappings.

### Annotation of miRNA pathway proteins

RNAi pathway proteins identified in *Mnemiopsis* throughout the course of this study have been deposited in GenBank (http://www.ncbi.nlm.nih.gov/Genbank/), with accessions JQ437405 (Dicer), JQ437406 (Argonaute), JQ437407 (Exportin-5), and JQ437408 (Ran). Two additional Argonaute family members were annotated: JX483728 and JX483729. Identification and annotation of *Mnemiopsis* proteins was based on high-scoring reciprocal BLASTP hits to the human RefSeq protein set. TBLASTN was also used but did not identify any better candidates. Human Dicer and Drosha both hit uniquely to the same *Mnemiopsis* protein, but reciprocal BLASTP results favored Dicer. The protein models of all species represented in Figure 4 were searched with HMMER 3.0 [42] for tandem RNase III domains; no Dicer or Drosha candidates were identified in the closest non-metazoan outgroups (i.e., *Monosiga brevicollis*, *Salpingoeca rosetta*, *Capsapora owczarzaki* and *Sphaeroforma arctica*). *Nematostella*, *Hydra*, *Trichoplax*, and *Amphimedon* protein sequence data were downloaded from the Joint Genome Institute (JGI) Web site and protein sequence data for the closest non-metazoan outgroups were downloaded from the Origins of Multicellularity Sequencing Project Web site of the Broad Institute of Harvard and MIT (http://www.broadinstitute.org/) in November 2011. In some of these species, the RNase III domains of Dicer and Drosha proteins were not properly annotated. In these cases, we instead used published, manually curated sequences [9] or the appropriate RefSeq entries when those were not available. Other RNase III sequences from the bilateria and eubacteria included in our analysis were selected from sequences used in a previous study [10] or sampled from RefSeq and GenBank. All accession numbers for RNase III enzymes included in our final analysis are reported in Additional file 7: Table S1. The trimmed RNase III domain sequences used to build the phylogenetic tree in Figure 3 were aligned with HMMER 3.0 [42] and manually padded in cases where terminal gaps could be reliably filled. Residues 59–98 were manually trimmed from the alignment based on poor conservation. Both alignments are reported in Additional file 1: Dataset 1a-b.

Figure 3 was generated to better-categorize the *Mnemiopsis* RNase III enzyme as a Dicer or Drosha and to better-understand the origin of Drosha. This phylogenetic tree was built on the trimmed alignment described above. ProtTest v2.4 [52] was used to pick the best model of evolution and selected the LG model with optimization of substitution rates, gamma model of rate heterogeneity, and empirical amino acid frequencies (PROTGAMMAILGF model). We used RAxML v7.2.8a [53] to build trees seeded on 24 random starting trees and 24 maximum parsimony trees. We also ran MrBayes v3.1.2 [54] to construct a Bayesian tree, using five million iterations on five chains with a burn-in factor of 25%. MrBayes was run using the second best model selected by ProtTest since the LG model is not available in MrBayes: RtRev with optimized substitution rates, gamma model of rate heterogeneity, and empirical amino acid frequencies. All 49 trees were compared in a maximum likelihood framework, and we reported the tree with the highest likelihood (RAxML with maximum parsimony starting tree, log likelihood = −5895.384778). Support for clades was assessed using 1000 bootstrap replicates and posterior probabilities computed with MrBayes. NEWICK formatted trees are provided in Additional file 1: Dataset 1c-d with bootstraps and Bayesian posterior probabilities.

## Competing interests

The authors declare no competing interests.

## Authors’ contributions

EKM performed the majority of computational analyses and was primary author of the manuscript. JFR, CES, WEB and ADB contributed to performing the miRNA predictions, protein/pathway identification, and phylogenetic analyses. WEB performed experimental analysis. All authors contributed to the design of the study and preparation of the manuscript.

## Supplementary Material

Additional file 1 **Dataset 1.** contains a folder of source data files (i.e., protein sequence alignments and NEWICK formatted trees containing bootstrap support and Bayesian posterior probabilities, respectively) in plain text format to accompany the phylogenetic trees produced for Figure 3 and Additional file 2: Figure S1. Click here for file

Additional file 2 **Figure S1.** provides a phylogenetic tree, and the corresponding most parsimonious evolutionary scenario, produced on the data used in Figure 3 with the addition of eubacterial sequences, addressing the less parsimonious scenario of Drosha’s direct evolution from eubacterial RNase III enzymes [10]. Click here for file

Additional file 3 **Dataset 2.** contains a folder of output data files in plain text format related to the miRNA predictions (both canonical and mirtron) produced by the various programs described in the Methods. Click here for file

Additional file 4 **Figure S2.** provides the prediction score histograms produced by the mirtron prediction method used [51]. Click here for file

Additional file 5 **Figure S3.** shows the intron length distribution for *Mnemiopsis leidyi*. Click here for file

Additional file 6 **Figures S4-S8.** illustrate the top five mirtron preditions based on the criteria described in the Methods. Click here for file

Additional file 7 **Table S1.** defines the RNase III protein sequence identifiers used in the phylogenetic trees described above Click here for file
