# Supplementary material for: MicroRNAs and essential components of the microRNA processing machinery are not encoded in the genome of the ctenophore Mnemiopsis leidyi
Source: BMC Genomics. 2012 Dec 20;13:714. doi: 10.1186/1471-2164-13-714 (PMC3563456; doi:10.1186/1471-2164-13-714)
Supplement: Additional file 2 — Figure S1. provides a phylogenetic tree, and the corresponding most parsimonious evolutionary scenario, produced on the data used in Figure 3 with the addition of eubacterial sequences, addressing the less parsimonious scenario of Drosha’s direct evolution from eubacterial RNase III enzymes [10]. [file 1471-2164-13-714-S2.pdf]

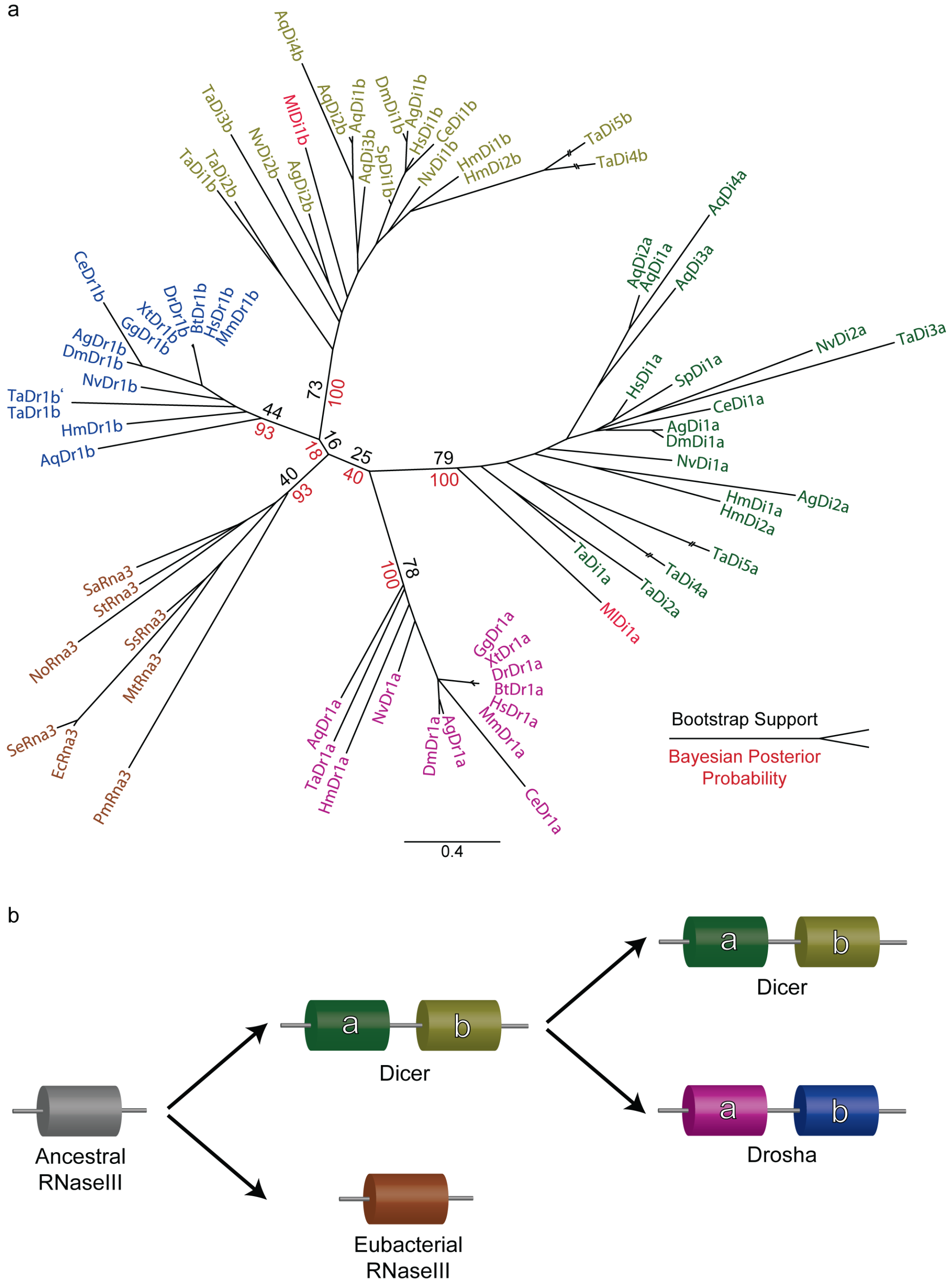

Additional Figure 1: **(a)** Phylogenetic tree of isolated RNaseIII domains from metazoan Dicer (green and yellow) and Drosha (pink and blue) proteins and eubacterial RNaseIII class 1 enzymes (brown). The RNaseIIIa and RNaseIIIb domains from the Mnemiopsis Dicer protein are highlighted in red. Support for branches is based on 1000 bootstrap replicates and Bayesian posterior probabilities computed with Mr. Bayes. The displayed tree was generated by the same steps as Figure 3 and shows the maximum likelihood tree based on 49 trees; 24 with RAxML based on random starting trees, 24 with RAxML based on 24 maximum parsimony starting trees, and one bayesian tree from Mr. Bayes. The maximum likelihood tree (log likelihood = −6838.149995) shown was computed by RAxML using a random starting tree. See Additional Table 1 for information on sequence identifiers and Additional Dataset 1e-h for source files. **(b)** Indicates the most parsimonious scenario for Drosha evolution as described in Figure 3, with the addition of eubacterial RNaseIII proteins. This scenario supports independent evolution of the Dicer and Drosha sequences from eubacterial RNaseIIIs. The RNaseIIIa and RNaseIIIb domains of Dicer and Drosha proteins are indicated with the respective a and b labels in white. Domain colors correspond to clades in **(a)**.
