# Supplementary material for: MicroRNAs and essential components of the microRNA processing machinery are not encoded in the genome of the ctenophore Mnemiopsis leidyi
Source: BMC Genomics. 2012 Dec 20;13:714. doi: 10.1186/1471-2164-13-714 (PMC3563456; doi:10.1186/1471-2164-13-714)
Supplement: Additional file 3 — Dataset 2. contains a folder of output data files in plain text format related to the miRNA predictions (both canonical and mirtron) produced by the various programs described in the Methods. [file 1471-2164-13-714-S3.zip › Additional_Dataset_2/a_sample1_md2_scores_gt2.pdf]

Provisional ID	:	ML3140_38009
Score total	:	82.3
Score for star read(s)	:	3.9
Score for read counts	:	80.7
Score for mfe	:	0.5
Score for randfold	:	-2.2
Score for cons. seed	:	-0.6
Total read count	:	170
Mature read count	:	155
Loop read count	:	14
Star read count	:	1

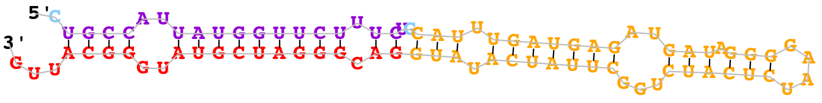

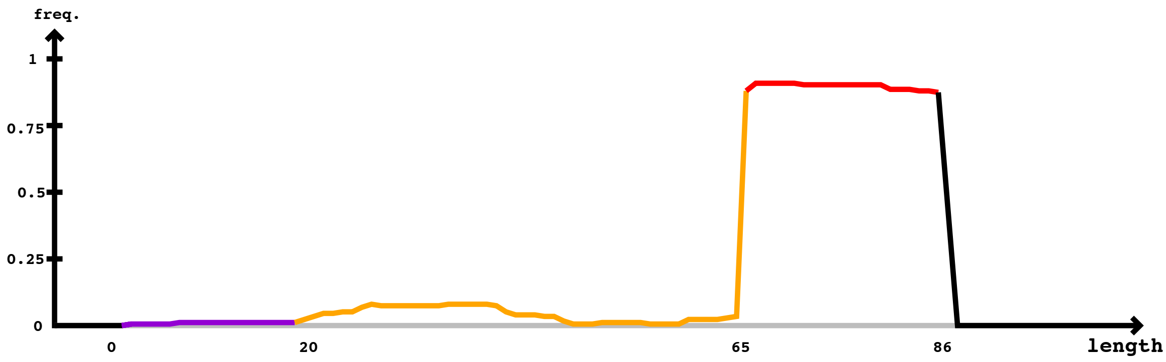

Star **Mature**

```
5'- acucccugccauuaugguucuuucugcauuugaugagaugauaggggaaucucaucuggcuuaucauauggacgggaucguaugggcauugaaucaauucuucacgaucag   -3'   obs
    acucccugccauuaugguucuuucugcauuugaugagaugauaggggaaucucaucuggcuuaucauauggacgggaucguaugggcauugaaucaauucuucacgaucag         exp
    ..((..((((..((((((((.((..(((.(((((((..(((.(((....))))))...))))))).))))).))))))))..))))..)).....................   reads   mm   sample
    ......ugccauuaugguucuuucu......................................................................................   1   0   seq
    ...........uuaugguucuuucugcauuug...............................................................................   1   0   seq
    ........................ugcauuugaugagaugauag...................................................................   1   0   seq
    ........................ugcauuugaugagaugauagg..................................................................   4   0   seq
    .........................gcauuugaugagaugauaggg.................................................................   2   0   seq
    .............................uugaugagaugauaggggaa..............................................................   1   0   seq
    ..............................ugaugagaugauaggggaauc............................................................   3   0   seq
    ...............................gaugagaugauaggggaaucu...........................................................   2   0   seq
    .......................................gauaggggaaucucaucuggc...................................................   1   0   seq
    .......................................................cuggcuuaucauauggacggg...................................   1   0   seq
    ................................................................Aauauggacgggaucguaugg..........................   2   1   seq
    ................................................................cauauggacgggaucguaugg..........................   1   0   seq
    ....................................................................uggacgggaucguaugggca.......................   1   0   seq
    .....................................................................ggacgggaucguaugggcauu.....................   1   0   seq
    ......................................................................Uacgggaucguaugggcauug....................   1   1   seq
    ......................................................................gacgCgaucguaugggcauug....................   1   1   seq
    ......................................................................gacgggaucguauggAcauug....................   1   1   seq
    ......................................................................Aacgggaucguaugggcauug....................   1   1   seq
    ......................................................................Nacgggaucguaugggcauug....................   1   1   seq
    ......................................................................gacgggGucguaugggcauug....................   1   1   seq
    ......................................................................gacgggaucguaugggcCuug....................   1   1   seq
    ......................................................................gacgggaucguaugggcauug....................   141   0   seq
    .......................................................................acgggaucguauggAcauuga...................   1   1   seq
    .......................................................................acgggaucguaugggcauCga...................   1   1   seq
    .......................................................................acgggaucguaugggcauuga...................   3   0   seq
```

Provisional ID	:	ML0346_9413
Score total	:	36.7
Score for star read(s)	:	3.9
Score for read counts	:	27.7
Score for mfe	:	0.5
Score for randfold	:	1.6
Score for cons. seed	:	3
Total read count	:	66
Mature read count	:	63
Loop read count	:	2
Star read count	:	1

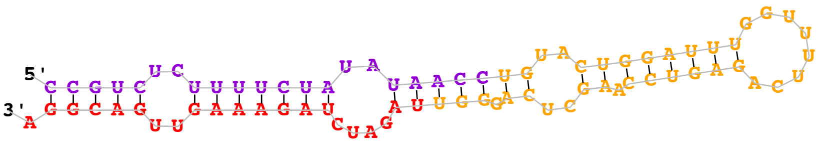

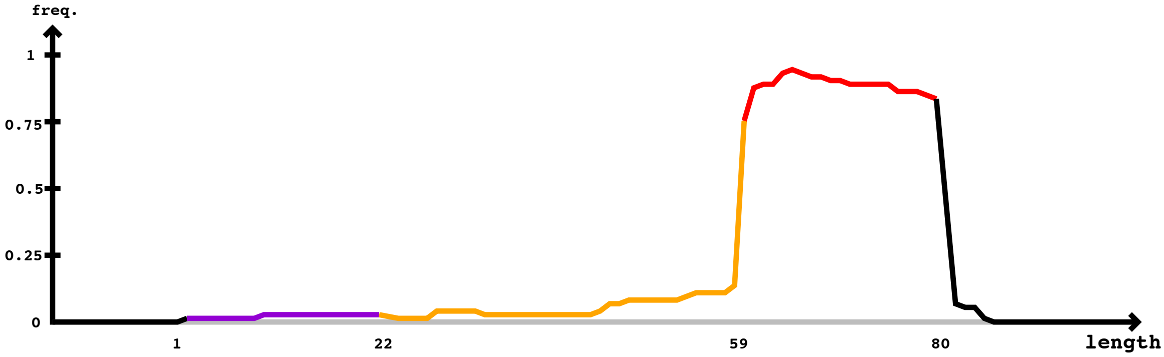

Star **Mature**

```
5'- aguuucucgcauccgucucuuuucuauauaaccuguacuggauuugguuuucagaguccaagcucaggguuagaucuagaaaguugacggauuuucuaauuccgaaugguu   -3'    obs
    aguuucucgcaucccgucucuuuucuauauaaccuguacuggauuugguuuucagaguccaagcucaggguuagaucuagaaaguugacggauuuucuaauuccgaaugguu          exp
    ..........(((((((..(((((((..(((((((..((((((((........)))))).))..)).)))))....)))))))..)))))))...................   reads   mm   sample
    ............ccgucucuuuucuauauaacc..............................................................................   1       0    seq
    ....................uuucuauauaaccuguacuggau....................................................................   1       0    seq
    ......................................uggauuugguuuucGgaguccaagcucaggg..........................................   1       1    seq
    ......................................uggauuugguuuucGgaguccaagcucagggu.........................................   1       1    seq
    .......................................................guccaagcucaggguuagauc...................................   1       0    seq
    ........................................................uccaagcucaggguuagaucu..................................   1       0    seq
    ........................................................uccaagcucaggguuaAaucuagaa...............................   1       1    seq
    ..........................................................caagcucaggguuagaucuag................................   1       0    seq
    ................................................................caggguuaAaucuagaaaguug.........................   1       1    seq
    .................................................................aggguuagaucuagaaaguug.........................   1       0    seq
    .....................................................................uuagaucuagaaaguugacgg.....................   1       0    seq
    .....................................................................uuagaucuagaaaguugacggau...................   2       0    seq
    ......................................................................uagaucuagaaaguugacg......................   1       0    seq
    ......................................................................uagaucuagaaaguugacgga....................   45      0    seq
    .......................................................................agaucuagaaaguugacggau...................   9       0    seq
    ........................................................................gaucuagaaaguugacggauu..................   1       0    seq
    ..........................................................................ucuGgaaaguugacggauuuu................   1       1    seq
    ..........................................................................ucuagaaaguugacggauuuu................   2       0    seq
    ...........................................................................cuagaaaguugacggauuuuc...............   1       0    seq
```

Provisional ID	:	ML0082_2930
Score total	:	23
Score for star read(s)	:	3.9
Score for read counts	:	19
Score for mfe	:	-4.4
Score for randfold	:	1.6
Score for cons. seed	:	3
Total read count	:	49
Mature read count	:	32
Loop read count	:	6
Star read count	:	11

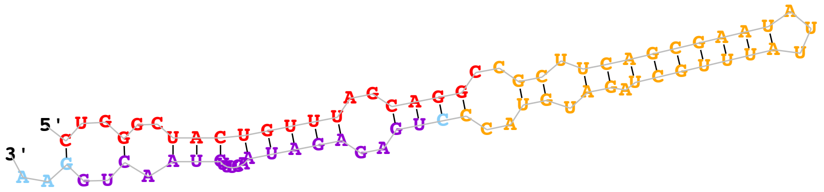

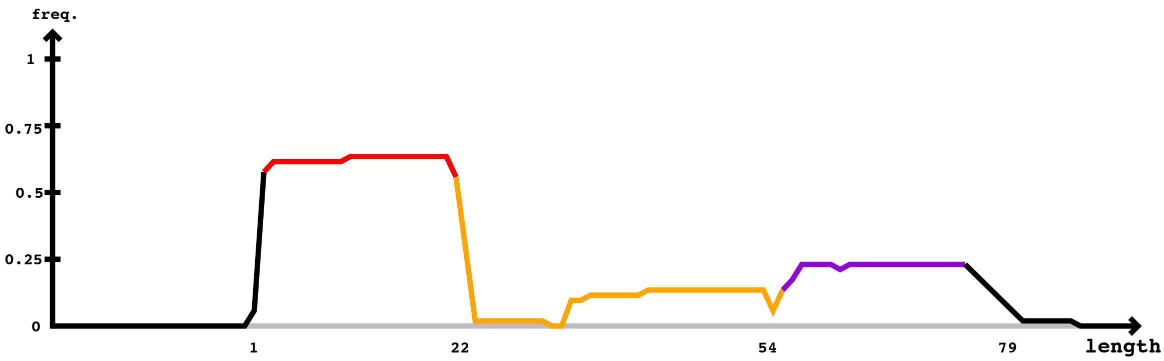

Mature   Star

```
5'- nnnnnnnnnnnnnnnnnncucugggcuacuguuuagcaggccgcuucagcgaauauuauuugcuagauguacccugagagauaacuuuguaacuggaacuuuagucauuua  -3'  obs
    nnnnnnnnnnnnnnnnnncucugggcuacuguuuagcaggccgcuucagcgaauauuauuugcuagauguacccugagagauaacuuuguaacuggaacuuuagucauuua       exp
    ...................((..(..((((((((..((((..((.(((((((((...))))))).)).))..))))..))))).....))).)..))..............   reads   mm   sample
    ...................ucugggcuacuguuuagcagg.......................................................................   3       0    seq
    ....................cugggcuGcuguuuagcaggc......................................................................   1       1    seq
    ....................cugggcuacuguuuagcaggc......................................................................   26      0    seq
    .....................ugggcuacuguuuagcagg.......................................................................   1       0    seq
    .....................ugggcuacuguuuagcaggcc.....................................................................   1       0    seq
    .............................uguuuagcaggccgcuucagc.............................................................   1       0    seq
    ....................................................auauuauuugcuagauguacc......................................   4       0    seq
    ....................................................auGuuauuugcuagauguacc......................................   1       1    seq
    ......................................................auuauuugcuagauguacc......................................   1       0    seq
    ............................................................ugcuagauguacccugagag...............................   1       0    seq
    .........................................................................cugagagauaacuuuguaacu.................   2       0    seq
    ..........................................................................ugagagauaacuuuguaacug................   3       0    seq
    ..........................................................................ugagagauaacuuuguaacuggaa.............   1       0    seq
    ...........................................................................gagagauaacuuuguaacugg...............   2       0    seq
    ............................................................................agagauaacuuuguaacugg...............   1       0    seq
    ............................................................................agagauaacuuugAaacugga..............   1       1    seq
    ............................................................................agagauaacuuuguaacugga..............   1       0    seq
    .................................................................................uaacuuuguaacuggaacuuuagC......   1       1    seq
```

Provisional ID	:	ML0246_7391
Score total	:	12.6
Score for star read(s)	:	3.9
Score for read counts	:	10.8
Score for mfe	:	-2.9
Score for randfold	:	-2.2
Score for cons. seed	:	3
Total read count	:	33
Mature read count	:	32
Loop read count	:	0
Star read count	:	1

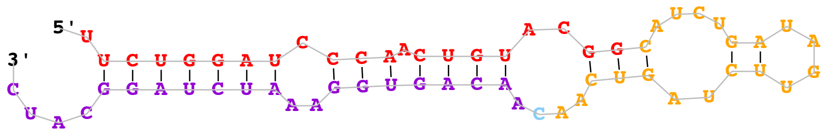

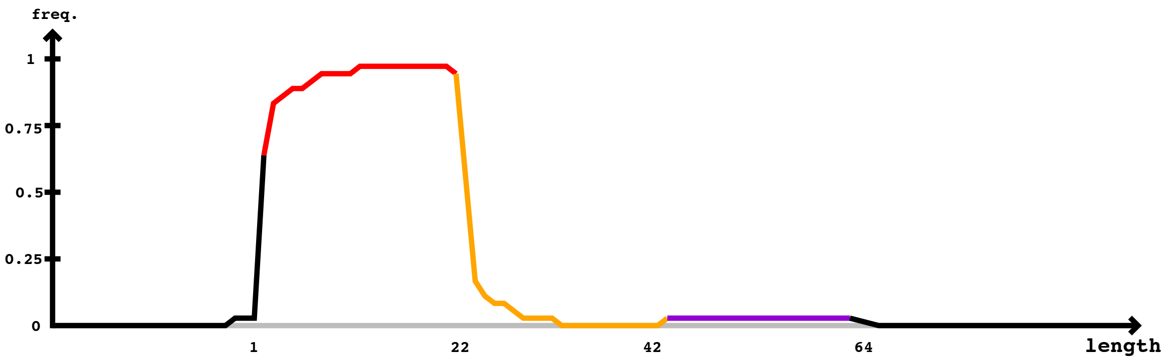


```
                         Mature                                          Star
5'- gaacaauggaacuaaaacacuucuggaucccaacuguacggcaucugauaguucuagucaacaacaguggaaaucuaggcaucugauaguucuagucaacaacaguggaaa   -3'    obs
    gaacaauggaacuaaaacacuucuggaucccaacuguacggcaucugauaguucuagucaacaacaguggaaaucuaggcaucugauaguucuagucaacaacaguggaaa          exp
    ((....(((((((.......(((((((.(((.((((..(((....((....))..)))....)))))))..)))))))......))))))).)).................   reads     mm     sample
    .................cacuucuggauccUaacuguacggca....................................................................   1         1      seq
    ....................uucuggaucccaacuguacg.......................................................................   1         0      seq
    ....................uucuggaucccaacuguacgg......................................................................   20        0      seq
    ....................uucuggaucccaacuguacggc.....................................................................   1         0      seq
    .....................ucuggaucccaacuguacgg......................................................................   1         0      seq
    .....................ucuggaucccaacuguacggc.....................................................................   6         0      seq
    ......................cuggaucccaacuguacggca....................................................................   1         0      seq
    .......................uggaucccaacuguacggcau...................................................................   1         0      seq
    .........................gaucccaacuguacggcaucu.................................................................   1         0      seq
    ..........................aucccaacuguacggcaucug................................................................   1         0      seq
    ..............................caacuguacggcaucugauag............................................................   1         0      seq
    ..............................................................aacaguggaaaucuaggcauc............................   1         0      seq
```

Provisional ID	:	ML0622_14689
Score total	:	5.3
Score for star read(s)	:	-1.3
Score for read counts	:	0
Score for mfe	:	2
Score for randfold	:	1.6
Score for cons. seed	:	3
Total read count	:	54
Mature read count	:	54
Loop read count	:	0
Star read count	:	0

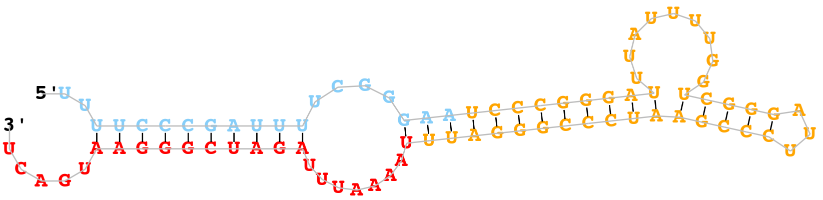

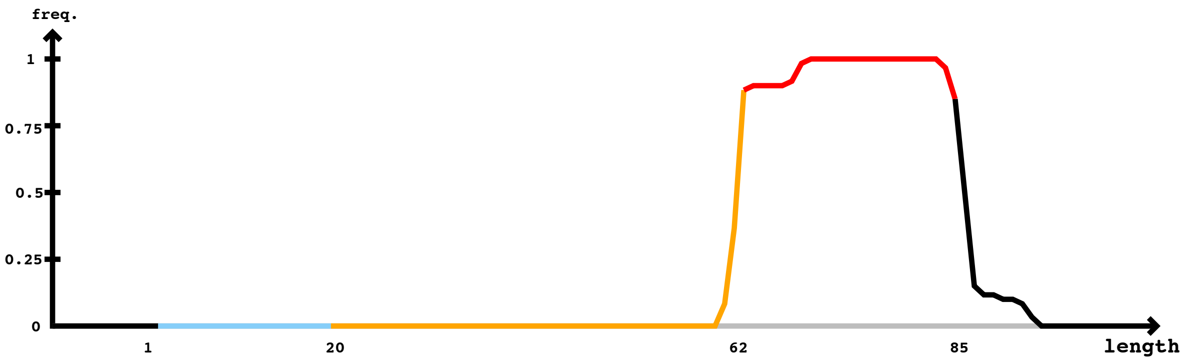

Star   Mature

```
5'- ucgggaaaguuuucccgauuuucgggaaucccgggauuuauuuuggucgggauucccgaaucccgggauuuaaaauuuagaucgggaaugacuucacuaguuguacuuguagg   -3'    exp
    ..((..(((((((((((((((....((((((((((((.........(((((...))))))))))))))))).......)))))))))).)))))..))...............   reads   mm   sample
    ....................................................................uuuaaaauuuagaucgggaaugG......................   1       1    seq
    ....................................................................uuuaaaauuuagaucgggaaugac.....................   3       0    seq
    ....................................................................uuuaaaauuuagaucgggaaugacu....................   1       0    seq
    .....................................................................uuaaaauuuagaucgggaaugac.....................   4       0    seq
    .....................................................................uuaaaauuuagaucgggaaugacu....................   13      0    seq
    ......................................................................uaaaauuuagaucgggaauga......................   1       0    seq
    ......................................................................uaaaauuuagaucgggaaugacu....................   27      0    seq
    ......................................................................uaaaauuuagaucgggaaugacuu...................   1       0    seq
    ......................................................................uaaaauuuagaucgggaaugacuuc..................   2       0    seq
    .......................................................................Uaaauuuagaucgggaaugacuucac................   1       1    seq
    ...........................................................................uuuagaucgggaaugacuucacuag.............   1       0    seq
    ............................................................................uuagaucgggaaugacuucacua..............   1       0    seq
    ............................................................................uuagaucgggaaugacuucacuaC.............   1       1    seq
    ............................................................................uuagaucgggaaugacuucaUuag.............   1       1    seq
    ............................................................................uuagaucgggaaugacuucacuagu............   1       0    seq
    .............................................................................uagaucgggaaugacuucacuagC............   1       1    seq
```

Provisional ID	:	ML0266_7721
Score total	:	5.1
Score for star read(s)	:	-1.3
Score for read counts	:	0
Score for mfe	:	1.8
Score for randfold	:	1.6
Score for cons. seed	:	3
Total read count	:	38089
Mature read count	:	31913
Loop read count	:	5844
Star read count	:	332

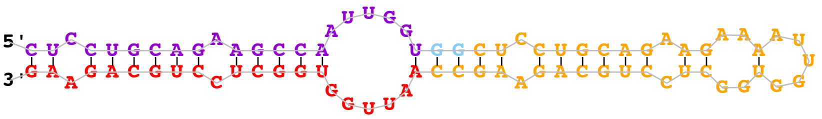

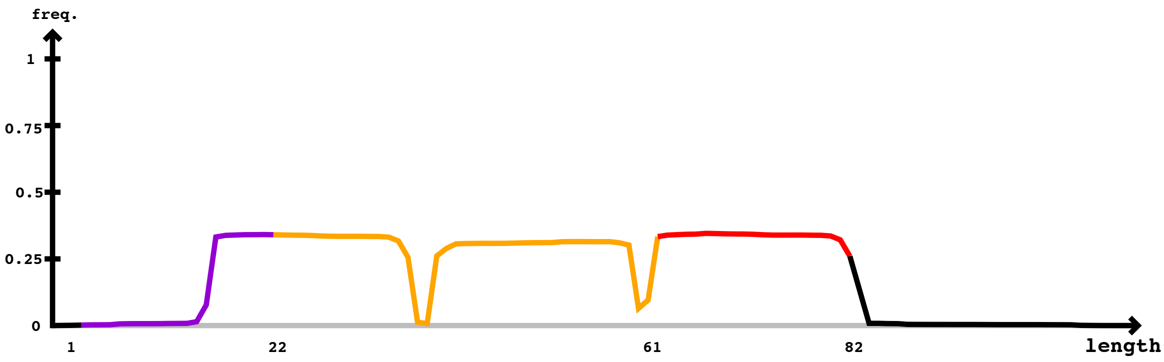

Star **Mature**

```
5'- gcuccugcagaagccaauugguggcuccugcagaagaaaauugguggcuccugcagaagccaauugguggcuccugcagaagaucagucccuuaaguaaaugaugguccac  -3'   obs
    gcuccugcagaagccaauugguggcuccugcagaagaaaauugguggcuccugcagaagccaauugguggcuccugcagaagaucagucccuuaaguaaaugaugguccac        exp
    .((.((((((.(((((.....(((((.((((((.((..(.....)..)).)))))).))))).....))))).)))))).))....(..(((((......))).))..)..   reads   mm   sample
    Acuccugcagaagccaauu............................................................................................   2       1    seq
    gcuccugcagaagccaauug...........................................................................................   6       0    seq
    gcuccugcagaagccaauugg..........................................................................................   162     0    seq
    Acuccugcagaagccaauugg..........................................................................................   6       1    seq
    gcuccugcagaUgccaauugg..........................................................................................   2       1    seq
    gcuccugcCgaagccaauugg..........................................................................................   2       1    seq
    .cuccugcagaagccaauu............................................................................................   2       0    seq
    .cuccugcagaagccaauugg..........................................................................................   4       0    seq
    .cuUcugcagaagccaauuggu.........................................................................................   4       1    seq
    .cuccugcagaagccaauuAgu.........................................................................................   2       1    seq
    .cuccugcagaagcAaauuggu.........................................................................................   2       1    seq
    .cuccugcagaagccaauuggu.........................................................................................   190     0    seq
    .cuccugcagaagccaauugguU........................................................................................   2       1    seq
    ..uccugcagaagccaauuggu.........................................................................................   2       0    seq
    ..uccugcagaagccaauuggug........................................................................................   110     0    seq
    ..uccuUcagaagccaauuggug........................................................................................   2       1    seq
    ..Accugcagaagccaauuggug........................................................................................   6       1    seq
    ..uccugcagaagccaauuggCg........................................................................................   2       1    seq
    ..Nccugcagaagccaauuggug........................................................................................   2       1    seq
    ..uccugcagaagccaauuggugU.......................................................................................   8       1    seq
    ...ccugcagaagccaauuggug........................................................................................   2       0    seq
    ...ccugcagaagcAaauuggugg.......................................................................................   4       1    seq
    ...Ucugcagaagccaauuggugg.......................................................................................   2       1    seq
    ...ccCgcagaagccaauuggugg.......................................................................................   2       1    seq
    ...ccugcagaagccaaCuggugg.......................................................................................   2       1    seq
    ...ccugcagaagccaauuggugg.......................................................................................   28      0    seq
    ....cugcagaagccaauuggug........................................................................................   4       0    seq
    ....cugcagaagccaauuggugg.......................................................................................   8       0    seq
    ....cugcagaagccaauugguggc......................................................................................   68      0    seq
    ....cugcagaagcAaauugguggc......................................................................................   2       1    seq
    ....cugcagaagccaauugguggcuU....................................................................................   2       1    seq
    .....ugcagaagccaauuggug........................................................................................   6       0    seq
    .....ugcagaagccaauuggugg.......................................................................................   8       0    seq
```

Star **Mature**

```
gcuccugcagaagccaauugguggcuccugcagaagaaaauugguggcuccugcagaagccaauugguggcuccugcagaagaucagucccuuaaguaaaugaugguccac
.....Cgcagaagccaauugguggg.......................................................................................  2     1  seq
.....uAcagaagccaauugguggc......................................................................................  2     1  seq
.....ugcagaagccaauugguggc......................................................................................  144   0  seq
.....ugcagaagccaauugguggcG.....................................................................................  2     1  seq
.....ugcagaagccCauugguggcu.....................................................................................  2     1  seq
.....ugcagaagcAaauugguggcu.....................................................................................  2     1  seq
.....ugcagUagccaauugguggcu.....................................................................................  2     1  seq
.....ugcagaagccaauugguggcC.....................................................................................  2     1  seq
.....ugcagaagccaauuAguggcu.....................................................................................  2     1  seq
.....ugcagaagccaauugguggcu.....................................................................................  356   0  seq
.....ugcagaagccaauugguggcuc....................................................................................  2     0  seq
.....ugcagaagccaauugguggcuU....................................................................................  116   1  seq
......gcagaagccaauugguggc......................................................................................  2     0  seq
......gcagaagccaauugguggcu.....................................................................................  6     0  seq
......gcagaagcUaauugguggcuc....................................................................................  2     1  seq
......gcagaagcAaauugguggcuc....................................................................................  2     1  seq
......gcagaagccaauugguggcuc....................................................................................  102   0  seq
......gcagUagccaauugguggcuc....................................................................................  2     1  seq
......gcagaagccaauugguggcucU...................................................................................  6     1  seq
.......cagaagcAaauugguggcuc....................................................................................  2     1  seq
.......cagaagccaauugguggcucc...................................................................................  18    0  seq
.......cagaagcAaauugguggcucc...................................................................................  2     1  seq
........agaagcAaauugguggcuccu..................................................................................  2     1  seq
........agaagccaauugguggcuccu..................................................................................  8     0  seq
.........gaagccaauugguggcuccug.................................................................................  6     0  seq
..........aagccaauugguggcuccug.................................................................................  2     0  seq
..........aagccGauugguggcuccugc................................................................................  2     1  seq
..........aagccaauugguggcuccugc................................................................................  102   0  seq
..........aagcUaauugguggcuccugc................................................................................  2     1  seq
..........aagccaauugguggcuccCgc................................................................................  2     1  seq
..........aagccaauuggCggcuccugc................................................................................  2     1  seq
..........aagcAaauugguggcuccugc................................................................................  16    1  seq
..........aGgccaauugguggcuccugc................................................................................  2     1  seq
..........aagccaauugguggcuccugcU...............................................................................  4     1  seq
...........agccaauugguggcuccugc................................................................................  2     0  seq
...........agccaauugguggcuccugca...............................................................................  18    0  seq
...........agcAaauugguggcuccugca...............................................................................  6     1  seq
...........agAcaauugguggcuccugca...............................................................................  2     1  seq
............gccaauugguggcuccug.................................................................................  2     0  seq
............gccaauugguggcuccugc................................................................................  2     0  seq
............gcAaauugguggcuccugcag..............................................................................  4     1  seq
............Nccaauugguggcuccugcag..............................................................................  2     1  seq
............gccaauugguggcuccugcag..............................................................................  58    0  seq
............gccaauugguggcuccuAcag..............................................................................  2     1  seq
............gccaauugguggcuccugcagU.............................................................................  2     1  seq
............gUcaauugguggcuccugcagaa............................................................................  2     1  seq
............gccaauugguggcuccugcagaagU..........................................................................  2     1  seq
.............ccaauugguggcuccugc................................................................................  2     0  seq
.............ccaauugguggcuccugca...............................................................................  6     0  seq
.............ccaauugguggAuccugcag..............................................................................  2     1  seq
.............cAaauugguggcuccugcag..............................................................................  2     1  seq
.............ccaauugguggcuccugcag..............................................................................  54    0  seq
.............ccaauugguggcuccCgcag..............................................................................  2     1  seq
.............ccaauugguggcuccugcaga.............................................................................  1084  0  seq
.............ccaauugguggcuccugcGga.............................................................................  2     1  seq
.............ccaauugguUgcuccugcaga.............................................................................  2     1  seq
.............Acaauugguggcuccugcaga.............................................................................  2     1  seq
.............Ncaauugguggcuccugcaga.............................................................................  6     1  seq
.............ccaauugguggcCccugcaga.............................................................................  2     1  seq
.............ccaauuggugAcuccugcaga.............................................................................  2     1  seq
.............ccaauugguggcuccCgcaga.............................................................................  2     1  seq
.............ccaaCugguggcuccugcaga.............................................................................  2     1  seq
.............ccaauugUuggcuccugcaga.............................................................................  2     1  seq
.............cAaauugguggcuccugcaga.............................................................................  14    1  seq
.............ccaauugguggcuccugcagU.............................................................................  4     1  seq
.............ccCauugguggcuccugcaga.............................................................................  2     1  seq
.............ccaaAugguggcuccugcaga.............................................................................  2     1  seq
.............ccaauuUguggcuccugcaga.............................................................................  4     1  seq
.............ccaauuggugUcuccugcaga.............................................................................  2     1  seq
.............ccaauugguggcuUcugcaga.............................................................................  6     1  seq
```

Star **Mature**

```
gcuccugcagaagccaauugguggcuccugcagaagaaaauugguggcuccugcagaagccaauugguggcuccugcagaagaucagucccuuaaguaaaugaugguccac
.............ccaauugguggcuccugcagaU............................................................................    24      1    seq
.............Acaauugguggcuccugcagaa............................................................................    2       1    seq
.............ccaauugguggcuccugcagaa............................................................................    2       0    seq
.............ccaauugguggcuccugcagaag...........................................................................    2       0    seq
..............caauuggugAcuccugca...............................................................................    2       1    seq
..............caauugguggcuccugca...............................................................................    22      0    seq
..............Naauugguggcuccugcag..............................................................................    6       1    seq
..............caaCugguggcuccugcag..............................................................................    2       1    seq
..............caauugguggcuUcugcag..............................................................................    2       1    seq
..............caauugguggcuccCgcag..............................................................................    2       1    seq
..............caauugguggcuccugcag..............................................................................    356     0    seq
..............caauugAuggcuccugcag..............................................................................    4       1    seq
..............caauugguggcuccugcUg..............................................................................    2       1    seq
..............caauugguggcuUcugcaga.............................................................................    10      1    seq
..............caauugguCgcuccugcaga.............................................................................    6       1    seq
..............Naauugguggcuccugcaga.............................................................................    6       1    seq
..............cCauugguggcuccugcaga.............................................................................    4       1    seq
..............caauugguggcuccugcCga.............................................................................    2       1    seq
..............Gaauugguggcuccugcaga.............................................................................    3       1    seq
..............caauuggugAcuccugcaga.............................................................................    2       1    seq
..............caauugguggcuccugcUga.............................................................................    2       1    seq
..............caauuggCggcuccugcaga.............................................................................    2       1    seq
..............caauugAuggcuccugcaga.............................................................................    10      1    seq
..............caauugguAgcuccugcaga.............................................................................    2       1    seq
..............caauugguggcuccugUaga.............................................................................    2       1    seq
..............caauugguggcuccugcGga.............................................................................    2       1    seq
..............caauugguggcuccugcaga.............................................................................    990     0    seq
..............caauugguggcCccugcaga.............................................................................    4       1    seq
..............caauuUguggcuccugcaga.............................................................................    2       1    seq
..............caauugguggcuccuUcaga.............................................................................    2       1    seq
..............caauugguggcucAugcaga.............................................................................    2       1    seq
..............caauugguggcuccugcagU.............................................................................    8       1    seq
..............caauGgguggcuccugcagaa............................................................................    2       1    seq
..............caauugguggcuUcugcagaa............................................................................    34      1    seq
..............caauuggCggcuccugcagaa............................................................................    4       1    seq
..............caauugguggUuccugcagaa............................................................................    4       1    seq
..............caauugguggcCccugcagaa............................................................................    26      1    seq
..............caauuggugAcuccugcagaa............................................................................    8       1    seq
..............caauugguggcucAugcagaa............................................................................    8       1    seq
..............caauugUuggcuccugcagaa............................................................................    8       1    seq
..............caauugguggcuccugcagaa............................................................................    10600   0    seq
..............caauugguggcuccugUagaa............................................................................    6       1    seq
..............caauugguggcuccugcagGa............................................................................    8       1    seq
..............caauugguggcuccugcCgaa............................................................................    6       1    seq
..............caauugguggcuccugcagaC............................................................................    2       1    seq
..............cCauugguggcuccugcagaa............................................................................    14      1    seq
..............caauugguAgcuccugcagaa............................................................................    2       1    seq
..............caaCugguggcuccugcagaa............................................................................    8       1    seq
..............caauugguggcuccugcaCaa............................................................................    2       1    seq
..............caauAgguggcuccugcagaa............................................................................    16      1    seq
..............Naauugguggcuccugcagaa............................................................................    111     1    seq
..............caauugguggcuccugcagaU............................................................................    72      1    seq
..............caauugAuggcuccugcagaa............................................................................    82      1    seq
..............caauugguggAuccugcagaa............................................................................    6       1    seq
..............caauugguggcucGugcagaa............................................................................    2       1    seq
..............caauugguggcucUugcagaa............................................................................    6       1    seq
..............caauugguggcuccugcGgaa............................................................................    2       1    seq
..............caGuugguggcuccugcagaa............................................................................    4       1    seq
..............caauugguggcuccCgcagaa............................................................................    24      1    seq
..............caauuUguggcuccugcagaa............................................................................    30      1    seq
..............caauugguCgcuccugcagaa............................................................................    10      1    seq
..............caauugguggcuccugAagaa............................................................................    4       1    seq
..............caauugguggcuccugcaAaa............................................................................    2       1    seq
..............caauugguggcuccugcagaG............................................................................    6       1    seq
..............caauCgguggcuccugcagaa............................................................................    16      1    seq
..............caauugguggcuccugcagCa............................................................................    4       1    seq
..............caauugguggcuAcugcagaa............................................................................    6       1    seq
..............caauugguggcuccAgcagaa............................................................................    2       1    seq
..............caauuggugUcuccugcagaa............................................................................    8       1    seq
..............caauugguggcuccugGagaa............................................................................    6       1    seq
```

Star **Mature**

```
gcuccugcagaagccaauugguggcuccugcagaagaaaauugguggcuccugcagaagccaauugguggcuccugcagaagaucagucccuuaaguaaaugaugguccac
..............caauugguggcuccugcagaag...........................................................................  98  0  seq
..............Uaauugguggcuccugcagaag...........................................................................  3  1  seq
..............cGauugguggcuccugcagaag...........................................................................  2  1  seq
..............caaCugguggcuccugcagaag...........................................................................  2  1  seq
..............caauugguggcuccugcagaaC...........................................................................  14  1  seq
..............Gaauugguggcuccugcagaag...........................................................................  3  1  seq
..............caauugguggcuccugcagaaAa..........................................................................  14  1  seq
..............caauugguggcuccugcagaaCa..........................................................................  4  1  seq
..............caauugguggcuccugcagaaga..........................................................................  12  0  seq
..............caauugguggcuccugcagaagU..........................................................................  16  1  seq
..............caauugguggcuccugcagaagUa.........................................................................  1  1  seq
..............caauugguggcuccugcagaaAaa.........................................................................  2  1  seq
..............caauugguggcuccugcagaagaG.........................................................................  4  1  seq
..............caauugguggcuccugcagaagaa.........................................................................  2  0  seq
..............caauugguggcuccugcagaagUaa........................................................................  1  1  seq
...............aauugguggcuccugcag..............................................................................  48  0  seq
...............aauugguggcuccugcCg..............................................................................  3  1  seq
...............aauuggCggcuccugcaga.............................................................................  3  1  seq
...............aauGgguggcuccugcaga.............................................................................  3  1  seq
...............aauugguggcuccugcaga.............................................................................  1047  0  seq
...............aauugguggcuccugAaga.............................................................................  3  1  seq
...............aauugguggcuccCgcaga.............................................................................  3  1  seq
...............aauugguggcuccugcagC.............................................................................  6  1  seq
...............aCuugguggcuccugcaga.............................................................................  6  1  seq
...............Nauugguggcuccugcaga.............................................................................  9  1  seq
...............aauugguggcuccugGagaa............................................................................  3  1  seq
...............Nauugguggcuccugcagaa............................................................................  9  1  seq
...............aauugguggcuccugcCgaa............................................................................  3  1  seq
...............aauuCguggcuccugcagaa............................................................................  3  1  seq
...............aauugguggcucUugcagaa............................................................................  3  1  seq
...............aauugguggcuccugcagaa............................................................................  1818  0  seq
...............aauuggugAcuccugcagaa............................................................................  3  1  seq
...............aauugguggAuccugcagaa............................................................................  3  1  seq
...............aauugguggcuccuUcagaa............................................................................  3  1  seq
...............aUuugguggcuccugcagaa............................................................................  3  1  seq
...............aaAugguggcuccugcagaa............................................................................  3  1  seq
...............aauugguggcuccugcagaG............................................................................  3  1  seq
...............aauugguggcuccugcagaCg...........................................................................  12  1  seq
...............aauugguggcuccugcUgaag...........................................................................  3  1  seq
...............aauugguggcuccugAagaag...........................................................................  3  1  seq
...............aauugguggcuccugcaCaag...........................................................................  21  1  seq
...............aauugguggUuccugcagaag...........................................................................  24  1  seq
...............aauugguggcuccugcagaUg...........................................................................  12  1  seq
...............aauugguggcuccugcaAaag...........................................................................  15  1  seq
...............Nauugguggcuccugcagaag...........................................................................  264  1  seq
...............aauuggCggcuccugcagaag...........................................................................  48  1  seq
...............aauugguggcuccugUagaag...........................................................................  21  1  seq
...............aauuggAggcuccugcagaag...........................................................................  9  1  seq
...............aauugguggcuccugcagGag...........................................................................  33  1  seq
...............aauugguggcuccCgcagaag...........................................................................  63  1  seq
...............aauugguCgcuccugcagaag...........................................................................  54  1  seq
...............aauugguggcuGcugcagaag...........................................................................  3  1  seq
...............aauugUuggcuccugcagaag...........................................................................  15  1  seq
...............aauuggGggcuccugcagaag...........................................................................  3  1  seq
...............aGuugguggcuccugcagaag...........................................................................  21  1  seq
...............aauuggugUcuccugcagaag...........................................................................  9  1  seq
...............aauugguggcuccugcagaaC...........................................................................  27  1  seq
...............aauCgguggcuccugcagaag...........................................................................  6  1  seq
...............aauugguggcuccugcagCag...........................................................................  84  1  seq
...............aauugguggcuccugcagUag...........................................................................  3  1  seq
...............aUuugguggcuccugcagaag...........................................................................  12  1  seq
...............aauugguggcuccugcCgaag...........................................................................  54  1  seq
...............aauuUguggcuccugcagaag...........................................................................  123  1  seq
...............aauugguggcuccGgcagaag...........................................................................  3  1  seq
...............aauugguggcuccuUcagaag...........................................................................  33  1  seq
...............aauugguUgcuccugcagaag...........................................................................  3  1  seq
...............aauAgguggcuccugcagaag...........................................................................  15  1  seq
...............aauugguggcuccuAcagaag...........................................................................  57  1  seq
...............aaAugguggcuccugcagaag...........................................................................  18  1  seq
...............aauuCguggcuccugcagaag...........................................................................  81  1  seq
```

Star Mature

gcuccugcagaagccaauugguggcuccugcagaagaaaauugguggcuccugcagaagccaauugguggcuccugcagaagaucagucccuuaaguaaaugaugguccac			
...............aauugguggGuccugcagaag...	9	1	seq
...............aauGgguggcuccugcagaag...	9	1	seq
...............aauuggugCcuccugcagaag...	6	1	seq
...............aauugguggcuccugcGgaag...	30	1	seq
...............aCuugguggcuccugcagaag...	21	1	seq
...............Uauugguggcuccugcagaag...	12	1	seq
...............aauugguggcAccugcagaag...	3	1	seq
...............aauugguggcuUcugcagaag...	33	1	seq
...............aaGugguggcuccugcagaag...	3	1	seq
...............aauugguggAuccugcagaag...	12	1	seq
...............Cauugguggcuccugcagaag...	3	1	seq
...............aauugguggcCccugcagaag...	9	1	seq
...............aauugguggcuccugcagaag...	71832	0	seq
...............aauuAguggcuccugcagaag...	78	1	seq
...............Gauugguggcuccugcagaag...	24	1	seq
...............aauuggugAcuccugcagaag...	72	1	seq
...............aauugguggcuccugcaUaag...	9	1	seq
...............aauugguggcuAcugcagaag...	3	1	seq
...............aauugguggcucUugcagaag...	24	1	seq
...............aauugAuggcuccugcagaag...	30	1	seq
...............aauugguggcuccAgcagaag...	9	1	seq
...............aauugguggcucAugcagaag...	3	1	seq
...............aaCugguggcuccugcagaag...	3	1	seq
...............aauugguAgcuccugcagaag...	105	1	seq
...............aauugguggcuccugcagaGg...	15	1	seq
...............aauugguggcuccuCcagaag...	27	1	seq
...............aauugguggcuccugcagaaga..	58	0	seq
...............aauugguggcuccugcagaagU..	798	1	seq
...............aUuugguggcuccugcagaaga..	2	1	seq
...............aauugguggcuccugcagaagG..	12	1	seq
...............aauugguggcuccugcagaagUa...	1	1	seq
...............aauugguggcuccugcagaagaC...	2	1	seq
...............aauugguggcuccugcagaaAaa...	1	1	seq
...............aauugguggcuccugcagaagaa...	1	0	seq
...............aauugguggcuccugcagaagaGa..	2	1	seq
...............aauugguggcuccugcagaagaaa..	1	0	seq
...............aauugguggcuccugcagaagaGaa...	1	1	seq
................auugguggcuccugcaga...	15	0	seq
................auugguggcuccuUcagaa..	3	1	seq
................auugguggcuccugcagaa..	105	0	seq
................auugCuggcuccugcagaa..	3	1	seq
................auugguggcCccugcagaa..	3	1	seq
................auugAuggcuccugcagaag...	3	1	seq
................auugguggcuccugcagaag...	276	0	seq
................auugguggcuccugcagaaga..	928	0	seq
................aAugguggcuccugcagaaga..	2	1	seq
................auugAuggcuccugcagaaga..	2	1	seq
................auugguggcuccugGagaaga..	2	1	seq
................aCugguggcuccugcagaaga..	2	1	seq
................Nuugguggcuccugcagaaga..	4	1	seq
................auugguggcCccugcagaaga..	2	1	seq
................auugguggcuccugcagaagU..	15	1	seq
................auugguggcuccugUagaaga..	2	1	seq
................auugguggcuccCgcagaaga..	4	1	seq
................auugguAgcuccugcagaaga..	2	1	seq
.................uugguggcuccugcagaa..	15	0	seq
.................uugguggcuccugUagaag...	3	1	seq
.................Augguggcuccugcagaag...	3	1	seq
.................uugguggcuccugcagaag...	54	0	seq
.................uugguggcuccugcagaaga..	6	0	seq
.................uugguggcuccugcagaagaC...	4	1	seq
.................uugguggcuccugcagaagaa...	99	0	seq
.................uugguAgcuccugcagaagaa...	1	1	seq
.................uugguggcuccugcagaagaaG..	1	1	seq
.................uugguggcuccugcagaagaaU..	2	1	seq
..................ugguggcuccugcagaCg...	3	1	seq
..................uggugAcuccugcagaag...	3	1	seq
..................ugguCgcuccugcagaag...	3	1	seq
..................ugguggcuccugcagaag...	183	0	seq
..................ugguggcuccugcagaaga..	2	0	seq

Star **Mature**

gcuccugcagaagccaauugguggcuccugcagaagaaaauugguggcuccugcagaagccaauugguggcuccugcagaagaucagucccuuaaguaaaugaugguccac

..................ugguggcuccugcagaagU..	6	1	seq
..................ugguggcuccugcagaagaa...	2	0	seq
..................ugguUgcuccugcagaagaaa..	1	1	seq
..................ugguggcuccugcCgaagaaa..	1	1	seq
..................ugguggcuccugcagaagaCa..	1	1	seq
..................ugguggcuccugcagaagaaa..	48	0	seq
..................ugguggcuccugcagaagCaa..	1	1	seq
..................Ngguggcuccugcagaagaaa..	1	1	seq
..................ugguggcCccugcagaagaaa..	1	1	seq
...................gguggcuccugcagaagaa...	1	0	seq
...................gguggcuccugcagaagaaa..	2	0	seq
...................gguggcuccugcagaagaaaa...	7	0	seq
...................gguggcuccugcagaagCaaa...	1	1	seq
....................guggcuccugcagaagaaa..	1	0	seq
....................guggcuccCgcagaagaaa..	1	1	seq
....................guggcuccugcagaagCaaa...	1	1	seq
....................guggcuccugcagaagaaaa...	1	0	seq
....................guggcuccugcagaagaaaau..	53	0	seq
....................guggcuccugcagaagCaaau..	3	1	seq
....................guggcuccugcagaagaaaauuU..	1	1	seq
....................Uuggcuccugcagaagaaaauug..	1	1	seq
.....................uggcuccugcagaagaaaauu...	5	0	seq
......................ggcuccugcagaagaaaauug..	1	0	seq
.......................gcuccugcagaagaaaauugg...	2	0	seq
.......................Acuccugcagaagaaaauugg...	1	1	seq
.......................gUuccugcagaagaaaauugg...	1	1	seq
........................cuccugcagaagCaaauuggu..	1	1	seq
........................cuccugcagaagaaaauuggu..	9	0	seq
.........................uccugcagaagaaaauuggug...	3	0	seq
..........................ccugcagaagCaaauuggugg..	2	1	seq
..........................ccugcagaagaaaauuggugg..	2	0	seq
...........................cugcagaagaaaauugguggc...	5	0	seq
...........................cugcagaagCaaauugguggc...	1	1	seq
............................ugcagaagCaaauugguggcu..	1	1	seq
............................ugcagaagaaaauugguggcu..	1	0	seq
.............................gcagaagCaaauugguggcuc...	1	1	seq
.............................gcagaagaaaauugguggcuc...	3	0	seq
..............................cagaagaaaauugguggcuc...	1	0	seq
..............................cagaagCaaauugguggcuc...	1	1	seq
..............................cagaagCaaauugguggcucc..	1	1	seq
..............................cagaagaaaauugguggcucc..	10	0	seq
..............................Uagaagaaaauugguggcucc..	1	1	seq
...............................agaagaaaauugguggcu..	1	0	seq
...............................agaagaaaauugguggcucc..	1	0	seq
...............................agaagaaaauugguggcuccu...	8	0	seq
...............................agaagCaaauugguggcuccu...	1	1	seq
...............................agaaUaaaauugguggcuccu...	1	1	seq
................................gaagaaaauugguggcuccu...	1	0	seq
................................gaagaaaauugguggcuccug..	9	0	seq
.................................aagaaaauugguggcuccug..	1	0	seq
.................................aagCaaauugguggcuccugc...	8	1	seq
.................................aagaaaauugguggcuccugc...	13	0	seq
..................................agCaaauugguggcuccugca..	3	1	seq
..................................agaCaauugguggcuccugca..	1	1	seq
..................................agaaaauugguggcuccugca..	18	0	seq
..................................aAaaaauugguggcuccugcagaag..	1	1	seq
...................................gaaaauugguggcuccugc...	1	0	seq
...................................Naaaauugguggcuccugcag...	1	1	seq
...................................gaaaauugguggcuccugcag...	10	0	seq
...................................gCaaauugguggcuccugcag...	2	1	seq
...................................gaaaauugAuggcuccugcag...	1	1	seq
...................................Aaaaauugguggcuccugcaga..	1	1	seq
...................................gaaaauugguggcuccugcagaag..	1	0	seq
....................................aaaauugguggcuccugca..	1	0	seq
....................................Caaauugguggcuccugcag...	1	1	seq
....................................aaaauugguggcuccugcag...	3	0	seq
....................................aaaauuggAggcuccugcaga..	1	1	seq
....................................aaaauugguggcuccugcaga..	50	0	seq
....................................Naaauugguggcuccugcaga..	1	1	seq
....................................Caaauugguggcuccugcaga..	7	1	seq

Star **Mature**

```
gcuccugcagaagccaauugguggcuccugcagaagaaaauugguggcuccugcagaagccaauugguggcuccugcagaagaucagucccuuaaguaaaugaugguccac
....................................aCaauugguggcuccugcaga......................................................   1      1   seq
....................................aCaauugguggcuccugcagaa.....................................................   1      1   seq
.....................................aaauugguggcuccugcag.......................................................   6      0   seq
.....................................Naauugguggcuccugcag.......................................................   6      1   seq
.....................................Gaauugguggcuccugcaga......................................................   3      1   seq
.....................................aaauugguggcuccugcaga......................................................   16     0   seq
.....................................Naauugguggcuccugcaga......................................................   6      1   seq
.....................................aaauugguggcuAcugcagaa.....................................................   1      1   seq
.....................................aaauugguggcuccugcagaa.....................................................   205    0   seq
.....................................aaauuCguggcuccugcagaa.....................................................   1      1   seq
.....................................Naauugguggcuccugcagaa.....................................................   111    1   seq
.....................................aUauugguggcuccugcagaag....................................................   1      1   seq
.....................................aaauugguggcuccugcagaaU....................................................   9      1   seq
.....................................Gaauugguggcuccugcagaag....................................................   3      1   seq
.....................................aaUuugguggcuccugcagaag....................................................   3      1   seq
.....................................Uaauugguggcuccugcagaag....................................................   3      1   seq
.....................................aaauugguggcuccugcagaaC....................................................   1      1   seq
.....................................aaauugguggcuccugcagaaA....................................................   1      1   seq
.....................................aaauugguggcuccugcagaagcca.................................................   1      0   seq
.....................................aaauugguggcuccugcagaaAcca.................................................   2      1   seq
.....................................Uaauugguggcuccugcagaagcca.................................................   1      1   seq
......................................aauugguggcuccugcCg.......................................................   3      1   seq
......................................aauugguggcuccugcag.......................................................   48     0   seq
......................................aauugguggcuccugcaga......................................................   1047   0   seq
......................................aauuggCggcuccugcaga......................................................   3      1   seq
......................................aCuugguggcuccugcaga......................................................   6      1   seq
......................................aauugguggcuccugAaga......................................................   3      1   seq
......................................aauugguggcuccugcagC......................................................   6      1   seq
......................................aauugguggcuccCgcaga......................................................   3      1   seq
......................................aauGgguggcuccugcaga......................................................   3      1   seq
......................................Nauugguggcuccugcaga......................................................   9      1   seq
......................................aauuCguggcuccugcagaa.....................................................   3      1   seq
......................................aauugguggcuccugGagaa.....................................................   3      1   seq
......................................aauuggugAcuccugcagaa.....................................................   3      1   seq
......................................aauugguggcuccugcCgaa.....................................................   3      1   seq
......................................aauugguggcuccugcagaG.....................................................   3      1   seq
......................................aUuugguggcuccugcagaa.....................................................   3      1   seq
......................................aauugguggcuccuUcagaa.....................................................   3      1   seq
......................................aauugguggAuccugcagaa.....................................................   3      1   seq
......................................aauugguggcucUugcagaa.....................................................   3      1   seq
......................................aaAugguggcuccugcagaa.....................................................   3      1   seq
......................................aauugguggcuccugcagaa.....................................................   1818   0   seq
......................................Nauugguggcuccugcagaa.....................................................   9      1   seq
......................................aaAugguggcuccugcagaag....................................................   18     1   seq
......................................aauugguggcuUcugcagaag....................................................   33     1   seq
......................................aauuUguggcuccugcagaag....................................................   123    1   seq
......................................aauugguggcuccugcUgaag....................................................   3      1   seq
......................................aauugguCgcuccugcagaag....................................................   54     1   seq
......................................aauugguggcuccugcCgaag....................................................   54     1   seq
......................................aauuCguggcuccugcagaag....................................................   81     1   seq
......................................aauuggugCcuccugcagaag....................................................   6      1   seq
......................................aauugguggUuccugcagaag....................................................   24     1   seq
......................................aauugguggcuccugcGgaag....................................................   30     1   seq
......................................aauGgguggcuccugcagaag....................................................   9      1   seq
......................................aauugguggcucUugcagaag....................................................   24     1   seq
......................................Nauugguggcuccugcagaag....................................................   264    1   seq
......................................aauuggCggcuccugcagaag....................................................   48     1   seq
......................................Uauugguggcuccugcagaag....................................................   12     1   seq
......................................aauugguggcuccugcagaUg....................................................   12     1   seq
......................................aauugguggcuGcugcagaag....................................................   3      1   seq
......................................aauugguggAuccugcagaag....................................................   12     1   seq
......................................aauugguggcuccugAagaag....................................................   3      1   seq
......................................aauugguggcuccuCcagaag....................................................   27     1   seq
......................................aauuggAggcuccugcagaag....................................................   9      1   seq
......................................aauugguggcuAcugcagaag....................................................   3      1   seq
......................................aaGugguggcuccugcagaag....................................................   3      1   seq
......................................Gauugguggcuccugcagaag....................................................   24     1   seq
......................................aauAgguggcuccugcagaag....................................................   15     1   seq
......................................aauugguggcuccugcagaCg....................................................   12     1   seq
......................................aauugAuggcuccugcagaag....................................................   30     1   seq
```

Star **Mature**

```
gcuccugcagaagccaauugguggcuccugcagaagaaaauugguggcuccugcagaagccaauugguggcuccugcagaagaucagucccuuaaguaaaugaugguccac
......................................aauCgguggcuccugcagaag....................................................  6      1  seq
......................................aauugguggcuccuAcagaag....................................................  57     1  seq
......................................aaCugguggcuccugcagaag....................................................  3      1  seq
......................................aauuggugUcuccugcagaag....................................................  9      1  seq
......................................aauugguggcuccGgcagaag....................................................  3      1  seq
......................................aauugguggcuccugcagCag....................................................  84     1  seq
......................................aGuugguggcuccugcagaag....................................................  21     1  seq
......................................aauuggugAcuccugcagaag....................................................  72     1  seq
......................................aauugguggcuccugcagUag....................................................  3      1  seq
......................................aauuggGggcuccugcagaag....................................................  3      1  seq
......................................aauugguggcuccugcagGag....................................................  33     1  seq
......................................aauugguggcuccCgcagaag....................................................  63     1  seq
......................................aauugguggcuccugcaUaag....................................................  9      1  seq
......................................aUuugguggcuccugcagaag....................................................  12     1  seq
......................................aauugguggcuccugcagaag....................................................  71832  0  seq
......................................aauugguAgcuccugcagaag....................................................  105    1  seq
......................................aauugUuggcuccugcagaag....................................................  15     1  seq
......................................aauugguggcCccugcagaag....................................................  9      1  seq
......................................aauugguUgcuccugcagaag....................................................  3      1  seq
......................................aauugguggcuccugcagaaC....................................................  27     1  seq
......................................aauugguggGuccugcagaag....................................................  9      1  seq
......................................aauuAguggcuccugcagaag....................................................  78     1  seq
......................................aauugguggcuccAgcagaag....................................................  9      1  seq
......................................aauugguggcucAugcagaag....................................................  3      1  seq
......................................aCuugguggcuccugcagaag....................................................  21     1  seq
......................................aauugguggcuccugUagaag....................................................  21     1  seq
......................................aauugguggcuccugcaCaag....................................................  21     1  seq
......................................aauugguggcAccugcagaag....................................................  3      1  seq
......................................aauugguggcuccugcagaGg....................................................  15     1  seq
......................................Cauugguggcuccugcagaag....................................................  3      1  seq
......................................aauugguggcuccuUcagaag....................................................  33     1  seq
......................................aauugguggcuccugcaAaag....................................................  15     1  seq
......................................Cauugguggcuccugcagaagc...................................................  2      1  seq
......................................aauugguggcuccugcagaagG...................................................  12     1  seq
......................................aauugguggcuccugcagaagU...................................................  798    1  seq
......................................aauugguggcuccugcagaagc...................................................  39     0  seq
......................................aauugguggcuccugcagaagcc..................................................  3      0  seq
......................................aauugguggcuccugcagaagAc..................................................  1      1  seq
......................................aauugguggcuccugcagaagcca.................................................  3      0  seq
......................................aUuugguggcuccugcagaagccaa................................................  1      1  seq
.......................................auugguggcuccugcaga......................................................  15     0  seq
.......................................auugguggcuccugcagaa.....................................................  105    0  seq
.......................................auugguggcuccuUcagaa.....................................................  3      1  seq
.......................................auugguggcCccugcagaa.....................................................  3      1  seq
.......................................auugCuggcuccugcagaa.....................................................  3      1  seq
.......................................auugguggcuccugcagaag....................................................  276    0  seq
.......................................auugAuggcuccugcagaag....................................................  3      1  seq
.......................................aAugguggcuccugcagaagc...................................................  5      1  seq
.......................................auugguggcuccugcGgaagc...................................................  1      1  seq
.......................................auugguggcCccugcagaagc...................................................  1      1  seq
.......................................auugguggcuccugcagaCgc...................................................  3      1  seq
.......................................auugguggcuccuAcagaagc...................................................  1      1  seq
.......................................auugguggcuccugcCgaagc...................................................  5      1  seq
.......................................auugguggcuccuCcagaagc...................................................  2      1  seq
.......................................auugguggcuUcugcagaagc...................................................  1      1  seq
.......................................auugAuggcuccugcagaagc...................................................  5      1  seq
.......................................auugguggcuccugcagaagc...................................................  2665   0  seq
.......................................auugCuggcuccugcagaagc...................................................  4      1  seq
.......................................auuggCggcuccugcagaagc...................................................  2      1  seq
.......................................auugguggcuccCgcagaagc...................................................  6      1  seq
.......................................auugguggcuccugcagaagU...................................................  15     1  seq
.......................................auuggugAcuccugcagaagc...................................................  6      1  seq
.......................................auugguggcuccuUcagaagc...................................................  5      1  seq
.......................................auugguggcuGcugcagaagc...................................................  1      1  seq
.......................................auugUuggcuccugcagaagc...................................................  3      1  seq
.......................................auugguggcuccugcagaaCc...................................................  2      1  seq
.......................................auugguAgcuccugcagaagc...................................................  4      1  seq
.......................................auugguggcuccugUagaagc...................................................  3      1  seq
.......................................auugguggcuccugcagCagc...................................................  1      1  seq
.......................................auugguggcuAcugcagaagc...................................................  1      1  seq
```

Star **Mature**

```
gcuccugcagaagccaauugguggcuccugcagaagaaaauugguggcuccugcagaagccaauugguggcuccugcagaagaucagucccuuaaguaaaugaugguccac
.......................................auugguCgcuccugcagaagc...................................................   8     1   seq
.......................................auuAguggcuccugcagaagc...................................................   2     1   seq
.......................................Nuugguggcuccugcagaagc...................................................   8     1   seq
.......................................aCugguggcuccugcagaagc...................................................   2     1   seq
.......................................Guugguggcuccugcagaagc...................................................   1     1   seq
.......................................auugguggcuccugcagaaUc...................................................   1     1   seq
.......................................auugguggcuccugcagaagcU..................................................   11    1   seq
.......................................Uuugguggcuccugcagaagcc..................................................   1     1   seq
.......................................auugguggcuccugcagaagcc..................................................   6     0   seq
........................................uugguggcuccugcagaa.....................................................   15    0   seq
........................................Augguggcuccugcagaag....................................................   3     1   seq
........................................uugguggcuccugUagaag....................................................   3     1   seq
........................................uugguggcuccugcagaag....................................................   54    0   seq
........................................uugguggcuccugcagaagc...................................................   55    0   seq
........................................uugguggcucUugcagaagc...................................................   1     1   seq
........................................uugguggcucAugcagaagcc..................................................   1     1   seq
........................................uuggGggcuccugcagaagcc..................................................   2     1   seq
........................................uugguggcuUcugcagaagcc..................................................   1     1   seq
........................................uugguggcuccugcagaaCcc..................................................   1     1   seq
........................................Nugguggcuccugcagaagcc..................................................   5     1   seq
........................................uugguggcuccugcagaagcc..................................................   1658  0   seq
........................................uugguggcuccugcCgaagcc..................................................   2     1   seq
........................................uugguggcuccugcagaagcG..................................................   1     1   seq
........................................uugguggcuccugcagaGgcc..................................................   1     1   seq
........................................uugguggcuccugcagaagAc..................................................   2     1   seq
........................................uuggAggcuccugcagaagcc..................................................   1     1   seq
........................................Cugguggcuccugcagaagcc..................................................   1     1   seq
........................................uugguggcuccugcagaagcU..................................................   2     1   seq
........................................uuggCggcuccugcagaagcc..................................................   3     1   seq
........................................uuggugAcuccugcagaagcc..................................................   1     1   seq
........................................uugguAgcuccugcagaagcc..................................................   1     1   seq
........................................uugguggcuccCgcagaagcc..................................................   3     1   seq
........................................uugguggcuccugcagaagccU.................................................   4     1   seq
.........................................ugguggcuccugcagaag....................................................   183   0   seq
.........................................ugguCgcuccugcagaag....................................................   3     1   seq
.........................................ugguggcuccugcagaCg....................................................   3     1   seq
.........................................uggugAcuccugcagaag....................................................   3     1   seq
.........................................ugguggcuccugcagaagc...................................................   4     0   seq
.........................................ugguggcuccugcagaagU...................................................   6     1   seq
.........................................ugguggcuccugcagaagcc..................................................   36    0   seq
.........................................ugguggcuccugcagCagcc..................................................   1     1   seq
.........................................ugguAgcuccugcagaagcca.................................................   1     1   seq
.........................................ugguggcuccugcagaagAca.................................................   1     1   seq
.........................................ugguggcuccugcagaagcUa.................................................   1     1   seq
.........................................ugguggcuccugcagaagcAa.................................................   1     1   seq
.........................................ugguggcuccugcagaagcca.................................................   58    0   seq
.........................................ugguggcuccugcagaagccaU................................................   1     1   seq
.........................................ugguggcuccugcagaagccaUu...............................................   1     1   seq
..........................................gguggcuccugcagaagc...................................................   38    0   seq
..........................................gguggcuccugcGgaagc...................................................   1     1   seq
..........................................gguggcuccugcaUaagc...................................................   1     1   seq
..........................................gguggcuccugcagaagcU..................................................   1     1   seq
..........................................gguggcuccugcagaagccaC................................................   3     1   seq
..........................................gguggcuccugcagaagccaa................................................   7     0   seq
..........................................gguCgcuccugcagaagccaa................................................   1     1   seq
..........................................gguggcuccugcagaagcAaa................................................   1     1   seq
..........................................gguggcuccugcagaagccaau...............................................   1     0   seq
...........................................guggcuccugcagaagcc..................................................   2     0   seq
...........................................guggcuccCgcagaagcc..................................................   1     1   seq
...........................................guggcuccugcagaagcAaa................................................   1     1   seq
...........................................gugAcuccugcagaagccaa................................................   1     1   seq
...........................................guggcuccugcagaagccaa................................................   1     0   seq
...........................................guCgcuccugcagaagccaau...............................................   1     1   seq
...........................................guggcuccugcagaagcAaau...............................................   3     1   seq
...........................................guggcuccugcagaagccaau...............................................   11    0   seq
...........................................guggcuccugcagaagccaauu..............................................   3     0   seq
...........................................guggcuccugcagaagccaauA..............................................   1     1   seq
............................................uggcuccugcagaagccaauu..............................................   1     0   seq
.............................................Ugcuccugcagaagccaauug.............................................   1     1   seq
.............................................ggcuccugcagaagccaauug.............................................   13    0   seq
```

Star **Mature**

```
gcuccugcagaagccaauugguggcuccugcagaagaaaauugguggcuccugcagaagccaauugguggcuccugcagaagaucagucccuuaaguaaaugaugguccac
..............................................Acuccugcagaagccaauu..............................................   2     1   seq
..............................................gcuccugcagaagccaauug.............................................   6     0   seq
..............................................Acuccugcagaagccaauugg............................................   6     1   seq
..............................................gcuccugcagaUgccaauugg............................................   2     1   seq
..............................................gcuccugcCgaagccaauugg............................................   2     1   seq
..............................................gcuccugcagaagccaauugg............................................   162   0   seq
...............................................cuccugcagaagccaauu..............................................   2     0   seq
...............................................cuccugcagaagccaauugg............................................   4     0   seq
...............................................cuccugcagaagcAaauuggu...........................................   2     1   seq
...............................................cuccugcagaagccaauuAgu...........................................   2     1   seq
...............................................cuUcugcagaagccaauuggu...........................................   4     1   seq
...............................................cuccugcagaagccaauuggu...........................................   190   0   seq
...............................................cuccugcagaagccaauugguU..........................................   2     1   seq
................................................uccugcagaagccaauuggu...........................................   2     0   seq
................................................uccugcagaagccaauuggCg..........................................   2     1   seq
................................................uccugcagaagccaauuggug..........................................   110   0   seq
................................................Nccugcagaagccaauuggug..........................................   2     1   seq
................................................uccuUcagaagccaauuggug..........................................   2     1   seq
................................................Accugcagaagccaauuggug..........................................   6     1   seq
................................................uccugcagaagccaauuggugU.........................................   8     1   seq
.................................................ccugcagaagccaauuggug..........................................   2     0   seq
.................................................ccugcagaagccaaCuggugg.........................................   2     1   seq
.................................................ccCgcagaagccaauuggugg.........................................   2     1   seq
.................................................Ucugcagaagccaauuggugg.........................................   2     1   seq
.................................................ccugcagaagccaauuggugg.........................................   28    0   seq
.................................................ccugcagaagcAaauuggugg.........................................   4     1   seq
..................................................cugcagaagccaauuggug..........................................   4     0   seq
..................................................cugcagaagccaauuggugg.........................................   8     0   seq
..................................................cugcagaagcAaauugguggc........................................   2     1   seq
..................................................cugcagaagccaauugguggc........................................   68    0   seq
..................................................cugcagaagccaauugguggcuU......................................   2     1   seq
...................................................ugcagaagccaauuggug..........................................   6     0   seq
...................................................Cgcagaagccaauuggugg.........................................   2     1   seq
...................................................ugcagaagccaauuggugg.........................................   8     0   seq
...................................................uAcagaagccaauugguggc........................................   2     1   seq
...................................................ugcagaagccaauugguggc........................................   144   0   seq
...................................................ugcagaagccCauugguggcu.......................................   2     1   seq
...................................................ugcagaagccaauugguggcG.......................................   2     1   seq
...................................................ugcagaagccaauugguggcu.......................................   356   0   seq
...................................................ugcagaagccaauuAguggcu.......................................   2     1   seq
...................................................ugcagaagcAaauugguggcu.......................................   2     1   seq
...................................................ugcagaagccaauugguggcC.......................................   2     1   seq
...................................................ugcagUagccaauugguggcu.......................................   2     1   seq
...................................................ugcagaagccaauugguggcuc......................................   2     0   seq
...................................................ugcagaagccaauugguggcuU......................................   116   1   seq
....................................................gcagaagccaauugguggc........................................   2     0   seq
....................................................gcagaagccaauugguggcu.......................................   6     0   seq
....................................................gcagaagcUaauugguggcuc......................................   2     1   seq
....................................................gcagUagccaauugguggcuc......................................   2     1   seq
....................................................gcagaagccaauugguggcuc......................................   102   0   seq
....................................................gcagaagcAaauugguggcuc......................................   2     1   seq
....................................................gcagaagccaauugguggcucU.....................................   6     1   seq
.....................................................cagaagcAaauugguggcuc......................................   2     1   seq
.....................................................cagaagccaauugguggcucc.....................................   18    0   seq
.....................................................cagaagcAaauugguggcucc.....................................   2     1   seq
......................................................agaagccaauugguggcuccu....................................   8     0   seq
......................................................agaagcAaauugguggcuccu....................................   2     1   seq
.......................................................gaagccaauugguggcuccug...................................   6     0   seq
........................................................aagccaauugguggcuccug...................................   2     0   seq
........................................................aagcAaauugguggcuccugc..................................   16    1   seq
........................................................aagccGauugguggcuccugc..................................   2     1   seq
........................................................aagccaauugguggcuccugc..................................   102   0   seq
........................................................aagccaauugguggcuccCgc..................................   2     1   seq
........................................................aagcUaauugguggcuccugc..................................   2     1   seq
........................................................aagccaauuggCggcuccugc..................................   2     1   seq
........................................................aGgccaauugguggcuccugc..................................   2     1   seq
........................................................aagccaauugguggcuccugcU.................................   4     1   seq
.........................................................agccaauugguggcuccugc..................................   2     0   seq
.........................................................agcAaauugguggcuccugca.................................   6     1   seq
.........................................................agccaauugguggcuccugca.................................   18    0   seq
```

Star **Mature**

```
gcuccugcagaagccaauugguggcuccugcagaagaaaauugguggcuccugcagaagccaauugguggcuccugcagaagaucagucccuuaaguaaaugaugguccac
........................................................agAcaauugguggcuccugca..................................  2     1  seq
..........................................................gccaauugguggcuccug...................................  2     0  seq
..........................................................gccaauugguggcuccugc..................................  2     0  seq
..........................................................gccaauugguggcuccuAcag................................  2     1  seq
..........................................................Nccaauugguggcuccugcag................................  2     1  seq
..........................................................gcAaauugguggcuccugcag................................  4     1  seq
..........................................................gccaauugguggcuccugcag................................  58    0  seq
..........................................................gccaauugguggcuccugcagU...............................  2     1  seq
..........................................................gUcaauugguggcuccugcagaa..............................  2     1  seq
..........................................................gccaauugguggcuccugcagaagU............................  2     1  seq
...........................................................ccaauugguggcuccugc..................................  2     0  seq
...........................................................ccaauugguggcuccugca.................................  6     0  seq
...........................................................ccaauugguggAuccugcag................................  2     1  seq
...........................................................ccaauugguggcuccCgcag................................  2     1  seq
...........................................................ccaauugguggcuccugcag................................  54    0  seq
...........................................................cAaauugguggcuccugcag................................  2     1  seq
...........................................................ccaauugguggcuUcugcaga...............................  6     1  seq
...........................................................ccaaAugguggcuccugcaga...............................  2     1  seq
...........................................................ccaauUgguggcuccugcaga...............................  4     1  seq
...........................................................Ncaauugguggcuccugcaga...............................  6     1  seq
...........................................................ccaauuggugAcuccugcaga...............................  2     1  seq
...........................................................ccaauugguUgcuccugcaga...............................  2     1  seq
...........................................................ccCauugguggcuccugcaga...............................  2     1  seq
...........................................................ccaauugguggcuccugcaga...............................  1084  0  seq
...........................................................ccaauugguggcuccugcGga...............................  2     1  seq
...........................................................ccaaCugguggcuccugcaga...............................  2     1  seq
...........................................................ccaauugguggcuccCgcaga...............................  2     1  seq
...........................................................ccaauugguggcCccugcaga...............................  2     1  seq
...........................................................cAaauugguggcuccugcaga...............................  14    1  seq
...........................................................Acaauugguggcuccugcaga...............................  2     1  seq
...........................................................ccaauugUuggcuccugcaga...............................  2     1  seq
...........................................................ccaauuggugUcuccugcaga...............................  2     1  seq
...........................................................ccaauugguggcuccugcagU...............................  4     1  seq
...........................................................Acaauugguggcuccugcagaa..............................  2     1  seq
...........................................................ccaauugguggcuccugcagaa..............................  2     0  seq
...........................................................ccaauugguggcuccugcagaU..............................  24    1  seq
...........................................................ccaauugguggcuccugcagaag.............................  2     0  seq
............................................................caauugguggcuccugca.................................  22    0  seq
............................................................caauuggugAcuccugca.................................  2     1  seq
............................................................Naauugguggcuccugcag................................  6     1  seq
............................................................caauugguggcuccugcag................................  356   0  seq
............................................................caauugguggcuUcugcag................................  2     1  seq
............................................................caauugAuggcuccugcag................................  4     1  seq
............................................................caauugguggcuccCgcag................................  2     1  seq
............................................................caaCugguggcuccugcag................................  2     1  seq
............................................................caauugguggcuccugcUg................................  2     1  seq
............................................................caauuggugAcuccugcaga...............................  2     1  seq
............................................................caauugguggcuccuUcaga...............................  2     1  seq
............................................................caauugguggcuccugcUga...............................  2     1  seq
............................................................caauugguggcuUcugcaga...............................  10    1  seq
............................................................caauugguggcuccugcCga...............................  2     1  seq
............................................................caauugguggcuccugcGga...............................  2     1  seq
............................................................caauuggCggcuccugcaga...............................  2     1  seq
............................................................caauugguggcCccugcaga...............................  4     1  seq
............................................................caauugguCgcuccugcaga...............................  6     1  seq
............................................................caauugguggcuccugcaga...............................  990   0  seq
............................................................caauugguggcucAugcaga...............................  2     1  seq
............................................................Gaauugguggcuccugcaga...............................  3     1  seq
............................................................caauugAuggcuccugcaga...............................  10    1  seq
............................................................caauugguggcuccugcagU...............................  8     1  seq
............................................................caauugguAgcuccugcaga...............................  2     1  seq
............................................................caauugguggcuccugUaga...............................  2     1  seq
............................................................Naauugguggcuccugcaga...............................  6     1  seq
............................................................cCauugguggcuccugcaga...............................  4     1  seq
............................................................caauUgguggcuccugcaga...............................  2     1  seq
............................................................caauugguggcucGugcagaa..............................  2     1  seq
............................................................caaCugguggcuccugcagaa..............................  8     1  seq
............................................................caauugguAgcuccugcagaa..............................  2     1  seq
............................................................cCauugguggcuccugcagaa..............................  14    1  seq
............................................................Naauugguggcuccugcagaa..............................  111   1  seq
```

Star **Mature**

```
gcuccugcagaagccaauugguggcuccugcagaagaaaauugguggcuccugcagaagccaauugguggcuccugcagaagaucagucccuuaaguaaaugaugguccac
............................................................caauugguggcuccugcGgaa..............................  2      1  seq
............................................................caauuggugAcuccugcagaa..............................  8      1  seq
............................................................caauugguggcCccugcagaa..............................  26     1  seq
............................................................caauugguggcuccugUagaa..............................  6      1  seq
............................................................caauAgguggcuccugcagaa..............................  16     1  seq
............................................................caauugguggcuccugAagaa..............................  4      1  seq
............................................................caauugguggcuccugcaAaa..............................  2      1  seq
............................................................caauugguggcuccugcagCa..............................  4      1  seq
............................................................caauugguggAuccugcagaa..............................  6      1  seq
............................................................caauugguggcuccugcCgaa..............................  6      1  seq
............................................................caauugguggUuccugcagaa..............................  4      1  seq
............................................................caauugguggcucUugcagaa..............................  6      1  seq
............................................................caauugguggcuccugcagaC..............................  2      1  seq
............................................................caauugguggcuccugGagaa..............................  6      1  seq
............................................................caauugguggcuccugcagGa..............................  8      1  seq
............................................................caauugguggcuccCgcagaa..............................  24     1  seq
............................................................caauugguggcuAcugcagaa..............................  6      1  seq
............................................................caauugguggcuUcugcagaa..............................  34     1  seq
............................................................caauugguggcuccugcagaa..............................  10600  0  seq
............................................................caGuugguggcuccugcagaa..............................  4      1  seq
............................................................caauugguCgcuccugcagaa..............................  10     1  seq
............................................................caauCgguggcuccugcagaa..............................  16     1  seq
............................................................caauugguggcuccugcaCaa..............................  2      1  seq
............................................................caauugguggcuccugcagaU..............................  72     1  seq
............................................................caauugUuggcuccugcagaa..............................  8      1  seq
............................................................caauugguggcuccugcagaG..............................  6      1  seq
............................................................caauuggCggcuccugcagaa..............................  4      1  seq
............................................................caauuUguggcuccugcagaa..............................  30     1  seq
............................................................caauugAuggcuccugcagaa..............................  82     1  seq
............................................................caauugguggcucAugcagaa..............................  8      1  seq
............................................................caauuggugUcuccugcagaa..............................  8      1  seq
............................................................caauugguggcuccAgcagaa..............................  2      1  seq
............................................................caauGgguggcuccugcagaa..............................  2      1  seq
............................................................Gaauugguggcuccugcagaag.............................  3      1  seq
............................................................Uaauugguggcuccugcagaag.............................  3      1  seq
............................................................caauugguggcuccugcagaag.............................  98     0  seq
............................................................caauugguggcuccugcagaaC.............................  14     1  seq
............................................................caaCugguggcuccugcagaag.............................  2      1  seq
............................................................cGauugguggcuccugcagaag.............................  2      1  seq
............................................................caauugguggcuccugcagaaCa............................  4      1  seq
............................................................caauugguggcuccugcagaaga............................  12     0  seq
............................................................caauugguggcuccugcagaagU............................  16     1  seq
............................................................caauugguggcuccugcagaaAa............................  14     1  seq
............................................................Naauugguggcuccugcagaagau...........................  1      1  seq
............................................................caauugguggcuccugcagaagaG...........................  4      1  seq
............................................................caauugguggcuccugcagaaAau...........................  1      1  seq
............................................................caauugguggcuccugcagaagau...........................  1      0  seq
............................................................caauugguggcuccugcagaagUu...........................  4      1  seq
............................................................caauugguggcuccugcagaagaCc..........................  1      1  seq
............................................................Aaauugguggcuccugcagaagauc..........................  1      1  seq
.............................................................aauugguggcuccugcag................................  48     0  seq
.............................................................aauugguggcuccugcCg................................  3      1  seq
.............................................................aauugguggcuccugcagC...............................  6      1  seq
.............................................................Nauugguggcuccugcaga...............................  9      1  seq
.............................................................aauugguggcuccugAaga...............................  3      1  seq
.............................................................aauugguggcuccugcaga...............................  1047   0  seq
.............................................................aauGgguggcuccugcaga...............................  3      1  seq
.............................................................aCuugguggcuccugcaga...............................  6      1  seq
.............................................................aauugguggcuccCgcaga...............................  3      1  seq
.............................................................aauuggCggcuccugcaga...............................  3      1  seq
.............................................................aauugguggcuccugcCgaa..............................  3      1  seq
.............................................................aauuggugAcuccugcagaa..............................  3      1  seq
.............................................................aauugguggcuccugcagaa..............................  1818   0  seq
.............................................................aauugguggAuccugcagaa..............................  3      1  seq
.............................................................aaAugguggcuccugcagaa..............................  3      1  seq
.............................................................aUuugguggcuccugcagaa..............................  3      1  seq
.............................................................aauuCguggcuccugcagaa..............................  3      1  seq
.............................................................aauugguggcuccugGagaa..............................  3      1  seq
.............................................................aauugguggcucUugcagaa..............................  3      1  seq
.............................................................aauugguggcuccugcagaG..............................  3      1  seq
```

```
Star                                                        Mature
gcuccugcagaagccaauugguggcuccugcagaagaaaauugguggcuccugcagaagccaauugguggcuccugcagaagaucagucccuuaaguaaaugaugguccac
.............................................................aauugguggcuccuUcagaa..............................	3	1	seq
.............................................................Nauugguggcuccugcagaa..............................	9	1	seq
.............................................................aauugAuggcuccugcagaag.............................	30	1	seq
.............................................................aauugguggcuccugcagaGg.............................	15	1	seq
.............................................................aaCugguggcuccugcagaag.............................	3	1	seq
.............................................................aauuggAggcuccugcagaag.............................	9	1	seq
.............................................................aauuggCggcuccugcagaag.............................	48	1	seq
.............................................................aauugguggcCccugcagaag.............................	9	1	seq
.............................................................aauugguAgcuccugcagaag.............................	105	1	seq
.............................................................aauuAguggcuccugcagaag.............................	78	1	seq
.............................................................aCuugguggcuccugcagaag.............................	21	1	seq
.............................................................aauugguggcuccuUcagaag.............................	33	1	seq
.............................................................aauugguggcuccugcagaCg.............................	12	1	seq
.............................................................aauugguggcuccugcagaaC.............................	27	1	seq
.............................................................aauugguggcuccAgcagaag.............................	9	1	seq
.............................................................aauAgguggcuccugcagaag.............................	15	1	seq
.............................................................aauugguggcuccugcagaag.............................	71832	0	seq
.............................................................aauuUguggcuccugcagaag.............................	123	1	seq
.............................................................aauuggugCcuccugcagaag.............................	6	1	seq
.............................................................aauCgguggcuccugcagaag.............................	6	1	seq
.............................................................Nauugguggcuccugcagaag.............................	264	1	seq
.............................................................aauugguggcuccugcagUag.............................	3	1	seq
.............................................................aauugguggUuccugcagaag.............................	24	1	seq
.............................................................aauugguggcuccugcCgaag.............................	54	1	seq
.............................................................aUuugguggcuccugcagaag.............................	12	1	seq
.............................................................aauugguggcuccCgcagaag.............................	63	1	seq
.............................................................aauuggGggcuccugcagaag.............................	3	1	seq
.............................................................aaAugguggcuccugcagaag.............................	18	1	seq
.............................................................aauugguggcucUugcagaag.............................	24	1	seq
.............................................................aauugguggcuccugcaCaag.............................	21	1	seq
.............................................................aauugguggcuUcugcagaag.............................	33	1	seq
.............................................................aaGugguggcuccugcagaag.............................	3	1	seq
.............................................................aauugguggcuccuAcagaag.............................	57	1	seq
.............................................................Uauugguggcuccugcagaag.............................	12	1	seq
.............................................................aauugguUgcuccugcagaag.............................	3	1	seq
.............................................................aGuugguggcuccugcagaag.............................	21	1	seq
.............................................................aauugguggAuccugcagaag.............................	12	1	seq
.............................................................aauugguggcuAcugcagaag.............................	3	1	seq
.............................................................aauugguggGuccugcagaag.............................	9	1	seq
.............................................................aauugguggcuGcugcagaag.............................	3	1	seq
.............................................................aauugguggcuccugcaAaag.............................	15	1	seq
.............................................................Gauugguggcuccugcagaag.............................	24	1	seq
.............................................................aauGgguggcuccugcagaag.............................	9	1	seq
.............................................................aauugUuggcuccugcagaag.............................	15	1	seq
.............................................................aauugguggcuccugAagaag.............................	3	1	seq
.............................................................aauugguggcAccugcagaag.............................	3	1	seq
.............................................................aauugguggcuccugcGgaag.............................	30	1	seq
.............................................................aauugguCgcuccugcagaag.............................	54	1	seq
.............................................................aauugguggcucAugcagaag.............................	3	1	seq
.............................................................aauuggugAcuccugcagaag.............................	72	1	seq
.............................................................aauugguggcuccugUagaag.............................	21	1	seq
.............................................................aauuggugUcuccugcagaag.............................	9	1	seq
.............................................................aauugguggcuccugcagaUg.............................	12	1	seq
.............................................................aauugguggcuccGgcagaag.............................	3	1	seq
.............................................................aauugguggcuccugcagCag.............................	84	1	seq
.............................................................Cauugguggcuccugcagaag.............................	3	1	seq
.............................................................aauugguggcuccugcUgaag.............................	3	1	seq
.............................................................aauugguggcuccugcaUaag.............................	9	1	seq
.............................................................aauugguggcuccugcagGag.............................	33	1	seq
.............................................................aauugguggcuccuCcagaag.............................	27	1	seq
.............................................................aauuCguggcuccugcagaag.............................	81	1	seq
.............................................................aauugguggcuccugcagaaga............................	58	0	seq
.............................................................aauugguggcuccugcagaagU............................	798	1	seq
.............................................................aUuugguggcuccugcagaaga............................	2	1	seq
.............................................................aauugguggcuccugcagaagG............................	12	1	seq
.............................................................aauugguggcuccugcagaagaC...........................	2	1	seq
.............................................................Nauugguggcuccugcagaagau...........................	1	1	seq
.............................................................aauugguggcuccugcagaagGu...........................	3	1	seq
.............................................................aauugguggcuccugcagaagUu...........................	15	1	seq
.............................................................aauugguggcuccugcagaagau...........................	6	0	seq
```

Star **Mature**

```
gcuccugcagaagccaauugguggcuccugcagaagaaaauugguggcuccugcagaagccaauugguggcuccugcagaagaucagucccuuaaguaaaugaugguccac
.............................................................aauugguggcuccugcagaagaCc..........................	2	1	seq
.............................................................aauugguggcuccugcagaagUuc..........................	1	1	seq
.............................................................aauugguggcuccugcagaagauU..........................	1	1	seq
.............................................................aauugguggcuccugcagaagaCca.........................	2	1	seq
..............................................................auugguggcuccugcaga...............................	15	0	seq
..............................................................auugguggcuccuUcagaa..............................	3	1	seq
..............................................................auugguggcCccugcagaa..............................	3	1	seq
..............................................................auugguggcuccugcagaa..............................	105	0	seq
..............................................................auugCuggcuccugcagaa..............................	3	1	seq
..............................................................auugAuggcuccugcagaag.............................	3	1	seq
..............................................................auugguggcuccugcagaag.............................	276	0	seq
..............................................................auugguggcuccugcagaagU............................	15	1	seq
..............................................................auugguAgcuccugcagaaga............................	2	1	seq
..............................................................Nuugguggcuccugcagaaga............................	4	1	seq
..............................................................auugAuggcuccugcagaaga............................	2	1	seq
..............................................................auugguggcuccCgcagaaga............................	4	1	seq
..............................................................auugguggcuccugcagaaga............................	928	0	seq
..............................................................auugguggcCccugcagaaga............................	2	1	seq
..............................................................auugguggcuccugUagaaga............................	2	1	seq
..............................................................aCugguggcuccugcagaaga............................	2	1	seq
..............................................................aAugguggcuccugcagaaga............................	2	1	seq
..............................................................auugguggcuccugGagaaga............................	2	1	seq
..............................................................auugguggcuccugcagaagCu...........................	11	1	seq
..............................................................auugguggcuccugcagaagau...........................	2	0	seq
..............................................................auugguggcuccugcagaagaCca.........................	1	1	seq
...............................................................uugguggcuccugcagaa..............................	15	0	seq
...............................................................uugguggcuccugUagaag.............................	3	1	seq
...............................................................Augguggcuccugcagaag.............................	3	1	seq
...............................................................uugguggcuccugcagaag.............................	54	0	seq
...............................................................uugguggcuccugcagaaga............................	6	0	seq
...............................................................uugguggcuccuAcagaagau...........................	1	1	seq
...............................................................uugguggcuccugcagCagau...........................	1	1	seq
...............................................................uugguggcuccugcaAaagau...........................	2	1	seq
...............................................................Nugguggcuccugcagaagau...........................	1	1	seq
...............................................................uugguggcuccugcagaagau...........................	154	0	seq
...............................................................uugguggcuccugcagaagCu...........................	2	1	seq
...............................................................uugguggcuccugcagaagaC...........................	4	1	seq
...............................................................uugguggcuccugcagaagauU..........................	3	1	seq
................................................................ugguggcuccugcagaag.............................	183	0	seq
................................................................ugguggcuccugcagaCg.............................	3	1	seq
................................................................ugguCgcuccugcagaag.............................	3	1	seq
................................................................uggugAcuccugcagaag.............................	3	1	seq
................................................................ugguggcuccugcagaaga............................	2	0	seq
................................................................ugguggcuccugcagaagU............................	6	1	seq
................................................................ugguAgcuccugcagaagau...........................	1	1	seq
................................................................ugguggcuccugcagaagauc..........................	108	0	seq
................................................................Ngguggcuccugcagaagauc..........................	2	1	seq
................................................................Cgguggcuccugcagaagauca.........................	1	1	seq
................................................................ugguggcuccugcagaagaucaA........................	1	1	seq
................................................................ugguggcuccugcagaagaucaAu.......................	1	1	seq
.................................................................gguggcuccugcagaagCu...........................	1	1	seq
.................................................................gguggcuccugcagaagaCc..........................	1	1	seq
.................................................................gguggcuccugcagaagauc..........................	4	0	seq
.................................................................gguggcuccuCcagaagauca.........................	1	1	seq
.................................................................gguggcuccugcagaagaucC.........................	1	1	seq
.................................................................gguggcuccugcagaagauca.........................	48	0	seq
.................................................................gguggcuccugcagaagaucU.........................	2	1	seq
..................................................................guggcuccugcagaagauc..........................	8	0	seq
..................................................................gugAcuccugcagaagauc..........................	1	1	seq
..................................................................guggcuccugcagaagauca.........................	5	0	seq
..................................................................guggcuccugcagaaUaucag........................	2	1	seq
..................................................................gGggcuccugcagaagaucag........................	1	1	seq
..................................................................guggcuccugcCgaagaucag........................	1	1	seq
..................................................................guggUuccugcagaagaucag........................	2	1	seq
..................................................................guggcuccugcaAaagaucag........................	1	1	seq
..................................................................guggcuccugcGgaagaucag........................	1	1	seq
..................................................................guggcuccugcagaagaucaC........................	2	1	seq
..................................................................guggcuccugcagaagaucag........................	257	0	seq
..................................................................guggcuccuAcagaagaucag........................	1	1	seq
..................................................................guggcuccugcagCagaucag........................	1	1	seq
```

Star **Mature**

```
gcuccugcagaagccaauugguggcuccugcagaagaaaauugguggcuccugcagaagccaauugguggcuccugcagaagaucagucccuuaaguaaaugaugguccac
..................................................................guggcuccugcagaagaucagu.......................	10	0	seq
..................................................................guggcuccugcagaagaucaguU......................	3	1	seq
...................................................................uggcuccugcagaagaucagu.......................	15	0	seq
....................................................................ggcuccugcagaagaucaguc......................	4	0	seq
.....................................................................gcuccugcagaagaucagucc.....................	6	0	seq
......................................................................cuccugcagaagaucaguccc....................	9	0	seq
......................................................................cuccugcagaagaucagucccu...................	1	0	seq
......................................................................cuccugcagaagaucagucccuuU.................	1	1	seq
.......................................................................uccugcagaagaucagucccu...................	3	0	seq
.......................................................................uccugcagaagaucagucccuu..................	1	0	seq
.........................................................................cugcagaagaucagucccuua.................	1	0	seq
.........................................................................cugcagaagaucagucccuAa.................	2	1	seq
.........................................................................cugcagaagaucagucccuuaU................	1	1	seq
..........................................................................ugcagaagaucagucccuuaa................	4	0	seq
..........................................................................ugcagaagaucagucccuAaa................	6	1	seq
...........................................................................gcagaagaucagucccuuaag...............	7	0	seq
...........................................................................gcagaagaucagucccuAaag...............	11	1	seq
...........................................................................gcaAaagaucagucccuuaag...............	1	1	seq
............................................................................cagaagaucagucccuAaa................	1	1	seq
............................................................................cagaagaucagucccuuaag...............	1	0	seq
............................................................................cagaagaucagucccuuaagu..............	8	0	seq
............................................................................cagaagaucagucccuAaagu..............	3	1	seq
.............................................................................agaagaucagucccuuaag...............	1	0	seq
.............................................................................agaagaucagucccuuaagua.............	2	0	seq
..............................................................................gaagaucagucccuAaag...............	2	1	seq
..............................................................................gUagaucagucccuuaaguaa............	1	1	seq
................................................................................agaucagucccuAaaguaa............	1	1	seq
................................................................................agaucagucccuAaaguaaa...........	2	1	seq
................................................................................agaucagucccuuaaguaaau..........	2	0	seq
................................................................................agaucagucccuAaaguaaau..........	1	1	seq
................................................................................agaucagucccuAaaguaaaugaugguc...	1	1	seq
.................................................................................gaucagucccuAaaguaaau..........	1	1	seq
.................................................................................gaucagucccuAaaguaaaug.........	10	1	seq
.................................................................................gaucagucccuuaaguaaaug.........	9	0	seq
.................................................................................gaucaguccUuuaaguaaaug.........	1	1	seq
..................................................................................aucagucccuuaaguaaauga........	2	0	seq
...................................................................................ucagucccuAaaguaaaug.........	1	1	seq
...................................................................................ucagucccuuaaguaaaugau.......	11	0	seq
...................................................................................ucagucccuAaaguaaaugau.......	9	1	seq
...................................................................................ucagucccuuaaguaaaugaugg.....	5	0	seq
...................................................................................ucagucccuAaaguaaaugaugg.....	2	1	seq
...................................................................................ucagucccuAaaguaaaugauggu....	2	1	seq
...................................................................................ucagucccuAaaguaaaugaugguc...	1	1	seq
....................................................................................cagucccuAaaguaaaug.........	1	1	seq
....................................................................................cagucccuAaaguaaauga........	4	1	seq
....................................................................................cagucccuuaaguaaaugau.......	1	0	seq
....................................................................................cagucccuAaaguaaaugau.......	4	1	seq
....................................................................................cagucccuuaaguaaaugaug......	77	0	seq
....................................................................................cagucccuAaaguaaaugaug......	117	1	seq
....................................................................................cagucccuuaaguaaaugaugg.....	1	0	seq
....................................................................................cagucccuuaaguaaaugaugUu....	2	1	seq
.....................................................................................agucccuAaaguaaaugau.......	1	1	seq
.....................................................................................agucccuuaaguaaaugau.......	1	0	seq
.....................................................................................agucccuuaaguaaaugaug......	2	0	seq
.....................................................................................agucccuAaaguaaaugaug......	2	1	seq
.....................................................................................agucccuAaaguaaaugaugg.....	22	1	seq
.....................................................................................agucccuuaaguaaaugaugg.....	20	0	seq
......................................................................................gucccuAaaguaaaugauggu....	14	1	seq
......................................................................................gucccuuaaguaaaugauggu....	17	0	seq
.......................................................................................ucccuAaaguaaaugaugguc...	1	1	seq
.......................................................................................ucccuuaaguaaaugaugguc...	1	0	seq
........................................................................................cccuuaaguaaaugaugg.....	1	0	seq
........................................................................................cccuAaaguaaaugaugg.....	1	1	seq
........................................................................................cccuAaaguaaaugaugguc...	1	1	seq
........................................................................................cccuuaaguaaaugauggucc..	1	0	seq
```

Provisional ID	:	ML0273_7758
Score total	:	5.1
Score for star read(s)	:	-1.3
Score for read counts	:	0
Score for mfe	:	1.8
Score for randfold	:	1.6
Score for cons. seed	:	3
Total read count	:	54
Mature read count	:	54
Loop read count	:	0
Star read count	:	0

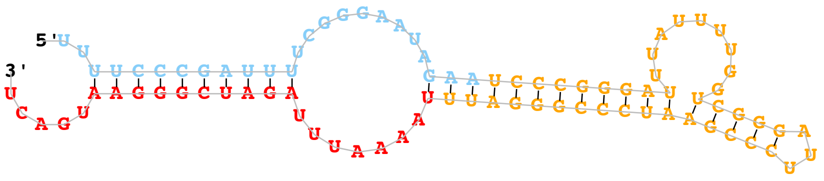

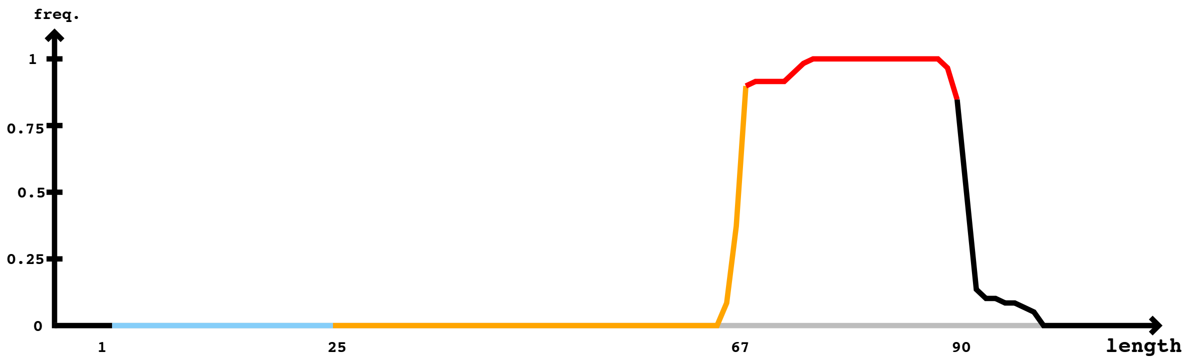

Star

Mature

```
5'- aaaguuuucccgauuuucgggaauagaaucccgggauuuauuuuggucgggauucccgaaucccgggauuuaaaauuuagaucgggaaugacuucacuaacggacacacuaau   -3'    exp
    .(((((((((((((((.........((((((((((((.........(((((...))))))))))))))))).......)))))))))).)))))...................   reads      mm        sample
    ....................................................................uuuaaaauuuagaucgggaaugG......................   1          1         seq
    ....................................................................uuuaaaauuuagaucgggaaugac.....................   3          0         seq
    ....................................................................uuuaaaauuuagaucgggaaugacu....................   1          0         seq
    .....................................................................uuaaaauuuagaucgggaaugac.....................   4          0         seq
    .....................................................................uuaaaauuuagaucgggaaugacu....................   13         0         seq
    ......................................................................uaaaauuuagaucgggaauga......................   1          0         seq
    ......................................................................uaaaauuuagaucgggaaugacu....................   27         0         seq
    ......................................................................uaaaauuuagaucgggaaugacuu...................   1          0         seq
    ......................................................................uaaaauuuagaucgggaaugacuuc..................   2          0         seq
    .......................................................................Uaaauuuagaucgggaaugacuucac................   1          1         seq
    ...........................................................................uuuagaucgggaaugacuucacuaCc............   2          1         seq
    ............................................................................uuagaucgggaaugacuucacua..............   1          0         seq
    ............................................................................uuagaucgggaaugacuucacuaC.............   1          1         seq
    .............................................................................uagaucgggaaugacuucacuaGc............   1          1         seq
```

Provisional ID	:	ML0407_10813
Score total	:	5
Score for star read(s)	:	-1.3
Score for read counts	:	0
Score for mfe	:	1.7
Score for randfold	:	1.6
Score for cons. seed	:	3
Total read count	:	161
Mature read count	:	160
Loop read count	:	1
Star read count	:	0

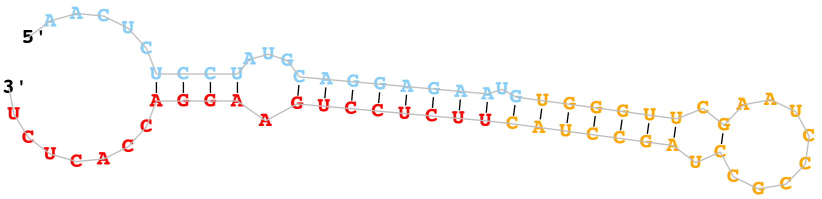

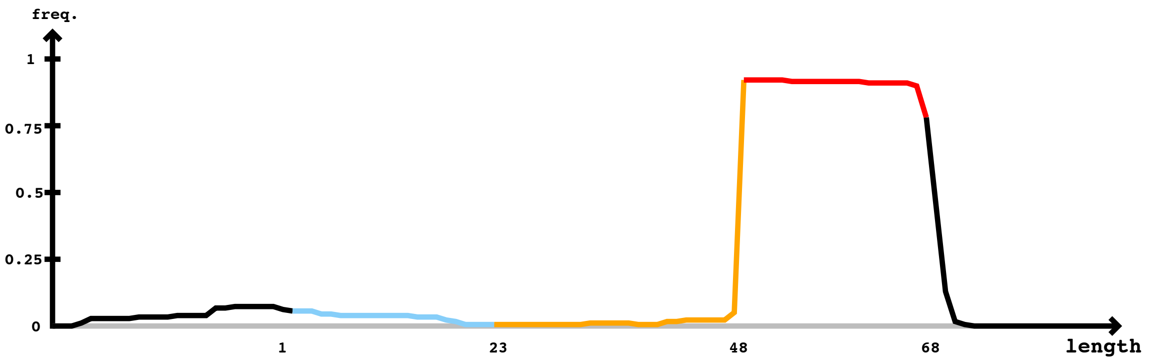

Star　　　　　　　　　　　　　Mature

```
5'- ccucaggcuagugagucaacgguaacucuccuaugcaggagaauguggguucgaaucccgccuagccuacuucuccugaaggaccacucuuuugaauugaguggccuca   -3'    exp
    ....((((((.(.((((((.((......((((...((((((((.(((((((.(........).))))))))))))))).)))).....)).))).))).).))))))..   reads   mm   sample
    .cucaggcuagugagucaacgg.......................................................................................   2   0   seq
    ..ucaggcuagugagucaacggu......................................................................................   1   0   seq
    ..ucaggcuagugagucaacgguaac...................................................................................   2   0   seq
    .......cuagugagucaacgguaacuc.................................................................................   1   0   seq
    ...........ugagucaacgguaacucuccuaugc.........................................................................   1   0   seq
    ...............ucaacgguaacucuccuaugcagA......................................................................   1   1   seq
    ...............ucaacgguaacucuccuaugcagC......................................................................   1   1   seq
    ...............ucaacgguaacucuccuaugcagga.....................................................................   1   0   seq
    ...............ucaacgguaacucuccuaugcagAag....................................................................   2   1   seq
    .................aacgguaacucuccuaugcag.......................................................................   1   0   seq
    ......................................gagaauguggguucgaauccc..................................................   1   0   seq
    ......................................................aucccgccuagccuacuucuc..................................   1   0   seq
    ..............................................................Nagccuacuucuccugaagga..........................   1   1   seq
    ..............................................................uagccuacuucuccugaaggaccacu.....................   1   0   seq
    ................................................................gccuacuucuccugaaggaccacucu...................   1   0   seq
    .....................................................................cuucuccugaaggaccacucu...................   4   0   seq
    .....................................................................cuucuccugaaggaccacucuA..................   1   1   seq
    ......................................................................uucuccugaaggaccacu.....................   1   0   seq
    ......................................................................uucuccugaaggaUcacuc....................   1   1   seq
    ......................................................................uucuccugaaggaccacuc....................   20   0   seq
    ......................................................................uucuccugaaggaccacucA...................   3   1   seq
    ......................................................................uucuccugaaggaccacucu...................   47   0   seq
    ......................................................................Aucuccugaaggaccacucu...................   1   1   seq
    ......................................................................Gucuccugaaggaccacucu...................   1   1   seq
    ......................................................................uucuccugaaggaccacucC...................   4   1   seq
    ......................................................................uucuccugaaggaccacucCu..................   1   1   seq
    ......................................................................uucuccugaaggaccacucuG..................   1   1   seq
    ......................................................................uucuccugaaggaccacucuu..................   45   0   seq
    ......................................................................uucuccugaaggaccacucAu..................   1   1   seq
    ......................................................................uucuccugaaggaccacucuA..................   5   1   seq
    ......................................................................uucuccugaaggCccacucu...................   1   1   seq
    ......................................................................uucuccugaaggaccacucuuu.................   19   0   seq
    ......................................................................Nucuccugaaggaccacucuuu.................   1   1   seq
    ......................................................................uucuccugaaggaccacucuuuu................   2   0   seq
```

```
                       Star                                          Mature
ccucaggcuagugagucaacgguaacucuccuaugcaggagaaugugguucgaaucccgccuagccuacuucuccugaaggaccacucuuuugaauugaguggccuca
......................................................................uucuccugaaggaccacucuuuuU...............    1    1    seq
```

Provisional ID	:	ML0673_15349
Score total	:	4.7
Score for star read(s)	:	-1.3
Score for read counts	:	0
Score for mfe	:	1.4
Score for randfold	:	1.6
Score for cons. seed	:	3
Total read count	:	54
Mature read count	:	54
Loop read count	:	0
Star read count	:	0

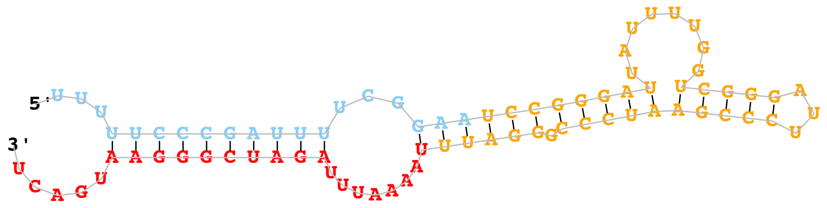

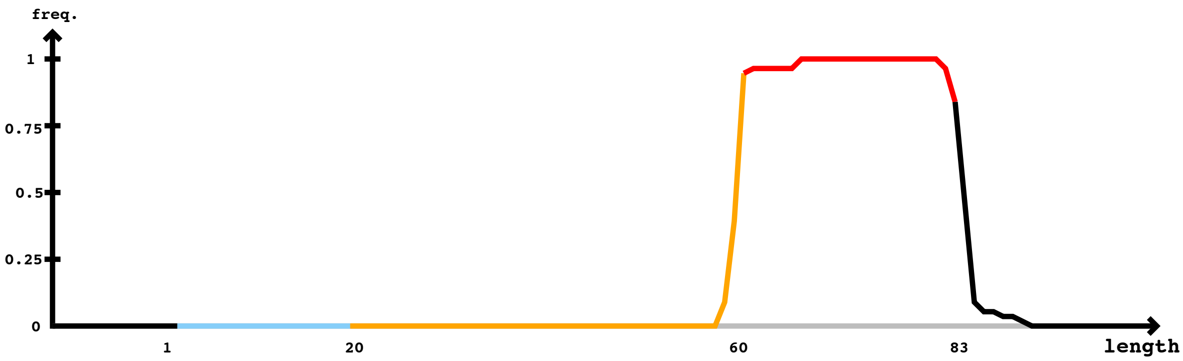

Star

Mature

```
5'- uuuucgggaaauuuuucccgauuuucggaauccgggauuauuuuggucgggauucccgaaucccgggauuuaaaauuuagaucgggaaugacuucacuaagguaaacuccaua   -3'    exp
    .....(((((.((.((((((((((...(((((((((((........(((((...))))))))).).......)))))))))).)).))).))..((......))...    reads    mm    sample
    ....................................................................uuuaaaauuuagaucgggaaugG......................    1    1    seq
    ....................................................................uuuaaaauuuagaucgggaaugac.....................    3    0    seq
    ....................................................................uuuaaaauuuagaucgggaaugacu....................    1    0    seq
    .....................................................................uuaaaauuuagaucgggaaugac.....................    4    0    seq
    .....................................................................uuaaaauuuagaucgggaaugacu....................    13    0    seq
    ......................................................................uaaaauuuagaucgggaauga......................    1    0    seq
    ......................................................................uaaaauuuagaucgggaaugacu....................    27    0    seq
    ......................................................................uaaaauuuagaucgggaaugacuu...................    1    0    seq
    ......................................................................uaaaauuuagaucgggaaugacuuc..................    2    0    seq
    .......................................................................Uaaauuuagaucgggaaugacuucac................    1    1    seq
    ............................................................................uuagaucgggaaugacuucacua..............    1    0    seq
    ............................................................................uuagaucgggaaugacuucacuaC.............    1    1    seq
```

Provisional ID	:	ML0969_21055
Score total	:	4.6
Score for star read(s)	:	-1.3
Score for read counts	:	0
Score for mfe	:	1.3
Score for randfold	:	1.6
Score for cons. seed	:	3
Total read count	:	117
Mature read count	:	117
Loop read count	:	0
Star read count	:	0

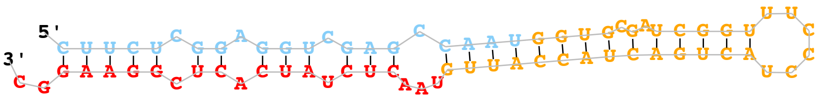

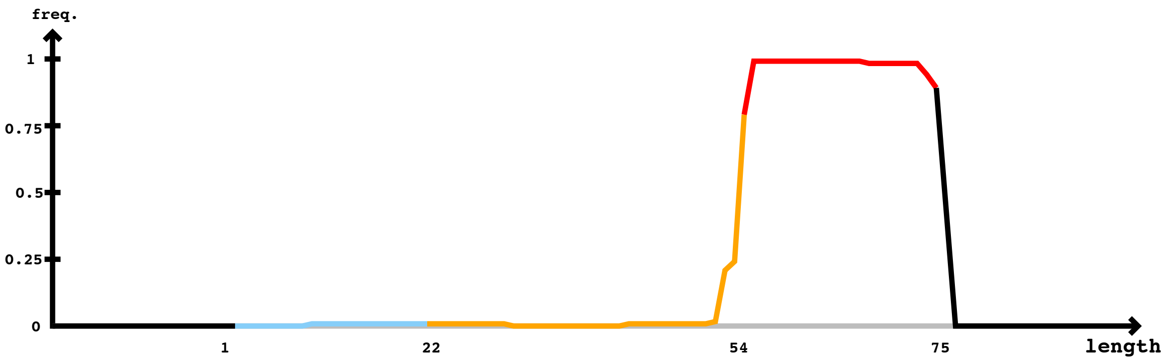

Star Mature

```
5'- gguccucacggaaaccccuucucggaggucgagccaauggugcgaucgguuucccuacugacuaccauuguaacucuaucacucggaaggccccacuuuuccugcaaggaa  -3'   exp
    ..((((..((((((..((((((.((.(((.(((.((((((((.(.(((((......))))))))))))))...))).))).)).)))))).......))))).)..)))).   reads   mm   sample
    .........................aggucgagccaauggugcgCu.................................................................   1       1    seq
    ..........................................................ugacuaccauuguaacucuaucacu............................   1       0    seq
    ...................................................................uuguaacucuaucacucggaaggc....................   1       0    seq
    ....................................................................uguaacuUuaucacucggaag......................   2       1    seq
    ....................................................................uguaacucuaucacucggaag......................   3       0    seq
    ....................................................................uguaacucuaucacucggaaggcc...................   17      0    seq
    ....................................................................uguaaUucuaucacucggaaggcc...................   1       1    seq
    .....................................................................guaacucuaucacucggaagg.....................   4       0    seq
    ......................................................................uaacuUuaucacucggaagg.....................   1       1    seq
    ......................................................................uaacucuaucacucggaaggc....................   52      0    seq
    ......................................................................uaacuUuaucacucggaaggc....................   13      1    seq
    .......................................................................aacucuaucacucggaagg.....................   1       0    seq
    .......................................................................aacucuaucacucggaaggcc...................   19      0    seq
    .......................................................................aacAcuaucacucggaaggcc...................   1       1    seq
    .......................................................................aacuUuaucacucggaaggcc...................   2       1    seq
    .......................................................................aacucuaucacucgUaaggcc...................   1       1    seq
```

Provisional ID	:	ML0537_13398
Score total	:	4.3
Score for star read(s)	:	-1.3
Score for read counts	:	0
Score for mfe	:	1
Score for randfold	:	1.6
Score for cons. seed	:	3
Total read count	:	327
Mature read count	:	327
Loop read count	:	0
Star read count	:	0

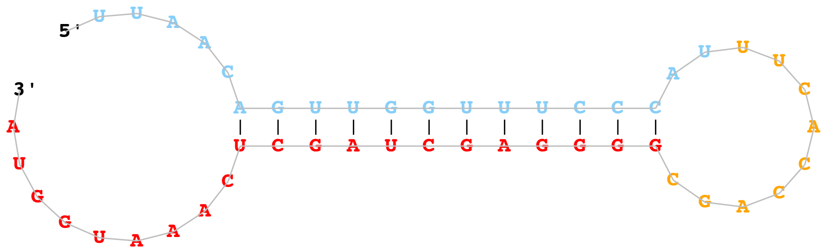

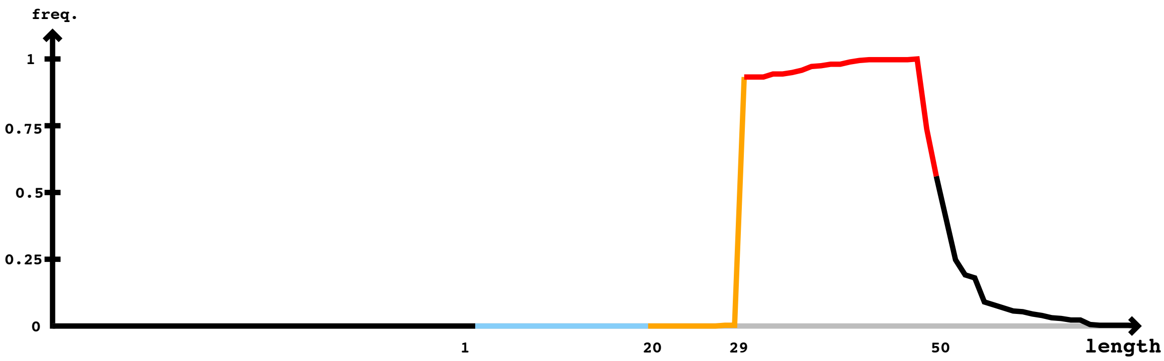

Star Mature

```
5'- aauuaagaagguuugacuuuagcaucaaggcauucaguuuuauuaacaguugguuucccauuucaccagcggggagcuagcucaaaugguagagcgcucgcuuagcaugcg   -3'    exp
    ......((((......)))).(((((.((((.....((((((((..(((((((((((((...........))))))))))))....)))))))))....)))).).)))).   reads   mm   sample
    ....................................................................gcggggagcuagcucaaaugguagag.................   1   0   seq
    ......................................................................ggAgagcuagcucaaaugg......................   1   1   seq
    ......................................................................ggggagcuagcucaaaugg......................   90   0   seq
    ......................................................................ggggUgcuagcucaaaugg......................   2   1   seq
    ......................................................................ggggagcuagcucaaauggu.....................   62   0   seq
    ......................................................................gUggagcuagcucaaauggu.....................   1   1   seq
    ......................................................................ggggagcuagcucaaaugguU....................   4   1   seq
    ......................................................................ggggagcuagcucaaauggua....................   95   0   seq
    ......................................................................Ngggagcuagcucaaauggua....................   1   1   seq
    ......................................................................ggggagcuaAcucaaauggua....................   3   1   seq
    ......................................................................ggggagcuagcucaaaugguaU...................   4   1   seq
    ......................................................................ggggagcuagcucaaaugguag...................   3   0   seq
    ......................................................................ggggagcuagcucaaaugguGga..................   1   1   seq
    ......................................................................ggggagcuagcucaaaugguaga..................   16   0   seq
    ......................................................................ggggagcuagcucaaaugguagag.................   3   0   seq
    ......................................................................ggggaUcuagcucaaaugguagagc................   1   1   seq
    ......................................................................ggggagcuaCcucaaaugguagagc................   1   1   seq
    ......................................................................ggggagcuagcucaaaugguagagc................   26   0   seq
    ......................................................................ggggagcuagcucaaaugguagagcU...............   1   1   seq
    ......................................................................ggggagcuagcucaaaugguagagcC...............   2   1   seq
    ......................................................................ggggagcuagcucaaaugguagagcUc..............   2   1   seq
    ......................................................................ggggagcuagcucaaaugguagagcgc..............   1   0   seq
    ......................................................................ggggagcuagcucaaaugguagagcAc..............   1   1   seq
    ......................................................................ggggagcuagcucaaaugguagagAgcu.............   1   1   seq
    ......................................................................ggggagcuagcucaaaugguagagcAcu.............   1   1   seq
    ......................................................................ggggagcuagcucaaaugguagagcAcuc............   1   1   seq
    ......................................................................ggggagcuagcucaaaugguagagcUcucg...........   2   1   seq
    ......................................................................ggggagcuagcucaaaugguagagcgcucgc..........   1   0   seq
    ......................................................................ggggagcuagcucaaaugguagagcgcucgcu.........   1   0   seq
    ......................................................................ggggagcuagcucaaaugguagagcgcucgcuuGgc.....   2   1   seq
    .........................................................................gagcuagcucaaaugguag...................   1   0   seq
    .........................................................................gagcuagcucaaaugguaga..................   3   0   seq
    ...........................................................................gcuagcucaaaugguagagcU...............   1   1   seq
    ...........................................................................gcuagcucaaaugguagagcUcu.............   1   1   seq
```

Star Mature

aauuaagaagguuugacuuuagcaucaaggcauucaguuuuauuaaacaguugguuucccauuucaccagcggggagcuagcucaaaugguagagcgcucgcuuagcaugcg			
....cuagcucaaaugguagagc................	2	0	seq
....cuagcucaaaugguagagcUcu.............	1	1	seq
....uagcucaaaugguagagc................	2	0	seq
....uagcucaaaugguagagcgcucgcuu........	1	0	seq
....uagcucaaaugguagagcgcucgcuuU.......	2	1	seq
....agcucaaaugguagagcgcucgcuuGgc.....	1	1	seq
....gcucaaaugguagagcgcucgcu.........	1	0	seq
....gcucaaaugguagagcgcucgcuuagcaugcg	1	0	seq
....ucaaaugguagagcUcucg...........	1	1	seq
....ucaaaugguagagcCucgcu..........	1	1	seq
....ucaaaugguagagcgcucgcuuGgc.....	1	1	seq
....caaaugguagagcUcucgc..........	1	1	seq
....caaaugguagagcgcucgcuuGgc.....	1	1	seq
....aaaugguagagcgcucgcuuGgc.....	1	1	seq
....guagagcgcucgcuuagca....	1	0	seq

Provisional ID	:	ML0303_8318
Score total	:	4.2
Score for star read(s)	:	3.9
Score for read counts	:	-0.8
Score for mfe	:	0.4
Score for randfold	:	-2.2
Score for cons. seed	:	3
Total read count	:	10
Mature read count	:	9
Loop read count	:	0
Star read count	:	1

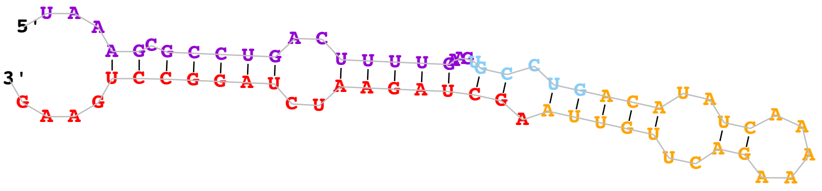

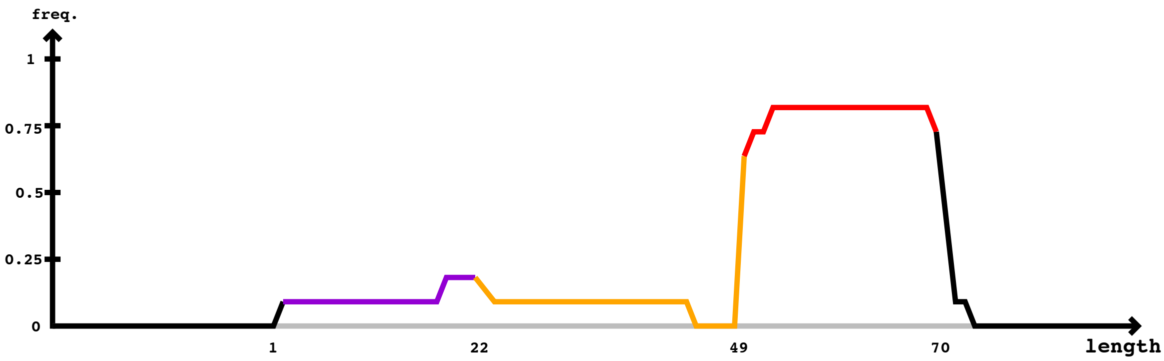


```
                     Star                                          Mature
5'- gucaccuguaaagcgccugacauaaagcgccugacuuuugaagugccugacauaucaaaaagacuuguuaagcuagaaucuaggccugaaguggccuguugcgggaaaacc   -3'    obs
    gucaccuguaaagcgccugacauaaagcgccugacuuuugaagugccugacauaucaaaaagacuuguuaagcuagaaucuaggccugaaguggccuguugcgggaaaacc          exp
    ....(((((((((.(((........((.(((((..(((((....((.(((((..((.....))..))))).)))))))..))))))).....))))).)))))))......   reads   mm   sample
    ......................uaaagcgccugacuuuugaag....................................................................   1       0    seq
    .......................................gaagugccugacauaucaaaaagacu..............................................   1       0    seq
    ......................................................................agcuagaaucuaggccugaa.....................   1       0    seq
    ......................................................................agcuagaaucuaggccugaag....................   6       0    seq
    .......................................................................gcuagaaucuaggccugaagu...................   1       0    seq
    .........................................................................uagaaucuaggccugaagugg.................   1       0    seq
```

Provisional ID	:	ML2016_32098
Score total	:	3.6
Score for star read(s)	:	-1.3
Score for read counts	:	0
Score for mfe	:	0.3
Score for randfold	:	1.6
Score for cons. seed	:	3
Total read count	:	12
Mature read count	:	12
Loop read count	:	0
Star read count	:	0

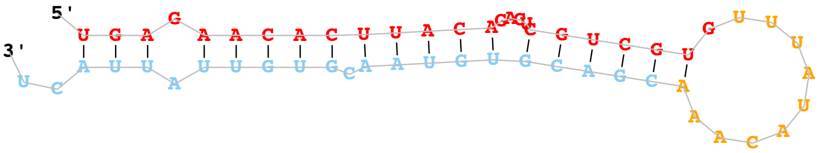

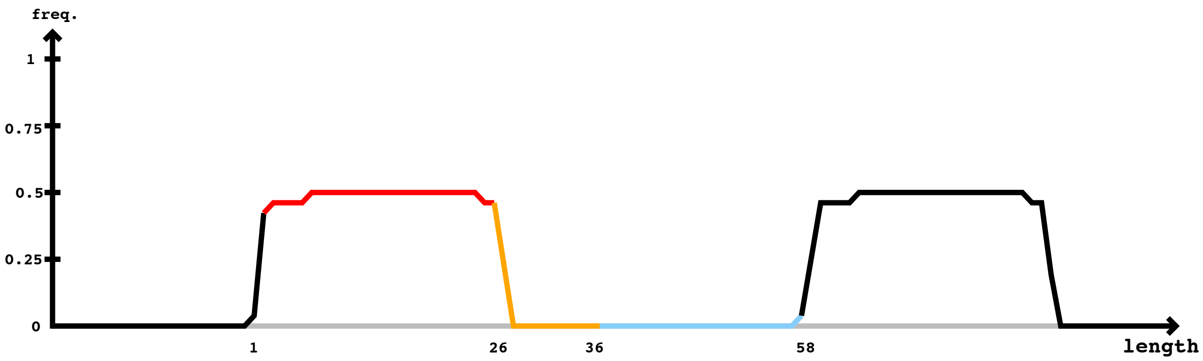

Mature **Star**

```
5'- acguguaacguguuauuacuugagaacacuuacagacucgucguguuuauacaaacgacguguaacguguuauuacuugagaacacuuacagacucgucguguuuauacaaacga   -3'    exp
    ...(((((.(((((.(((..(((.((((((((((....((((((..........))))))))))).))))).)))..))).))))))))))...((((..(((....))).))))    reads      mm         sample
    ...................uugaUaacacuuacagacucgucgug......................................................................    2          1          seq
    ....................ugagaacacuuacagacucgucgug......................................................................    12         0          seq
    ....................ugagaacacuuacagacucgucgugu.....................................................................    6          0          seq
    ....................ugagaacacuuacaAacucgucgugu.....................................................................    2          1          seq
    .....................gagaacacuuacagacucgucg........................................................................    2          0          seq
    .........................acacuuacagacucgucgugu.....................................................................    2          0          seq
    ............................................................................uugaUaacacuuacagacucgucgug.............    2          1          seq
    .............................................................................ugagaacacuuacagacucgucgug.............    12         0          seq
    .............................................................................ugagaacacuuacaAacucgucgugu............    2          1          seq
    .............................................................................ugagaacacuuacagacucgucgugu............    6          0          seq
    ..............................................................................gagaacacuuacagacucgucg...............    2          0          seq
    ..................................................................................acacuuacagacucgucgugu............    2          0          seq
```

Provisional ID	:	ML0702_16005
Score total	:	2.7
Score for star read(s)	:	-1.3
Score for read counts	:	-2.8
Score for mfe	:	2.3
Score for randfold	:	1.6
Score for cons. seed	:	3
Total read count	:	6
Mature read count	:	6
Loop read count	:	0
Star read count	:	0

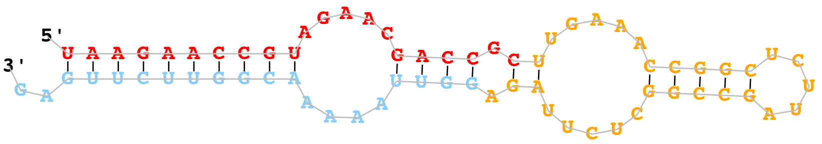

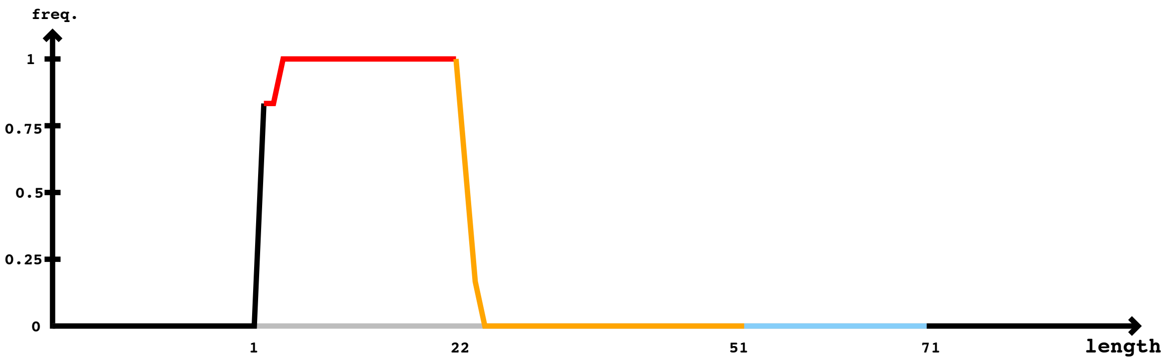

Mature Star

```
5'- agaaacgcuucagaacuguuuaagaaccguagaacgaccgcuugaaaccggcucuuagccggcucuuagagguuaaaaacgguucuugagcgguucuugagcgguucuugg   -3'    exp
    ((((.(((((.(((((((((((((((((((.....((((.((.....(((((.....))))).....)).))))....))))))))))))))))))).))))).))))...    reads    mm    sample
    ....................uaagaaccguagaacgaccgc......................................................................    5        0     seq
    ......................agaaccguagaacgaccgcuu....................................................................    1        0     seq
```

Provisional ID	:	ML0211_6802
Score total	:	2.2
Score for star read(s)	:	3.9
Score for read counts	:	3.7
Score for mfe	:	-2.5
Score for randfold	:	-2.2
Score for cons. seed	:	-0.6
Total read count	:	19
Mature read count	:	18
Loop read count	:	0
Star read count	:	1

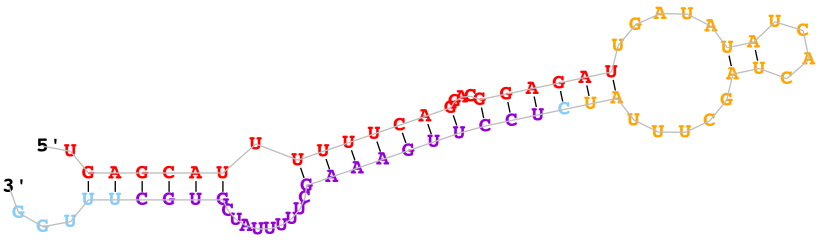

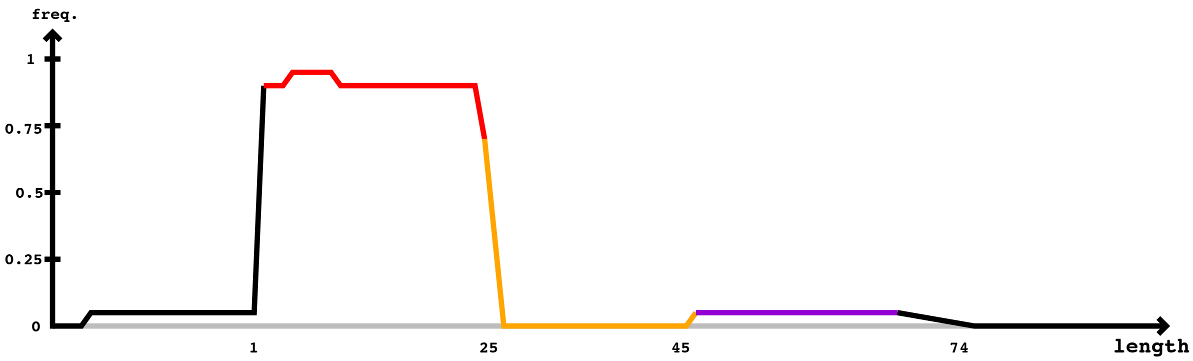

Mature **Star**

```
5'- agguggcaagaaauaauuggugagcauuuuuucaggacggagauugauauaucacuagcuuuaucuccuugaaagcuuuuuaucgugcuuuggauuucuucacugucgcagggu   -3'    obs
    agguggcaagaaauaauuggugagcauuuuuucaggacggagauugauauaucacuagcuuuaucuccuugaaagcuuuuuaucgugcuuuggauuucuucacugucgcagggu          exp
    ..((((((.(((..((((.(.((((((.(((((((...((((((.....((....)).....)))))))))))))).........))))))).)))).))).))))))....   reads   mm   sample
    ..guggcaagaaauaauuggugagcauu......................................................................................   1       0    seq
    ....................ugagcauuuuuucaggacggaga.......................................................................   3       0    seq
    ....................ugagcauuuuuucaggacggagau......................................................................   10      0    seq
    ....................ugagcauuuuuucaggacggagauu.....................................................................   3       0    seq
    ....................Ngagcauuuuuucaggacggagauu.....................................................................   1       1    seq
    .......................gcauuuuuucaggacggaga.......................................................................   1       0    seq
    .................................................................uccuugaaagcuuuuuaucgugc..........................   1       0    seq
```

Provisional ID	:	ML0821_18450
Score total	:	2.2
Score for star read(s)	:	3.9
Score for read counts	:	3.7
Score for mfe	:	-2.5
Score for randfold	:	-2.2
Score for cons. seed	:	-0.6
Total read count	:	19
Mature read count	:	18
Loop read count	:	0
Star read count	:	1

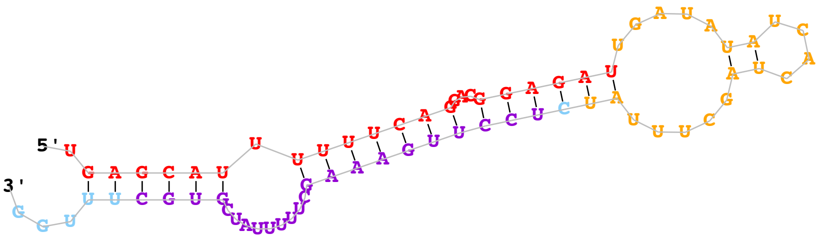

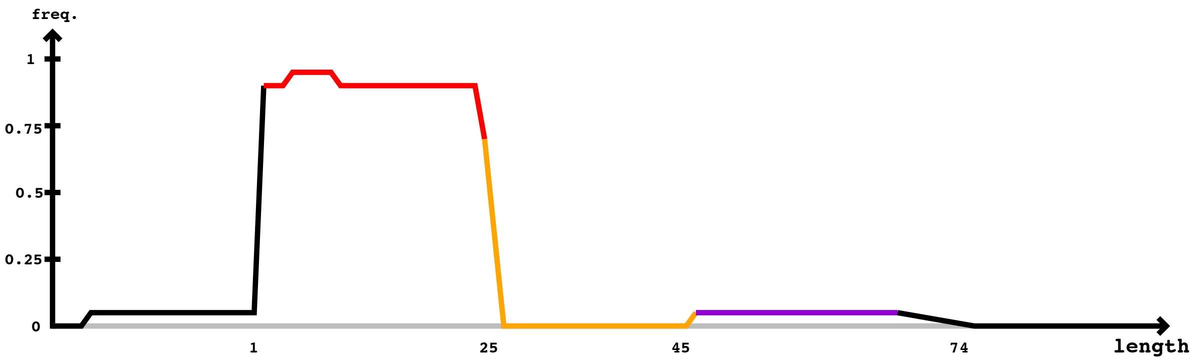

Mature　　　Star

```
5'- agguggcaagaaauaauuggugagcauuuuuucaggacggagauugauauaucacuagcuuuaucuccuugaaagcuuuuuaucgugcuuuggauuucuucacugucgcagggu  -3'   obs
    agguggcaagaaauaauuggugagcauuuuuucaggacggagauugauauaucacuagcuuuaucuccuugaaagcuuuuuaucgugcuuuggauuucuucacugucgcagggu        exp
    ..((((((.(((..((((.(.((((((.(((((((...((((((.....((....)).....)))))))))))))).........))))))).)))).)))..))))))..... reads  mm  sample
    ..guggcaagaaauaauuggugagcauu.......................................................................................... 1  0  seq
    ....................ugagcauuuuuucaggacggaga........................................................................... 3  0  seq
    ....................ugagcauuuuuucaggacggagau.......................................................................... 10 0  seq
    ....................Ngagcauuuuuucaggacggagauu..................................................................... 1  1  seq
    ....................ugagcauuuuuucaggacggagauu..................................................................... 3  0  seq
    ......................gcauuuuuucaggacggaga........................................................................ 1  0  seq
    .................................................................uccuugaaagcuuuuuaucgugc.......................... 1  0  seq
```

Provisional ID	:	ML0966_20966
Score total	:	2.2
Score for star read(s)	:	-1.3
Score for read counts	:	0
Score for mfe	:	-1
Score for randfold	:	1.6
Score for cons. seed	:	3
Total read count	:	97
Mature read count	:	97
Loop read count	:	0
Star read count	:	0

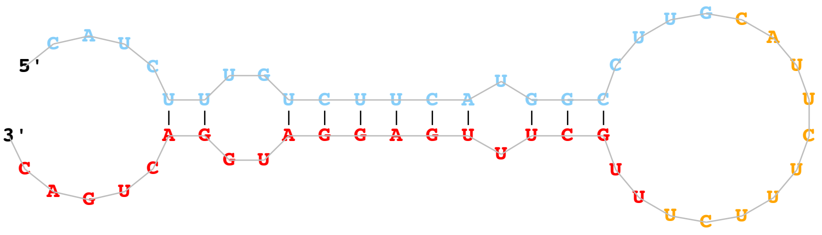

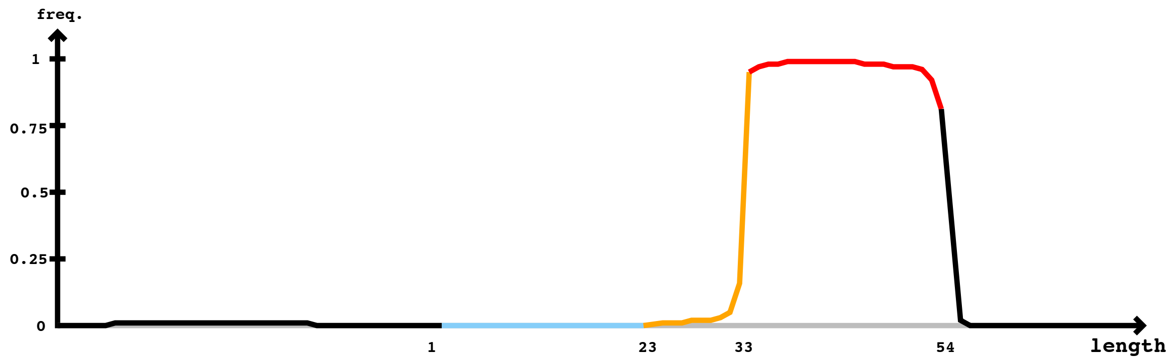

Star Mature

```
5'- cugcugguguucagcaagugaaauuucccaucuucuuucaucuuugucuucauggccuugcauucuuucuuugcuuugaggauggacugacuggauugaacucuucacacu -3'  exp
    .(((((.....))))).(((((..(((..((((....(((..((..((((((.(((................))).))))))..)).)))..)))).)))...)))))...  reads  mm  sample
    ....ugguguucagcaagugaaauu......................................................................................  1  0  seq
    .............................................................auucuuuUuugcuuugagga..............................  1  1  seq
    ................................................................cuuucuuugcuuugaggaugg..........................  1  0  seq
    ...................................................................ucuuugcuuugaggauggacu.......................  1  0  seq
    ....................................................................Uuuugcuuugaggauggacug......................  1  1  seq
    ....................................................................cuuugcuuugaggauggacug......................  1  0  seq
    .....................................................................uuugcuuugaggauggacuga.....................  11  0  seq
    ......................................................................uugcuuugaggauggacug......................  2  0  seq
    ......................................................................Nugcuuugaggauggacugac....................  1  1  seq
    ......................................................................uugcuuugaggauggacugac....................  77  0  seq
    .......................................................................ugcuuugaggauggacugacu...................  2  0  seq
    ........................................................................Acuuugaggauggacugacug..................  1  1  seq
    ..........................................................................uuugaggauggacugacug..................  1  0  seq
```
