# Supplementary material for: MicroRNAs and essential components of the microRNA processing machinery are not encoded in the genome of the ctenophore Mnemiopsis leidyi
Source: BMC Genomics. 2012 Dec 20;13:714. doi: 10.1186/1471-2164-13-714 (PMC3563456; doi:10.1186/1471-2164-13-714)
Supplement: Additional file 3 — Dataset 2. contains a folder of output data files in plain text format related to the miRNA predictions (both canonical and mirtron) produced by the various programs described in the Methods. [file 1471-2164-13-714-S3.zip › Additional_Dataset_2/b_sample2_md2_scores_gt10.pdf]

Provisional ID	:	ML3582_44088
Score total	:	10227.2
Score for star read(s)	:	3.9
Score for read counts	:	10222.1
Score for mfe	:	0.4
Score for randfold	:	-2.2
Score for cons. seed	:	3
Total read count	:	20062
Mature read count	:	19955
Loop read count	:	96
Star read count	:	11

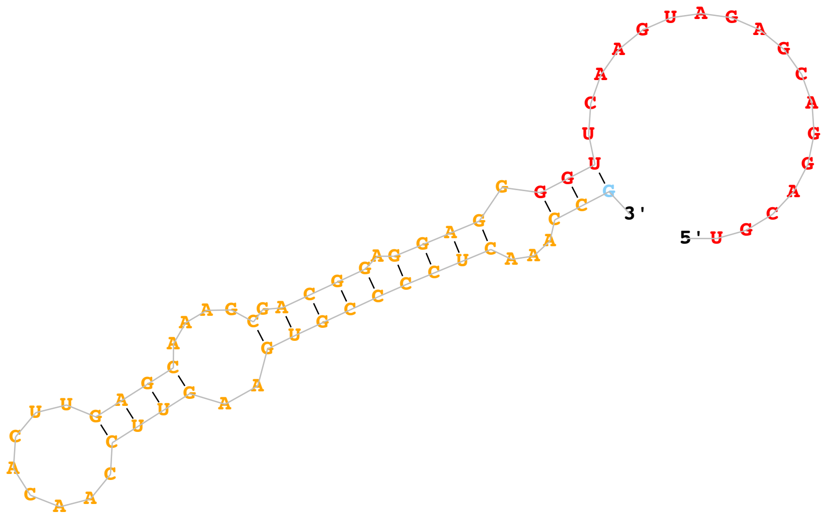

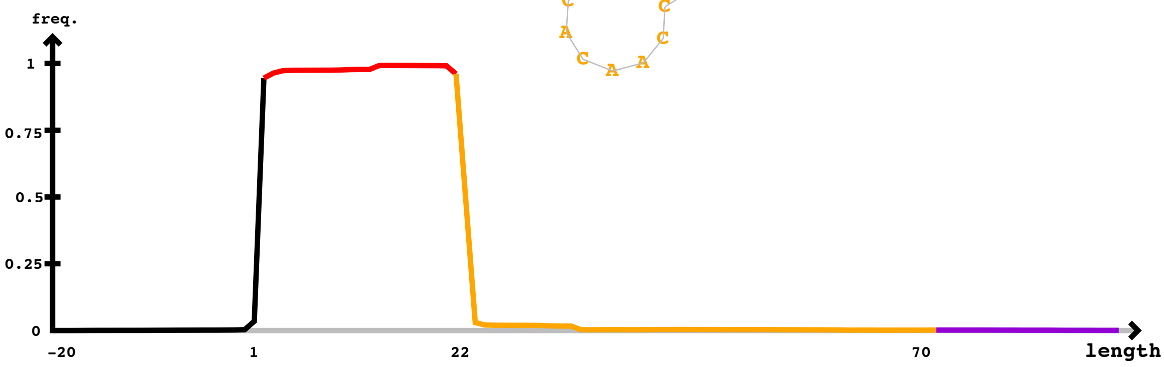

Mature **Star**

```
5'- uucucgaaaaucauucagcuugcaggacgagaugaacuuggggaggaggcagcgaaacgaguucacaaccuugaagugccccucaaaccgcaguuuggccugcucuuuaca  -3'   obs
    uucucgaaaaucauucagcuugcaggacgagaugaacuuggggaggaggcagcgaaacgaguucacaaccuugaagugccccucaaaccgcaguuuggccugcucuuuaca         exp
    .....................(((((......(((((((((.((((.((((.(....((((........))))..)))))))))...))).)))))).))))).......   reads   mm   sample
    uucucgaaaaucauucagcuu.........................................................................................   1       0    seq
    .ucucgaaaaucauucagcuug........................................................................................   3       0    seq
    ..cucgaaaaucauucagcuugc.......................................................................................   5       0    seq
    ...ucgaaaaucauucagcuugcaggac..................................................................................   1       0    seq
    .....gaaaaucauucagcuugcagg....................................................................................   3       0    seq
    ........aaucauucagcuugcaggacg.................................................................................   1       0    seq
    .........aucauucagcuugcaggacga................................................................................   5       0    seq
    ..........ucauucagcuugcaggacgag...............................................................................   2       0    seq
    ...........cauucagcuugcaggacgaga..............................................................................   4       0    seq
    ............auucagcuugcaggacgagau.............................................................................   1       0    seq
    .............uucagcuugcaggacgagaug............................................................................   2       0    seq
    .............uucagcuugcaggacgagaugU...........................................................................   1       1    seq
    ..............ucagcuugcaggacgagauga...........................................................................   1       0    seq
    ................agcuugcaggacgagaugaa..........................................................................   1       0    seq
    ................agcuugcaggacgagaugaac.........................................................................   5       0    seq
    .................gcuugcaggacgagaugaacu........................................................................   3       0    seq
    .................gcuugcaggacgagaugaacuugg.....................................................................   5       0    seq
    ..................cuugcaggacgagaugaacuu.......................................................................   15      0    seq
    ..................cuugcaggacgagGugaacuu.......................................................................   1       1    seq
    ..................cuugcaggacgagaugaacuuU......................................................................   3       1    seq
    ...................uugcaggacgagauAaacuug......................................................................   5       1    seq
    ...................uugcaggacgagauUaacuug......................................................................   1       1    seq
    ...................uugcaggacgagaugaacuug......................................................................   606     0    seq
    ...................uugcaggaUgagaugaacuug......................................................................   2       1    seq
    ...................uugcaggacgagauCaacuug......................................................................   4       1    seq
    ...................uugUaggacgagaugaacuug......................................................................   1       1    seq
    ...................Cugcaggacgagaugaacuugg.....................................................................   7       1    seq
    ...................uugcaggacgagaugaacuugA.....................................................................   6       1    seq
    ...................uugcaggacgagaugaacuugU.....................................................................   2       1    seq
    ...................uugcaggacgagaugaacuugg.....................................................................   12      0    seq
    ...................uugcaggacgagaugaacuuggC....................................................................   1       1    seq
    ...................uugcaggacgagaugaacuuggg....................................................................   1       0    seq
    ...................uugcaggacgagaugaacuugggg...................................................................   1       0    seq
```

```
                    Mature                                                Star
uucucgaaaaucauucagcuugcaggacgagaugaacuuggggaggaggcagcgaaacgaguucacaaccuugaagugccccucaaaccgcaguuuggccugcucuuuaca
....................ugcaggacgagaugaacuug.......................................................................  1      0   seq
....................ugcaggacgagaugaacuugA......................................................................  9      1   seq
....................ugcaAgacgagaugaacuugg......................................................................  1      1   seq
....................ugcaggacgaCaugaacuugg......................................................................  1      1   seq
....................ugcaggacgagaCgaacuugg......................................................................  1      1   seq
....................ugcaggacgagaugaacAugg......................................................................  1      1   seq
....................ugcagUacgagaugaacuugg......................................................................  2      1   seq
....................ugcaggaUgagaugaacuugg......................................................................  4      1   seq
....................ugcaggacgagaugaacuugg......................................................................  18134  0   seq
....................ugcaggacgagaugaGcuugg......................................................................  2      1   seq
....................ugcaggacgagaugaacuNgg......................................................................  2      1   seq
....................ugcaggacgagaugUacuugg......................................................................  54     1   seq
....................ugcaggacgagaugaaUuugg......................................................................  5      1   seq
....................Cgcaggacgagaugaacuugg......................................................................  10     1   seq
....................ugUaggacgagaugaacuugg......................................................................  10     1   seq
....................ugcaggaAgagaugaacuugg......................................................................  2      1   seq
....................Agcaggacgagaugaacuugg......................................................................  7      1   seq
....................ugcaggCcgagaugaacuugg......................................................................  4      1   seq
....................ugcagCacgagaugaacuugg......................................................................  1      1   seq
....................ugcaggacgagauAaacuugg......................................................................  1      1   seq
....................ugcaggacgaAaugaacuugg......................................................................  3      1   seq
....................ugcaggaGgagaugaacuugg......................................................................  2      1   seq
....................ugcaggacgaUaugaacuugg......................................................................  1      1   seq
....................ugcaggacgCgaugaacuugg......................................................................  1      1   seq
....................ugcagAacgagaugaacuugg......................................................................  3      1   seq
....................ugcaggacgUgaugaacuugg......................................................................  1      1   seq
....................ugcaggacUagaugaacuugg......................................................................  1      1   seq
....................ugcaggacgagaugaacCugg......................................................................  3      1   seq
....................ugcaggacgagaugaacuugC......................................................................  14     1   seq
....................Ggcaggacgagaugaacuugg......................................................................  18     1   seq
....................ugcaggacgagaugaacuuUg......................................................................  1      1   seq
....................ugcUggacgagaugaacuugg......................................................................  1      1   seq
....................ugcaggacgagaugCacuugg......................................................................  62     1   seq
....................uAcaggacgagaugaacuugg......................................................................  10     1   seq
....................uCcaggacgagaugaacuugg......................................................................  2      1   seq
....................ugcaggacgagaugaacuugN......................................................................  1      1   seq
....................ugcaUgacgagaugaacuugg......................................................................  3      1   seq
....................Ngcaggacgagaugaacuugg......................................................................  91     1   seq
....................uNcaggacgagaugaacuugg......................................................................  2      1   seq
....................ugcaggacgGgaugaacuugg......................................................................  4      1   seq
....................ugcaggacgagaugaacuGgg......................................................................  1      1   seq
....................ugcaggacgagaugaacuugU......................................................................  57     1   seq
....................ugcGggacgagaugaacuugg......................................................................  9      1   seq
....................ugcaggacgagUugaacuugg......................................................................  2      1   seq
....................ugcaggacgagaugaaAuugg......................................................................  3      1   seq
....................ugcaggGcgagaugaacuugg......................................................................  2      1   seq
....................ugcCggacgagaugaacuugg......................................................................  2      1   seq
....................ugcaggacgagGugaacuugg......................................................................  7      1   seq
....................ugcaggacAagaugaacuugg......................................................................  5      1   seq
....................uUcaggacgagaugaacuugg......................................................................  1      1   seq
....................ugcaggacgagaugaacuuAg......................................................................  2      1   seq
....................ugcaggacgagaugNacuugg......................................................................  37     1   seq
....................ugcaggacgagaugGacuugg......................................................................  29     1   seq
....................ugcaggacgagCugaacuugg......................................................................  4      1   seq
....................ugcaggacgagaugaacuuggU.....................................................................  19     1   seq
....................ugcaggacgagaugCacuuggg.....................................................................  1      1   seq
....................ugcaggacgagaugaacuuggA.....................................................................  2      1   seq
....................ugcaggacgagaugaacuugAg.....................................................................  1      1   seq
....................Ngcaggacgagaugaacuuggg.....................................................................  1      1   seq
....................ugcaggacgagaugaacuuggg.....................................................................  9      0   seq
....................ugcaggacgagaugaacuuggggag..................................................................  1      0   seq
....................ugcaggacgagaugaacuuggggagg.................................................................  1      0   seq
.....................gcaggacgagaugaacuug.......................................................................  1      0   seq
.....................gcaggacgagaugaacuugg......................................................................  28     0   seq
.....................Acaggacgagaugaacuuggg.....................................................................  1      1   seq
.....................gcaggacAagaugaacuuggg.....................................................................  1      1   seq
.....................gcaggacgagaugaCcuuggg.....................................................................  2      1   seq
.....................gcaggacgagaugaNcuuggg.....................................................................  2      1   seq
.....................gcaggacgagaugaacuuggg.....................................................................  350    0   seq
.....................gcaggacgagaugaUcuuggg.....................................................................  2      1   seq
```

Mature Star

```
uucucgaaaaucauucagcuugcaggacgagaugaacuuggggaggaggcagcgaaacgaguucacaaccuugaagugccccucaaaccgcaguuuggccugcucuuuaca
.....................gcaggacgagaugaacuugggU.................................................................... 3 1 seq
......................caggacgagaugaacuugg...................................................................... 1 0 seq
......................caggacgagaugaacuuggg..................................................................... 1 0 seq
......................caggacgagaugaacuugggg.................................................................... 176 0 seq
......................Uaggacgagaugaacuugggg.................................................................... 1 1 seq
......................caggacgagaugCacuugggg.................................................................... 1 1 seq
......................caggacgagaugaaUuugggg.................................................................... 2 1 seq
......................caggacgGgaugaacuugggg.................................................................... 1 1 seq
......................caggacgagaugaaAuugggg.................................................................... 3 1 seq
......................Naggacgagaugaacuugggg.................................................................... 1 1 seq
......................caggacgaCaugaacuugggg.................................................................... 1 1 seq
......................caggacgagaugaacuugggC.................................................................... 1 1 seq
......................caggacgagaugaacuuggggU................................................................... 1 1 seq
.......................aggacgagaugaacuugg...................................................................... 4 0 seq
.......................aggacgagaugaacuugggg.................................................................... 2 0 seq
.......................aggacgagaugaacuugggga................................................................... 20 0 seq
.......................aggacgagaugaacuuggAga................................................................... 1 1 seq
.......................aggacgagaugaacuuggggaU.................................................................. 2 1 seq
........................ggacgagaugaacuuggggag.................................................................. 1 0 seq
........................ggacgagaugaacuuggggagU................................................................. 2 1 seq
........................ggacgagaugaacuuggggagg................................................................. 1 0 seq
.........................gacgagaugaacuuggggagg................................................................. 2 0 seq
..........................acgagaugaacuuggggagga................................................................ 5 0 seq
..........................acgagaugaacuuggggaggaggcag........................................................... 1 0 seq
...........................cgagaugaacuuggggaggag............................................................... 1 0 seq
...........................cgagaugaacuuggggaggagU.............................................................. 1 1 seq
............................gagaugaacuuggggaggagg.............................................................. 12 0 seq
............................gagaugaacuuggggaggaggU............................................................. 3 1 seq
.............................agaugaacuuggggaggaggU............................................................. 1 1 seq
.............................Cgaugaacuuggggaggaggc............................................................. 1 1 seq
.............................agaugaacuuggggaggaggc............................................................. 29 0 seq
..............................gaugaacuuggggaggaggc............................................................. 1 0 seq
..............................gaugaacuuggggaggaggca............................................................ 12 0 seq
..............................gaugaacuuggggaggaggcaU........................................................... 2 1 seq
...............................augaacuuggggaggaggcag........................................................... 1 0 seq
...............................augaacuuggggaggaggcagU.......................................................... 1 1 seq
................................ugaacuuggggaggaggcag........................................................... 2 0 seq
................................Ngaacuuggggaggaggcagc.......................................................... 1 1 seq
................................ugaacuuggggaggUggcagc.......................................................... 2 1 seq
................................ugaacuuggggaggaggcagA.......................................................... 1 1 seq
................................ugaacuuggggaggCggcagc.......................................................... 4 1 seq
................................ugaacuuggggaggaggcaAc.......................................................... 1 1 seq
................................ugaacuuggggaggGggcagc.......................................................... 1 1 seq
................................ugaacuuggggaggaggcagc.......................................................... 275 0 seq
................................ugaacuuggggaggaggcagcA......................................................... 2 1 seq
................................ugaacuuggggaggaggcagcU......................................................... 1 1 seq
................................ugaacuugggAaggaggcagcgaaac..................................................... 1 1 seq
................................ugaacuuggggaggaggcagcgaaaA..................................................... 1 1 seq
................................ugaacuuAgggaggaggcagcgaaac..................................................... 1 1 seq
................................ugaacuuggggaggaggGagcgaaac..................................................... 1 1 seq
................................ugaacuuggggaggaggcagcgaaac..................................................... 11 0 seq
................................ugaacuugAggaggaggcagcgaaac..................................................... 1 1 seq
................................ugaacuuggggaggaggcagcgaaacU.................................................... 1 1 seq
.................................gaacuuggggaggaggcagcg......................................................... 4 0 seq
.......................................ggggaggaggcagcgaaacga................................................... 4 0 seq
........................................gggaggaggcagcgaaacgag.................................................. 2 0 seq
...........................................aggaggcagcgaaacgaguuc............................................... 4 0 seq
...........................................aggaggcagcgaaacgaguucU.............................................. 2 1 seq
............................................ggaggcagcgaaacgaguuca.............................................. 1 0 seq
.............................................gaggcagcgaaacgaguucac............................................. 1 0 seq
..............................................aggcagcgaaacgaguucaca............................................ 2 0 seq
...............................................ggcagcgaaacgaguucacaa........................................... 2 0 seq
................................................gcagcgaaacgaguucacaac.......................................... 2 0 seq
.................................................cagcgaaacgaguucacaacc......................................... 1 0 seq
..................................................agcgaaacgaguucacaaccu........................................ 1 0 seq
..................................................agcgaaacgaguucacaaccuuU...................................... 1 1 seq
...................................................gcgaaacgaguucacaaccuu....................................... 1 0 seq
....................................................cgaaacgaguucacaaccuug...................................... 5 0 seq
.....................................................gaaacgaguucacaaccuuga..................................... 8 0 seq
.......................................................aacgaguucacaaccuugaag................................... 6 0 seq
```

Mature **Star**

```
uucucgaaaaucauucagcuugcaggacgagaugaacuuggggaggaggcagcgaaacgaguucacaaccuugaagugccccucaaaccgcaguuuggccugcucuuuaca
.......................................................aacgaguucacaaccuugaagu..................................  1   0   seq
........................................................acgaUuucacaaccuugaagu..................................  1   1   seq
........................................................acgaguucacaaccuugaagu..................................  3   0   seq
.........................................................cgaguucacaaccuugaagug.................................  3   0   seq
..........................................................gaguucacaaccuugaagugc................................  7   0   seq
...........................................................aguucacaaccuugaagugcc...............................  2   0   seq
............................................................guucacaaccuugaagugccc..............................  17  0   seq
.............................................................uucacaaccuugaagugcccc.............................  3   0   seq
..............................................................ucacaaccuugaagugccccu............................  1   0   seq
...............................................................cacaaccuugaaguUccccuc...........................  1   1   seq
...............................................................cacaaccuugaagugccccuc...........................  4   0   seq
................................................................acaaccuugaagugccccuc...........................  2   0   seq
...................................................................accuugaagugccccucaaac.......................  3   0   seq
....................................................................ccuugaagugccccucaaacc......................  1   0   seq
.....................................................................cuugaagugccccucaaaccg.....................  1   0   seq
......................................................................uugaagugccccucaaaccgc....................  1   0   seq
.......................................................................ugaagugccccucaaaccgca...................  2   0   seq
...........................................................................gugccccucaaaccgcaguuu...............  1   0   seq
............................................................................ugccccucaaaccgcaguuug..............  2   0   seq
.............................................................................gccccucaaaccgcaguuugg.............  1   0   seq
..............................................................................ccccucaaaccgcaguuuggc............  3   0   seq
...............................................................................cccucaaaccgcaguuuggcc...........  1   0   seq
..................................................................................ucaaaccgcaguuuggccugc........  8   0   seq
...................................................................................caaaccgcaguuuggccugcu.......  2   0   seq
......................................................................................accgcaguuuggccugcucuu....  3   0   seq
........................................................................................cgcaguuuggccugcucuuua..  1   0   seq
.........................................................................................gcaguuuggccugcucuuuac.  2   0   seq
..........................................................................................caguuuggccugcucuuuaca  7   0   seq
..........................................................................................Aaguuuggccugcucuuuaca  1   1   seq
```

Provisional ID	:	ML0081_3464
Score total	:	2555.9
Score for star read(s)	:	3.9
Score for read counts	:	2555.9
Score for mfe	:	-0.9
Score for randfold	:	-2.2
Score for cons. seed	:	-0.6
Total read count	:	5025
Mature read count	:	2774
Loop read count	:	2231
Star read count	:	20

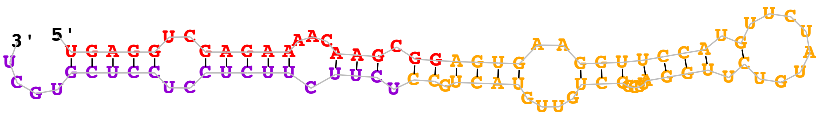

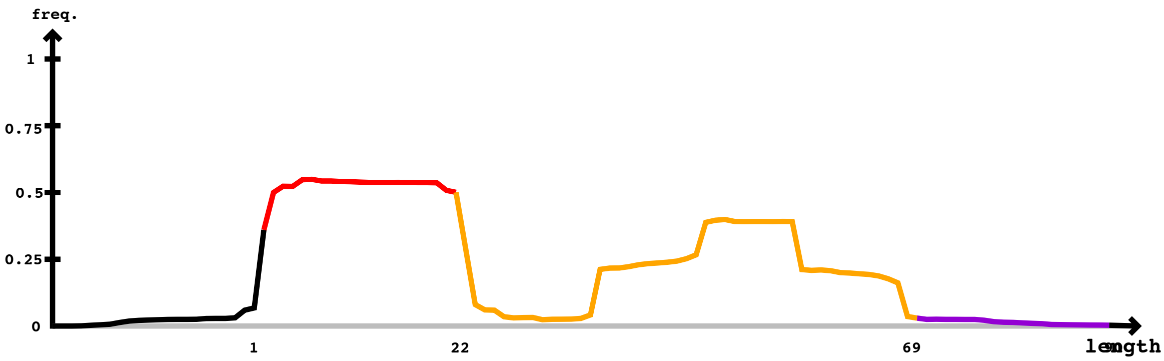

Mature **Star**

```
5'- cauucuuuggacauuccuccugaggucgagaaaacaagcggagugaagguuccauguucuaugucuuggauucggcuguuguacugccucuucuucuccuccucgugcuga   -3'   obs
    cauucuuuggacauuccuccugaggucgagaaaacaagcggagugaagguuccauguucuaugucuuggauucggcuguuguacugccucuucuucuccuccucgugcuga         exp
    ........(((......))).((((..(((((...(((.((((((..(((((((.(.........).))))....)))....)))).)).))).)))))..))))......   reads   mm   sample
    .auucuuuggacauuccuccug.........................................................................................   3       0    seq
    ..uucuuuggacauucUuccuga........................................................................................   1       1    seq
    ..uucuuuggacauuccuccuga........................................................................................   8       0    seq
    ..uucuuuggacauuccuccugU........................................................................................   2       1    seq
    ..uucuAuggacauuccuccugaggucgag.................................................................................   1       1    seq
    ...ucuuuggacauuccuccuga........................................................................................   1       0    seq
    ...ucuuuggacauuccuccugag.......................................................................................   8       0    seq
    ...ucuAuggacauuccuccugaggucgaga................................................................................   1       1    seq
    ....cuuuggacauuccuccugagg......................................................................................   11      0    seq
    ....cuuuggacauuccuccugaggu.....................................................................................   1       0    seq
    .....uuuggacauuccuccugagg......................................................................................   1       0    seq
    .....uuuggacauuccuccugaggu.....................................................................................   33      0    seq
    .....uuuggacauuccuccugaggA.....................................................................................   1       1    seq
    ......Nuggacauuccuccugagguc....................................................................................   1       1    seq
    ......uuggacauuccuccNgagguc....................................................................................   1       1    seq
    ......uuggacauuccuccugagguc....................................................................................   27      0    seq
    .......uggacauuccuccugaggucg...................................................................................   13      0    seq
    .......uggacauuccuccugaggucgU..................................................................................   1       1    seq
    ........ggacauuccuccCgaggucga..................................................................................   1       1    seq
    ........ggacauuccuccugaggucga..................................................................................   4       0    seq
    ........ggacauuccuccugaggucgaU.................................................................................   1       1    seq
    .........gacauuccuccugaggucgag.................................................................................   6       0    seq
    ..........acauuccuccugaggucAaga................................................................................   1       1    seq
    ..........acauuccuccugaggucgaga................................................................................   5       0    seq
    ...........cauuccuccugaggucgagaa...............................................................................   2       0    seq
    .............uuccuccugaggucgagaaaacaagcgg......................................................................   2       0    seq
    ..............uccuccugaggucgagaaaac............................................................................   2       0    seq
    ..............uccuccugaggucgagaaaacaagcgg......................................................................   6       0    seq
    ..............uccuccugaggucgagaaaacaagcgga.....................................................................   5       0    seq
    ..............uccuccugaggucgagaaaacaagcggagu...................................................................   2       0    seq
    ...............ccuccugaggucgagaaGaca...........................................................................   1       1    seq
    ...............ccuccugaggucgagaaaaca...........................................................................   1       0    seq
    .................uccugaggucgagaaGacaag.........................................................................   1       1    seq
```

Mature   Star

```
cauucuuuggacauuccuccugaggucgagaaaacaagcggagugaagguuccauguucuaugucuuggauucggcuguuguacugccucuucuucuccuccucgugcuga
.................uccugaggucgagaaaacaag.........................................................................   5     0   seq
.................uccugaggucgagaaaacaagcgg......................................................................   1     0   seq
.................uccugaggucgagaaaacaagcggag....................................................................   2     0   seq
.................uccugaggucgagaaaacaagcggagug..................................................................   3     0   seq
..................ccugaggucgagaaaacaag.........................................................................   1     0   seq
..................ccugaggucgagaaaacaagc........................................................................   147   0   seq
..................ccugaggucgagaaCacaagc........................................................................   1     1   seq
..................ccugaggucgagaaaacaagU........................................................................   1     1   seq
..................Ncugaggucgagaaaacaagc........................................................................   1     1   seq
..................ccugaggucgagaaGacaagc........................................................................   4     1   seq
...................cugaggucgagaaaacaagc........................................................................   2     0   seq
...................cugagguUgagaaaacaagcg.......................................................................   1     1   seq
...................cugaggucgagaaaacaagUg.......................................................................   5     1   seq
...................cugaggucgagaaaacaagcg.......................................................................   31    0   seq
...................cugaggucgagaaGacaagcg.......................................................................   2     1   seq
...................cugaggucgagaaaacaagcgU......................................................................   3     1   seq
...................cugaggucgagaaaacaagcgg......................................................................   2     0   seq
....................ugaggucgagaaaacaagcg.......................................................................   2     0   seq
....................ugaggucgagaaaacaagUg.......................................................................   1     1   seq
....................ugaggucgagaaaacaagcAg......................................................................   2     1   seq
....................ugaggGcgagaaaacaagcgg......................................................................   1     1   seq
....................ugaggucgagaaaaAaagcgg......................................................................   5     1   seq
....................ugaggucgagaaCacaagcgg......................................................................   1     1   seq
....................ugaggucgagaaaaUaagcgg......................................................................   6     1   seq
....................ugaggucgagaaaaNaagcgg......................................................................   4     1   seq
....................Cgaggucgagaaaacaagcgg......................................................................   1     1   seq
....................ugaggucgagaaaaGaagcgg......................................................................   3     1   seq
....................ugaAgucgagaaaacaagcgg......................................................................   1     1   seq
....................ugaggAcgagaaaacaagcgg......................................................................   2     1   seq
....................ugagAucgagaaaacaagcgg......................................................................   1     1   seq
....................ugaggucgagaaGacaagcgg......................................................................   10    1   seq
....................ugaggCcgagaaaacaagcgg......................................................................   4     1   seq
....................ugaggucgagaaaacaagcgA......................................................................   1     1   seq
....................ugaggucgagaaaacaagUgg......................................................................   67    1   seq
....................ugaggucgagaaaacaagcgg......................................................................   1425  0   seq
....................Ngaggucgagaaaacaagcgg......................................................................   6     1   seq
....................ugagguUgagaaaacaagcgg......................................................................   3     1   seq
....................ugaggucgagaaaacaagUgga.....................................................................   1     1   seq
....................ugaggucgagaaaacaagcgga.....................................................................   11    0   seq
....................ugaggucgagaaaacaagcggU.....................................................................   6     1   seq
....................ugaggucgagaaaacaagcggag....................................................................   1     0   seq
....................ugaggucgagaaaacaagUggagu...................................................................   1     1   seq
....................ugaggucgagaaaacaagcggagugaag...............................................................   11    0   seq
....................ugaggucgagaaaacaagcggagugaagU..............................................................   1     1   seq
....................ugaggucgagaaaacaagcggagugaagg..............................................................   2     0   seq
....................ugaggucgagaaaacaagcggagugaagguu............................................................   1     0   seq
.....................gaggucgagaaaacaagUgg......................................................................   1     1   seq
.....................gaggucgagaaaacaagcgg......................................................................   7     0   seq
.....................gaggucgagaaaacUagcgga.....................................................................   2     1   seq
.....................gaggucgagaaGacaagcgga.....................................................................   7     1   seq
.....................Aaggucgagaaaacaagcgga.....................................................................   3     1   seq
.....................gaggCcgagaaaacaagcgga.....................................................................   2     1   seq
.....................gaggucgagaaaacCagcgga.....................................................................   4     1   seq
.....................gaggucgagaaaacaagNgga.....................................................................   1     1   seq
.....................gaggucgagaaaacaagUgga.....................................................................   66    1   seq
.....................gaggucgagaaaacaagcAga.....................................................................   3     1   seq
.....................Naggucgagaaaacaagcgga.....................................................................   2     1   seq
.....................gaggucgagaaaacGagcgga.....................................................................   2     1   seq
.....................gaggucgagaaaacaagcggU.....................................................................   1     1   seq
.....................gaggucgagaaaacaagcgga.....................................................................   601   0   seq
.....................gaggucgagaaaaAaagcgga.....................................................................   1     1   seq
.....................gaggucgagaaaacaagcggaU....................................................................   1     1   seq
.....................gaggucgagaaaacaagcggag....................................................................   2     0   seq
.....................gaggucgagaaaacaagcggagug..................................................................   1     0   seq
.....................Naggucgagaaaacaagcggagugaag...............................................................   1     1   seq
.....................gaggucgagaaaacaagcggagugaag...............................................................   1     0   seq
.....................Naggucgagaaaacaagcggagugaagg..............................................................   1     1   seq
.....................gaggucgagaaaacaagcggagugaagg..............................................................   46    0   seq
.....................gaggucgagaaaacaagcggagugaaggu.............................................................   2     0   seq
......................aggucgagaaaacaagcgga.....................................................................   2     0   seq
```

Mature **Star**

```
cauucuuuggacauuccuccugaggucgagaaaacaagcggagugaagguuccauguucuaugucuuggauucggcuguuguacugccucuucuucuccuccucgugcuga
......................aggucgagaaaacaagUggag....................................................................    11    1    seq
......................aggucgagaaGacaagcggag....................................................................    1     1    seq
......................aggucgagaaaacaUgcggag....................................................................    1     1    seq
......................aggucgagaaaacaCgcggag....................................................................    1     1    seq
......................aggucgagaaaacaagcggag....................................................................    96    0    seq
......................aggucgagaaaacaagcggagu...................................................................    14    0    seq
......................aggucgagaaaacaUgcggagu...................................................................    1     1    seq
......................aggucgagaaaacaagcggagA...................................................................    1     1    seq
......................aggucgagaaaacaagcggagugaaggu.............................................................    2     0    seq
.......................Ugucgagaaaacaagcggagu...................................................................    1     1    seq
.......................ggucgagaaaacaagcggagu...................................................................    7     0    seq
.......................ggucgagaaaacaagcggaguU..................................................................    1     1    seq
........................gucgagaaaacaagcggagGg..................................................................    1     1    seq
........................gucgagaaaacaagUggagug..................................................................    4     1    seq
........................gucgagaaaacaagcggagug..................................................................    125   0    seq
........................gucgagaaaacaagcgAagug..................................................................    1     1    seq
........................gucgagaaaacaagGggagug..................................................................    1     1    seq
........................gucgagaaaacaagcggagugU.................................................................    11    1    seq
.........................ucgagaaaacaagcggagug..................................................................    1     0    seq
.........................ucgagaaaacaagcAgaguga.................................................................    1     1    seq
.........................ucgagaaaacaagcggaguga.................................................................    18    0    seq
..........................cgagaaaacaagcggagugaa................................................................    1     0    seq
...........................gagaaaacaagcggagugaag...............................................................    23    0    seq
...........................gagaaaacaagcUgagugaag...............................................................    1     1    seq
...........................gagaaaacaagcggagugaaC...............................................................    5     1    seq
............................agaaaacaagcggagugaagg..............................................................    1     0    seq
............................agaaCacaagcggagugaagg..............................................................    1     1    seq
............................agaaaacaagcggagugaaCg..............................................................    1     1    seq
.............................gaaaacaagcggagugaaggu.............................................................    1     0    seq
.............................gaaaacaagcggagugaaUgu.............................................................    1     1    seq
................................aacaagcggagugaaUguucc..........................................................    1     1    seq
.................................acaagcggagugaagguucca.........................................................    1     0    seq
..................................caagcggagugaagguuccau........................................................    1     0    seq
......................................cggagugaaCguuccauguuc....................................................    1     1    seq
......................................cggagugaagguuccauguuc....................................................    2     0    seq
.......................................ggagugaagguuccauguucu...................................................    3     0    seq
........................................gagugaagguuccauguucua..................................................    2     0    seq
..........................................gugaaUguuccauguucuaug................................................    1     1    seq
..........................................gugaaAguuccauguucuaug................................................    3     1    seq
..........................................gugaagguuccauguucuaug................................................    6     0    seq
...........................................ugaagguuccauguucuaugu...............................................    8     0    seq
............................................Naagguuccauguucuauguc..............................................    1     1    seq
............................................gaagguuccauguucuauguU..............................................    1     1    seq
............................................gaaAguuccauguucuauguc..............................................    2     1    seq
............................................gaaUguuccauguucuauguc..............................................    1     1    seq
............................................gaagguuccauguucuauguc..............................................    16    0    seq
.............................................aagguuccauguucuaugucu.............................................    5     0    seq
..............................................agguuccauguucuaugucu.............................................    1     0    seq
..............................................agguuccauguucuaugucuu............................................    6     0    seq
...............................................Cguuccauguucuaugucuug...........................................    1     1    seq
...............................................Aguuccauguucuaugucuug...........................................    3     1    seq
...............................................gguuccauguucuaugucuug...........................................    3     0    seq
................................................gCuccauguucuaugucuugg..........................................    1     1    seq
................................................guuccauguucuaugucuugU..........................................    1     1    seq
................................................guuccauguucuaugucuugg..........................................    42    0    seq
.................................................uuccauguucuaugucuugga.........................................    6     0    seq
.................................................uuccauguucuaugucuuggauucggc...................................    2     0    seq
..................................................uccauguucuaugucuuggauucggc...................................    15    0    seq
...................................................ccauguucuaugucuugUauu.......................................    1     1    seq
...................................................ccauguucuaugucuugAauu.......................................    1     1    seq
....................................................cauguucuaugucuugUauuc......................................    2     1    seq
.....................................................auguucuaugucuuNgauucg.....................................    1     1    seq
.....................................................auguucuaugucuugUauucg.....................................    4     1    seq
.....................................................auguucuaugucuuggauucg.....................................    8     0    seq
.....................................................auguucuaugucuuggauucgC....................................    1     1    seq
.....................................................auguucuaugucuuggauucggc...................................    1     0    seq
......................................................uguucuaugucuugUauucgg....................................    6     1    seq
......................................................uguucuaugucuugAauucgg....................................    2     1    seq
......................................................uguucuaugucuuggauucgg....................................    20    0    seq
......................................................uguucuaugucuugUauucggc...................................    2     1    seq
```

Mature **Star**

```
cauucuuuggacauuccuccugaggucgagaaaacaagcggagugaagguuccauguucuaugucuuggauucggcuguuguacugccucuucuucuccuccucgugcuga
......................................................Cguucuaugucuuggauucggc...................................	1	1	seq
......................................................uguucuaugucuuggauucggc...................................	36	0	seq
......................................................uguucuaugucCuggauucggc...................................	1	1	seq
......................................................Nguucuaugucuuggauucggc...................................	1	1	seq
......................................................uguucuaugucuuggauucggcugu................................	1	0	seq
......................................................uguucuaugucuuggauucggcuguug..............................	1	0	seq
......................................................uguucuaugucuuggauucggcuguugu.............................	1	0	seq
.......................................................guucuaugucuuggauucgg....................................	1	0	seq
.......................................................guucuauguAuuggauucggc...................................	1	1	seq
.......................................................guucuaugucuugAauucggc...................................	24	1	seq
.......................................................guucuaugucuugUauucggc...................................	92	1	seq
.......................................................guucCaugucuuggauucggc...................................	1	1	seq
.......................................................guucuaugucuuggUuucggc...................................	2	1	seq
.......................................................guucuaugucuuggauucggc...................................	769	0	seq
.......................................................guucuaugucuuggauucggU...................................	7	1	seq
.......................................................Nuucuaugucuuggauucggc...................................	6	1	seq
.......................................................guucuaugucuCggauucggc...................................	1	1	seq
.......................................................Auucuaugucuuggauucggc...................................	1	1	seq
.......................................................guucuaugucuuUgauucggc...................................	1	1	seq
.......................................................guucuaugucuuggCuucggc...................................	5	1	seq
.......................................................guucuauguGuuggauucggc...................................	1	1	seq
.......................................................guucuaugucuuggNuucggc...................................	3	1	seq
.......................................................guucuaugucuuggauucCgc...................................	1	1	seq
.......................................................guucuaugucuuggauucggG...................................	1	1	seq
.......................................................Uuucuaugucuuggauucggc...................................	2	1	seq
.......................................................guucuaugucuuggGuucggc...................................	3	1	seq
.......................................................guucuaugucuuggauucggcugu................................	2	0	seq
........................................................uucuaugucuuggauucggc...................................	5	0	seq
........................................................uucuaugucuugUauucggc...................................	1	1	seq
........................................................uucuaugucuuggauucggcu..................................	16	0	seq
........................................................uucuaugucuugUauucggcu..................................	2	1	seq
........................................................uucuaugucuugAauucggcu..................................	2	1	seq
.........................................................ucuaugucuuggauucggcug.................................	1	0	seq
.........................................................ucuaugucuuggauucggcugu................................	1	0	seq
..........................................................cuaugucuugAauucggcugu................................	2	1	seq
..........................................................cuaugucuuggauucggcugu................................	17	0	seq
..........................................................cuaugucuugUauucggcugu................................	1	1	seq
..........................................................cuaugucuuggauucggcuguu...............................	6	0	seq
..........................................................cuaugucuugUauucggcuguu...............................	1	1	seq
..........................................................cuaugucuuggauucggcuguuUu.............................	1	1	seq
...........................................................uaugucuuggauucNgcuguu...............................	1	1	seq
...........................................................uaugucuuggauucAgcuguu...............................	1	1	seq
...........................................................uaugucuuggauucggcuguu...............................	26	0	seq
...........................................................uaugucuuggauucggUuguu...............................	1	1	seq
...........................................................uaugucuugAauucggcuguu...............................	2	1	seq
...........................................................uaugucuuggauucgAcuguu...............................	1	1	seq
...........................................................uaugucuugUauucggcuguu...............................	5	1	seq
...........................................................uaugucuuggauucggcuguugua............................	1	0	seq
...........................................................uaugucuuggauucggcuguuguacugc........................	2	0	seq
...........................................................uaugucuuggauucggcuguuguacugU........................	1	1	seq
............................................................augucuugUauucggcuguug..............................	2	1	seq
............................................................augucuuggauucgAcuguug..............................	1	1	seq
............................................................augucuuggauucggcuguug..............................	19	0	seq
............................................................augucuugAauucggcuguug..............................	1	1	seq
............................................................augucuuggauucggcuguugu.............................	3	0	seq
.............................................................ugucuugUauucggcuguu...............................	1	1	seq
.............................................................ugucuugUauucggcuguugu.............................	2	1	seq
.............................................................ugucuuggauucggcuguugu.............................	11	0	seq
.............................................................ugucuuggauucggcuguuguacugccu......................	1	0	seq
..............................................................gucuugUauucggcuguugua............................	4	1	seq
..............................................................gucuugAauucggcuguugua............................	3	1	seq
..............................................................gucuuggauucggcuguugua............................	8	0	seq
...............................................................ucuuggauucggcuguugua............................	1	0	seq
...............................................................ucuuggauucggcugGuguac...........................	1	1	seq
...............................................................ucuugUauucggcuguuguac...........................	2	1	seq
...............................................................ucuuggauucggcuguuguac...........................	23	0	seq
...............................................................ucuugAauucggcuguuguac...........................	5	1	seq
...............................................................ucuuggauucggcuguuguacu..........................	1	0	seq
................................................................cuuggauucggcuguuguacu..........................	43	0	seq
................................................................cuugUauucggcuguuguacu..........................	10	1	seq
```

Mature | Star

cauucuuuggacauuccuccugaggucgagaaaacaagcggagugaaagguuccauguucuaugucuuggauucggcuguuguacugccucuucuucuccuccucgugcuga			
....cuuggauucggcugAuguacu..........................	2	1	seq
....Auuggauucggcuguuguacu..........................	2	1	seq
....uuggauucggcuguuguac...........................	1	0	seq
....uuggauucggcuguuguacu..........................	2	0	seq
....uugUauucggcuguuguacug.........................	2	1	seq
....uugAauucggcuguuguacug.........................	1	1	seq
....uuggauucggcuguuguacug.........................	74	0	seq
....uuggauucggcuguGguacug.........................	1	1	seq
....uuggauucggcuguuguacuU.........................	5	1	seq
....uuggauucggcuguuguacugA........................	5	1	seq
....uuggauucggcuguuguacugc........................	1	0	seq
....uuggauucggcuguuguacugU........................	8	1	seq
....uggauucggcuguuguacug.........................	1	0	seq
....uggauucggAuguuguacugc........................	1	1	seq
....uggauucggcuguuguacugc........................	540	0	seq
....uggauucggcuguuAuacugc........................	1	1	seq
....ugAauucggcuguuguacugc........................	30	1	seq
....uggauucggcuguuUuacugc........................	4	1	seq
....ugUauucggcuguuguacugc........................	70	1	seq
....uggauucggcuguuCuacugc........................	4	1	seq
....uggauucggcuguugAacugc........................	1	1	seq
....uggauucggcuguuguacuUc........................	2	1	seq
....uggauucggcuguuguacuAc........................	1	1	seq
....uggauucggcuguuguGcugc........................	1	1	seq
....uggauucggcuAuuguacugc........................	1	1	seq
....uggauucCgcuguuguacugc........................	1	1	seq
....Aggauucggcuguuguacugc........................	2	1	seq
....uggauucggcuguuguacugU........................	2	1	seq
....uggauucggcuguuguacugcU.......................	1	1	seq
....ggauucggcuguuguacugc........................	5	0	seq
....ggauucggcuguuguacugcc.......................	36	0	seq
....ggauucggcuguugAacugcc.......................	1	1	seq
....ggauucggcuguuguacuUcc.......................	2	1	seq
....gUauucggcuguuguacugcc.......................	2	1	seq
....ggauucggcuguuguacugccu......................	3	0	seq
....Nauucggcuguuguacugccu......................	1	1	seq
....gauucggcuguuguacCgccu......................	1	1	seq
....gauucggcuguuguacugccu......................	20	0	seq
....auucggcuguuguacugc........................	1	0	seq
....Nuucggcuguuguacugccuc.....................	1	1	seq
....auucggcuguuguacugccuc.....................	2	0	seq
....uucggcuguuguacugccucu....................	3	0	seq
....ucggcuguuguacugccucuucuucuUcu...........	1	1	seq
....ucggcuguuguacugccucuucuucuccucc.........	1	0	seq
....cggcuguuguacugccucuuc..................	1	0	seq
....cgAcuguuguacugccucuuc..................	1	1	seq
....gcuguuguacugccucuucuu................	15	0	seq
....gcuguuguacugccucuucuuU...............	2	1	seq
....cuguuguacugccucuucuu................	1	0	seq
....cuguuguacugccucuucuuc...............	27	0	seq
....uguuguacuUccucuucuucu..............	1	1	seq
....uguuguacugccucuucuucu..............	10	0	seq
....uguuguacugccucuucuucuU.............	1	1	seq
....uguuguacugccucuucuucuUcuccuc.......	1	1	seq
....guuguacugccucuucuucuc.............	2	0	seq
....guuguacuUccucuucuucuc.............	1	1	seq
....uuguacugccucuucuucucc............	10	0	seq
....uguacugccuUuucuucuccu...........	1	1	seq
....uguaUugccucuucuucuccu...........	1	1	seq
....uguacugccucuucuucuccu...........	6	0	seq
....guacugccucuucuucuccuc..........	7	0	seq
....uacugccucuucuucuccucc.........	14	0	seq
....uacugccucuucuucuccucU.........	1	1	seq
....acugccucuucuucuccuccu........	3	0	seq
....cugccucuucuucuccuccuc.......	2	0	seq
....cugccucuucuucuccuccucUu.....	1	1	seq
....ugccucuucuucuccuccucg......	1	0	seq
....ugccucuucuucuUcuccucg......	1	1	seq
....gccucuucuucuccuccucgu.....	2	0	seq
....ccucuucuucuccuccucgug....	1	0	seq

```
                    Mature                                                                Star
cauucuuuggacauuccuccugaggucgagaaaacaagcggagugaagguuccauguucuaugucuuggauucggcuguuguacugccucuucuucuccuccucgugcuga
......................................................................................ccucuucuucuccuccucgugU...    1    1    seq
.......................................................................................cucuucuucuccuccucgugc...    2    0    seq
........................................................................................ucuucuucuccuccucgugcu..    8    0    seq
........................................................................................ucuucuucuccuccucgugcuU.    1    1    seq
.........................................................................................cuucuucuccuccucgugcug.    1    0    seq
..........................................................................................uucuucuccuccucgugcuga    5    0    seq
..........................................................................................uucuucuccuccucgugcugU    1    1    seq
```

Provisional ID	:	ML0397_11858
Score total	:	2555.9
Score for star read(s)	:	3.9
Score for read counts	:	2555.9
Score for mfe	:	-0.9
Score for randfold	:	-2.2
Score for cons. seed	:	-0.6
Total read count	:	5025
Mature read count	:	2774
Loop read count	:	2231
Star read count	:	20

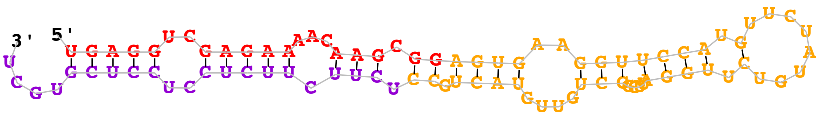

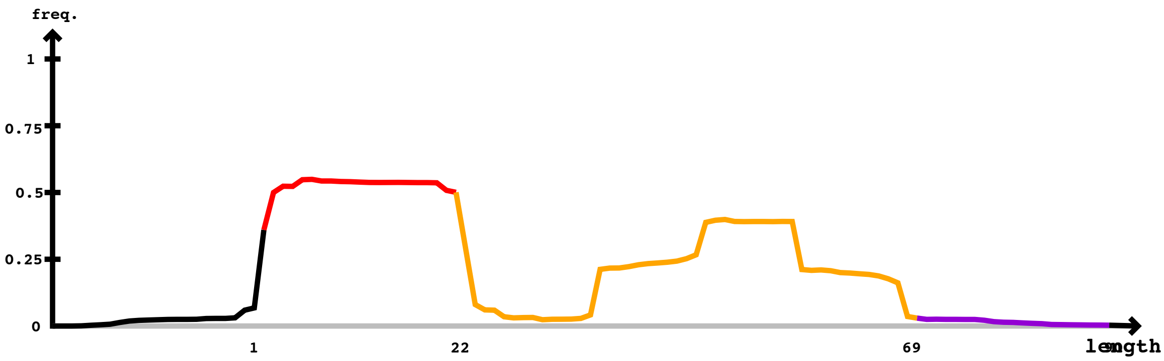

Mature **Star**

```
5'- cauucuuuggacauuccuccugaggucgagaaaacaagcggagugaagguuccauguucuaugucuuggauucggcuguuguacugccucuucuucuccuccucgugcuga  -3'   obs
    cauucuuuggacauuccuccugaggucgagaaaacaagcggagugaagguuccauguucuaugucuuggauucggcuguuguacugccucuucuucuccuccucgugcuga         exp
    ........(((......))).((((..(((((...(((.((((((..(((((((.(.........).))))....)))....)))).)).))).)))))..))))......   reads   mm   sample
    .auucuuuggacauuccuccug.........................................................................................   3       0    seq
    ..uucuuuggacauuccuccuga........................................................................................   8       0    seq
    ..uucuuuggacauucUuccuga........................................................................................   1       1    seq
    ..uucuuuggacauuccuccugU........................................................................................   2       1    seq
    ..uucuAuggacauuccuccugaggucgag.................................................................................   1       1    seq
    ...ucuuuggacauuccuccuga........................................................................................   1       0    seq
    ...ucuuuggacauuccuccugag.......................................................................................   8       0    seq
    ...ucuAuggacauuccuccugaggucgaga................................................................................   1       1    seq
    ....cuuuggacauuccuccugagg......................................................................................   11      0    seq
    ....cuuuggacauuccuccugaggu.....................................................................................   1       0    seq
    .....uuuggacauuccuccugagg......................................................................................   1       0    seq
    .....uuuggacauuccuccugaggA.....................................................................................   1       1    seq
    .....uuuggacauuccuccugaggu.....................................................................................   33      0    seq
    ......uuggacauuccuccugagguc....................................................................................   27      0    seq
    ......uuggacauuccuccNgagguc....................................................................................   1       1    seq
    ......Nuggacauuccuccugagguc....................................................................................   1       1    seq
    .......uggacauuccuccugaggucg...................................................................................   13      0    seq
    .......uggacauuccuccugaggucgU..................................................................................   1       1    seq
    ........ggacauuccuccCgaggucga..................................................................................   1       1    seq
    ........ggacauuccuccugaggucga..................................................................................   4       0    seq
    ........ggacauuccuccugaggucgaU.................................................................................   1       1    seq
    .........gacauuccuccugaggucgag.................................................................................   6       0    seq
    ..........acauuccuccugaggucgaga................................................................................   5       0    seq
    ..........acauuccuccugaggucAaga................................................................................   1       1    seq
    ...........cauuccuccugaggucgagaa...............................................................................   2       0    seq
    .............uuccuccugaggucgagaaaacaagcgg......................................................................   2       0    seq
    ..............uccuccugaggucgagaaaac............................................................................   2       0    seq
    ..............uccuccugaggucgagaaaacaagcgg......................................................................   6       0    seq
    ..............uccuccugaggucgagaaaacaagcgga.....................................................................   5       0    seq
    ..............uccuccugaggucgagaaaacaagcggagu...................................................................   2       0    seq
    ...............ccuccugaggucgagaaGaca...........................................................................   1       1    seq
    ...............ccuccugaggucgagaaaaca...........................................................................   1       0    seq
    .................uccugaggucgagaaGacaag.........................................................................   1       1    seq
```

```
                    Mature                                                        Star
cauucuuuggacauuccuccugaggucgagaaaacaagcggagugaagguuccauguucuaugucuuggauucggcuguuguacugccucuucuucuccuccucgugcuga
.................uccugaggucgagaaaacaag.........................................................................   5      0     seq
.................uccugaggucgagaaaacaagcgg......................................................................   1      0     seq
.................uccugaggucgagaaaacaagcggag....................................................................   2      0     seq
.................uccugaggucgagaaaacaagcggagug.................................................................   3      0     seq
..................ccugaggucgagaaaacaag.........................................................................   1      0     seq
..................ccugaggucgagaaaacaagc........................................................................   147    0     seq
..................ccugaggucgagaaCacaagc........................................................................   1      1     seq
..................ccugaggucgagaaGacaagc........................................................................   4      1     seq
..................ccugaggucgagaaaacaagU........................................................................   1      1     seq
..................Ncugaggucgagaaaacaagc........................................................................   1      1     seq
...................cugaggucgagaaaacaagc........................................................................   2      0     seq
...................cugaggucgagaaGacaagcg.......................................................................   2      1     seq
...................cugagguUgagaaaacaagcg.......................................................................   1      1     seq
...................cugaggucgagaaaacaagcg.......................................................................   31     0     seq
...................cugaggucgagaaaacaagUg.......................................................................   5      1     seq
...................cugaggucgagaaaacaagcgg......................................................................   2      0     seq
...................cugaggucgagaaaacaagcgU......................................................................   3      1     seq
....................ugaggucgagaaaacaagUg.......................................................................   1      1     seq
....................ugaggucgagaaaacaagcg.......................................................................   2      0     seq
....................ugaggucgagaaaaGaagcgg......................................................................   3      1     seq
....................ugaggucgagaaaacaagcgA......................................................................   1      1     seq
....................ugaggucgagaaaaNaagcgg......................................................................   4      1     seq
....................ugaggGcgagaaaacaagcgg......................................................................   1      1     seq
....................ugaggucgagaaaacaagcAg......................................................................   2      1     seq
....................ugaggucgagaaaaUaagcgg......................................................................   6      1     seq
....................Ngaggucgagaaaacaagcgg......................................................................   6      1     seq
....................ugaggAcgagaaaacaagcgg......................................................................   2      1     seq
....................ugaggCcgagaaaacaagcgg......................................................................   4      1     seq
....................ugaggucgagaaaaAaagcgg......................................................................   5      1     seq
....................ugaAgucgagaaaacaagcgg......................................................................   1      1     seq
....................ugaggucgagaaaacaagcgg......................................................................   1425   0     seq
....................ugaggucgagaaCacaagcgg......................................................................   1      1     seq
....................ugaggucgagaaaacaagUgg......................................................................   67     1     seq
....................Cgaggucgagaaaacaagcgg......................................................................   1      1     seq
....................ugagAucgagaaaacaagcgg......................................................................   1      1     seq
....................ugagguUgagaaaacaagcgg......................................................................   3      1     seq
....................ugaggucgagaaGacaagcgg......................................................................   10     1     seq
....................ugaggucgagaaaacaagUgga.....................................................................   1      1     seq
....................ugaggucgagaaaacaagcggU.....................................................................   6      1     seq
....................ugaggucgagaaaacaagcgga.....................................................................   11     0     seq
....................ugaggucgagaaaacaagcggag....................................................................   1      0     seq
....................ugaggucgagaaaacaagUggagu...................................................................   1      1     seq
....................ugaggucgagaaaacaagcggagugaag...............................................................   11     0     seq
....................ugaggucgagaaaacaagcggagugaagg..............................................................   2      0     seq
....................ugaggucgagaaaacaagcggagugaagU..............................................................   1      1     seq
....................ugaggucgagaaaacaagcggagugaagguu............................................................   1      0     seq
.....................gaggucgagaaaacaagcgg......................................................................   7      0     seq
.....................gaggucgagaaaacaagUgg......................................................................   1      1     seq
.....................gaggucgagaaaacCagcgga.....................................................................   4      1     seq
.....................gaggucgagaaGacaagcgga.....................................................................   7      1     seq
.....................gaggucgagaaaacaagcggU.....................................................................   1      1     seq
.....................gaggucgagaaaacaagNgga.....................................................................   1      1     seq
.....................gaggucgagaaaacUagcgga.....................................................................   2      1     seq
.....................gaggucgagaaaacGagcgga.....................................................................   2      1     seq
.....................gaggucgagaaaacaagUgga.....................................................................   66     1     seq
.....................Aaggucgagaaaacaagcgga.....................................................................   3      1     seq
.....................gaggucgagaaaacaagcgga.....................................................................   601    0     seq
.....................gaggucgagaaaacaagcAga.....................................................................   3      1     seq
.....................gaggCcgagaaaacaagcgga.....................................................................   2      1     seq
.....................Naggucgagaaaacaagcgga.....................................................................   2      1     seq
.....................gaggucgagaaaaAaagcgga.....................................................................   1      1     seq
.....................gaggucgagaaaacaagcggaU....................................................................   1      1     seq
.....................gaggucgagaaaacaagcggag....................................................................   2      0     seq
.....................gaggucgagaaaacaagcggagug..................................................................   1      0     seq
.....................gaggucgagaaaacaagcggagugaag...............................................................   1      0     seq
.....................Naggucgagaaaacaagcggagugaag...............................................................   1      1     seq
.....................Naggucgagaaaacaagcggagugaagg..............................................................   1      1     seq
.....................gaggucgagaaaacaagcggagugaagg..............................................................   46     0     seq
.....................gaggucgagaaaacaagcggagugaaggu.............................................................   2      0     seq
......................aggucgagaaaacaagcgga.....................................................................   2      0     seq
```

Mature Star

```
cauucuuuggacauuccuccugaggucgagaaaacaagcggagugaagguuccauguucuaugucuuggauucggcuguuguacugccucuucuucuccuccucgugcuga
......................aggucgagaaGacaagcggag....................................................................  1    1  seq
......................aggucgagaaaacaUgcggag....................................................................  1    1  seq
......................aggucgagaaaacaagcggag....................................................................  96   0  seq
......................aggucgagaaaacaagUggag....................................................................  11   1  seq
......................aggucgagaaaacaCgcggag....................................................................  1    1  seq
......................aggucgagaaaacaagcggagA...................................................................  1    1  seq
......................aggucgagaaaacaUgcggagu...................................................................  1    1  seq
......................aggucgagaaaacaagcggagu...................................................................  14   0  seq
......................aggucgagaaaacaagcggagugaaggu.............................................................  2    0  seq
.......................ggucgagaaaacaagcggagu...................................................................  7    0  seq
.......................Ugucgagaaaacaagcggagu...................................................................  1    1  seq
.......................ggucgagaaaacaagcggaguU..................................................................  1    1  seq
........................gucgagaaaacaagcggagug..................................................................  125  0  seq
........................gucgagaaaacaagcggagGg..................................................................  1    1  seq
........................gucgagaaaacaagcgAagug..................................................................  1    1  seq
........................gucgagaaaacaagUggagug..................................................................  4    1  seq
........................gucgagaaaacaagGggagug..................................................................  1    1  seq
........................gucgagaaaacaagcggagugU.................................................................  11   1  seq
.........................ucgagaaaacaagcggagug..................................................................  1    0  seq
.........................ucgagaaaacaagcAgaguga.................................................................  1    1  seq
.........................ucgagaaaacaagcggaguga.................................................................  18   0  seq
..........................cgagaaaacaagcggagugaa................................................................  1    0  seq
...........................gagaaaacaagcUgagugaag...............................................................  1    1  seq
...........................gagaaaacaagcggagugaaC...............................................................  5    1  seq
...........................gagaaaacaagcggagugaag...............................................................  23   0  seq
............................agaaCacaagcggagugaagg..............................................................  1    1  seq
............................agaaaacaagcggagugaaCg..............................................................  1    1  seq
............................agaaaacaagcggagugaagg..............................................................  1    0  seq
.............................gaaaacaagcggagugaaggu.............................................................  1    0  seq
.............................gaaaacaagcggagugaaUgu.............................................................  1    1  seq
................................aacaagcggagugaaUguucc..........................................................  1    1  seq
.................................acaagcggagugaagguucca.........................................................  1    0  seq
..................................caagcggagugaagguuccau........................................................  1    0  seq
......................................cggagugaaCguuccauguuc....................................................  1    1  seq
......................................cggagugaagguuccauguuc....................................................  2    0  seq
.......................................ggagugaagguuccauguucu...................................................  3    0  seq
........................................gagugaagguuccauguucua..................................................  2    0  seq
..........................................gugaaUguuccauguucuaug................................................  1    1  seq
..........................................gugaaAguuccauguucuaug................................................  3    1  seq
..........................................gugaagguuccauguucuaug................................................  6    0  seq
...........................................ugaagguuccauguucuaugu...............................................  8    0  seq
............................................gaaUguuccauguucuauguc..............................................  1    1  seq
............................................Naagguuccauguucuauguc..............................................  1    1  seq
............................................gaagguuccauguucuauguU..............................................  1    1  seq
............................................gaagguuccauguucuauguc..............................................  16   0  seq
............................................gaaAguuccauguucuauguc..............................................  2    1  seq
.............................................aagguuccauguucuaugucu.............................................  5    0  seq
..............................................agguuccauguucuaugucu.............................................  1    0  seq
..............................................agguuccauguucuaugucuu............................................  6    0  seq
...............................................gguuccauguucuaugucuug...........................................  3    0  seq
...............................................Aguuccauguucuaugucuug...........................................  3    1  seq
...............................................Cguuccauguucuaugucuug...........................................  1    1  seq
................................................gCuccauguucuaugucuugg..........................................  1    1  seq
................................................guuccauguucuaugucuugg..........................................  42   0  seq
................................................guuccauguucuaugucuugU..........................................  1    1  seq
.................................................uuccauguucuaugucuugga.........................................  6    0  seq
.................................................uuccauguucuaugucuuggauucggc...................................  2    0  seq
..................................................uccauguucuaugucuuggauucggc...................................  15   0  seq
...................................................ccauguucuaugucuugAauu.......................................  1    1  seq
...................................................ccauguucuaugucuugUauu.......................................  1    1  seq
....................................................cauguucuaugucuugUauuc......................................  2    1  seq
.....................................................auguucuaugucuuggauucg.....................................  8    0  seq
.....................................................auguucuaugucuugUauucg.....................................  4    1  seq
.....................................................auguucuaugucuuNgauucg.....................................  1    1  seq
.....................................................auguucuaugucuuggauucgC....................................  1    1  seq
.....................................................auguucuaugucuuggauucggc...................................  1    0  seq
......................................................uguucuaugucuuggauucgg....................................  20   0  seq
......................................................uguucuaugucuugAauucgg....................................  2    1  seq
......................................................uguucuaugucuugUauucgg....................................  6    1  seq
......................................................uguucuaugucuuggauucggc...................................  36   0  seq
```

Mature   Star

```
cauucuuuggacauuccuccugaggucgagaaaacaagcggagugaagguuccauguucuaugucuuggauucggcuguuguacugccucuucuucuccuccucgugcuga
......................................................uguucuaugucuugUauucggc...................................	2	1	seq
......................................................Nguucuaugucuuggauucggc...................................	1	1	seq
......................................................Cguucuaugucuuggauucggc...................................	1	1	seq
......................................................uguucuaugucCuggauucggc...................................	1	1	seq
......................................................uguucuaugucuuggauucggcugu................................	1	0	seq
......................................................uguucuaugucuuggauucggcuguug..............................	1	0	seq
......................................................uguucuaugucuuggauucggcuguugu.............................	1	0	seq
.......................................................guucuaugucuuggauucgg....................................	1	0	seq
.......................................................Nuucuaugucuuggauucggc...................................	6	1	seq
.......................................................guucuaugucuuggauucggc...................................	769	0	seq
.......................................................Auucuaugucuuggauucggc...................................	1	1	seq
.......................................................guucuaugucuCggauucggc...................................	1	1	seq
.......................................................guucuaugucuuUgauucggc...................................	1	1	seq
.......................................................guucuaugucuuggauucCgc...................................	1	1	seq
.......................................................guucuaugucuuggauucggG...................................	1	1	seq
.......................................................guucuaugucuuggCuucggc...................................	5	1	seq
.......................................................guucuaugucuuggUuucggc...................................	2	1	seq
.......................................................Uuucuaugucuuggauucggc...................................	2	1	seq
.......................................................guucCaugucuuggauucggc...................................	1	1	seq
.......................................................guucuaugucuuggGuucggc...................................	3	1	seq
.......................................................guucuaugucuugUauucggc...................................	92	1	seq
.......................................................guucuaugucuuggauucggU...................................	7	1	seq
.......................................................guucuauguAuuggauucggc...................................	1	1	seq
.......................................................guucuaugucuugAauucggc...................................	24	1	seq
.......................................................guucuauguGuuggauucggc...................................	1	1	seq
.......................................................guucuaugucuuggNuucggc...................................	3	1	seq
.......................................................guucuaugucuuggauucggcugu................................	2	0	seq
........................................................uucuaugucuuggauucggc...................................	5	0	seq
........................................................uucuaugucuugUauucggc...................................	1	1	seq
........................................................uucuaugucuuggauucggcu..................................	16	0	seq
........................................................uucuaugucuugAauucggcu..................................	2	1	seq
........................................................uucuaugucuugUauucggcu..................................	2	1	seq
.........................................................ucuaugucuuggauucggcug.................................	1	0	seq
.........................................................ucuaugucuuggauucggcugu................................	1	0	seq
..........................................................cuaugucuugUauucggcugu................................	1	1	seq
..........................................................cuaugucuuggauucggcugu................................	17	0	seq
..........................................................cuaugucuugAauucggcugu................................	2	1	seq
..........................................................cuaugucuugUauucggcuguu...............................	1	1	seq
..........................................................cuaugucuuggauucggcuguu...............................	6	0	seq
..........................................................cuaugucuuggauucggcuguuUu.............................	1	1	seq
...........................................................uaugucuuggauucggUuguu...............................	1	1	seq
...........................................................uaugucuuggauucgAcuguu...............................	1	1	seq
...........................................................uaugucuuggauucggcuguu...............................	26	0	seq
...........................................................uaugucuuggauucAgcuguu...............................	1	1	seq
...........................................................uaugucuugAauucggcuguu...............................	2	1	seq
...........................................................uaugucuugUauucggcuguu...............................	5	1	seq
...........................................................uaugucuuggauucNgcuguu...............................	1	1	seq
...........................................................uaugucuuggauucggcuguugua............................	1	0	seq
...........................................................uaugucuuggauucggcuguuguacugU........................	1	1	seq
...........................................................uaugucuuggauucggcuguuguacugc........................	2	0	seq
............................................................augucuugUauucggcuguug..............................	2	1	seq
............................................................augucuuggauucgAcuguug..............................	1	1	seq
............................................................augucuuggauucggcuguug..............................	19	0	seq
............................................................augucuugAauucggcuguug..............................	1	1	seq
............................................................augucuuggauucggcuguugu.............................	3	0	seq
.............................................................ugucuugUauucggcuguu...............................	1	1	seq
.............................................................ugucuugUauucggcuguugu.............................	2	1	seq
.............................................................ugucuuggauucggcuguugu.............................	11	0	seq
.............................................................ugucuuggauucggcuguuguacugccu......................	1	0	seq
..............................................................gucuugUauucggcuguugua............................	4	1	seq
..............................................................gucuuggauucggcuguugua............................	8	0	seq
..............................................................gucuugAauucggcuguugua............................	3	1	seq
...............................................................ucuuggauucggcuguugua............................	1	0	seq
...............................................................ucuuggauucggcuguuguac...........................	23	0	seq
...............................................................ucuugAauucggcuguuguac...........................	5	1	seq
...............................................................ucuugUauucggcuguuguac...........................	2	1	seq
...............................................................ucuuggauucggcugGuguac...........................	1	1	seq
...............................................................ucuuggauucggcuguuguacu..........................	1	0	seq
................................................................cuuggauucggcuguuguacu..........................	43	0	seq
................................................................cuugUauucggcuguuguacu..........................	10	1	seq
```

Mature **Star**

```
cauucuuuggacauuccuccugaggucgagaaaacaagcggagugaagguuccauguucuaugucuuggauucggcuguuguacugccucuucuucuccuccucgugcuga
................................................................Auuggauucggcuguuguacu..........................	2	1	seq
................................................................cuuggauucggcugAuguacu..........................	2	1	seq
.................................................................uuggauucggcuguuguac...........................	1	0	seq
.................................................................uuggauucggcuguuguacu..........................	2	0	seq
.................................................................uuggauucggcuguuguacuU.........................	5	1	seq
.................................................................uugAauucggcuguuguacug.........................	1	1	seq
.................................................................uuggauucggcuguGguacug.........................	1	1	seq
.................................................................uuggauucggcuguuguacug.........................	74	0	seq
.................................................................uugUauucggcuguuguacug.........................	2	1	seq
.................................................................uuggauucggcuguuguacugA........................	5	1	seq
.................................................................uuggauucggcuguuguacugU........................	8	1	seq
.................................................................uuggauucggcuguuguacugc........................	1	0	seq
..................................................................uggauucggcuguuguacug.........................	1	0	seq
..................................................................uggauucggcuguuguacugU........................	2	1	seq
..................................................................ugAauucggcuguuguacugc........................	30	1	seq
..................................................................uggauucggcuguuguacuUc........................	2	1	seq
..................................................................uggauucggcuguuguacugc........................	540	0	seq
..................................................................uggauucggcuguuCuacugc........................	4	1	seq
..................................................................uggauucggcuAuuguacugc........................	1	1	seq
..................................................................uggauucggAuguuguacugc........................	1	1	seq
..................................................................ugUauucggcuguuguacugc........................	70	1	seq
..................................................................uggauucggcuguuUuacugc........................	4	1	seq
..................................................................uggauucggcuguuguGcugc........................	1	1	seq
..................................................................uggauucggcuguuguacuAc........................	1	1	seq
..................................................................uggauucggcuguugAacugc........................	1	1	seq
..................................................................uggauucCgcuguuguacugc........................	1	1	seq
..................................................................uggauucggcuguuAuacugc........................	1	1	seq
..................................................................Aggauucggcuguuguacugc........................	2	1	seq
..................................................................uggauucggcuguuguacugcU.......................	1	1	seq
...................................................................ggauucggcuguuguacugc........................	5	0	seq
...................................................................ggauucggcuguuguacuUcc.......................	2	1	seq
...................................................................gUauucggcuguuguacugcc.......................	2	1	seq
...................................................................ggauucggcuguugAacugcc.......................	1	1	seq
...................................................................ggauucggcuguuguacugcc.......................	36	0	seq
...................................................................ggauucggcuguuguacugccu......................	3	0	seq
....................................................................gauucggcuguuguacugccu......................	20	0	seq
....................................................................gauucggcuguuguacCgccu......................	1	1	seq
....................................................................Nauucggcuguuguacugccu......................	1	1	seq
.....................................................................auucggcuguuguacugc........................	1	0	seq
.....................................................................Nuucggcuguuguacugccuc.....................	1	1	seq
.....................................................................auucggcuguuguacugccuc.....................	2	0	seq
......................................................................uucggcuguuguacugccucu....................	3	0	seq
.......................................................................ucggcuguuguacugccucuucuucuUcu...........	1	1	seq
.......................................................................ucggcuguuguacugccucuucuucuccucc.........	1	0	seq
........................................................................cgAcuguuguacugccucuuc..................	1	1	seq
........................................................................cggcuguuguacugccucuuc..................	1	0	seq
..........................................................................gcuguuguacugccucuucuu................	15	0	seq
..........................................................................gcuguuguacugccucuucuuU...............	2	1	seq
...........................................................................cuguuguacugccucuucuu................	1	0	seq
...........................................................................cuguuguacugccucuucuuc...............	27	0	seq
............................................................................uguuguacugccucuucuucu..............	10	0	seq
............................................................................uguuguacuUccucuucuucu..............	1	1	seq
............................................................................uguuguacugccucuucuucuU.............	1	1	seq
............................................................................uguuguacugccucuucuucuUcuccuc.......	1	1	seq
.............................................................................guuguacugccucuucuucuc.............	2	0	seq
.............................................................................guuguacuUccucuucuucuc.............	1	1	seq
..............................................................................uuguacugccucuucuucucc............	10	0	seq
...............................................................................uguacugccucuucuucuccu...........	6	0	seq
...............................................................................uguaUugccucuucuucuccu...........	1	1	seq
...............................................................................uguacugccuUuucuucuccu...........	1	1	seq
................................................................................guacugccucuucuucuccuc..........	7	0	seq
.................................................................................uacugccucuucuucuccucc.........	14	0	seq
.................................................................................uacugccucuucuucuccucU.........	1	1	seq
..................................................................................acugccucuucuucuccuccu........	3	0	seq
...................................................................................cugccucuucuucuccuccuc.......	2	0	seq
...................................................................................cugccucuucuucuccuccucUu.....	1	1	seq
....................................................................................ugccucuucuucucUuccucg......	1	1	seq
....................................................................................ugccucuucuucuccuccucg......	1	0	seq
.....................................................................................gccucuucuucuccuccucgu.....	2	0	seq
......................................................................................ccucuucuucuccuccucgug....	1	0	seq
```

```
                    Mature                                                            Star
cauucuuuggacauuccuccugaggucgagaaaacaagcggagugaagguuccauguucuaugucuuggauucggcuguuguacugccucuucuucuccuccucgugcuga
......................................................................................ccucuucuucuccuccucgugU...    1    1    seq
.......................................................................................cucuucuucuccuccucgugc...    2    0    seq
........................................................................................ucuucuucuccuccucgugcu..    8    0    seq
........................................................................................ucuucuucuccuccucgugcuU.    1    1    seq
.........................................................................................cuucuucuccuccucgugcug.    1    0    seq
..........................................................................................uucuucuccuccucgugcuga    5    0    seq
..........................................................................................uucuucuccuccucgugcugU    1    1    seq
```

Provisional ID : ML4635_47451
Score total : 2555.9
Score for star read(s) : 3.9
Score for read counts : 2555.9
Score for mfe : -0.9
Score for randfold : -2.2
Score for cons. seed : -0.6
Total read count : 5025
Mature read count : 2774
Loop read count : 2231
Star read count : 20

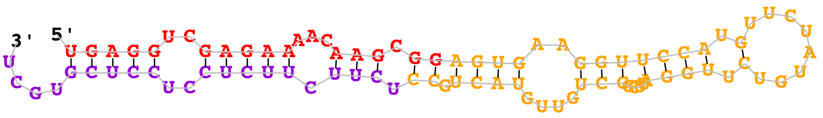

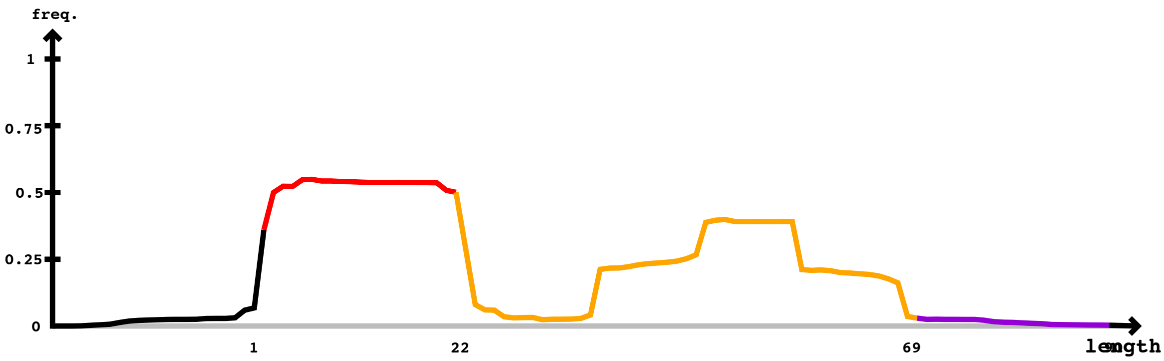

Mature **Star**

```
5'- cauucuuuggacauuccuccugaggucgagaaaacaagcggagugaagguuccauguucuaugucuuggauucggcuguuguacugccucuucuucuccuccucgugcuga   -3'   obs
    cauucuuuggacauuccuccugaggucgagaaaacaagcggagugaagguuccauguucuaugucuuggauucggcuguuguacugccucuucuucuccuccucgugcuga         exp
    ........(((......))).((((..(((((...(((.((((((..(((((((.(.........).))))....)))....)))).)).))).))))).)))).......   reads   mm   sample
    .auucuuuggacauuccuccug.........................................................................................   3       0    seq
    ..uucuuuggacauuccuccugU........................................................................................   2       1    seq
    ..uucuuuggacauucUuccuga........................................................................................   1       1    seq
    ..uucuuuggacauuccuccuga........................................................................................   8       0    seq
    ..uucuAuggacauuccuccugaggucgag.................................................................................   1       1    seq
    ...ucuuuggacauuccuccuga........................................................................................   1       0    seq
    ...ucuuuggacauuccuccugag.......................................................................................   8       0    seq
    ...ucuAuggacauuccuccugaggucgaga................................................................................   1       1    seq
    ....cuuuggacauuccuccugagg......................................................................................   11      0    seq
    ....cuuuggacauuccuccugaggu.....................................................................................   1       0    seq
    .....uuuggacauuccuccugagg......................................................................................   1       0    seq
    .....uuuggacauuccuccugaggu.....................................................................................   33      0    seq
    .....uuuggacauuccuccugaggA.....................................................................................   1       1    seq
    ......uuggacauuccuccNgagguc....................................................................................   1       1    seq
    ......Nuggacauuccuccugagguc....................................................................................   1       1    seq
    ......uuggacauuccuccugagguc....................................................................................   27      0    seq
    .......uggacauuccuccugaggucg...................................................................................   13      0    seq
    .......uggacauuccuccugaggucgU..................................................................................   1       1    seq
    ........ggacauuccuccugaggucga..................................................................................   4       0    seq
    ........ggacauuccuccCgaggucga..................................................................................   1       1    seq
    ........ggacauuccuccugaggucgaU.................................................................................   1       1    seq
    .........gacauuccuccugaggucgag.................................................................................   6       0    seq
    ..........acauuccuccugaggucgaga................................................................................   5       0    seq
    ..........acauuccuccugaggucAaga................................................................................   1       1    seq
    ...........cauuccuccugaggucgagaa...............................................................................   2       0    seq
    .............uuccuccugaggucgagaaaacaagcgg......................................................................   2       0    seq
    ..............uccuccugaggucgagaaaac............................................................................   2       0    seq
    ..............uccuccugaggucgagaaaacaagcgg......................................................................   6       0    seq
    ..............uccuccugaggucgagaaaacaagcgga.....................................................................   5       0    seq
    ..............uccuccugaggucgagaaaacaagcggagu...................................................................   2       0    seq
    ...............ccuccugaggucgagaaGaca...........................................................................   1       1    seq
    ...............ccuccugaggucgagaaaaca...........................................................................   1       0    seq
    .................uccugaggucgagaaaacaag.........................................................................   5       0    seq
```

Mature Star

```
cauucuuuggacauuccuccugaggucgagaaaacaagcggagugaagguuccauguucuaugucuuggauucggcuguuguacugccucuucuucuccuccucgugcuga
.................uccugaggucgagaaGacaag.........................................................................   1     1     seq
.................uccugaggucgagaaaacaagcgg......................................................................   1     0     seq
.................uccugaggucgagaaaacaagcggag....................................................................   2     0     seq
.................uccugaggucgagaaaacaagcggagug..................................................................   3     0     seq
..................ccugaggucgagaaaacaag.........................................................................   1     0     seq
..................ccugaggucgagaaCacaagc........................................................................   1     1     seq
..................Ncugaggucgagaaaacaagc........................................................................   1     1     seq
..................ccugaggucgagaaGacaagc........................................................................   4     1     seq
..................ccugaggucgagaaaacaagU........................................................................   1     1     seq
..................ccugaggucgagaaaacaagc........................................................................   147   0     seq
...................cugaggucgagaaaacaagc........................................................................   2     0     seq
...................cugaggucgagaaGacaagcg.......................................................................   2     1     seq
...................cugaggucgagaaaacaagUg.......................................................................   5     1     seq
...................cugaggucgagaaaacaagcg.......................................................................   31    0     seq
...................cugagguUgagaaaacaagcg.......................................................................   1     1     seq
...................cugaggucgagaaaacaagcgg......................................................................   2     0     seq
...................cugaggucgagaaaacaagcgU......................................................................   3     1     seq
....................ugaggucgagaaaacaagcg.......................................................................   2     0     seq
....................ugaggucgagaaaacaagUg.......................................................................   1     1     seq
....................ugaggGcgagaaaacaagcgg......................................................................   1     1     seq
....................ugaggucgagaaaaNaagcgg......................................................................   4     1     seq
....................ugaggucgagaaaacaagcgg......................................................................   1425  0     seq
....................ugaAgucgagaaaacaagcgg......................................................................   1     1     seq
....................ugaggucgagaaaaUaagcgg......................................................................   6     1     seq
....................Ngaggucgagaaaacaagcgg......................................................................   6     1     seq
....................ugaggucgagaaaacaagcgA......................................................................   1     1     seq
....................ugaggucgagaaCacaagcgg......................................................................   1     1     seq
....................ugaggCcgagaaaacaagcgg......................................................................   4     1     seq
....................ugaggucgagaaaaAaagcgg......................................................................   5     1     seq
....................ugaggucgagaaaacaagUgg......................................................................   67    1     seq
....................Cgaggucgagaaaacaagcgg......................................................................   1     1     seq
....................ugaggucgagaaaacaagcAg......................................................................   2     1     seq
....................ugaggucgagaaaaGaagcgg......................................................................   3     1     seq
....................ugagAucgagaaaacaagcgg......................................................................   1     1     seq
....................ugaggucgagaaGacaagcgg......................................................................   10    1     seq
....................ugagguUgagaaaacaagcgg......................................................................   3     1     seq
....................ugaggAcgagaaaacaagcgg......................................................................   2     1     seq
....................ugaggucgagaaaacaagUgga.....................................................................   1     1     seq
....................ugaggucgagaaaacaagcggU.....................................................................   6     1     seq
....................ugaggucgagaaaacaagcgga.....................................................................   11    0     seq
....................ugaggucgagaaaacaagcggag....................................................................   1     0     seq
....................ugaggucgagaaaacaagUggagu...................................................................   1     1     seq
....................ugaggucgagaaaacaagcggagugaag...............................................................   11    0     seq
....................ugaggucgagaaaacaagcggagugaagU..............................................................   1     1     seq
....................ugaggucgagaaaacaagcggagugaagg..............................................................   2     0     seq
....................ugaggucgagaaaacaagcggagugaagguu............................................................   1     0     seq
.....................gaggucgagaaaacaagcgg......................................................................   7     0     seq
.....................gaggucgagaaaacaagUgg......................................................................   1     1     seq
.....................gaggucgagaaaacaagNgga.....................................................................   1     1     seq
.....................gaggCcgagaaaacaagcgga.....................................................................   2     1     seq
.....................gaggucgagaaGacaagcgga.....................................................................   7     1     seq
.....................gaggucgagaaaacUagcgga.....................................................................   2     1     seq
.....................gaggucgagaaaaAaagcgga.....................................................................   1     1     seq
.....................Naggucgagaaaacaagcgga.....................................................................   2     1     seq
.....................gaggucgagaaaacaagcggU.....................................................................   1     1     seq
.....................gaggucgagaaaacaagcgga.....................................................................   601   0     seq
.....................gaggucgagaaaacaagUgga.....................................................................   66    1     seq
.....................gaggucgagaaaacCagcgga.....................................................................   4     1     seq
.....................gaggucgagaaaacaagcAga.....................................................................   3     1     seq
.....................Aaggucgagaaaacaagcgga.....................................................................   3     1     seq
.....................gaggucgagaaaacGagcgga.....................................................................   2     1     seq
.....................gaggucgagaaaacaagcggag....................................................................   2     0     seq
.....................gaggucgagaaaacaagcggaU....................................................................   1     1     seq
.....................gaggucgagaaaacaagcggagug..................................................................   1     0     seq
.....................Naggucgagaaaacaagcggagugaag...............................................................   1     1     seq
.....................gaggucgagaaaacaagcggagugaag...............................................................   1     0     seq
.....................Naggucgagaaaacaagcggagugaagg..............................................................   1     1     seq
.....................gaggucgagaaaacaagcggagugaagg..............................................................   46    0     seq
.....................gaggucgagaaaacaagcggagugaaggu.............................................................   2     0     seq
......................aggucgagaaaacaagcgga.....................................................................   2     0     seq
```

Mature   Star

```
cauucuuuggacauuccuccugaggucgagaaaacaagcggagugaagguuccauguucuaugucuuggauucggcuguuguacugccucuucuucuccuccucgugcuga
......................aggucgagaaaacaUgcggag....................................................................	1	1	seq
......................aggucgagaaaacaagcggag....................................................................	96	0	seq
......................aggucgagaaaacaagUggag....................................................................	11	1	seq
......................aggucgagaaaacaCgcggag....................................................................	1	1	seq
......................aggucgagaaGacaagcggag....................................................................	1	1	seq
......................aggucgagaaaacaUgcggagu...................................................................	1	1	seq
......................aggucgagaaaacaagcggagu...................................................................	14	0	seq
......................aggucgagaaaacaagcggagA...................................................................	1	1	seq
......................aggucgagaaaacaagcggagugaaggu.............................................................	2	0	seq
.......................ggucgagaaaacaagcggagu...................................................................	7	0	seq
.......................Ugucgagaaaacaagcggagu...................................................................	1	1	seq
.......................ggucgagaaaacaagcggaguU..................................................................	1	1	seq
........................gucgagaaaacaagcgAagug..................................................................	1	1	seq
........................gucgagaaaacaagGggagug..................................................................	1	1	seq
........................gucgagaaaacaagUggagug..................................................................	4	1	seq
........................gucgagaaaacaagcggagGg..................................................................	1	1	seq
........................gucgagaaaacaagcggagug..................................................................	125	0	seq
........................gucgagaaaacaagcggagugU.................................................................	11	1	seq
.........................ucgagaaaacaagcggagug..................................................................	1	0	seq
.........................ucgagaaaacaagcggaguga.................................................................	18	0	seq
.........................ucgagaaaacaagcAgaguga.................................................................	1	1	seq
..........................cgagaaaacaagcggagugaa................................................................	1	0	seq
...........................gagaaaacaagcggagugaag...............................................................	23	0	seq
...........................gagaaaacaagcggagugaaC...............................................................	5	1	seq
...........................gagaaaacaagcUgagugaag...............................................................	1	1	seq
............................agaaaacaagcggagugaaCg..............................................................	1	1	seq
............................agaaCacaagcggagugaagg..............................................................	1	1	seq
............................agaaaacaagcggagugaagg..............................................................	1	0	seq
.............................gaaaacaagcggagugaaggu.............................................................	1	0	seq
.............................gaaaacaagcggagugaaUgu.............................................................	1	1	seq
................................aacaagcggagugaaUguucc..........................................................	1	1	seq
.................................acaagcggagugaagguucca.........................................................	1	0	seq
..................................caagcggagugaagguuccau........................................................	1	0	seq
......................................cggagugaaCguuccauguuc....................................................	1	1	seq
......................................cggagugaagguuccauguuc....................................................	2	0	seq
.......................................ggagugaagguuccauguucu...................................................	3	0	seq
........................................gagugaagguuccauguucua..................................................	2	0	seq
..........................................gugaaAguuccauguucuaug................................................	3	1	seq
..........................................gugaaUguuccauguucuaug................................................	1	1	seq
..........................................gugaagguuccauguucuaug................................................	6	0	seq
...........................................ugaagguuccauguucuaugu...............................................	8	0	seq
............................................gaagguuccauguucuauguU..............................................	1	1	seq
............................................gaaUguuccauguucuauguc..............................................	1	1	seq
............................................gaagguuccauguucuauguc..............................................	16	0	seq
............................................Naagguuccauguucuauguc..............................................	1	1	seq
............................................gaaAguuccauguucuauguc..............................................	2	1	seq
.............................................aagguuccauguucuaugucu.............................................	5	0	seq
..............................................agguuccauguucuaugucu.............................................	1	0	seq
..............................................agguuccauguucuaugucuu............................................	6	0	seq
...............................................Aguuccauguucuaugucuug...........................................	3	1	seq
...............................................gguuccauguucuaugucuug...........................................	3	0	seq
...............................................Cguuccauguucuaugucuug...........................................	1	1	seq
................................................guuccauguucuaugucuugg..........................................	42	0	seq
................................................gCuccauguucuaugucuugg..........................................	1	1	seq
................................................guuccauguucuaugucuugU..........................................	1	1	seq
.................................................uuccauguucuaugucuugga.........................................	6	0	seq
.................................................uuccauguucuaugucuuggauucggc...................................	2	0	seq
..................................................uccauguucuaugucuuggauucggc...................................	15	0	seq
...................................................ccauguucuaugucuugUauu.......................................	1	1	seq
...................................................ccauguucuaugucuugAauu.......................................	1	1	seq
....................................................cauguucuaugucuugUauuc......................................	2	1	seq
.....................................................auguucuaugucuugUauucg.....................................	4	1	seq
.....................................................auguucuaugucuuggauucg.....................................	8	0	seq
.....................................................auguucuaugucuuNgauucg.....................................	1	1	seq
.....................................................auguucuaugucuuggauucgC....................................	1	1	seq
.....................................................auguucuaugucuuggauucggc...................................	1	0	seq
......................................................uguucuaugucuugAauucgg....................................	2	1	seq
......................................................uguucuaugucuugUauucgg....................................	6	1	seq
......................................................uguucuaugucuuggauucgg....................................	20	0	seq
......................................................uguucuaugucCuggauucggc...................................	1	1	seq
```

Mature **Star**

```
cauucuuuggacauuccuccugaggucgagaaaacaagcggagugaagguuccauguucuaugucuuggauucggcuguuguacugccucuucuucuccuccucgugcuga
......................................................Cguucuaugucuuggauucggc...................................  1  1  seq
......................................................uguucuaugucuuggauucggc...................................  36  0  seq
......................................................Nguucuaugucuuggauucggc...................................  1  1  seq
......................................................uguucuaugucuugUauucggc...................................  2  1  seq
......................................................uguucuaugucuuggauucggcugu................................  1  0  seq
......................................................uguucuaugucuuggauucggcuguug..............................  1  0  seq
......................................................uguucuaugucuuggauucggcuguugu.............................  1  0  seq
.......................................................guucuaugucuuggauucgg....................................  1  0  seq
.......................................................guucCaugucuuggauucggc...................................  1  1  seq
.......................................................Nuucuaugucuuggauucggc...................................  6  1  seq
.......................................................Auucuaugucuuggauucggc...................................  1  1  seq
.......................................................guucuaugucuuggauucggG...................................  1  1  seq
.......................................................guucuauguGuuggauucggc...................................  1  1  seq
.......................................................guucuaugucuuggauucggU...................................  7  1  seq
.......................................................guucuauguAuuggauucggc...................................  1  1  seq
.......................................................guucuaugucuuUgauucggc...................................  1  1  seq
.......................................................Uuucuaugucuuggauucggc...................................  2  1  seq
.......................................................guucuaugucuuggUuucggc...................................  2  1  seq
.......................................................guucuaugucuugUauucggc...................................  92  1  seq
.......................................................guucuaugucuugAauucggc...................................  24  1  seq
.......................................................guucuaugucuuggNuucggc...................................  3  1  seq
.......................................................guucuaugucuuggauucCgc...................................  1  1  seq
.......................................................guucuaugucuuggauucggc...................................  769  0  seq
.......................................................guucuaugucuuggCuucggc...................................  5  1  seq
.......................................................guucuaugucuCggauucggc...................................  1  1  seq
.......................................................guucuaugucuuggGuucggc...................................  3  1  seq
.......................................................guucuaugucuuggauucggcugu................................  2  0  seq
........................................................uucuaugucuuggauucggc...................................  5  0  seq
........................................................uucuaugucuugUauucggc...................................  1  1  seq
........................................................uucuaugucuugUauucggcu..................................  2  1  seq
........................................................uucuaugucuuggauucggcu..................................  16  0  seq
........................................................uucuaugucuugAauucggcu..................................  2  1  seq
.........................................................ucuaugucuuggauucggcug.................................  1  0  seq
.........................................................ucuaugucuuggauucggcugu................................  1  0  seq
..........................................................cuaugucuuggauucggcugu................................  17  0  seq
..........................................................cuaugucuugUauucggcugu................................  1  1  seq
..........................................................cuaugucuugAauucggcugu................................  2  1  seq
..........................................................cuaugucuuggauucggcuguu...............................  6  0  seq
..........................................................cuaugucuugUauucggcuguu...............................  1  1  seq
..........................................................cuaugucuuggauucggcuguuUu.............................  1  1  seq
...........................................................uaugucuuggauucggUuguu...............................  1  1  seq
...........................................................uaugucuuggauucgAcuguu...............................  1  1  seq
...........................................................uaugucuugAauucggcuguu...............................  2  1  seq
...........................................................uaugucuuggauucggcuguu...............................  26  0  seq
...........................................................uaugucuuggauucNgcuguu...............................  1  1  seq
...........................................................uaugucuugUauucggcuguu...............................  5  1  seq
...........................................................uaugucuuggauucAgcuguu...............................  1  1  seq
...........................................................uaugucuuggauucggcuguugua............................  1  0  seq
...........................................................uaugucuuggauucggcuguuguacugc........................  2  0  seq
...........................................................uaugucuuggauucggcuguuguacugU........................  1  1  seq
............................................................augucuugUauucggcuguug..............................  2  1  seq
............................................................augucuuggauucggcuguug..............................  19  0  seq
............................................................augucuugAauucggcuguug..............................  1  1  seq
............................................................augucuuggauucgAcuguug..............................  1  1  seq
............................................................augucuuggauucggcuguugu.............................  3  0  seq
.............................................................ugucuugUauucggcuguu...............................  1  1  seq
.............................................................ugucuuggauucggcuguugu.............................  11  0  seq
.............................................................ugucuugUauucggcuguugu.............................  2  1  seq
.............................................................ugucuuggauucggcuguuguacugccu......................  1  0  seq
..............................................................gucuugAauucggcuguugua............................  3  1  seq
..............................................................gucuuggauucggcuguugua............................  8  0  seq
..............................................................gucuugUauucggcuguugua............................  4  1  seq
...............................................................ucuuggauucggcuguugua............................  1  0  seq
...............................................................ucuuggauucggcugGuguac...........................  1  1  seq
...............................................................ucuugAauucggcuguuguac...........................  5  1  seq
...............................................................ucuugUauucggcuguuguac...........................  2  1  seq
...............................................................ucuuggauucggcuguuguac...........................  23  0  seq
...............................................................ucuuggauucggcuguuguacu..........................  1  0  seq
................................................................cuugUauucggcuguuguacu..........................  10  1  seq
................................................................cuuggauucggcugAuguacu..........................  2  1  seq
```

```
                    Mature                                                            Star
cauucuuuggacauuccuccugaggucgagaaaacaagcggagugaagguuccauguucuaugucuuggauucggcuguuguacugccucuucuucuccuccucgugcuga
................................................................Auuggauucggcuguuguacu..........................	2	1	seq
................................................................cuuggauucggcuguuguacu..........................	43	0	seq
.................................................................uuggauucggcuguuguac...........................	1	0	seq
.................................................................uuggauucggcuguuguacu..........................	2	0	seq
.................................................................uuggauucggcuguuguacuU.........................	5	1	seq
.................................................................uuggauucggcuguGguacug.........................	1	1	seq
.................................................................uugUauucggcuguuguacug.........................	2	1	seq
.................................................................uuggauucggcuguuguacug.........................	74	0	seq
.................................................................uugAauucggcuguuguacug.........................	1	1	seq
.................................................................uuggauucggcuguuguacugU........................	8	1	seq
.................................................................uuggauucggcuguuguacugc........................	1	0	seq
.................................................................uuggauucggcuguuguacugA........................	5	1	seq
..................................................................uggauucggcuguuguacug.........................	1	0	seq
..................................................................uggauucggcuAuuguacugc........................	1	1	seq
..................................................................uggauucggcuguuUuacugc........................	4	1	seq
..................................................................ugAauucggcuguuguacugc........................	30	1	seq
..................................................................uggauucggcuguuguacuUc........................	2	1	seq
..................................................................uggauucggAuguuguacugc........................	1	1	seq
..................................................................uggauucCgcuguuguacugc........................	1	1	seq
..................................................................uggauucggcuguuguacugc........................	540	0	seq
..................................................................uggauucggcuguuCuacugc........................	4	1	seq
..................................................................uggauucggcuguugAacugc........................	1	1	seq
..................................................................uggauucggcuguuguGcugc........................	1	1	seq
..................................................................Aggauucggcuguuguacugc........................	2	1	seq
..................................................................uggauucggcuguuguacugU........................	2	1	seq
..................................................................uggauucggcuguuguacuAc........................	1	1	seq
..................................................................uggauucggcuguuAuacugc........................	1	1	seq
..................................................................ugUauucggcuguuguacugc........................	70	1	seq
..................................................................uggauucggcuguuguacugcU.......................	1	1	seq
...................................................................ggauucggcuguuguacugc........................	5	0	seq
...................................................................ggauucggcuguuguacuUcc.......................	2	1	seq
...................................................................ggauucggcuguuguacugcc.......................	36	0	seq
...................................................................gUauucggcuguuguacugcc.......................	2	1	seq
...................................................................ggauucggcuguugAacugcc.......................	1	1	seq
...................................................................ggauucggcuguuguacugccu......................	3	0	seq
....................................................................gauucggcuguuguacCgccu......................	1	1	seq
....................................................................Nauucggcuguuguacugccu......................	1	1	seq
....................................................................gauucggcuguuguacugccu......................	20	0	seq
.....................................................................auucggcuguuguacugc........................	1	0	seq
.....................................................................Nuucggcuguuguacugccuc.....................	1	1	seq
.....................................................................auucggcuguuguacugccuc.....................	2	0	seq
......................................................................uucggcuguuguacugccucu....................	3	0	seq
.......................................................................ucggcuguuguacugccucuucuucuUcu...........	1	1	seq
.......................................................................ucggcuguuguacugccucuucuucuccucc.........	1	0	seq
........................................................................cgAcuguuguacugccucuuc..................	1	1	seq
........................................................................cggcuguuguacugccucuuc..................	1	0	seq
..........................................................................gcuguuguacugccucuucuu................	15	0	seq
..........................................................................gcuguuguacugccucuucuuU...............	2	1	seq
...........................................................................cuguuguacugccucuucuu................	1	0	seq
...........................................................................cuguuguacugccucuucuuc...............	27	0	seq
............................................................................uguuguacuUccucuucuucu..............	1	1	seq
............................................................................uguuguacugccucuucuucu..............	10	0	seq
............................................................................uguuguacugccucuucuucuU.............	1	1	seq
............................................................................uguuguacugccucuucuucuUcuccuc.......	1	1	seq
.............................................................................guuguacugccucuucuucuc.............	2	0	seq
.............................................................................guuguacuUccucuucuucuc.............	1	1	seq
..............................................................................uuguacugccucuucuucucc............	10	0	seq
...............................................................................uguacugccuUuucuucuccu...........	1	1	seq
...............................................................................uguacugccucuucuucuccu...........	6	0	seq
...............................................................................uguaUugccucuucuucuccu...........	1	1	seq
................................................................................guacugccucuucuucuccuc..........	7	0	seq
.................................................................................uacugccucuucuucuccucU.........	1	1	seq
.................................................................................uacugccucuucuucuccucc.........	14	0	seq
..................................................................................acugccucuucuucuccuccu........	3	0	seq
...................................................................................cugccucuucuucuccuccuc.......	2	0	seq
...................................................................................cugccucuucuucuccuccucUu.....	1	1	seq
....................................................................................ugccucuucuucucUuccucg......	1	1	seq
....................................................................................ugccucuucuucuccuccucg......	1	0	seq
.....................................................................................gccucuucuucuccuccucgu.....	2	0	seq
......................................................................................ccucuucuucuccuccucgug....	1	0	seq
```

Mature **Star**

```
cauucuuuggacauuccuccugaggucgagaaaacaagcggagugaagguuccauguucuaugucuuggauucggcuguuguacugccucuucuucuccuccucgugcuga
......................................................................................ccucuucuucuccuccucgugU...	1	1	seq
.......................................................................................cucuucuucuccuccucgugc...	2	0	seq
........................................................................................ucuucuucuccuccucgugcu..	8	0	seq
........................................................................................ucuucuucuccuccucgugcuU.	1	1	seq
.........................................................................................cuucuucuccuccucgugcug.	1	0	seq
..........................................................................................uucuucuccuccucgugcuga	5	0	seq
..........................................................................................uucuucuccuccucgugcugU	1	1	seq
```

Provisional ID	:	ML1353_28801
Score total	:	963.3
Score for star read(s)	:	3.9
Score for read counts	:	962.2
Score for mfe	:	-3.5
Score for randfold	:	-2.2
Score for cons. seed	:	3
Total read count	:	1899
Mature read count	:	1488
Loop read count	:	27
Star read count	:	384

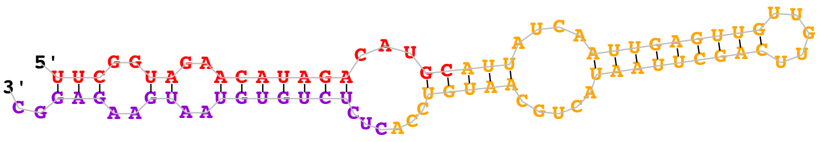

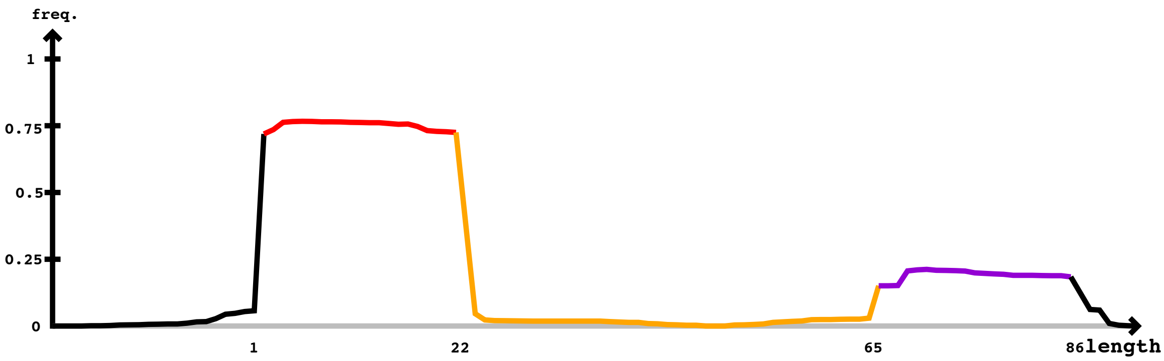

Mature **Star**

```
5'- ucacuaaacaacacacaauuuucgguagaacauagacaugcauuaucaauugaguuguuguucagcuuaauacugcaauguccacucucuguguaaugaagaggcuuugua  -3'   obs
    ucacuaaacaacacacaauuuucgguagaacauagacaugcauuaucaauugaguuguuguucagcuuaauacugcaauguccacucucuguguaaugaagaggcuuugua         exp
    ..............((((.((((..((..(((((((...(((((....(((((((((.....))))))))).....)))))......)))))))..))..))))..)))).   reads   mm   sample
    ..acuaaacaacacacaauuuuc........................................................................................   2       0    seq
    ....uaaacaacacacaauuuucgg......................................................................................   2       0    seq
    .....aaacaacacacaauAuucggu.....................................................................................   1       1    seq
    .....aaacaacacacaauuuucggu.....................................................................................   3       0    seq
    ......aacaacacacaauuuucggua....................................................................................   1       0    seq
    .......acaacacacaauuuucgguag...................................................................................   1       0    seq
    ........caacacacaauuuucgguaga..................................................................................   3       0    seq
    .........aacacacaauuuucgguagaa.................................................................................   1       0    seq
    ..........acacacaauuuucgguagaac................................................................................   2       0    seq
    ............acacaauuuucgguagaacaC..............................................................................   6       1    seq
    .............cacaauuuucgguagaacaua.............................................................................   2       0    seq
    .............cacaauuuucgguagaacaCa.............................................................................   4       1    seq
    .............cacaauuuucgguagaacaCagacaugc......................................................................   4       1    seq
    ..............acaauuuucgguagaacaCag............................................................................   2       1    seq
    ...............caauuuucgguagaacaCaga...........................................................................   19      1    seq
    ...............caauuuucgguagaacauaga...........................................................................   4       0    seq
    ................aauuuucgguagaacaCagac..........................................................................   24      1    seq
    ................aauuuucgguagaacauagac..........................................................................   10      0    seq
    .................auuuucgguagaacaCagaca.........................................................................   7       1    seq
    ..................uuuucgguagaacauagacaC........................................................................   5       1    seq
    ..................uuuucgguagaacauagacau........................................................................   3       0    seq
    ..................uuuucgguagaacaCagacau........................................................................   4       1    seq
    ..................uuuucgguagaacaCagacaugc......................................................................   2       1    seq
    ...................uuucgguagaacauagacaug.......................................................................   2       0    seq
    ...................uuucgguagaacauagacaCg.......................................................................   1       1    seq
    ...................uuucgguagaacaCagacaug.......................................................................   3       1    seq
    ....................Cucgguagaacauagacaugc......................................................................   1       1    seq
    ....................uuUgguagaacauagacaugc......................................................................   1       1    seq
    ....................uucggGagaacauagacaugc......................................................................   2       1    seq
    ....................uucgguagaacauaAacaugc......................................................................   1       1    seq
    ....................uucggCagaacauagacaugc......................................................................   2       1    seq
    ....................uucgguagaacaCagacaugc......................................................................   796     1    seq
    ....................uucgguagaacauagacaCgc......................................................................   18      1    seq
```

```
                    Mature                                                      Star
ucacuaaacaacacacaauuuucgguagaacauagacaugcauuaucaauugaguuguuguucagcuuaaauacugcaauguccacucucuguguaaugaagaggcuuugua
....................uucgguagaacauagacaugc.......................................................................  536  0  seq
....................Nucgguagaacauagacaugc.......................................................................  4    1  seq
....................uucgguagaacauagacaugU.......................................................................  1    1  seq
....................uucgguagaacauaNacaugc.......................................................................  1    1  seq
....................Aucgguagaacauagacaugc.......................................................................  4    1  seq
.....................ucgguagaacauagacaugc.......................................................................  1    0  seq
.....................ucgguagaacaCagacaugc.......................................................................  2    1  seq
.....................ucgguagaacaCagacaugca......................................................................  25   1  seq
.....................ucgguagaacauagacaugca......................................................................  6    0  seq
......................cgguagaacaCagacaug........................................................................  1    1  seq
......................cgguagaacauagacaCgcau.....................................................................  1    1  seq
......................Ngguagaacauagacaugcau.....................................................................  1    1  seq
......................cgguagaacaCagacaugcau.....................................................................  21   1  seq
......................cgguagaacauagacaugcau.....................................................................  28   0  seq
......................cgguagaacauagacaugcauu....................................................................  1    0  seq
......................cgguagaacauagacaugcauuU...................................................................  3    1  seq
.......................gguagaacaCagacaugc.......................................................................  1    1  seq
.......................gguagaacauagacaCgc.......................................................................  1    1  seq
.......................gguagaacauagacaugca......................................................................  1    0  seq
.......................gguagaacaCagacaugcauu....................................................................  5    1  seq
........................guagaacaCagacaugcauua...................................................................  2    1  seq
.........................uagaacaCagacaugcauuau..................................................................  1    1  seq
..........................agaacaCagacaugcauuauc.................................................................  1    1  seq
...........................gaacauagacaugcauuauca................................................................  1    0  seq
...................................acaugcauuaucaauugaguu........................................................  4    0  seq
....................................caugcauuaucaauugaguug.......................................................  3    0  seq
....................................caugcauuaucaauugaguugu......................................................  1    0  seq
.....................................augcauuaucaauugaguugu......................................................  2    0  seq
......................................ugcauuaucaauugaguuguuguucagc..............................................  1    0  seq
.......................................gcauuaucaauugaUuuguug....................................................  1    1  seq
.......................................gcauuaucaauugaguuguug....................................................  8    0  seq
........................................cauuaucaauugaguuguugu...................................................  1    0  seq
........................................cauuaucaauugagAuguugu...................................................  1    1  seq
.........................................auuaucaauugaguuguuguu..................................................  6    0  seq
.........................................auuaucaauugaguuguuguuU.................................................  1    1  seq
..........................................uuaucaauugaguuguuguuc.................................................  1    0  seq
...........................................uaucaauugaguuguuguuca................................................  3    0  seq
............................................aucaauugaguuguuguucagc..............................................  1    0  seq
.............................................ucaauugaguuguuguucagc..............................................  4    0  seq
......................................................................auacugcaauguccacucucu.....................  8    0  seq
.......................................................................uacugcaauguccacucucug....................  1    0  seq
........................................................................acugcaauguccacucucugu...................  2    0  seq
........................................................................acugcaauguccacucucuguUu.................  1    1  seq
.........................................................................cugcaauguccacucucugug..................  3    0  seq
.........................................................................cugcaauguccacucucugugu.................  1    0  seq
..........................................................................ugcaaugCccacucucugugu.................  1    1  seq
..........................................................................ugcaauguccacucucugugu.................  11   0  seq
...........................................................................gcaauguccacucucugugua................  4    0  seq
............................................................................caauguccacucucuguguaa...............  4    0  seq
.............................................................................aauguccacucucuguguaau..............  2    0  seq
.............................................................................aauguccacucucuAuguaau..............  1    1  seq
..............................................................................auguccacucucuguguaaug.............  8    0  seq
..............................................................................auguccacucucuguguaaugaagagg.......  1    0  seq
..............................................................................auguccacucucuguguaaugaagaggc......  1    0  seq
...............................................................................uguccacucucuguguaaugaagaggc......  1    0  seq
.................................................................................uccacucucuguguaaugaag..........  2    0  seq
..................................................................................ccacucucuguguaaugaaga.........  1    0  seq
....................................................................................acucucuguguaaugaagagg.......  1    0  seq
....................................................................................acuUucuguguaaugaagagg.......  7    1  seq
.....................................................................................cucuAuguguaaugaagaggc......  1    1  seq
.....................................................................................cuUucuguguaaugaagaggc......  11   1  seq
.....................................................................................Nucucuguguaaugaagaggc......  3    1  seq
.....................................................................................cucucuguguaaugaagaggc......  231  0  seq
.....................................................................................Uucucuguguaaugaagaggc......  1    1  seq
.....................................................................................cucucuguAuaaugaagaggc......  1    1  seq
.....................................................................................Aucucuguguaaugaagaggc......  1    1  seq
.....................................................................................cucucuguguaaugCagaggc......  1    1  seq
.......................................................................................cucuguguaaugaagaggcuu....  2    0  seq
........................................................................................ucuguguaaugaagaggcuu....  3    0  seq
........................................................................................Gcuguguaaugaagaggcuuu...  1    1  seq
```

Mature **Star**

```
ucacuaaacaacacacaauuuucgguagaacauagacaugcauuaucaauugaguuguuguucagcuuaaauacugcaaugucca cucucuguguaaugaagaggcuuugua
........................................................................................ucuguguaaugaagaggcuuu...   102   0   seq
........................................................................................ucuguguaaugaagaggcuuuU..   7     1   seq
.........................................................................................cuguguaaugaagaggcuuug..   7     0   seq
.........................................................................................cuguguaaugaagaggcuuuguU   1     1   seq
..........................................................................................uguguaaugaagaggcuuugu.   4     0   seq
...........................................................................................guguaaugaagaggcuuugua   1     0   seq
```

Provisional ID	:	ML1169_26070
Score total	:	934.2
Score for star read(s)	:	3.9
Score for read counts	:	936.7
Score for mfe	:	-3.5
Score for randfold	:	-2.2
Score for cons. seed	:	-0.6
Total read count	:	1849
Mature read count	:	1847
Loop read count	:	0
Star read count	:	2

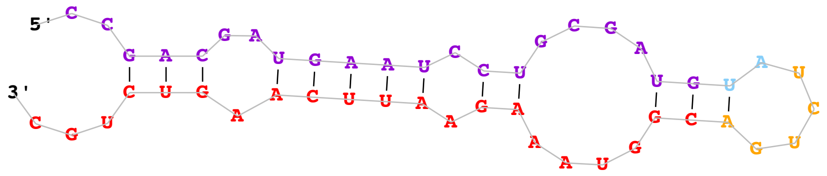

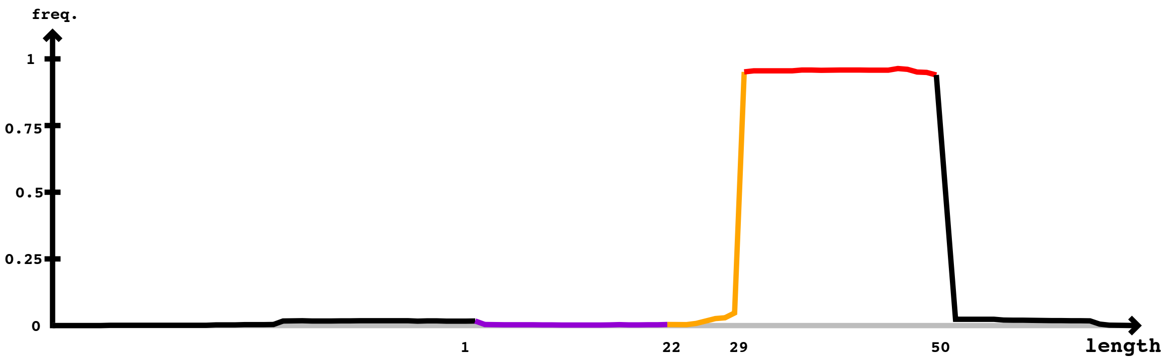

Star Mature

```
5'- uguuuguuuuaguugagacagagcuacagaguuuucaguuugccgacgaugaauccugcgaugauaucugacgguaaagaauucaagucugcaauaucgagguaggaguuuu  -3'  obs
    uguuuguuuuaguugagacagagcuacagaguuuucaguuugccgacgaugaauccugcgaugauaucugacgguaaagaauucaagucugcaauaucgagguaggaguuuu       exp
    ..((((((((....)))))))).((((.((.........((((.(((..(((((.((....(((.....)))....)).))))).))).))))..))...)))).......  reads  mm  sample
    ....uguuuuaguugagacagagcu......................................................................................  2  0  seq
    ...............agacagagcuacagaguuuuc...........................................................................  3  0  seq
    ..................cGgagcuacagaguuuucagu........................................................................  1  1  seq
    ..................cagagcuacagaguuuucagu........................................................................  1  0  seq
    .....................agcuacagaguuuucaguuug.....................................................................  1  0  seq
    ......................gcuacagaguuuucaguuugc....................................................................  26  0  seq
    .......................cuacagaguuuucaguuugcc...................................................................  1  0  seq
    ........................uacagaguuuucaguuugccg..................................................................  1  0  seq
    ............................gaguuuucaguuugccgacga..............................................................  1  0  seq
    ..............................guuuucaguuugccgacgaug............................................................  1  0  seq
    .....................................guuugccgacgaugaauccGg.....................................................  2  1  seq
    ..........................................ccgacgaugaauccugcgaug................................................  2  0  seq
    ........................................................Cgcgauguaucugacgguaaa..................................  1  1  seq
    .........................................................gcgauguaucugacgguaaag.................................  2  0  seq
    ............................................................auguaucugacgguaaagaau..............................  1  0  seq
    ..............................................................guaucugacgguaaagaauuc............................  2  0  seq
    ................................................................aucugacgguaaagaauucaa..........................  1  0  seq
    .................................................................ucugacgguaaagaauucaag.........................  9  0  seq
    ..................................................................cugacgguaaagaauucaagC........................  12  1  seq
    ..................................................................cugacgguaaagaauucaagu........................  5  0  seq
    ..................................................................cugacgguaaagaauucaaguU.......................  1  1  seq
    ...................................................................ugacgguaaagaauucaaguc.......................  15  0  seq
    ...................................................................ugacgguaaagaauucaagCc.......................  3  1  seq
    ....................................................................gacgguaaagaauucaaguc.......................  2  0  seq
    ....................................................................gacgguaaagaauucaagucu......................  4  0  seq
    .....................................................................acgguaaagaauucaagucug.....................  11  0  seq
    .....................................................................acgguaaagaauucGagucug.....................  1  1  seq
    .....................................................................acgguaaagaauucNagucug.....................  1  1  seq
    .....................................................................acgguUaagaauucaagucug.....................  3  1  seq
    .....................................................................acgguaaagaauucaagucugU....................  5  1  seq
    .....................................................................acgguaaagaauucaagucugc....................  15  0  seq
    ......................................................................cgguaaagaauucaaguc.......................  1  0  seq
    ......................................................................cgguaaagaauucaagucug.....................  3  0  seq
```

Star **Mature**

```
uguuuguuuuaguugagacagagcuacagaguuuucaguuugccgacgaugaauccugcgauguaucugacgguaaagaauucaagucugcaauaucgagguaggaguuuu
......................................................................cgguaaagaauucaagGcugc....................   1      1   seq
......................................................................cAguaaagaauucaagucugc....................   1      1   seq
......................................................................cgguaaagaauucaagucugc....................   1671   0   seq
......................................................................cgguaaagaauucaagucugU....................   5      1   seq
......................................................................cgguGaagaauucaagucugc....................   1      1   seq
......................................................................cgguUaagaauucaagucugc....................   64     1   seq
......................................................................cgguaaagaauucaagucuCc....................   1      1   seq
......................................................................cgAuaaagaauucaagucugc....................   1      1   seq
......................................................................Ngguaaagaauucaagucugc....................   9      1   seq
......................................................................cgguaaagaauucaUgucugc....................   6      1   seq
......................................................................cgguaaagaauucaCgucugc....................   4      1   seq
......................................................................cgguaaagaCuucaagucugc....................   1      1   seq
......................................................................cgguaaagaauucaGgucugc....................   2      1   seq
......................................................................cggCaaagaauucaagucugc....................   1      1   seq
......................................................................cgguaaagaauucaagucNgc....................   2      1   seq
......................................................................cgguaaagaauucaNgucugc....................   4      1   seq
......................................................................Ugguaaagaauucaagucugc....................   1      1   seq
......................................................................Agguaaagaauucaagucugc....................   2      1   seq
......................................................................cggGaaagaauucaagucugc....................   1      1   seq
......................................................................Ggguaaagaauucaagucugc....................   1      1   seq
......................................................................cgguaaagaauucaaguUugc....................   3      1   seq
......................................................................cggAaaagaauucaagucugc....................   1      1   seq
......................................................................cgguaaagaauucaagCcugc....................   10     1   seq
.......................................................................gguaaagaauucaagucugc....................   7      0   seq
.......................................................................gguaaagaauucaagucugca...................   1      0   seq
............................................................................agaauucaagCcugcaauauc..............   2      1   seq
............................................................................agaauucaagucugcaauauc..............   4      0   seq
.............................................................................gaauucaagucugcaauaucg.............   1      0   seq
...............................................................................auucaagucugcaauaucgag...........   1      0   seq
................................................................................uucaagucugcaauaucgagg..........   1      0   seq
.................................................................................ucaagucugcaauaucgaggu.........   1      0   seq
...................................................................................aagucugcaauaucgagguag.......   1      0   seq
.....................................................................................gucugcaauaucgagguagga.....   1      0   seq
......................................................................................ucugcaauaucgaggAaggag....   1      1   seq
......................................................................................ucugcaauaucgagguaggag....   20     0   seq
.......................................................................................cugcaauaucgagguaggag....   3      0   seq
.......................................................................................cugcaauaucgagguaggagu...   8      0   seq
........................................................................................ugcaauaucgagguaggaguu..   1      0   seq
........................................................................................ugcaauaucgagguaggaguuuu   1      0   seq
.........................................................................................gcaUuaucgagguaggaguuu.   1      1   seq
```

Provisional ID	:	ML4839_48010
Score total	:	742.1
Score for star read(s)	:	3.9
Score for read counts	:	731.7
Score for mfe	:	1.9
Score for randfold	:	1.6
Score for cons. seed	:	3
Total read count	:	1447
Mature read count	:	1414
Loop read count	:	0
Star read count	:	33

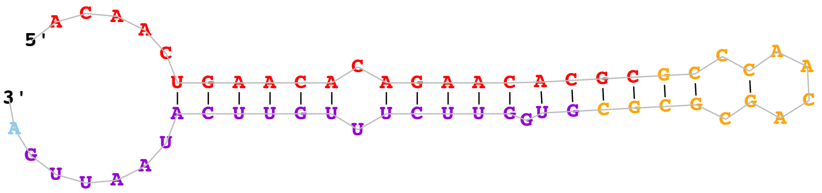

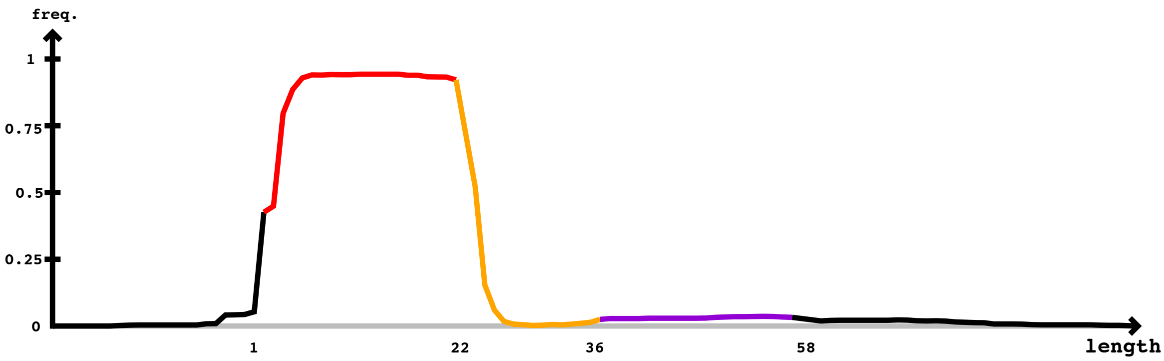

Mature **Star**

```
5'- cggcauuuuggcgccauaacacaacugaacacagaacacgcgcccaacagcgcgcgugguucuuuguucauaauugauauuuagcgccgaagguccugcacaaaagguguc   -3'    obs
    cggcauuuuggcgccauaacacaacugaacacagaacacgcgcccaacagcgcgcgugguucuuuguucauaauugauauuuagcgccgaagguccugcacaaaagguguc          exp
    .(((.((((((((.(((.((....((((((.((((((((((.(....).)))))).))))).))))))....)).)))...))))))))))))..((((.....)))).   reads   mm   sample
    .....uuuuggcgccauaacacaacu.....................................................................................   2       0    seq
    .....uuuuggcgccauaaNacaacu.....................................................................................   1       1    seq
    ......uuuggcgccauaacacaacug....................................................................................   1       0    seq
    ......uuuggcgccauaacacaacugaacacagaac..........................................................................   1       0    seq
    .......uuggcgccauaacacaacuga...................................................................................   1       0    seq
    ..............cauaacacaacugaacacaga............................................................................   6       0    seq
    ..............cauaacacaacugaacacagaacacgcg.....................................................................   1       0    seq
    ...............auaacacaacugaacacagaacacgcgU....................................................................   1       1    seq
    ................uaacacaacugaacCcagaac..........................................................................   1       1    seq
    ................uaacacaacugaacacagaac..........................................................................   7       0    seq
    ................uaacacaacugaacacagaacacgc......................................................................   2       0    seq
    ................uaGcacaacugaacacagaacacgc......................................................................   1       1    seq
    ................uaacacaacugaacacagaacacgcg.....................................................................   4       0    seq
    ................uUacacaacugaacacagaacacgcg.....................................................................   1       1    seq
    ................uaacacaacugaacacagaacacgcgc....................................................................   26      0    seq
    ................uaacacaacugaacacagaacacgcgcc...................................................................   8       0    seq
    ................uaacacaacugaacacagaacacgcgcccaa................................................................   1       0    seq
    .................aacacaacugaacacagaaca.........................................................................   1       0    seq
    ..................acacaacugaacacagaacac........................................................................   1       0    seq
    ..................acacaacugaacacagaacacgcgc....................................................................   1       0    seq
    ...................cacaacugaacacagaacacg.......................................................................   13      0    seq
    ...................cacaacugaacaGagaacacg.......................................................................   1       1    seq
    ...................cacaacugaacacagaacacgU......................................................................   1       1    seq
    ...................cacaacugaacacagaacacgcgc....................................................................   1       0    seq
    ....................acaacugaacacagaacacg.......................................................................   2       0    seq
    ....................acaacugaacaUagaacacgc......................................................................   23      1    seq
    ....................acCacugaacacagaacacgc......................................................................   2       1    seq
    ....................acaacugaCcacagaacacgc......................................................................   1       1    seq
    ....................acaacugaacacagaaAacgc......................................................................   3       1    seq
    ....................acaacugaacacagaacGcgc......................................................................   1       1    seq
    ....................acaacugaacacagGacacgc......................................................................   1       1    seq
    ....................acaacugaacacagUacacgc......................................................................   4       1    seq
    ....................acaacugaacacagaacacgU......................................................................   4       1    seq
```

```
                    Mature                          Star
cggcauuuuggcgccauaacacaacugaacacagaacacgcgcccaacagcgcgcgugguucuuuguucauaauugauauuuagcgccgaagguccugcacaaaagguguc
....................Ucaacugaacacagaacacgc......................................................................    2      1     seq
....................acaacugaacacagaacacAc......................................................................    1      1     seq
....................acaaGugaacacagaacacgc......................................................................    1      1     seq
....................acaacugaacacagaacacgc......................................................................    526    0     seq
....................acaacugaacacGgaacacgc......................................................................    1      1     seq
....................Gcaacugaacacagaacacgc......................................................................    1      1     seq
....................acaacugaacaGagaacacgc......................................................................    7      1     seq
....................Ncaacugaacacagaacacgc......................................................................    4      1     seq
....................acaacugaacacagCacacgc......................................................................    1      1     seq
....................acaacugaacacCgaacacgc......................................................................    2      1     seq
.....................caacugaacacagaacacgc......................................................................    3      0     seq
.....................caacugaacacagaacacgcg.....................................................................    27     0     seq
.....................caacugaacacagaCcacgcg.....................................................................    1      1     seq
.....................caacugaacacagaacacgcgc....................................................................    2      0     seq
.....................caacugaacacagaacacgcgU....................................................................    3      1     seq
......................aacugaacacagaacacgc......................................................................    5      0     seq
......................aacugaacacagaaAacgc......................................................................    1      1     seq
......................Nacugaacacagaacacgcgc....................................................................    4      1     seq
......................aacugaacaGagaacacgcgc....................................................................    8      1     seq
......................aacugaacaUagaacacgcgc....................................................................    5      1     seq
......................aacugaacacaAaacacgcgc....................................................................    1      1     seq
......................aacugaacacagaacacgcgU....................................................................    3      1     seq
......................aacugaacacagaaAacgcgc....................................................................    4      1     seq
......................aaGugaacacagaacacgcgc....................................................................    1      1     seq
......................aacugaacacagaacacgcgc....................................................................    505    0     seq
......................aacugaacacagaaGacgcgc....................................................................    1      1     seq
......................aacugaacacagaaNacgcgc....................................................................    1      1     seq
......................aacugaacacagaacacgcCc....................................................................    1      1     seq
......................Cacugaacacagaacacgcgc....................................................................    4      1     seq
......................aacugaacacagaaUacgcgc....................................................................    3      1     seq
.......................acugaacacagaacacgcgc....................................................................    3      0     seq
.......................acugaacacagaacacgcgcc...................................................................    134    0     seq
.......................acugaacacagaacNcgcgcc...................................................................    1      1     seq
.......................acugaacaUagaacacgcgcc...................................................................    1      1     seq
.......................acugaacacagaacGcgcgcc...................................................................    1      1     seq
.......................acugaacaGagaacacgcgcc...................................................................    1      1     seq
........................cugaacacagaacacgcgcc...................................................................    2      0     seq
........................cugaacacagaacacgcgccc..................................................................    62     0     seq
........................cugaacacagaacacgcgNcc..................................................................    1      1     seq
........................cugaacacagaaAacgcgccc..................................................................    1      1     seq
........................Augaacacagaacacgcgccc..................................................................    1      1     seq
........................cugaacacagaacacgAgccc..................................................................    1      1     seq
.........................ugaacacagaacacgcgccc..................................................................    1      0     seq
.........................ugaacacagaacacgcgccca.................................................................    14     0     seq
.........................ugaacacagaaAacgcgccca.................................................................    2      1     seq
.........................ugaacacagaacacgcgcccaU................................................................    1      1     seq
..........................gaacacagaacacgcgcccaa................................................................    1      0     seq
..........................gaacacagaacacgcgcccaaG...............................................................    1      1     seq
...........................aacacagaacacgcgcccaac...............................................................    4      0     seq
..............................acagaacacgcgcccaacagc............................................................    3      0     seq
.................................................gcgcgcgugguucuuuguuca.........................................    2      0     seq
..................................................cgUgcgugguucuuuguucau........................................    1      1     seq
..................................................cgcgcgugguucuuuguucau........................................    3      0     seq
...................................................gcgcgugguucuuuguucaua.......................................    1      0     seq
....................................................cgcgugguucuuuguucauaa......................................    2      0     seq
....................................................cgcgugguucuuuguucauaaA.....................................    1      1     seq
....................................................cgcgugguucuuuguucauaauu....................................    1      0     seq
.....................................................gcgugguucuuuguucauaa......................................    1      0     seq
.....................................................gcgugguucuuuguucauaau.....................................    3      0     seq
.....................................................gcgugguucuuuguucauaauu....................................    1      0     seq
......................................................cgugguucuuuguucauaauu....................................    6      0     seq
.......................................................gugguucuuuguucauaauug...................................    16     0     seq
.......................................................gugguucuuuguucaCaauug...................................    1      1     seq
........................................................ugguucuuuguucauaauuga..................................    5      0     seq
............................................................ucuuuguucauaauugauaAuuagcgc........................    2      1     seq
..................................................................uucauaauugauaAuuagcgccgaaggucc...............    1      1     seq
...................................................................ucauaauugauaAuuagcgcc.......................    1      1     seq
...................................................................ucauaauugauauuuagcgcc.......................    3      0     seq
....................................................................cauaauugauauuuagcgccg......................    2      0     seq
.....................................................................auaauugauauuuagcgccga.....................    2      0     seq
```

Mature Star

```
cggcauuuuggcgccauaacacaacugaacacagaacacgcgcccaacagcgcgcgugguucuuuguucauaauugauauuuagcgccgaagguccugcacaaaagguguc
......................................................................uaauugauauuuagcgccgaa....................	2	0	seq
.......................................................................aauugauauuuagcgccgaag...................	4	0	seq
.......................................................................aauugauaAuuagcgccgaag...................	1	1	seq
........................................................................auugauauuuagcgccgaagg..................	2	0	seq
.........................................................................uugauauuuagcgccgaaggu.................	1	0	seq
.........................................................................uugauaAuuagcgccgaaggu.................	1	1	seq
..........................................................................ugauauuuagcgccgaagguc................	1	0	seq
...........................................................................gauauuuagcgccgaaggucc...............	3	0	seq
...........................................................................gauaAuuagcgccgaaggucc...............	3	1	seq
..............................................................................auuuagcgccgaagguccugc............	1	0	seq
...............................................................................uuuagcgccgaagguccugca...........	3	0	seq
................................................................................uuagcgccgaagguccugcac..........	1	0	seq
......................................................................................ccgaagguccugcacaaaagg....	2	0	seq
.......................................................................................cgaagguccugcacaaaaggu...	1	0	seq
.........................................................................................aagguccugcacaaaaggugu.	1	0	seq
..........................................................................................agguccugcacaaaaggugu.	1	0	seq
..........................................................................................agguccugcacaaaagguguc	2	0	seq
```

Provisional ID	:	ML3693_44531
Score total	:	400.7
Score for star read(s)	:	3.9
Score for read counts	:	396.8
Score for mfe	:	-0.7
Score for randfold	:	-2.2
Score for cons. seed	:	3
Total read count	:	790
Mature read count	:	771
Loop read count	:	0
Star read count	:	19

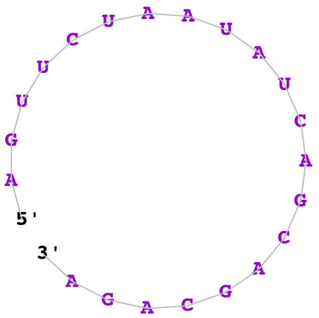

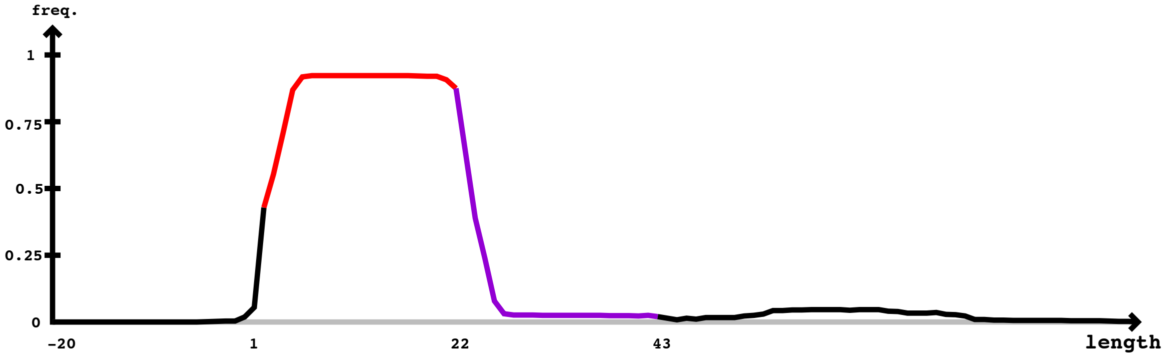

Mature Star

```
5'- guuguagugugguuuagauguaauggagauauguagaaagcaguucuaaauaucagcagcagagucacacaacacuuacagcacgggcuaccuggucugagagggagagacg  -3'  obs
    guuguagugugguuuagauguaauggagauauguagaaagcaguucuaaauaucagcagcagagucacacaacacuuacagcacgggcuaccuggucugagagggagagacg        exp
    (((...(((((..((...(((..((..(((((.(((((.....))))))))))..)))))))..)))))....(((.(((..((((...))))..))).))).....))).   reads   mm   sample
    ..............uaAauguaauggagauaugua.............................................................................   1   1   seq
    ...............aAauguaauggagauauguag............................................................................   1   1   seq
    ................Aauguaauggagauauguaga...........................................................................   1   1   seq
    ..................uguaauggagauauguagaa..........................................................................   1   0   seq
    ..................uguaauggagauauguagaaa.........................................................................   10   0   seq
    ..................uguaauggagauauguagaaag........................................................................   1   0   seq
    ..................uguaauggagauauguagaaagcaguu...................................................................   1   0   seq
    ...................guaauggagauauguagaaag........................................................................   27   0   seq
    ...................guaauggagauaugCagaaag........................................................................   1   1   seq
    ...................Nuaauggagauauguagaaag........................................................................   1   1   seq
    ...................guaauggagauaugAagaaag........................................................................   1   1   seq
    ....................uaauggagauauguagaaa.........................................................................   1   0   seq
    ....................uaauggagauauguNgaaagc.......................................................................   1   1   seq
    ....................Caauggagauauguagaaagc.......................................................................   1   1   seq
    ....................Aaauggagauauguagaaagc.......................................................................   2   1   seq
    ....................uaaugUagauauguagaaagc.......................................................................   1   1   seq
    ....................uaaAggagauauguagaaagc.......................................................................   1   1   seq
    ....................uaauAgagauauguagaaagc.......................................................................   1   1   seq
    ....................Gaauggagauauguagaaagc.......................................................................   1   1   seq
    ....................uaauggagCuauguagaaagc.......................................................................   1   1   seq
    ....................uaauggagauauguCgaaagc.......................................................................   2   1   seq
    ....................uaauggagauaAguagaaagc.......................................................................   1   1   seq
    ....................uaauggagauauguGgaaagc.......................................................................   1   1   seq
    ....................uaauggagauauguagaaagc.......................................................................   299   0   seq
    ....................uaauggagauauguagaaagcaguucuaU...............................................................   1   1   seq
    .....................aauggagauauguagaaagc.......................................................................   3   0   seq
    .....................aauggagauauguagaaagca......................................................................   98   0   seq
    .....................Nauggagauauguagaaagca......................................................................   1   1   seq
    .....................aauggagauauguaNaaagca......................................................................   1   1   seq
    .....................aauggagauaugGagaaagca......................................................................   1   1   seq
    .....................aauggagauauguagaaagcU......................................................................   1   1   seq
    ......................auggagauauguagaaagc.......................................................................   1   0   seq
    ......................auggagauauguagaaagcag.....................................................................   121   0   seq
```

Mature **Star**

```
guuguagugugguuuagauguaauggagauauguagaaagcaguucuaaauaucagcagcagagucacacaacacuuacagcacgggcuaccuggucugagagggagagacg
......................auggagauauguagCaagcag.....................................................................	1	1	seq
.....................Uuggagauauguagaaagcag......................................................................	1	1	seq
......................auggagauauguagaaagcaC.....................................................................	1	1	seq
......................auggagauauguagaaGgcag.....................................................................	1	1	seq
......................auggagauauguagaaagcagu....................................................................	4	0	seq
.......................uggagauauguagaaagcag.....................................................................	1	0	seq
.......................uggagauauguagaCagcagu....................................................................	1	1	seq
.......................uggagauauguagaaagcagu....................................................................	130	0	seq
.......................ugCagauauguagaaagcagu....................................................................	1	1	seq
.......................uggagauaugCagaaagcagu....................................................................	1	1	seq
.......................Gggagauauguagaaagcagu....................................................................	1	1	seq
........................ggagauauguagaaagcagu....................................................................	2	0	seq
........................ggagauauguagaaagcaguu...................................................................	39	0	seq
.........................gagauauguagaaagcaguuc..................................................................	4	0	seq
...................................gaaagcaguucuaaauaucagc.......................................................	1	0	seq
......................................agcaguucuaaauaucagcagc....................................................	1	0	seq
........................................caguucuaauUucagcagcag...................................................	1	1	seq
........................................caguucuaaauaucagcagcag..................................................	3	0	seq
.........................................aguucuaaauaucagGagcaga.................................................	1	1	seq
.........................................aguucuaaauaucagcagcaga.................................................	5	0	seq
..........................................guucuaaauaucagcagcagag................................................	4	0	seq
............................................ucuaaauaucagcagcagaguc..............................................	5	0	seq
.............................................................gagucacacaacacuuacagc..............................	2	0	seq
.................................................................cacacaacacuuacagcacgg..........................	5	0	seq
..................................................................acacaacacuuacagcacggg.........................	1	0	seq
..................................................................acacaacacuuacagcacgggUu.......................	1	1	seq
...................................................................cacaacacuuacagcacgggc........................	5	0	seq
.......................................................................acacuuacagcacgggcuacc....................	5	0	seq
........................................................................cacuuacagcacgggcuacc....................	1	0	seq
........................................................................cacuuacagcacgggcuaccu...................	1	0	seq
.........................................................................acuuacagcacgggcuaccug..................	4	0	seq
..........................................................................cuuacagcacgggcuaccugg.................	11	0	seq
............................................................................uacagcacgggcuaccugguc...............	2	0	seq
..............................................................................cagcacgggcuaccuggucug.............	1	0	seq
...................................................................................cgggcuaccuggucugagaggU.......	1	1	seq
...................................................................................cgggcuaccuggucugagagggagagacg	1	0	seq
.........................................................................................accuggucugagagNgagaga..	1	1	seq
...........................................................................................cuggucugagagggagag...	1	0	seq
...........................................................................................cuggucugagagggagagacg	1	0	seq
```

Provisional ID	:	ML1242_26766
Score total	:	368
Score for star read(s)	:	3.9
Score for read counts	:	358
Score for mfe	:	1.4
Score for randfold	:	1.6
Score for cons. seed	:	3
Total read count	:	714
Mature read count	:	678
Loop read count	:	35
Star read count	:	1

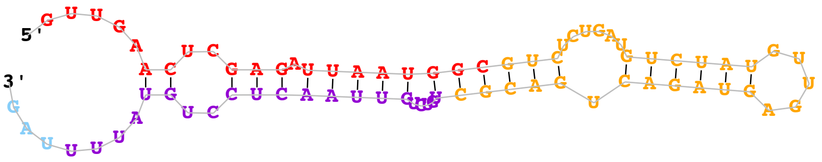

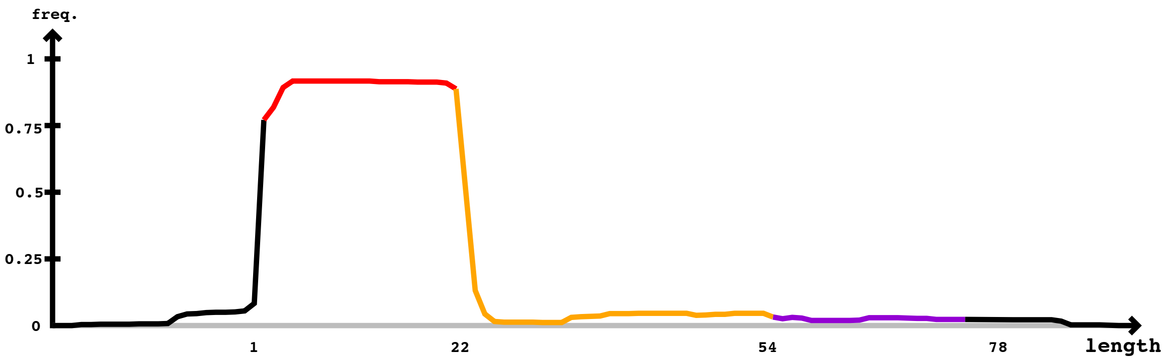

Mature **Star**

```
5'- acugccuugaguucucgucuguugaacucgagauuaaauggcgucucugaugucuauguugaguagacugacgcugucuguuaacuccuguauuuuagaagacucagggucg  -3'   obs
    acugccuugaguucucgucuguugaacucgagauuaaauggcgucucugaugucuauguugaguagacugacgcugucuguuaacuccuguauuuuagaagacucagggucg         exp
    ...((((((((((....((((..(.((..(((.(((((((((((......((((((.....)))))).))))))....))))))))..)).)..)))).))))))))))..   reads   mm   sample
    .cugccuugaguucuUgucugu..........................................................................................   1   1   seq
    .cugccuugaguucucCucugu..........................................................................................   1   1   seq
    .cugccuugaguucucgucugu..........................................................................................   1   0   seq
    ...gccuugaguucucgucuguug........................................................................................   1   0   seq
    .......ugaguucucgucuguugaacu....................................................................................   1   0   seq
    ..........guucucgucuguugaacucgagauuaaauggc......................................................................   1   0   seq
    ...........uucucgucuguugaacucgag................................................................................   2   0   seq
    ...........uucucgucuguugaacucgagauuaaauggc......................................................................   17  0   seq
    ...........uucucgucuguugaacucgagauuaaauggcg.....................................................................   1   0   seq
    ............ucucgucuguugaacucgagauuaaauggc......................................................................   6   0   seq
    ............ucucgucuguugaacucgagauuaaauggcguc...................................................................   2   0   seq
    .............cucgucuguugaacucgagauuaaauggcgu....................................................................   1   0   seq
    ..............ucgucuguugaacucAagauuaaauggc......................................................................   1   1   seq
    ..............ucgucuguugaacucgagauuaaauggcg.....................................................................   2   0   seq
    ...............cgucuguugaacucgagauua............................................................................   1   0   seq
    .................ucuguugaacucAagauuaaauggcguc...................................................................   1   1   seq
    ..................cuguugaacucgagauuaaaug........................................................................   3   0   seq
    ...................uguugaacucgagaAuaaaugg.......................................................................   1   1   seq
    ...................uguugaacucgagauAaaaugg.......................................................................   1   1   seq
    ...................uguugaacucgagauuaaaugg.......................................................................   15  0   seq
    ...................uguugaacucgagauuaaauggc......................................................................   1   0   seq
    ...................uguugaacucgagauuaaauggcgu....................................................................   1   0   seq
    ...................uguugaacucgagauuaaauggcguc...................................................................   2   0   seq
    ...................uguugaacucAagauuaaauggcguc...................................................................   1   1   seq
    ....................guugaacucgagauuaaauggc......................................................................   515 0   seq
    ....................guugaacucgagauuaaauggU......................................................................   2   1   seq
    ....................guugaacucgagauGaauggc......................................................................   1   1   seq
    ....................Nuugaacucgagauuaaauggc......................................................................   2   1   seq
    ....................guugaacucgagUuuaaauggc......................................................................   6   1   seq
    ....................gCugaacucgagauuaaauggc......................................................................   1   1   seq
    ....................guugaacucgagNuuaaauggc......................................................................   1   1   seq
    ....................guugaacucgagaAuaaauggc......................................................................   1   1   seq
    ....................Auugaacucgagauuaaauggc......................................................................   2   1   seq
```

Mature Star

```
acugccuugaguucucgucuguugaacucgagauuaauggcgucucugaugucuauguugaguagacugacgcugucuguuaacuccuguauuuuagaagacucagggucg
....................guugaacucgagauCaauggc......................................................................  1   1  seq
....................guugaacucgagauAaauggc......................................................................  3   1  seq
....................guugaacucgagauuaauAgc......................................................................  1   1  seq
....................Uuugaacucgagauuaauggc......................................................................  2   1  seq
.....................uugaacucgagauuaauggcg.....................................................................  24  0  seq
.....................Gugaacucgagauuaauggcgu....................................................................  1   1  seq
.....................uugaacucgagauuaauggcgu....................................................................  9   0  seq
.....................uugaacucgagauuaauggcguc...................................................................  3   0  seq
......................ugaacucgagauGaauggcgu....................................................................  1   1  seq
......................ugaacucgagauuaauggcgu....................................................................  56  0  seq
......................ugaacucgagauuaauggcguc...................................................................  1   0  seq
......................ugaacucgagauuaauggcgucucugauguc..........................................................  3   0  seq
.......................gaacucgagauuaauggcguc...................................................................  17  0  seq
.......................Naacucgagauuaauggcguc...................................................................  1   1  seq
.......................gaacucgagauuaauggcgucu..................................................................  1   0  seq
........................aacucgagauuaauggcgucu..................................................................  1   0  seq
............................cgagauuaauggcgucucuga..............................................................  1   0  seq
............................................ucugaugucuauguugaguag..............................................  6   0  seq
....................................................cuauguugaguagacugacgc......................................  12  0  seq
....................................................cuauguugaAuagacugacgc......................................  1   1  seq
....................................................cuauguugaguagaAugacgc......................................  1   1  seq
....................................................cuauguugaguagaUugacgc......................................  1   1  seq
.....................................................uauguugaguagacugacgcu.....................................  5   0  seq
......................................................auguugaguagacugacgcugu...................................  1   0  seq
.......................................................uguugaguagacugCcgcugu...................................  1   1  seq
........................................................guugaAuagacugacgcuguc..................................  2   1  seq
........................................................guugaguagacugacgcuguc..................................  5   0  seq
...........................................................gaguagacugacgcugucugu...............................  1   0  seq
..................................................................cugacgcugucGguuaacucc........................  1   1  seq
...................................................................ugacgcugucuguuaacucc........................  1   0  seq
...................................................................ugacgcugucuguuaacuccu.......................  1   0  seq
.....................................................................acgcugucuguuaacuccugu.....................  3   0  seq
.........................................................................ugucuguuaacuccuguauuu.................  1   0  seq
.........................................................................ugucuguuaacuccuguauuuuagaagacuc.......  3   0  seq
...........................................................................ucuguuaacuccuguauuuuagaagacu........  3   0  seq
...........................................................................ucuguuaacuccuguauuuuagaagacuc.......  1   0  seq
................................................................................uaacuccuguauuuuagaagacucagggu..  1   0  seq
..................................................................................acuccuguauuuuagaagacu........  1   0  seq
...................................................................................cuccuguauuuuagaagacuc.......  6   0  seq
...................................................................................cuccuguauuuuagaagCcuc.......  1   1  seq
.......................................................................................uguauuuuagaagacucaggg...  1   0  seq
```

Provisional ID	:	ML2059_35824
Score total	:	360
Score for star read(s)	:	3.9
Score for read counts	:	349.9
Score for mfe	:	1.7
Score for randfold	:	1.6
Score for cons. seed	:	3
Total read count	:	698
Mature read count	:	678
Loop read count	:	0
Star read count	:	20

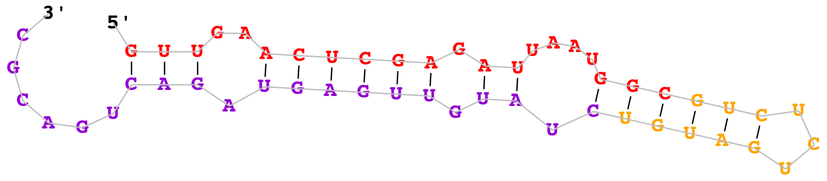

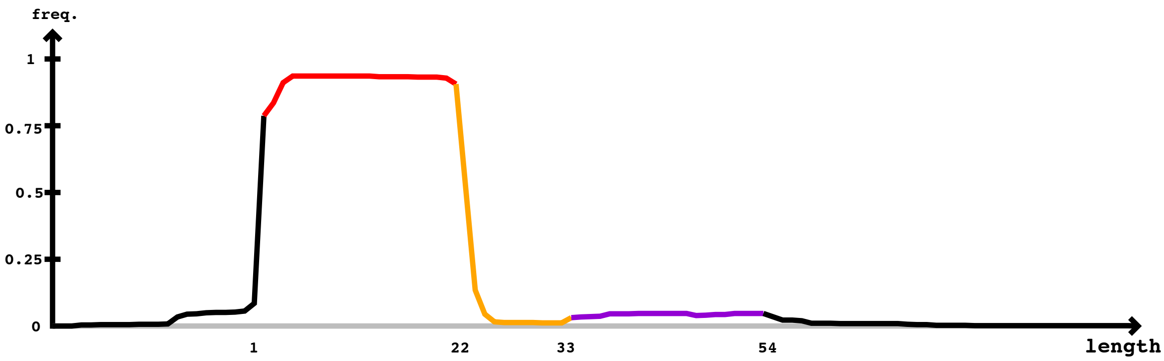

Mature **Star**

```
5'- acugccuugaguucucgucuguugaacucgagauuaauggcgucucugaugucuauguugaguagacugacgcugucuguuaacuccuguauuuuaggagacucagggucg -3'  obs
    acugccuugaguucucgucuguugaacucgagauuaauggcgucucugaugucuauguugaguagacugacgcugucuguuaacuccuguauuuuaggagacucagggucg       exp
    ...((((((((...((((.(((..((((((.((....((((((...)))))).)).)))))).))).)))).............((((((.....))))))))))))))..  reads  mm  sample
    .cugccuugaguucucCucugu.........................................................................................  1      1   seq
    .cugccuugaguucuUgucugu.........................................................................................  1      1   seq
    .cugccuugaguucucgucugu.........................................................................................  1      0   seq
    ...gccuugaguucucgucuguug.......................................................................................  1      0   seq
    .......ugaguucucgucuguugaacu...................................................................................  1      0   seq
    ..........guucucgucuguugaacucgagauuaauggc......................................................................  1      0   seq
    ...........uucucgucuguugaacucgag...............................................................................  2      0   seq
    ...........uucucgucuguugaacucgagauuaauggc......................................................................  17     0   seq
    ...........uucucgucuguugaacucgagauuaauggcg.....................................................................  1      0   seq
    ............ucucgucuguugaacucgagauuaauggc......................................................................  6      0   seq
    ............ucucgucuguugaacucgagauuaauggcguc...................................................................  2      0   seq
    .............cucgucuguugaacucgagauuaauggcgu....................................................................  1      0   seq
    ..............ucgucuguugaacucAagauuaauggc......................................................................  1      1   seq
    ..............ucgucuguugaacucgagauuaauggcg.....................................................................  2      0   seq
    ...............cgucuguugaacucgagauua...........................................................................  1      0   seq
    .................ucuguugaacucAagauuaauggcguc...................................................................  1      1   seq
    ..................cuguugaacucgagauuaaug........................................................................  3      0   seq
    ...................uguugaacucgagauAaaugg.......................................................................  1      1   seq
    ...................uguugaacucgagaAuaaugg.......................................................................  1      1   seq
    ...................uguugaacucgagauuaaugg.......................................................................  15     0   seq
    ...................uguugaacucgagauuaauggc......................................................................  1      0   seq
    ...................uguugaacucgagauuaauggcgu....................................................................  1      0   seq
    ...................uguugaacucAagauuaauggcguc...................................................................  1      1   seq
    ...................uguugaacucgagauuaauggcguc...................................................................  2      0   seq
    ....................guugaacucgagUuuaauggc......................................................................  6      1   seq
    ....................guugaacucgagauuaauAgc......................................................................  1      1   seq
    ....................guugaacucgagauuaauggc......................................................................  515    0   seq
    ....................Uuugaacucgagauuaauggc......................................................................  2      1   seq
    ....................guugaacucgagauCaauggc......................................................................  1      1   seq
    ....................guugaacucgagauGaauggc......................................................................  1      1   seq
    ....................Nuugaacucgagauuaauggc......................................................................  2      1   seq
    ....................guugaacucgagaAuaauggc......................................................................  1      1   seq
    ....................gCugaacucgagauuaauggc......................................................................  1      1   seq
```

```
                    Mature                        Star
acugccuugaguucucgucuguugaacucgagauuaauggcgucucugauguCuauguugaguagacugacgcugucuguuaacuccuguauuuuaggagacucagggucg
....................Auugaacucgagauuaauggc......................................................................	2	1	seq
....................guugaacucgagNuuaauggc......................................................................	1	1	seq
....................guugaacucgagauAaauggc......................................................................	3	1	seq
....................guugaacucgagauuaauggU......................................................................	2	1	seq
.....................uugaacucgagauuaauggcg.....................................................................	24	0	seq
.....................Gugaacucgagauuaauggcgu....................................................................	1	1	seq
.....................uugaacucgagauuaauggcgu....................................................................	9	0	seq
.....................uugaacucgagauuaauggcguc...................................................................	3	0	seq
......................ugaacucgagauGaauggcgu....................................................................	1	1	seq
......................ugaacucgagauuaauggcgu....................................................................	56	0	seq
......................ugaacucgagauuaauggcguc...................................................................	1	0	seq
......................ugaacucgagauuaauggcgucucugauguc..........................................................	3	0	seq
.......................Naacucgagauuaauggcguc...................................................................	1	1	seq
.......................gaacucgagauuaauggcguc...................................................................	17	0	seq
.......................gaacucgagauuaauggcgucu..................................................................	1	0	seq
........................aacucgagauuaauggcgucu..................................................................	1	0	seq
............................cgagauuaauggcgucucuga..............................................................	1	0	seq
............................................ucugaugucuauguugaguag..............................................	6	0	seq
....................................................cuauguugaguagacugacgc......................................	12	0	seq
....................................................cuauguugaAuagacugacgc......................................	1	1	seq
....................................................cuauguugaguagaAugacgc......................................	1	1	seq
....................................................cuauguugaguagaUugacgc......................................	1	1	seq
.....................................................uauguugaguagacugacgcu.....................................	5	0	seq
......................................................auguugaguagacugacgcugu...................................	1	0	seq
.......................................................uguugaguagacugCcgcugu...................................	1	1	seq
........................................................guugaguagacugacgcuguc..................................	5	0	seq
........................................................guugaAuagacugacgcuguc..................................	2	1	seq
...........................................................gaguagacugacgcugucugu...............................	1	0	seq
..................................................................cugacgcugucGguuaacucc........................	1	1	seq
...................................................................ugacgcugucuguuaacucc........................	1	0	seq
...................................................................ugacgcugucuguuaacuccu.......................	1	0	seq
.....................................................................acgcugucuguuaacuccugu.....................	3	0	seq
.........................................................................ugucuguuaacuccuguauuu.................	1	0	seq
..........................................................................................auuuuaggagacucagggucg	1	0	seq
```

Provisional ID	:	ML3506_43832
Score total	:	360
Score for star read(s)	:	3.9
Score for read counts	:	349.9
Score for mfe	:	1.7
Score for randfold	:	1.6
Score for cons. seed	:	3
Total read count	:	698
Mature read count	:	678
Loop read count	:	0
Star read count	:	20

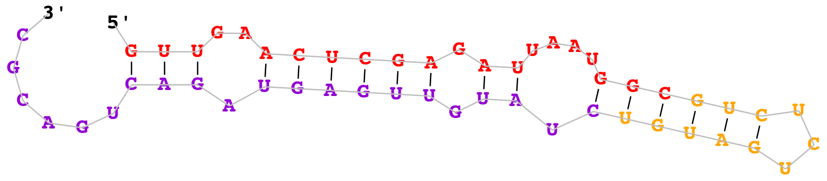

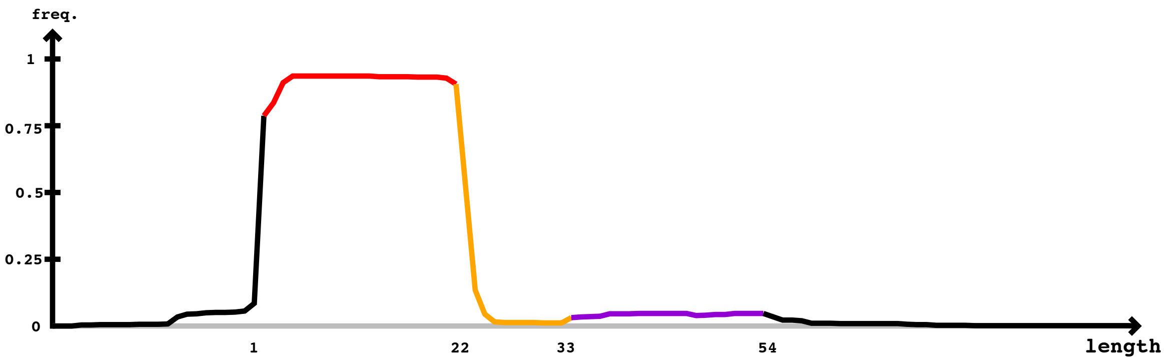

Mature **Star**

```
5'- acugccuugaguucucgucuguugaacucgagauuaauggcgucucugaugucuauguugaguagacugacgcugucuguuaacuccuguauuuuaggagacucagggucg   -3'   obs
    acugccuugaguucucgucuguugaacucgagauuaauggcgucucugaugucuauguugaguagacugacgcugucuguuaacuccuguauuuuaggagacucagggucg         exp
    ...((((((((...((((.(((..((((((.((....((((((...)))))).)).)))))).))).))))...........((((((.....))))))))))))))..   reads   mm   sample
    .cugccuugaguucucgucugu.........................................................................................   1   0   seq
    .cugccuugaguucuUgucugu.........................................................................................   1   1   seq
    .cugccuugaguucucCucugu.........................................................................................   1   1   seq
    ...gccuugaguucucgucuguug.......................................................................................   1   0   seq
    ......ugaguucucgucuguugaacu....................................................................................   1   0   seq
    ..........guucucgucuguugaacucgagauuaauggc......................................................................   1   0   seq
    ...........uucucgucuguugaacucgag...............................................................................   2   0   seq
    ...........uucucgucuguugaacucgagauuaauggc......................................................................   17   0   seq
    ...........uucucgucuguugaacucgagauuaauggcg.....................................................................   1   0   seq
    ............ucucgucuguugaacucgagauuaauggc......................................................................   6   0   seq
    ............ucucgucuguugaacucgagauuaauggcguc...................................................................   2   0   seq
    .............cucgucuguugaacucgagauuaauggcgu....................................................................   1   0   seq
    ..............ucgucuguugaacucAagauuaauggc......................................................................   1   1   seq
    ..............ucgucuguugaacucgagauuaauggcg.....................................................................   2   0   seq
    ...............cgucuguugaacucgagauua...........................................................................   1   0   seq
    .................ucuguugaacucAagauuaauggcguc...................................................................   1   1   seq
    ..................cuguugaacucgagauuaaug........................................................................   3   0   seq
    ...................uguugaacucgagaAuaaugg.......................................................................   1   1   seq
    ...................uguugaacucgagauAaaugg.......................................................................   1   1   seq
    ...................uguugaacucgagauuaaugg.......................................................................   15   0   seq
    ...................uguugaacucgagauuaauggc......................................................................   1   0   seq
    ...................uguugaacucgagauuaauggcgu....................................................................   1   0   seq
    ...................uguugaacucgagauuaauggcguc...................................................................   2   0   seq
    ...................uguugaacucAagauuaauggcguc...................................................................   1   1   seq
    ....................Uuugaacucgagauuaauggc......................................................................   2   1   seq
    ....................guugaacucgagUuuaauggc......................................................................   6   1   seq
    ....................guugaacucgagNuuaauggc......................................................................   1   1   seq
    ....................guugaacucgagauuaauAgc......................................................................   1   1   seq
    ....................gCugaacucgagauuaauggc......................................................................   1   1   seq
    ....................guugaacucgagauCaauggc......................................................................   1   1   seq
    ....................guugaacucgagauuaauggU......................................................................   2   1   seq
    ....................guugaacucgagauuaauggc......................................................................   515   0   seq
    ....................guugaacucgagauGaauggc......................................................................   1   1   seq
```

```
                    Mature                       Star
acugccuugaguucucgucuguugaacucgagauuaauggcgucucugaugucuauguugaguagacugacgcugucuguuaacuccuguauuuuaggagacucagggucg
....................Nuugaacucgagauuaauggc......................................................................  2   1   seq
....................Auugaacucgagauuaauggc......................................................................  2   1   seq
....................guugaacucgagaAuaauggc......................................................................  1   1   seq
....................guugaacucgagauAaauggc......................................................................  3   1   seq
.....................uugaacucgagauuaauggcg.....................................................................  24  0   seq
.....................Gugaacucgagauuaauggcgu....................................................................  1   1   seq
.....................uugaacucgagauuaauggcgu....................................................................  9   0   seq
.....................uugaacucgagauuaauggcguc...................................................................  3   0   seq
......................ugaacucgagauGaauggcgu....................................................................  1   1   seq
......................ugaacucgagauuaauggcgu....................................................................  56  0   seq
......................ugaacucgagauuaauggcguc...................................................................  1   0   seq
......................ugaacucgagauuaauggcgucucugauguc..........................................................  3   0   seq
.......................Naacucgagauuaauggcguc...................................................................  1   1   seq
.......................gaacucgagauuaauggcguc...................................................................  17  0   seq
.......................gaacucgagauuaauggcgucu..................................................................  1   0   seq
........................aacucgagauuaauggcgucu..................................................................  1   0   seq
............................cgagauuaauggcgucucuga..............................................................  1   0   seq
............................................ucugaugucuauguugaguag..............................................  6   0   seq
....................................................cuauguugaAuagacugacgc......................................  1   1   seq
....................................................cuauguugaguagacugacgc......................................  12  0   seq
....................................................cuauguugaguagaAugacgc......................................  1   1   seq
....................................................cuauguugaguagaUugacgc......................................  1   1   seq
.....................................................uauguugaguagacugacgcu.....................................  5   0   seq
......................................................auguugaguagacugacgcugu...................................  1   0   seq
.......................................................uguugaguagacugCcgcugu...................................  1   1   seq
........................................................guugaguagacugacgcuguc..................................  5   0   seq
........................................................guugaAuagacugacgcuguc..................................  2   1   seq
...........................................................gaguagacugacgcugucugu...............................  1   0   seq
..................................................................cugacgcugucGguuaacucc........................  1   1   seq
...................................................................ugacgcugucuguuaacucc........................  1   0   seq
...................................................................ugacgcugucuguuaacuccu.......................  1   0   seq
.....................................................................acgcugucuguuaacuccugu.....................  3   0   seq
.........................................................................ugucuguuaacuccuguauuu.................  1   0   seq
..........................................................................................auuuuaggagacucagggucg  1   0   seq
```

Provisional ID	:	ML2945_41526
Score total	:	232.7
Score for star read(s)	:	3.9
Score for read counts	:	229
Score for mfe	:	-0.9
Score for randfold	:	-2.2
Score for cons. seed	:	3
Total read count	:	461
Mature read count	:	460
Loop read count	:	0
Star read count	:	1

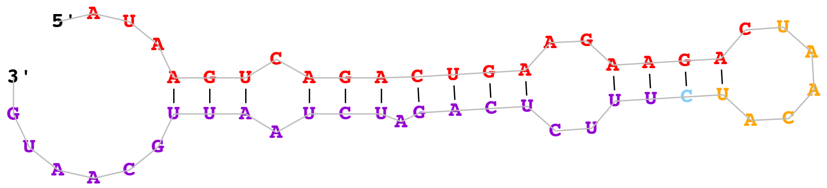

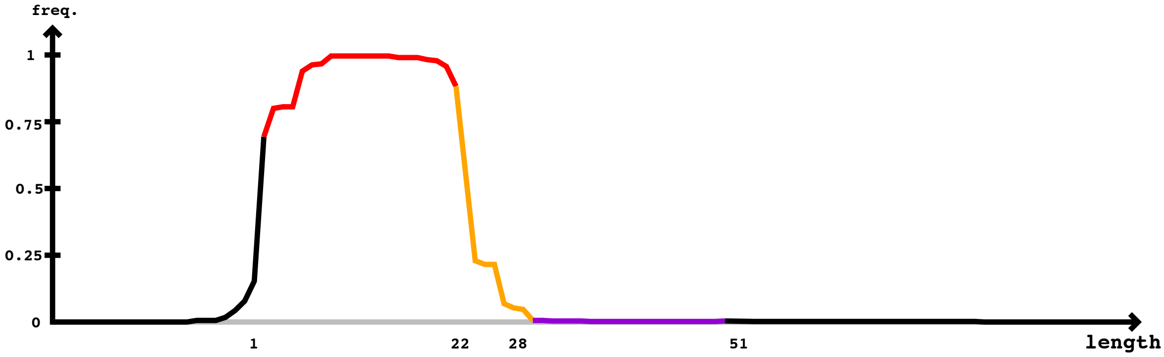

Mature Star

5'-	ggaccaauucaugacauuguauaagucagacugaagaagacuaacaucuuucucagaucuaauugcaauggauauacgaguaucaucugcgaagcaccugaugaucgagug	-3'	obs	
	ggaccaauucaugacauuguauaagucagacugaagaagacuaacaucuuucucagaucuaauugcaauggauauacgaguaucaucugcgaagcaccugaugaucgagug		exp	
	..............(((((((...((.(((((((..((((.....))))..)))).))).)))))))))......(((.(((((..(((...)))..))))).)))....,	reads	mm	sample
	.............acauuguauaagucagacuga...	2	0	seq
	.............acauuguauaagucagacugaagaagacu...	1	0	seq
	................uuguauaagucagacugU...	1	1	seq
	................uuguauaagucagacugaaga..	3	0	seq
	................uuguauaagucagacugaagaagac..	2	0	seq
	.................uguauaagucagacugaaga..	1	0	seq
	.................uguauaagucagacugaagaa...	1	0	seq
	.................uguGuaagucagacugaagaagac..	1	1	seq
	.................uguauaagucagacugaagaagac..	1	0	seq
	.................uguauaagucagacAgaagaagac..	1	1	seq
	.................uguauaagucagacugaagaagacuaac..	7	0	seq
	.................uguauaagucagacugaagaagacuaacauc...	1	0	seq
	..................guauaagucagacugaagaag..	10	0	seq
	..................guauaagucagacugaagaagU...	1	1	seq
	..................guauaagucagacugaagaagac..	2	0	seq
	..................guauaagucagacugaagaagacu...	2	0	seq
	..................guauaagucagacugaagaagacua..	2	0	seq
	..................guauaagucagacugaagaagacuaacauc...	1	0	seq
	...................uauaagucagacugaagaa...	1	0	seq
	...................uauaagucagacugaagaaga...	37	0	seq
	....................auaagucagacugaagaaUac..	1	1	seq
	....................auaagucagacugaGgaagac..	2	1	seq
	....................auaagucagacugaaAaagac..	1	1	seq
	....................auaagucagacugaagaagac..	263	0	seq
	....................Uuaagucagacugaagaagac..	1	1	seq
	....................auaagucagacugaagaagacu...	2	0	seq
	....................auaagucagacugaagaagacua..	1	0	seq
	....................auaagucagacugaagaagacuaacauc...	5	0	seq
	.....................uaagucagacugaagaag..	1	0	seq
	.....................uaagucagacugaagaagacu...	53	0	seq
	......................aagucagacugaagGagacua..	1	1	seq
	......................aagucagacugaagaagacua..	2	0	seq
	........................gucagGcugaagaagacuaac..	1	1	seq

Mature **Star**

```
ggaccaauucaugacauuguauaagucagacugaagaagacuaacaucuuucucagaucuaauugcaaugggauauacgaguaucaucugcgaagcaccugaugaucgagug
........................gucagacugaaUaagacuaac..................................................................   1    1    seq
........................gucagacugaagaaUacuaac..................................................................   1    1    seq
........................gucagacugaagaagacuaaU..................................................................   1    1    seq
........................gucagacugaagaagacuaac..................................................................   64   0    seq
.........................ucagacugaagaagacua....................................................................   1    0    seq
.........................ucagacugaagaagacuaaca.................................................................   8    0    seq
.........................ucagacugaagaagacuaacau................................................................   1    0    seq
.........................ucagacugaagaagacuaacaucuA.............................................................   1    1    seq
.........................ucagacugaagaagacuaacaucuuucuc.........................................................   1    0    seq
..........................cagacugaagaagacuaacau................................................................   2    0    seq
...........................agacugaagaagacuaacauc...............................................................   15   0    seq
................................................uuucucagaucuaauugcaaug.........................................   1    0    seq
....................................................................uggauauGcgaguaucaucugcgaagc................   1    1    seq
```

Provisional ID	:	ML0323_10049
Score total	:	229.3
Score for star read(s)	:	3.9
Score for read counts	:	228
Score for mfe	:	0.1
Score for randfold	:	-2.2
Score for cons. seed	:	-0.6
Total read count	:	459
Mature read count	:	266
Loop read count	:	148
Star read count	:	45

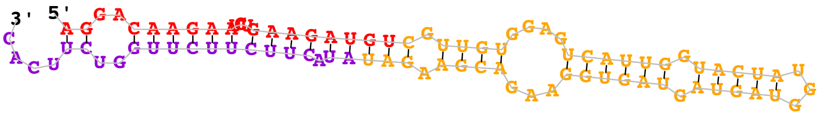

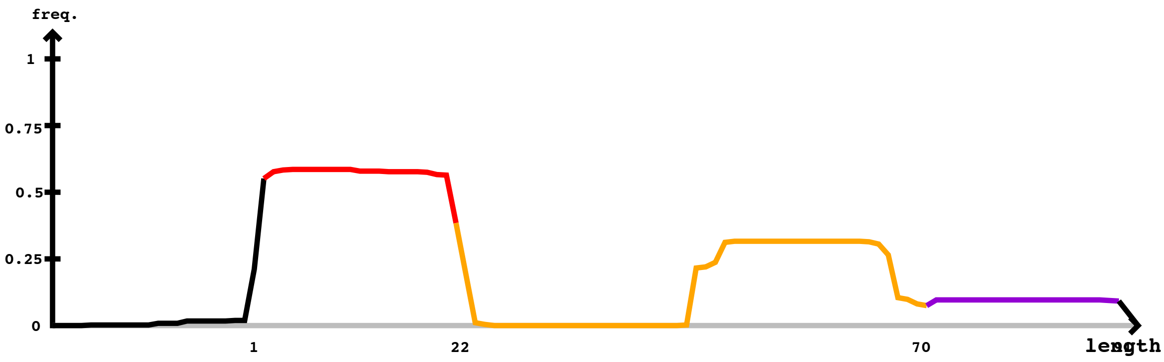


```
                                   Mature                                                            Star
5'- acggaaaaggaauccagcacaggacaagaaucugaagaugucguuguggagucauugguacuaugguaguaguaguggaagacgaagauauacuucuucuuggucuucaca  -3'   obs
    acggaaaaggaauccagcacaggacaagaaucugaagaugucguuguggagucauugguacuaugguaguaguaguggaagacgaagauauacuucuucuuggucuucaca         exp
    ..(((.......))).....((..((((((...(((((((((.((((....((((((.(((((...))))).))))))...)))).))))).))))))))))..)).....   reads   mm   sample
    ..ggaaaaggaauccagcacagg........................................................................................   1       0    seq
    .........gaauccagcacaggacaagaa.................................................................................   2       0    seq
    .........Aaauccagcacaggacaagaa.................................................................................   1       1    seq
    ............uccagcacaggacaagaaucu..............................................................................   1       0    seq
    ............uccagcacaggacaagaaucugaagC.........................................................................   3       1    seq
    .................cacaggacaagaaucugaaga.........................................................................   1       0    seq
    ...................caggacaagaaucugaag..........................................................................   1       0    seq
    ...................caggacaagaaucugaagau........................................................................   1       0    seq
    ...................caggacaagaaucugaagaug.......................................................................   79      0    seq
    ...................caggacaagaaucugaagaAg.......................................................................   1       1    seq
    ...................Uaggacaagaaucugaagaug.......................................................................   1       1    seq
    ...................cagAacaagaaucugaagaug.......................................................................   2       1    seq
    ...................caggacaagaaucugaagaugG......................................................................   1       1    seq
    ...................caggacaagaaucugaagaugu......................................................................   4       0    seq
    ....................aggacaagaaucugaagaug.......................................................................   1       0    seq
    ....................aggacaagaaucugCagaugu......................................................................   1       1    seq
    ....................Nggacaagaaucugaagaugu......................................................................   2       1    seq
    ....................aggacaagaaucugaagaugu......................................................................   153     0    seq
    ....................aggacaagaaucugaagauguc.....................................................................   1       0    seq
    ....................aggacaagaaucugaagauguU.....................................................................   1       1    seq
    .....................ggacaagaaucugaagauguc.....................................................................   12      0    seq
    ......................gacaagaaucugaagaugucg....................................................................   3       0    seq
    .......................acaagaaucugaagaugucgu...................................................................   2       0    seq
    ................................................................Nguaguaguaguggaagacga..........................   1       1    seq
    .................................................................guaguaguaguggaagac............................   1       0    seq
    .................................................................guaguaguaguggaagacg...........................   4       0    seq
    .................................................................guaguaguaguggaagacga..........................   18      0    seq
    .................................................................guaguaguagugggaCgacgaa.........................   2       1    seq
    .................................................................guaguaguaguggaagacgaa.........................   68      0    seq
    .................................................................guaguaguaguggaUgacgaa.........................   1       1    seq
    .................................................................guagAaguaguggaagacgaa.........................   3       1    seq
    .................................................................guaguaguaguggaGgacgaa.........................   1       1    seq
    .................................................................guaguaguaguggaagacgaaU........................   1       1    seq
```

Mature Star

acggaaaaggaauccagcacaggacaagaaucugaagaugucguuguggagucauugguacuaugguaguaguaguggaagacgaagauauacuucuucuuggucuucaca			
....guaguaguaguggaagacgaaAa.......................	1	1	seq
....uaguaguaguggaAacgaag.........................	1	1	seq
....uaguaguaguggaagacgaag........................	1	0	seq
....aguaguaguggaagacgaaga.......................	8	0	seq
....guaguaguggaagacgaag........................	1	0	seq
....Nuaguaguggaagacgaagau......................	1	1	seq
....guaguaguggaagaUgaagau......................	1	1	seq
....guaguaguggaagacgaagau......................	30	0	seq
....guaguUguggaagacgaagau......................	1	1	seq
....guaguaguggaagacgaagauU.....................	1	1	seq
....uaguaguggaagacgaagaua.....................	2	0	seq
....auauacuucuucuuggucuuc...	1	0	seq
....uauacuucuucuuggucuuca..	1	0	seq
....auacuucuucuuggucuucac.	25	0	seq
....auacuucuucuuggCcuucac.	1	1	seq
....auacuucuucuuggucuCcac.	3	1	seq
....auacuucuucuugUucuucac.	1	1	seq
....uacuucuucuuggucuucacU	1	1	seq
....uacuucuucuugAucuucaca	1	1	seq
....uacuucuucuuggucuGcaca	1	1	seq
....uacuucuucuuggucuCcaca	2	1	seq
....uacuucuucuuggucuucaGa	1	1	seq
....uacuucuucuuggucuucaca	7	0	seq

Provisional ID	:	ML0105_4573
Score total	:	191.6
Score for star read(s)	:	3.9
Score for read counts	:	196.9
Score for mfe	:	-9.9
Score for randfold	:	-2.2
Score for cons. seed	:	3
Total read count	:	398
Mature read count	:	376
Loop read count	:	14
Star read count	:	8

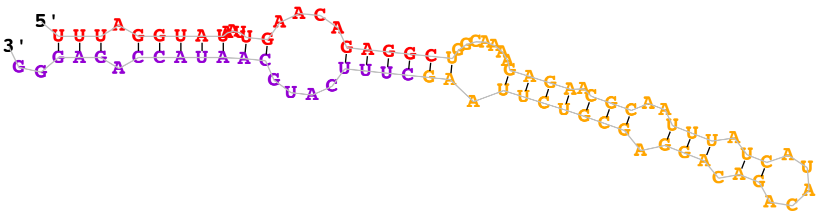

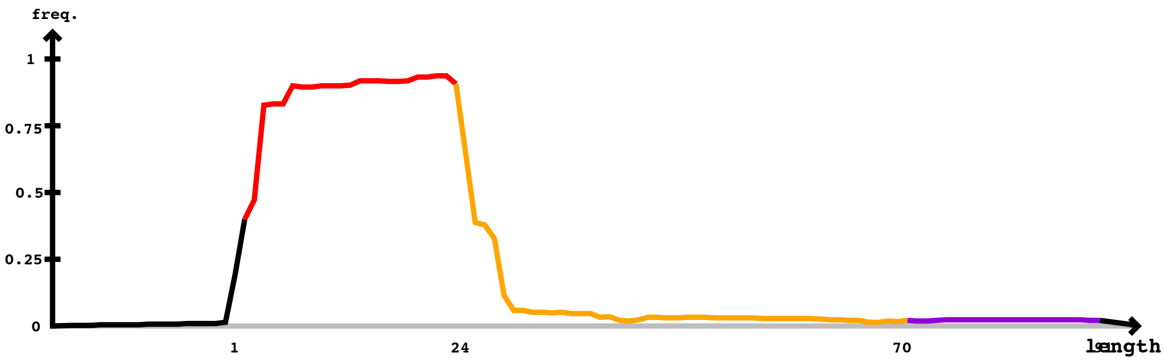

Mature **Star**

```
5'- gagaccacaggacagcguuuuagguauaauugaacagaggcuggcaaaagagaacgcaauuuaucauacagacaggagcgucuuaagcuuucaugcaauaccagagggugc  -3'   obs
    gagaccacaggacagcguuuuagguauaauugaacagaggcuggcaaaagagaacgcaauuuaucauacagacaggagcgucuuaagcuuucaugcaauaccagagggugc        exp
    .............((.((((.(((((...((....((((((.......((((.(((..(((.((.....)).))).))))))).))))))....))))))))))).))..   reads   mm   sample
    gagaccacaggacagcUuuuuagg.......................................................................................   1   1   seq
    ...accacaggacagcguuuuagg.......................................................................................   1   0   seq
    ........aggacagcguuuuagguauaauugaacagagC.......................................................................   1   1   seq
    ............cagcguuuuagguauaauuga..............................................................................   1   0   seq
    ................guuuuagguauaauugaacagaggc......................................................................   2   0   seq
    .................uuuuagguauaauugaacagagg.......................................................................   1   0   seq
    .................uuuuagguauaauugaacagagC.......................................................................   3   1   seq
    .................uuuuagguauaauugaacGgaggc......................................................................   1   1   seq
    .................uuuuagAuauaauugaacagaggc......................................................................   1   1   seq
    .................uuuuagguauGauugaacagaggc......................................................................   1   1   seq
    .................uuuuagguauaauugaacagaggc......................................................................   64  0   seq
    .................Nuuuagguauaauugaacagaggc......................................................................   1   1   seq
    .................uuuuagguauaauugaacagaggcu.....................................................................   2   0   seq
    .................uuuuagguauaauugaacagaggcuggc..................................................................   1   0   seq
    .................uuuuagguauaauugaacagaggcuggca.................................................................   1   0   seq
    ..................uuuagguauaauugaacagagg.......................................................................   4   0   seq
    ..................uuuagguauaauugaacagagC.......................................................................   2   1   seq
    ..................uuuagguauaauugUacagaggc......................................................................   1   1   seq
    ..................uuuagguauaauugaacagaggc......................................................................   78  0   seq
    ..................Nuuagguauaauugaacagaggc......................................................................   1   1   seq
    ..................uuuagguauaauugaacagaggcu.....................................................................   2   0   seq
    ..................uGuagguauaauugaacagaggcuggc..................................................................   1   1   seq
    ...................uuagguauaauugaacagagg.......................................................................   2   0   seq
    ...................uuagAuauaauugaacagaggc......................................................................   1   1   seq
    ...................uuaggGauaauugaacagaggc......................................................................   1   1   seq
    ...................uuagguauaauugaacagaggc......................................................................   9   0   seq
    ...................uuagguauaauugaacagaggcu.....................................................................   3   0   seq
    ...................uuagguauaauugaacagaggcug....................................................................   1   0   seq
    ...................uuagguauaauugaacagaggcuU....................................................................   1   1   seq
    ...................uuagguauaauugaacagaggcugg...................................................................   4   0   seq
    ...................uuagguauaauugaacagaggcuggc..................................................................   8   0   seq
    ...................uuagguauaauugaacagaggcuggca.................................................................   1   0   seq
    ....................uagAuauaauugaacagaggc......................................................................   2   1   seq
```

Mature   Star

```
gagaccacaggacagcguuuuagguauaauugaacagaggcuggcaaaagagaacgcaauuuaucauacagacaggagcgucuuaagcuuucaugcaauaccagagggugc
....................uagguauaauugaacagaggc......................................................................  51  0  seq
....................uagguauaauugaacagaggcug....................................................................  1   0  seq
....................uagguauaauugaacagagAcugg...................................................................  1   1  seq
....................uagguauaauugaacagaggcugg...................................................................  2   0  seq
....................uagguauaauugaacagaggcugC...................................................................  1   1  seq
....................uagguauaauugaaGagaggcuggc..................................................................  1   1  seq
....................uagguauaauugaacagaggcuggc..................................................................  59  0  seq
....................uagguauaauugaacGgaggcuggc..................................................................  3   1  seq
....................uagguauaauugaaAagaggcuggc..................................................................  1   1  seq
....................uagAuauaauugaacagaggcuggc..................................................................  2   1  seq
....................uagguauaauugaaNagaggcuggc..................................................................  1   1  seq
....................uaggCauaauugaacagaggcuggc..................................................................  1   1  seq
....................uagguauaauugaacagagAcuggc..................................................................  5   1  seq
....................uagguauaauugaaNagaggcuggca.................................................................  1   1  seq
....................uagguauaauugaacagaggcuggca.................................................................  15  0  seq
....................uagguauaaCugaacagaggcuggca.................................................................  1   1  seq
....................uagguauaauugaacGgaggcuggca.................................................................  2   1  seq
....................uagguauaauugaacagaAgcuggca.................................................................  1   1  seq
....................uagguauaauugaacagaggcCggcaaa...............................................................  1   1  seq
.....................agguauaauugaacagaggcu.....................................................................  1   0  seq
.....................Ggguauaauugaacagaggcuggcaaaagagaacgc......................................................  1   1  seq
.......................guauaauugaacagaggc......................................................................  1   0  seq
.......................guauaauugaacagaggcug....................................................................  1   0  seq
.......................guauaauugaacagaggcugg...................................................................  13  0  seq
.......................guaAaauugaacagaggcugg...................................................................  1   1  seq
.......................guauaauugaacagaggcuggc..................................................................  7   0  seq
.......................guauaauugaacagaggcuggca.................................................................  1   0  seq
.......................guauaauugaacagagAcuggca.................................................................  1   1  seq
.......................guauaauugaacagaggcuggcaaG...............................................................  1   1  seq
.......................guauaauugaacagaggcuggcaCa...............................................................  1   1  seq
.......................guauaauugaacagaggcuggcaaaag.............................................................  1   0  seq
.......................guauaauugaacagaggcuggcaaaagag...........................................................  1   0  seq
..........................uaauugaacagaggcuggA..................................................................  1   1  seq
..........................uaauugaacagaggcuggcaaaagaC...........................................................  1   1  seq
.............................uugaacagaggcuggcaaa...............................................................  1   0  seq
..............................ugaacGgaggcuggcaaaagagaac........................................................  2   1  seq
..............................ugaacagaggcuggcaaaagagaac........................................................  3   0  seq
..............................ugaacagaggcugAcaaaagagaac........................................................  1   1  seq
..............................ugaacagaggcuggcaaaagagaacgcaauuu.................................................  1   0  seq
...................................agaggcuggcaaaagagaacgca.....................................................  1   0  seq
....................................gaggcuggcaaaagagaacgc......................................................  6   0  seq
......................................ggcuggcaaaagagaacgcaauu..................................................  1   0  seq
......................................ggGuggcaaaagagaacgcaauuuaucau............................................  1   1  seq
.........................................uggcaaaagagaacgcaauuuauc..............................................  1   0  seq
................................................agagaacgcaauuuaucauacagacaggagc................................  1   0  seq
...................................................gaacgcaauuuaucauacaga.......................................  1   0  seq
........................................................caauuuaucauacagaUaggagcgucuuaa.........................  1   1  seq
.........................................................aauuuaucauacagacaggag.................................  1   0  seq
...........................................................uuuaucauacagacaggagUgucu............................  2   1  seq
............................................................uuaucauacagacaggagcgu..............................  1   0  seq
............................................................uuaucauacagaUaggagcgucuuaag........................  1   1  seq
............................................................uuaucauacagaUaggagcgucuuaagc.......................  2   1  seq
.............................................................uaucauacagaUaggagcgucuuaag........................  1   1  seq
................................................................cauacagacaggagcgucu............................  1   0  seq
.................................................................auacagaUaggagcgucuuaagc.......................  1   1  seq
....................................................................................agcuuucaugcaauaccagaggg...  2   0  seq
.......................................................................................cuuucaugcaauaccagag.....  1   0  seq
.......................................................................................cuuucaugcaauaccagaggg...  2   0  seq
.......................................................................................cuuucaugcaauaccagagggug.  1   0  seq
........................................................................................uuucaugcaauaccagagggug.  1   0  seq
........................................................................................uuucaugcaauaccagagggugc  1   0  seq
..........................................................................................ucaugcaauaccagagggugc  1   0  seq
...........................................................................................caugcaauaccagagggugc  1   0  seq
```

Provisional ID	:	ML1356_29007
Score total	:	191.6
Score for star read(s)	:	3.9
Score for read counts	:	196.9
Score for mfe	:	-9.9
Score for randfold	:	-2.2
Score for cons. seed	:	3
Total read count	:	398
Mature read count	:	376
Loop read count	:	14
Star read count	:	8

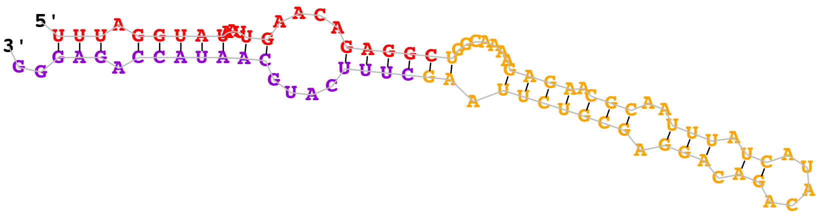

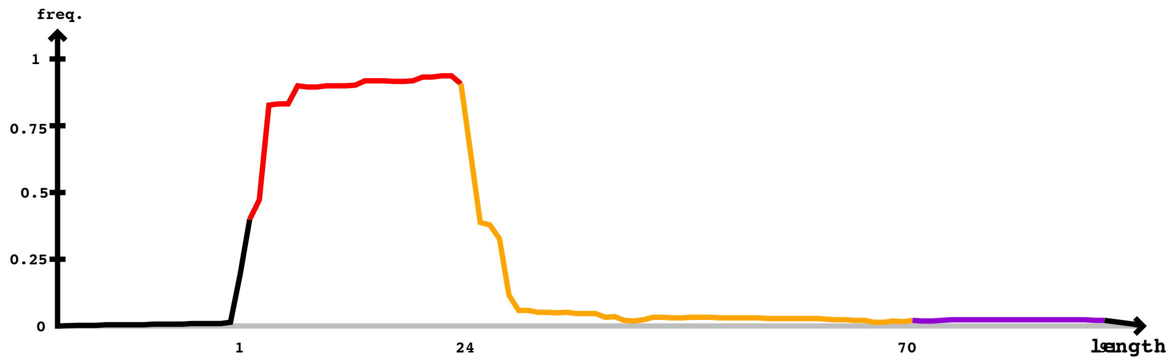


```
                 Mature                                                     Star
5'- gagaccacaggacagcguuuuagguauaauugaacagaggcuggcaaaagagaacgcaauuuaucauacagacaggagcgucuuaagcuuucaugcaauaccagaggguugc  -3'   obs
    gagaccacaggacagcguuuuagguauaauugaacagaggcuggcaaaagagaacgcaauuuaucauacagacaggagcgucuuaagcuuucaugcaauaccagagggugc        exp
    ..............((.((((.(((((...((....((((((.......((((.(((..(((.((.....)).))).))))))).))))))....))))))))))).))..   reads   mm   sample
    gagaccacaggacagcUuuuuagg.......................................................................................   1       1    seq
    ...accacaggacagcguuuuagg.......................................................................................   1       0    seq
    ........aggacagcguuuuagguauaauugaacagagC.......................................................................   1       1    seq
    ...........cagcguuuuagguauaauuga...............................................................................   1       0    seq
    ................guuuuagguauaauugaacagaggc......................................................................   2       0    seq
    .................uuuuagguauaauugaacagagC.......................................................................   3       1    seq
    .................uuuuagguauaauugaacagagg.......................................................................   1       0    seq
    .................uuuuagguauGauugaacagaggc......................................................................   1       1    seq
    .................Nuuuagguauaauugaacagaggc......................................................................   1       1    seq
    .................uuuuagAuauaauugaacagaggc......................................................................   1       1    seq
    .................uuuuagguauaauugaacGgaggc......................................................................   1       1    seq
    .................uuuuagguauaauugaacagaggc......................................................................   64      0    seq
    .................uuuuagguauaauugaacagaggcu.....................................................................   2       0    seq
    .................uuuuagguauaauugaacagaggcuggc..................................................................   1       0    seq
    .................uuuuagguauaauugaacagaggcuggca.................................................................   1       0    seq
    ..................uuuagguauaauugaacagagC.......................................................................   2       1    seq
    ..................uuuagguauaauugaacagagg.......................................................................   4       0    seq
    ..................uuuagguauaauugaacagaggc......................................................................   78      0    seq
    ..................uuuagguauaauugUacagaggc......................................................................   1       1    seq
    ..................Nuuagguauaauugaacagaggc......................................................................   1       1    seq
    ..................uuuagguauaauugaacagaggcu.....................................................................   2       0    seq
    ..................uGuagguauaauugaacagaggcuggc..................................................................   1       1    seq
    ...................uuagguauaauugaacagagg.......................................................................   2       0    seq
    ...................uuagAuauaauugaacagaggc......................................................................   1       1    seq
    ...................uuaggGauaauugaacagaggc......................................................................   1       1    seq
    ...................uuagguauaauugaacagaggc......................................................................   9       0    seq
    ...................uuagguauaauugaacagaggcu.....................................................................   3       0    seq
    ...................uuagguauaauugaacagaggcug....................................................................   1       0    seq
    ...................uuagguauaauugaacagaggcuU....................................................................   1       1    seq
    ...................uuagguauaauugaacagaggcugg...................................................................   4       0    seq
    ...................uuagguauaauugaacagaggcuggc..................................................................   8       0    seq
    ...................uuagguauaauugaacagaggcuggca.................................................................   1       0    seq
    ....................uagguauaauugaacagaggc......................................................................   51      0    seq
```

Mature **Star**

```
gagaccacaggacagcguuuuagguauaauugaacagaggcuggcaaaagagaacgcaauuuaucauacagacaggagcgucuuaagcuuucaugcaauaccagagggugc
....................uagAuauaauugaacagaggc......................................................................  2   1   seq
....................uagguauaauugaacagaggcug....................................................................  1   0   seq
....................uagguauaauugaacagaggcugg...................................................................  2   0   seq
....................uagguauaauugaacagagAcugg...................................................................  1   1   seq
....................uagguauaauugaacagaggcugC...................................................................  1   1   seq
....................uagguauaauugaaGagaggcuggc..................................................................  1   1   seq
....................uagguauaauugaaNagaggcuggc..................................................................  1   1   seq
....................uagguauaauugaacagaggcuggc..................................................................  59  0   seq
....................uagguauaauugaaAagaggcuggc..................................................................  1   1   seq
....................uagAuauaauugaacagaggcuggc..................................................................  2   1   seq
....................uagguauaauugaacGgaggcuggc..................................................................  3   1   seq
....................uaggCauaauugaacagaggcuggc..................................................................  1   1   seq
....................uagguauaauugaacagagAcuggc..................................................................  5   1   seq
....................uagguauaauugaacagaAgcuggca.................................................................  1   1   seq
....................uagguauaaCugaacagaggcuggca.................................................................  1   1   seq
....................uagguauaauugaacagaggcuggca.................................................................  15  0   seq
....................uagguauaauugaacGgaggcuggca.................................................................  2   1   seq
....................uagguauaauugaaNagaggcuggca.................................................................  1   1   seq
....................uagguauaauugaacagaggcCggcaaa...............................................................  1   1   seq
.....................agguauaauugaacagaggcu.....................................................................  1   0   seq
.....................Ggguauaauugaacagaggcuggcaaaagagaacgc......................................................  1   1   seq
.......................guauaauugaacagaggc......................................................................  1   0   seq
.......................guauaauugaacagaggcug....................................................................  1   0   seq
.......................guauaauugaacagaggcugg...................................................................  13  0   seq
.......................guaAaauugaacagaggcugg...................................................................  1   1   seq
.......................guauaauugaacagaggcuggc..................................................................  7   0   seq
.......................guauaauugaacagagAcuggca.................................................................  1   1   seq
.......................guauaauugaacagaggcuggca.................................................................  1   0   seq
.......................guauaauugaacagaggcuggcaaG...............................................................  1   1   seq
.......................guauaauugaacagaggcuggcaCa...............................................................  1   1   seq
.......................guauaauugaacagaggcuggcaaaag.............................................................  1   0   seq
.......................guauaauugaacagaggcuggcaaaagag...........................................................  1   0   seq
..........................uaauugaacagaggcuggA..................................................................  1   1   seq
..........................uaauugaacagaggcuggcaaaagaC...........................................................  1   1   seq
.............................uugaacagaggcuggcaaa...............................................................  1   0   seq
..............................ugaacGgaggcuggcaaaagagaac........................................................  2   1   seq
..............................ugaacagaggcugAcaaaagagaac........................................................  1   1   seq
..............................ugaacagaggcuggcaaaagagaac........................................................  3   0   seq
..............................ugaacagaggcuggcaaaagagaacgcaauuu.................................................  1   0   seq
...................................agaggcuggcaaaagagaacgca.....................................................  1   0   seq
....................................gaggcuggcaaaagagaacgc......................................................  6   0   seq
......................................ggcuggcaaaagagaacgcaauu..................................................  1   0   seq
......................................ggGuggcaaaagagaacgcaauuuaucau............................................  1   1   seq
.........................................uggcaaaagagaacgcaauuuauc..............................................  1   0   seq
................................................agagaacgcaauuuaucauacagacaggagc................................  1   0   seq
...................................................gaacgcaauuuaucauacaga.......................................  1   0   seq
........................................................caauuuaucauacagaUaggagcgucuuaa.........................  1   1   seq
.........................................................aauuuaucauacagacaggag.................................  1   0   seq
...........................................................uuuaucauacagacaggagUgucu............................  2   1   seq
............................................................uuaucauacagacaggagcgu..............................  1   0   seq
............................................................uuaucauacagaUaggagcgucuuaag........................  1   1   seq
............................................................uuaucauacagaUaggagcgucuuaagc.......................  2   1   seq
.............................................................uaucauacagaUaggagcgucuuaag........................  1   1   seq
................................................................cauacagacaggagcgucu............................  1   0   seq
.................................................................auacagaUaggagcgucuuaagc.......................  1   1   seq
.....................................................................................agcuuucaugcaauaccagaggg...  2   0   seq
.......................................................................................cuuucaugcaauaccagag.....  1   0   seq
.......................................................................................cuuucaugcaauaccagaggg...  2   0   seq
.......................................................................................cuuucaugcaauaccagagggug.  1   0   seq
........................................................................................uuucaugcaauaccagagggug.  1   0   seq
........................................................................................uuucaugcaauaccagagggugc  1   0   seq
..........................................................................................ucaugcaauaccagagggugc  1   0   seq
...........................................................................................caugcaauaccagagggugc  1   0   seq
```

Provisional ID : ML1994_35190
Score total : 179.8
Score for star read(s) : 3.9
Score for read counts : 182.1
Score for mfe : -6.9
Score for randfold : -2.2
Score for cons. seed : 3
Total read count : 369
Mature read count : 367
Loop read count : 0
Star read count : 2

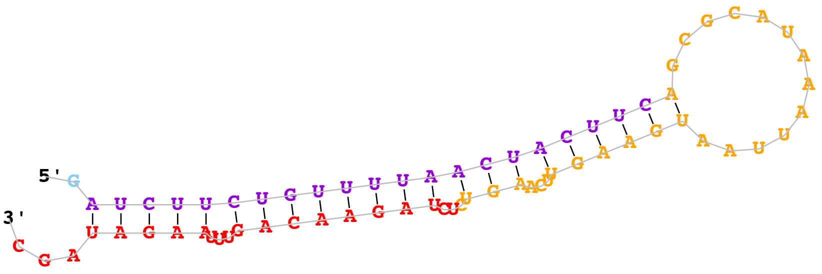

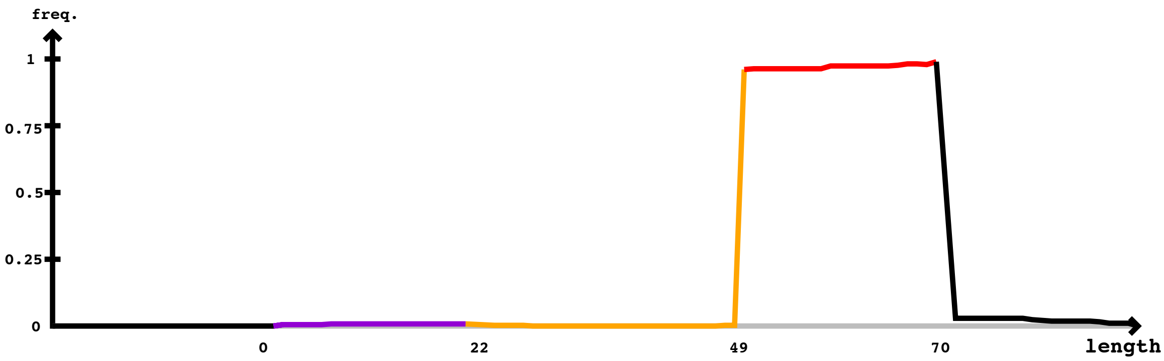

Star
Mature

```
5'- uaauccucuuucgaagauuaagaucuucuguuuuaacuacuucagcgcauaaaauuaaugaaguucaagucucuagaacaguuuaagauagcacaguucggaucauacaucg   -3'    obs
    uaauccucuuucgaagauuaagaucuucuguuuuaacuacuucagcgcauaaaauuaaugaaguucaagucucuagaacaguuuaagauagcacaguucggaucauacaucg          exp
    .........((((((.......((((((((((((((((((((((..............))))))...)))...))))))))...))))).......)))))).........    reads    mm    sample
    ......................aucuucuguuuuaacuacuuc.....................................................................    2        0     seq
    ...........................cuguuuuaacuacuucagcgc................................................................    1        0     seq
    .....................................................................ucucuagaacaguuuaagaua......................    1        0     seq
    .......................................................................ucuagaacaguuCaagauagc....................    3        1     seq
    .......................................................................ucuagaacaguuuaCgauagc....................    2        1     seq
    .......................................................................ucuagaacaguuuaagauagc....................    352      0     seq
    .......................................................................Gcuagaacaguuuaagauagc....................    1        1     seq
    .......................................................................ucuagaacaguuuaGgauagc....................    2        1     seq
    .......................................................................ucuagaacaguuuaUgauagc....................    5        1     seq
    ........................................................................cuagaacaguuuaagauagca...................    1        0     seq
    ................................................................................guuuaagauagcacaguucgg...........    1        0     seq
    ................................................................................guuuaagauagcacaguuUgg...........    1        1     seq
    ................................................................................guuuaagauagcacaguucggU..........    1        1     seq
    ................................................................................guuuaagauagcacaguucggCu.........    1        1     seq
    .......................................................................................auagcacaguucggaucauac....    1        0     seq
    ........................................................................................Nagcacaguucggaucauaca...    1        1     seq
    ........................................................................................uagcacaguucggaucauaca...    1        0     seq
    ...........................................................................................cacaguucggaucauacaucg    3        0     seq
    ...........................................................................................cacaguucggaucaAacaucg    1        1     seq
```

Provisional ID	:	ML2456_38734
Score total	:	167.1
Score for star read(s)	:	3.9
Score for read counts	:	167.3
Score for mfe	:	-4.8
Score for randfold	:	-2.2
Score for cons. seed	:	3
Total read count	:	340
Mature read count	:	336
Loop read count	:	0
Star read count	:	4

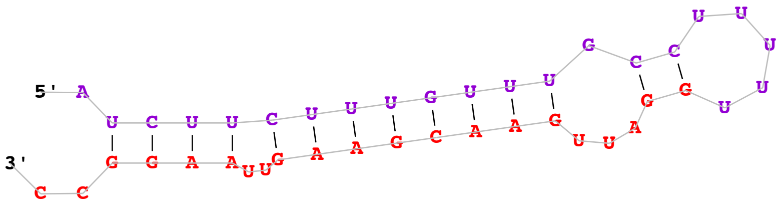

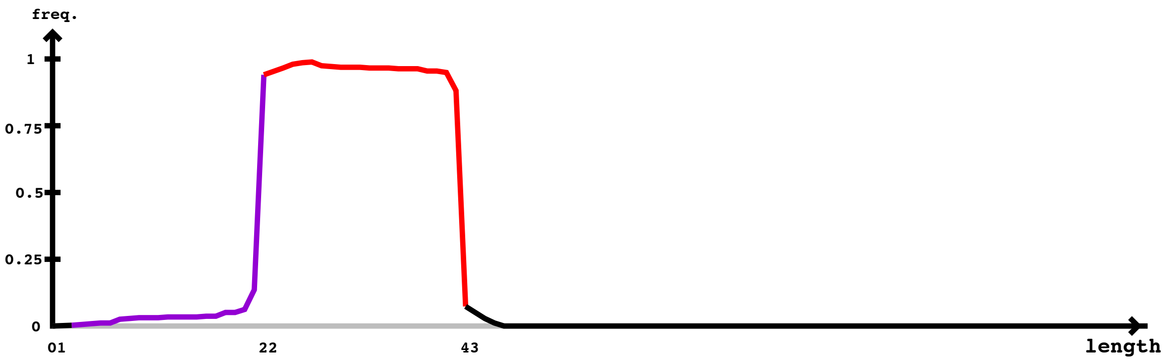

Star **Mature**

```
5'- aucuucuuuguuugccuuuuuggauugaacgaaguuaaggccucuucaaccuacaacuacaagacuagacaauguuugucugacuuugaccgacgcucauuugaagcgaau   -3'   obs
    aucuucuuuguuugccuuuuuggauugaacgaaguuaaggccucuucaaccuacaacuacaagacuagacaauguuugucugacuuugaccgacgcucauuugaagcgaau         exp
    .((((((((((((.((.....))...))))))))..)))).........................((((..((((((.....))))))..))))......(((((....).))))...   reads   mm   sample
    aucuucuuuguuugccuuuuu..........................................................................................   1   0   seq
    .ucuucuuuguuugccuuuuug.........................................................................................   1   0   seq
    ..cuucuuuguuugccuuuuugg........................................................................................   1   0   seq
    ...uucuuuguuugccuuuuugga.......................................................................................   1   0   seq
    .....cuuuguuugccuuuuuggauu.....................................................................................   5   0   seq
    ......uuuguuugccuuuuuggauug....................................................................................   1   0   seq
    .......uuguuugccuuuuuggauuga...................................................................................   1   0   seq
    ..........uuugccuuuuuggauugaacg................................................................................   1   0   seq
    ..............ccuuuuuggauugaacgaag.............................................................................   1   0   seq
    ................uuuuuggauugaacgaaguua..........................................................................   3   0   seq
    ................uuuuuggauugaacgaaguuaaggc......................................................................   1   0   seq
    ................uuuuuAgauugaacgaaguuaaggcc.....................................................................   1   1   seq
    ..................uuuggauugaacgaaguuaag........................................................................   2   0   seq
    ..................uuuggauugaacgaaguuaagg.......................................................................   1   0   seq
    ..................uuuggauugaacgaaguuaaggcc.....................................................................   1   0   seq
    ...................uuggauugaacgaaguuaagg.......................................................................   23   0   seq
    ...................uuggauugaacgaaguuaaggcc.....................................................................   2   0   seq
    ...................uuggauugaacgaaguuNaggcc.....................................................................   1   1   seq
    ....................Nggauugaacgaaguuaaggc......................................................................   1   1   seq
    ....................uggauugaaUgaaguuaaggc......................................................................   1   1   seq
    ....................uAgauugaacgaaguuaaggc......................................................................   16   1   seq
    ....................uggauugaacgaagNuaaggc......................................................................   1   1   seq
    ....................uggauugaacgaagGuaaggc......................................................................   2   1   seq
    ....................uggauugaacgaagAuaaggc......................................................................   3   1   seq
    ....................uggauugaacgaagCuaaggc......................................................................   1   1   seq
    ....................uggauugaacgaaguuaaggc......................................................................   260   0   seq
    .....................ggauugaacgaaguuaaggcc.....................................................................   8   0   seq
    ......................gauugaacgaaguuaaggccu....................................................................   3   0   seq
    .......................auugaacgaaguuaaggccuc...................................................................   6   0   seq
    ........................uugaacgaaguuaaggccucu..................................................................   3   0   seq
    .........................ugaacgaaguuaaggccucu..................................................................   1   0   seq
```

Provisional ID	:	ML0244_8266
Score total	:	133
Score for star read(s)	:	3.9
Score for read counts	:	127.1
Score for mfe	:	1.2
Score for randfold	:	-2.2
Score for cons. seed	:	3
Total read count	:	261
Mature read count	:	251
Loop read count	:	1
Star read count	:	9

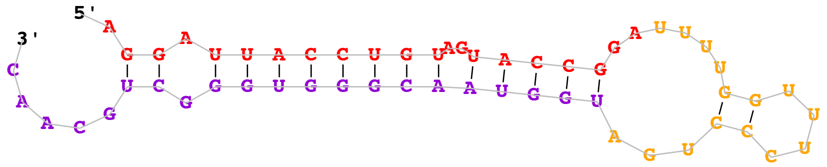

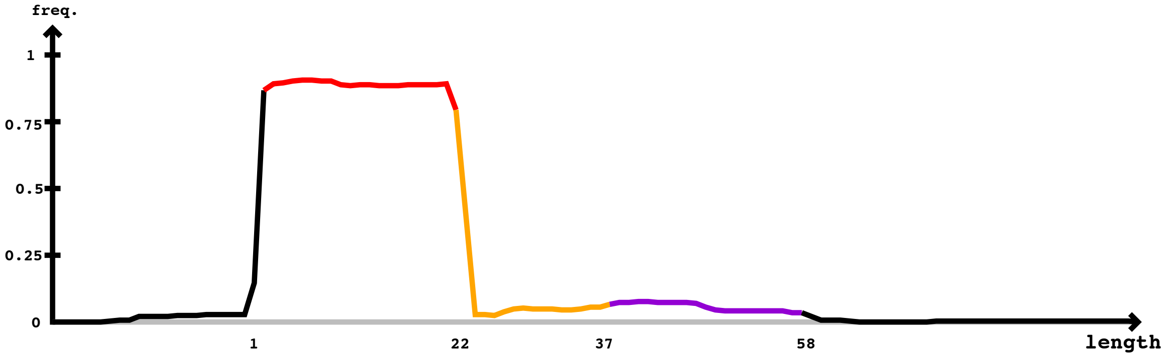


```
                        Mature                      Star
5'- gcuaugaaacguuucggggcaggauuaccuguaguaccggauuuugguuucccugaugguaacgggugggcugcaacuaggucagaaguugaaacaucaacuucaagcucc  -3'   obs
    gcuaugaaacguuucggggcaggauuaccuguaguaccggauuuugguuucccugaugguaacgggugggcugcaacuaggucagaaguugaaacaucaacuucaagcucc        exp
    (((............(..((((..((((((((..(((((......((....))...)))))))))))))).))))..).......((((((((....)))))))).)))...   reads   mm   sample
    ....ugaaacguuuAggggcaggauuacc..................................................................................   1       1    seq
    .....gaaacguuucggggcaggauu.....................................................................................   1       0    seq
    .......aacguuucggggcaggauuac...................................................................................   4       0    seq
    ...........uuuAggggcaggauuaccugu...............................................................................   1       1    seq
    ..............cggggcaggauuaccuguagu............................................................................   1       0    seq
    ...................caggauuaccuguaguaAcgg.......................................................................   3       1    seq
    ...................caggauuaccuguaguaccgg.......................................................................   25      0    seq
    ...................caggauuaccuguaguaccgga......................................................................   1       0    seq
    ...................caggauuaccuguaguaccggU......................................................................   5       1    seq
    ....................aggauuaccuguaguaccgga......................................................................   193     0    seq
    ....................aggauuaccuAuaguaccgga......................................................................   1       1    seq
    ....................aggauuaccuguaguaccggU......................................................................   2       1    seq
    ....................aAgauuaccuguaguaccgga......................................................................   2       1    seq
    ....................aggauuaccuguagCaccgga......................................................................   1       1    seq
    ....................aggauuaccuguagAaccgga......................................................................   2       1    seq
    ....................aggauuaccuguaguaAcgga......................................................................   3       1    seq
    ....................aCgauuaccuguaguaccgga......................................................................   1       1    seq
    ....................Nggauuaccuguaguaccgga......................................................................   2       1    seq
    .....................ggauuaccuguaguaccgga......................................................................   1       0    seq
    .....................ggauuaccCguaguaccggau.....................................................................   1       1    seq
    .....................ggauuaccuguaguaccggau.....................................................................   5       0    seq
    ......................gauuaccuguaguaccggauu....................................................................   1       0    seq
    .......................auuaccuguaguaccggauuu...................................................................   2       0    seq
    ........................uuaccuguaguaccggauuuuggC...............................................................   1       1    seq
    ..............................guaguaccggauuuugguuuc............................................................   1       0    seq
    ...................................accggauuuugguuucccuga.......................................................   2       0    seq
    .......................................gauuuugguuucccugauggua..................................................   1       0    seq
    ...........................................uuAguuucccugaugguaacg...............................................   1       1    seq
    ............................................ugguuucccugaugguaacgg..............................................   1       0    seq
    .............................................gguuucccugaugguaacggg.............................................   4       0    seq
    ..............................................guuucccugaugguaacgggu............................................   3       0    seq
    ...............................................uuucccugaugguaacgggug...........................................   1       0    seq
    .....................................................ugaugguaacgggugggcugcaac..................................   1       0    seq
```

```
                    Mature                           Star
gcuaugaaacguuucggggcaggauuaccuguaguaccggauuuugguuucccugaugguaacgggugggcugcaacuaggucagaaguugaaacaucaacuucaagcucc
......................................................gaugguaacgggugggcugca....................................    2    0    seq
........................................................ugguaacgggugggcugcaac..................................    5    0    seq
.........................................................gguaacgggugggcugcaacu.................................    2    0    seq
...........................................................uaacgggugggcugcaacuagU..............................    1    1    seq
................................................................ggugggcugcaacuaggC.............................    1    1    seq
..........................................................................................gaaacaucaacuucaagcucc    1    0    seq
```

Provisional ID	:	ML1218_26381
Score total	:	130
Score for star read(s)	:	3.9
Score for read counts	:	127.6
Score for mfe	:	1.3
Score for randfold	:	-2.2
Score for cons. seed	:	-0.6
Total read count	:	262
Mature read count	:	244
Loop read count	:	0
Star read count	:	18

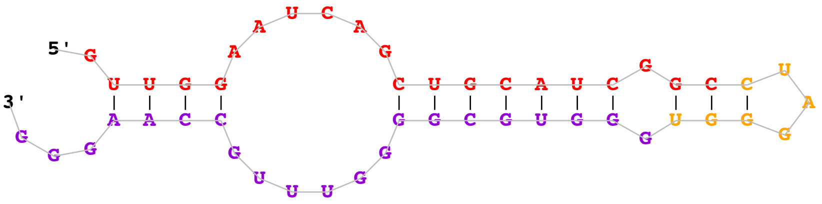

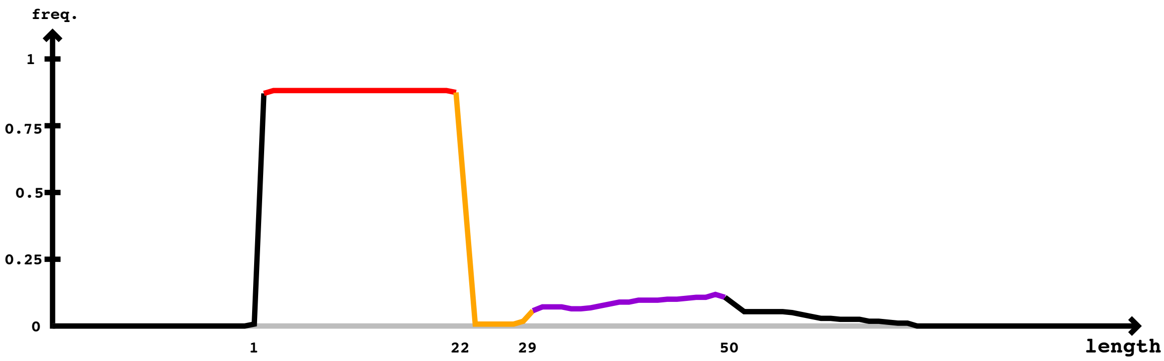

Mature **Star**

```
5'- uuggagcgagccucagccgcguuggaaucagcugcaucggccuagggugggugcgggguuugccaagggaguggaaggcaucauucagugugguagauucguacaggacacu  -3'   obs
    uuggagcgagccucagccgcguuggaaucagcugcaucggccuagggugggugcgggguuugccaagggaguggaaggcaucauucagugugguagauucguacaggacacu         exp
    ...(((.((((((...((((.((((......(((((((.(((...))).)))))))......))))....)))).)))).)).))).((((((......)))...)))).   reads   mm    sample
    ...................cguuggaaucagcugcaucgg........................................................................   2       0     seq
    ....................guuggaaucagcugcauAggc.......................................................................   40      1     seq
    ....................guuggaaucagcugUaucggc.......................................................................   1       1     seq
    ....................guuggaaucagcugcaucggc.......................................................................   197     0     seq
    ....................guuggaUucagcugcaucggc.......................................................................   1       1     seq
    ....................guuggaaucagcugGaucggc.......................................................................   2       1     seq
    .....................uuggaaucagcugcaucggc.......................................................................   1       0     seq
    .....................uuggaaucagcugcaucggccuagggugggu............................................................   1       0     seq
    .....................uuggaaucagcugcaucggccuagggGgggu............................................................   1       1     seq
    ...............................................ugggugUgggguuugccaagg............................................   3       1     seq
    ................................................gggugUgggguuugccaaggg...........................................   11      1     seq
    .................................................ggugUgggguuugccaaggga..........................................   4       1     seq
    ......................................................gAgguuugccaagggagugga.....................................   1       1     seq
    .......................................................ggguuugccaagAgaguggaa....................................   1       1     seq
    .......................................................ggguuugccaagggaguggaa....................................   1       0     seq
    ........................................................gguuugccaagggaguggaag...................................   2       0     seq
    .........................................................guuugccaagggaguggaagg..................................   1       0     seq
    .........................................................guuugccaagggaCuggaagg..................................   1       1     seq
    ...........................................................uugccaagggaguggaagAca................................   1       1     seq
    ...........................................................uugccaagggaguggaaggcauca.............................   1       0     seq
    ...............................................................ccaagggaguggaaggcauca............................   1       0     seq
    .................................................................aagggaguggaaggcaucauu..........................   1       0     seq
    ..................................................................agggaguggaaggcaucauuc.........................   1       0     seq
    ...................................................................ggaguggaaggcaucauucag........................   3       0     seq
```

Provisional ID	:	ML2170_36734
Score total	:	89.1
Score for star read(s)	:	3.9
Score for read counts	:	101.6
Score for mfe	:	-17.1
Score for randfold	:	-2.2
Score for cons. seed	:	3
Total read count	:	211
Mature read count	:	202
Loop read count	:	0
Star read count	:	9

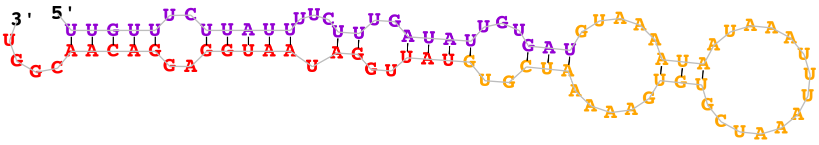

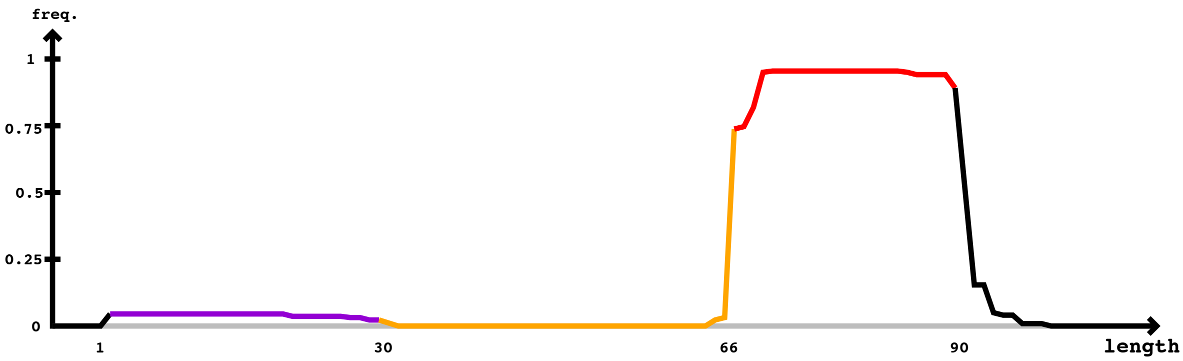

Star **Mature**

```
5'- uugauuguuucuuauuuucuuugauauugugauguaaaauaauaaaauuuaaaucgugugaaaaaucguguauuggauaauggaggacaacggucacaauuucccuguaaaauc  -3'   obs
    uugauuguuucuuauuuucuuugauauugugauguaaaauaauaaaauuuaaaucgugugaaaaaucguguauuggauaauggaggacaacggucacaauuucccuguaaaauc         exp
    .((((((((((((...((.((..((((...(((.....(((...............))).....)))..))))..)).))...)))).))))))))..................  reads   mm   sample
    ....uuguuucuuauuuucuuug...........................................................................................  2       0    seq
    ....uuguuucuuauuuucuuugauauug.....................................................................................  1       0    seq
    ....uuguuucuuauuuucuuugauauugug...................................................................................  1       0    seq
    ....uuguuucuuauuuucuuugauauuguU...................................................................................  1       1    seq
    ....uuguuucuuauuuucuuugauauugugau.................................................................................  4       0    seq
    ....uuguuucuuauuuucuuugauauugugauA................................................................................  1       1    seq
    ....................................................................uAuauuggauaauggaggacaacggu....................  5       1    seq
    .....................................................................Cuauuggauaauggaggacaacggu....................  1       1    seq
    .....................................................................Uuauuggauaauggaggacaacggu....................  1       1    seq
    ......................................................................uauuggauaauggaggac..........................  1       0    seq
    ......................................................................uauuggauaauggaggaca.........................  2       0    seq
    ......................................................................uauuggauaauggaggacaacgg.....................  5       0    seq
    ......................................................................uauuggauaauggaggacaacgC.....................  3       1    seq
    ......................................................................uauuggauaauggaUgacaacggu....................  1       1    seq
    ......................................................................uauuggauaauAgaggacaacggu....................  1       1    seq
    ......................................................................uauuggauaauggaggacaGcggu....................  1       1    seq
    ......................................................................uauuggauaauggaggacaacggu....................  90      0    seq
    ......................................................................uauuggauaauggaggacaacggC....................  16      1    seq
    ......................................................................Nauuggauaauggaggacaacggu....................  1       1    seq
    ......................................................................uauuggauaauggaggUcaacggu....................  1       1    seq
    ......................................................................uauuggauaauggaAgacaacggu....................  1       1    seq
    ......................................................................uauuggauaauggaggacaacgguA...................  2       1    seq
    ......................................................................uauuggauaauggaggacaGcgguc...................  3       1    seq
    ......................................................................uauuggauaauggaggacaacgguc...................  17      0    seq
    ......................................................................uauuggauaauggaggacaacgguU...................  1       1    seq
    ......................................................................uauuggauaauggaggacaacggGc...................  2       1    seq
    ......................................................................uauuggauaauggaggacaGcggucac.................  1       1    seq
    ......................................................................uauuggauaauggaggacaacggucac.................  7       0    seq
    .......................................................................auuggauaauggaggacaacggu....................  2       0    seq
    ........................................................................uuggauaauggaggacaacgg.....................  3       0    seq
    ........................................................................uuggauaauggaggacaacggu....................  3       0    seq
    ........................................................................uuggauaauggaggacaacggC....................  1       1    seq
    ........................................................................uuggauaauggaggacaacgguc...................  3       0    seq
```

```
    Star                                                            Mature
uugauuguuucuuauuuucuuugauauugugauguaaaauaauaaauuuaaaucgugugaaaaaucguguauuggauaauggaggacaacggucacaauuucccuguaaaauc
.......................................................................uuggauaauggaggacaacggucac.................    5    0    seq
.......................................................................uuggauaauggaggacaacggucacC................    1    1    seq
........................................................................uggauaauggaggacaacggC....................    4    1    seq
........................................................................uggauaauggaggacaacggu....................    5    0    seq
........................................................................uggauaauggaggacaacgguAac.................    1    1    seq
........................................................................uggauaauggaggacaacggGcac.................    2    1    seq
........................................................................uggauaauggaggacaacgguUac.................    1    1    seq
........................................................................uggauaauggaggacaacggucac.................    6    0    seq
........................................................................uggauaauggaggacaacggucacC................    1    1    seq
........................................................................uggauaauggaggacaacggucacaau..............    3    0    seq
........................................................................uggauaauggaggacaacggucacaaC..............    4    1    seq
........................................................................uggauaauggaggacaacggucacaauuCc...........    2    1    seq
.........................................................................Agauaauggaggacaacgguc...................    1    1    seq
```

Provisional ID : ML2170_36733
Score total : 86.4
Score for star read(s) : 3.9
Score for read counts : 99
Score for mfe : -17.2
Score for randfold : -2.2
Score for cons. seed : 3
Total read count : 206
Mature read count : 202
Loop read count : 3
Star read count : 1

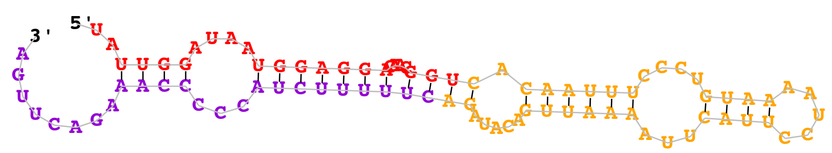

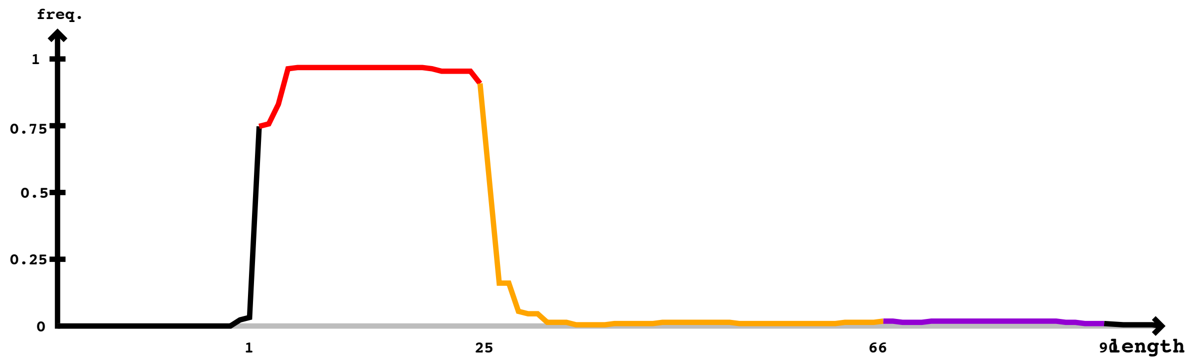


```
                    Mature                                                         Star
5'- aaucgugugaaaaaucguguauuggauaaauggaggacaacggucacaauuucccuguaaaaauccuuacuuaaaaauugacauagacuuuuucuacccccaaagacuugaucuac  -3'   obs
    aaucgugugaaaaaucguguauuggauaaauggaggacaacggucacaauuucccuguaaaaauccuuacuuaaaaauugacauagacuuuuucuacccccaaagacuugaucuac        exp
    ............((((.((.((((....(((((((....((((.((((((....((((.....))))...))))))).....)))))))))))...))))..)).))))....   reads   mm   sample
    .................uAuauuggauaaauggaggacaacggu........................................................................   5   1   seq
    ..................Cuauuggauaaauggaggacaacggu........................................................................   1   1   seq
    ..................Uuauuggauaaauggaggacaacggu........................................................................   1   1   seq
    ...................uauuggauaaauggaggac..............................................................................   1   0   seq
    ...................uauuggauaaauggaggaca.............................................................................   2   0   seq
    ...................uauuggauaaauggaggacaacgg.........................................................................   5   0   seq
    ...................uauuggauaaauggaggacaacgC.........................................................................   3   1   seq
    ...................uauuggauaaauggaggacaGcggu........................................................................   1   1   seq
    ...................uauuggauaaauggaggacaacggC........................................................................   16   1   seq
    ...................uauuggauaaauggaUgacaacggu........................................................................   1   1   seq
    ...................uauuggauaaauggaAgacaacggu........................................................................   1   1   seq
    ...................uauuggauaaauAgaggacaacggu........................................................................   1   1   seq
    ...................uauuggauaaauggaggacaacggu........................................................................   90   0   seq
    ...................uauuggauaaauggaggUcaacggu........................................................................   1   1   seq
    ...................Nauuggauaaauggaggacaacggu........................................................................   1   1   seq
    ...................uauuggauaaauggaggacaacgguU.......................................................................   1   1   seq
    ...................uauuggauaaauggaggacaacggGc.......................................................................   2   1   seq
    ...................uauuggauaaauggaggacaacgguc.......................................................................   17   0   seq
    ...................uauuggauaaauggaggacaacgguA.......................................................................   2   1   seq
    ...................uauuggauaaauggaggacaGcgguc.......................................................................   3   1   seq
    ...................uauuggauaaauggaggacaGcggucac.....................................................................   1   1   seq
    ...................uauuggauaaauggaggacaacggucac.....................................................................   7   0   seq
    ....................auuggauaaauggaggacaacggu........................................................................   2   0   seq
    .....................uuggauaaauggaggacaacgg.........................................................................   3   0   seq
    .....................uuggauaaauggaggacaacggC........................................................................   1   1   seq
    .....................uuggauaaauggaggacaacggu........................................................................   3   0   seq
    .....................uuggauaaauggaggacaacgguc.......................................................................   3   0   seq
    .....................uuggauaaauggaggacaacggucac.....................................................................   5   0   seq
    .....................uuggauaaauggaggacaacggucacC....................................................................   1   1   seq
    ......................uggauaaauggaggacaacggu........................................................................   5   0   seq
    ......................uggauaaauggaggacaacggC........................................................................   4   1   seq
    ......................uggauaaauggaggacaacggucac.....................................................................   6   0   seq
    ......................uggauaaauggaggacaacgguUac.....................................................................   1   1   seq
```

	Mature	Star			

```
                   Mature                                                          Star
aaucgugugaaaaaucguguauuggauaaauggaggacaacggucacaauuucccuguaaaauccuuacuuaaaaauugacauagacuuuuucuacccccaaagacuugaucuac
......................uggauaaauggaggacaacggGcac....................................................................   2   1   seq
......................uggauaaauggaggacaacgguAac....................................................................   1   1   seq
......................uggauaaauggaggacaacggucacC...................................................................   1   1   seq
......................uggauaaauggaggacaacggucacaaC.................................................................   4   1   seq
......................uggauaaauggaggacaacggucacaau.................................................................   3   0   seq
......................uggauaaauggaggacaacggucacaauuCc..............................................................   2   1   seq
.......................Agauaaauggaggacaacgguc......................................................................   1   1   seq
...........................................ucacaauuuccUuguaaaauccuuacu.............................................   1   1   seq
.........................................................uaaaauccuuacuuUaaaauugaca.................................   1   1   seq
..............................................................uccuuacuuaaaaauugacauagacC...........................   1   1   seq
..................................................................................uagacuuuuucuacccccaaagC..........   1   1   seq
..................................................................................uagacuuuuucuacccccaaagacu........   1   0   seq
......................................................................................cuuuuucuacccccaaagacuugC.....   1   1   seq
...........................................................................................ucuacccccaaagacuugaucuac   1   0   seq
```

Provisional ID	:	ML2798_40702
Score total	:	76.2
Score for star read(s)	:	3.9
Score for read counts	:	76.6
Score for mfe	:	-5
Score for randfold	:	-2.2
Score for cons. seed	:	3
Total read count	:	162
Mature read count	:	143
Loop read count	:	13
Star read count	:	6

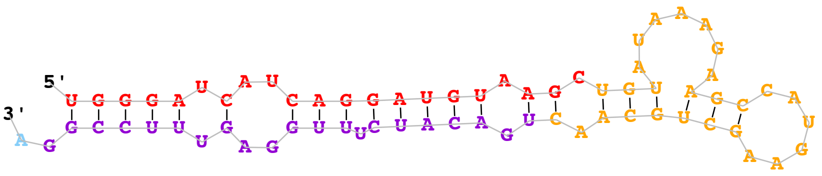

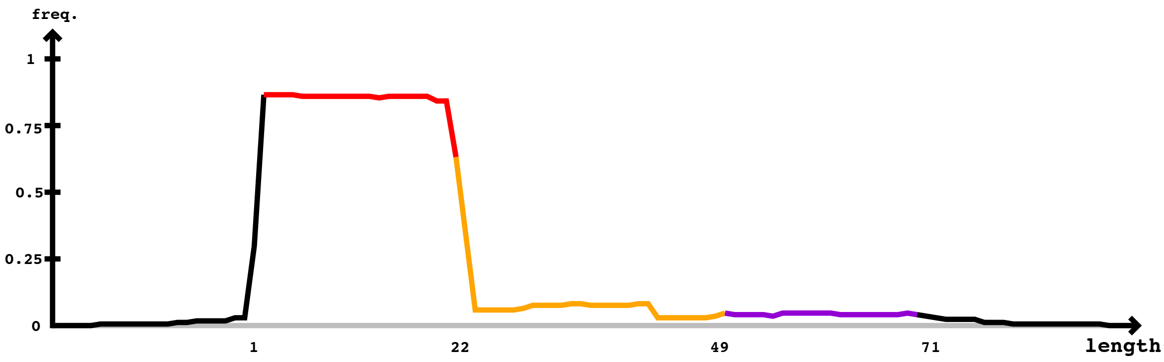


```
                        Mature                                      Star
5'- gcaaauaagaauaaucgugaugggaucaucaggauguaagcuguauaaagaagccaugaagcugcaacugacaucuuuggaguuuccggacugaguuuucgaaacacuucc  -3'   obs
    gcaaauaagaauaaucgugaugggaucaucaggauguaagcuguauaaagaagccaugaagcugcaacugacaucuuuggaguuuccggacugaguuuucgaaacacuucc         exp
    ........(((...(((...(((((.(..((((((((.((.(((.......(((......)))))).)).))))).)))..).)))))...)))...))).........    reads   mm   sample
    ...aauaagaauaaucgugauggg.......................................................................................    1       0    seq
    ...........uaaucgugaugggaucauUag...............................................................................    1       1    seq
    .............aucgugaugggaucauUaggauguaagc......................................................................    1       1    seq
    .................ugaugggaucauUaggaugua.........................................................................    2       1    seq
    ...................augggaucauUaggauguaag.......................................................................    32      1    seq
    ...................augggaucaucaggauguaag.......................................................................    13      0    seq
    ...................augggaucauUaggauguaagc......................................................................    1       1    seq
    ....................ugggaucauUaggaugua.........................................................................    1       1    seq
    ....................ugggaucaucaggauguaagc......................................................................    65      0    seq
    ....................ugggaucaucaggaCguaagc......................................................................    1       1    seq
    ....................ugggaucauUaggauguaagc......................................................................    30      1    seq
    .................................auguaagcuguauaaagaagc.........................................................    1       0    seq
    ........................................cuguauaaagaagccaugaag..................................................    9       0    seq
    ...............................................aagaagccaugaagcugcaac...........................................    1       0    seq
    ................................................agaagccaugaagcugcaac...........................................    1       0    seq
    ................................................agaagccaugaagcugcaacu..........................................    1       0    seq
    ....................................................gccaugaagcuAcaacugaca......................................    1       1    seq
    ...........................................................agcugcaacugacaucuuugg...............................    1       0    seq
    ...................................................................cugacaucuuuggaguuuccg.......................    1       0    seq
    ....................................................................ugacaucuuuggaguuuccgg......................    3       0    seq
    ....................................................................ugacaucuuuggaguuuccggacugaguuu.............    1       0    seq
    ..........................................................................cuuuggaguuuccggacugag................    2       0    seq
    .......................................................................................ggacugaguuuucgaaacacu...    1       0    seq
```

Provisional ID	:	ML3007_41813
Score total	:	52.6
Score for star read(s)	:	3.9
Score for read counts	:	53.1
Score for mfe	:	-5.2
Score for randfold	:	-2.2
Score for cons. seed	:	3
Total read count	:	116
Mature read count	:	104
Loop read count	:	0
Star read count	:	12

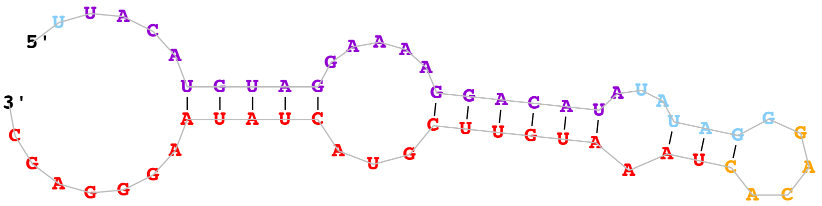

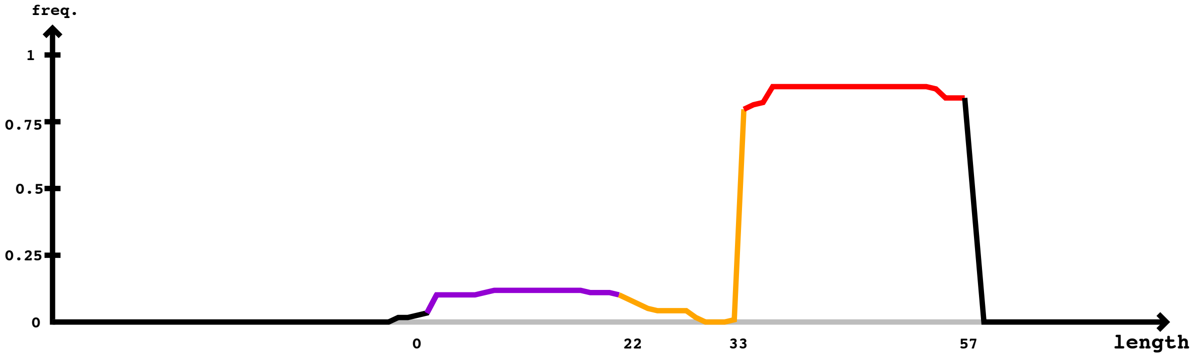

Star **Mature**

```
5'- aguccaacacaucaggaaaggaaaaguacaggauugguuacauguaggaaaaggacauauauagggacacuaaaauguucguacuauaagggagcaguguaagagggcugagcuu  -3'   obs
    aguccaacacaucaggaaaggaaaaguacaggauugguuacauguaggaaaaggacauauauagggacacuaaaauguucguacuauaagggagcaguguaagagggcugagcuu        exp
    (((((......((.......))........(.((((.(..(.(((((.....((((((...(((.....))).))))))...))))).)..).)))).)....)))))......  reads   mm   sample
    ..................................ugguuacauguaggaaaaggaca..........................................................  1       0    seq
    ..................................ugguuacauguaggaaaaggacaua........................................................  1       0    seq
    ....................................guuacauguaggaaaagg.............................................................  1       0    seq
    .....................................uuacauguaggaaaaggacauauauaggC.................................................  1       1    seq
    ......................................uacauguaggaaaaggacaua........................................................  5       0    seq
    ......................................uacauguaggaaaaggacauauauagC..................................................  2       1    seq
    ......................................uacauguaggaaaaggacauauauaggC.................................................  1       1    seq
    ...........................................guaggaaaaggacauaua......................................................  1       0    seq
    ............................................uaggaaaaggacauauauagg..................................................  1       0    seq
    .....................................................................cuaaaauguucguacuauaagg........................  1       0    seq
    ......................................................................uaaaauguucguacuauaaggg.......................  3       0    seq
    ......................................................................uaaaauguucguacuUuaaggg.......................  1       1    seq
    ......................................................................uaaaauguucguacuauaaAggagc....................  1       1    seq
    ......................................................................uaaaauguucguacuauaagggagc....................  83      0    seq
    ......................................................................uaaaauguucguacuauaaggAagc....................  2       1    seq
    ......................................................................uaaaauguucguacuauGagggagc....................  1       1    seq
    ......................................................................uaaaauguucguacuUuaagggagc....................  1       1    seq
    ......................................................................uaaaauguucguacuauaagAgagca...................  1       1    seq
    ........................................................................aaauguucguacuauaagggagc....................  2       0    seq
    .........................................................................aauguucguacuauaagggagc....................  1       0    seq
    ..........................................................................auguucguacuauaagggagc....................  7       0    seq
```

Provisional ID	:	ML3973_45281
Score total	:	38.5
Score for star read(s)	:	3.9
Score for read counts	:	28.2
Score for mfe	:	1.8
Score for randfold	:	1.6
Score for cons. seed	:	3
Total read count	:	67
Mature read count	:	65
Loop read count	:	0
Star read count	:	2

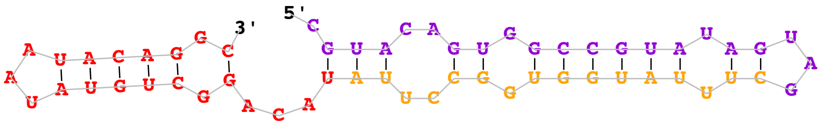

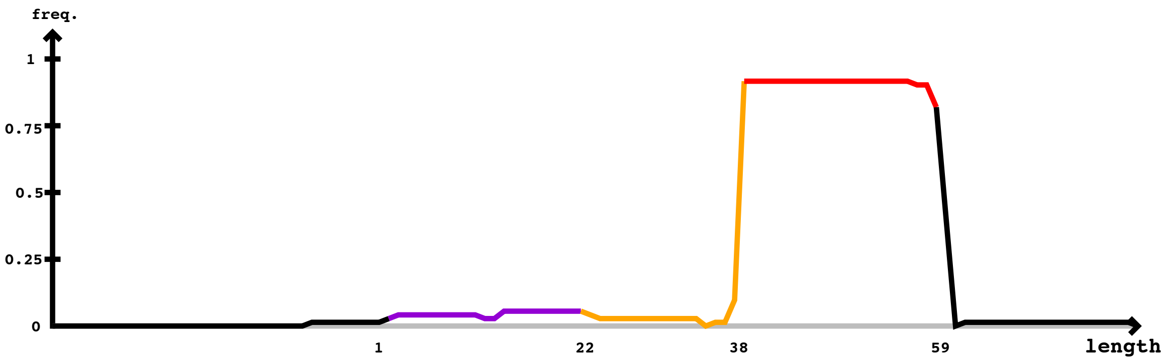


```
                                  Star                                 Mature
5'- aauacaagcuguauaauacaggcuuauauaggccguacaguggccguauaguagcuuuaugguggccuuauacaggcuguauaauacaggcuuauauaggccguacagugg   -3'     obs
    aauacaagcuguauaauacaggcuuauauaggccguacaguggccguauaguagcuuuaugguggccuuauacaggcuguauaauacaggcuuauauaggccguacagugg           exp
    .......(((((((......(((((((((((((((((..(..(((((((((..(((.......))).)))))).)))..)....))).)))))))))))))))))))))..   reads   mm   sample
    .........................auauaggccguacagugg....................................................................   2       0    seq
    .................................cguacaguggccguauaguag.........................................................   1       0    seq
    ..................................guacaguggccguauaguagc........................................................   1       0    seq
    .............................................guauaguagcuuuaugguggc.............................................   1       0    seq
    .............................................guauaguagcuuuGugguggc.............................................   1       1    seq
    ...................................................................uuaAacaggcuguauaauaca.......................   1       1    seq
    .....................................................................auacaggcuguauaauaAagg.....................   4       1    seq
    .....................................................................auacaggcuguauaauacagg.....................   2       0    seq
    ......................................................................uacaggcuguauaauUcaggc....................   1       1    seq
    ......................................................................uacaggcuguauaauaAaggc....................   14      1    seq
    ......................................................................uacaggcuguaAaauacaggc....................   2       1    seq
    ......................................................................uacaggcuguauaaAacaggc....................   3       1    seq
    ......................................................................uacaggcuguauaauacaggc....................   38      0    seq
    ......................................................................uacaggcuguUuaauacaggc....................   1       1    seq
    .............................................................................................auauaggccguacagugg   2       0    seq
```

Provisional ID	:	ML0604_16230
Score total	:	29.7
Score for star read(s)	:	3.9
Score for read counts	:	28.7
Score for mfe	:	0
Score for randfold	:	-2.2
Score for cons. seed	:	-0.6
Total read count	:	68
Mature read count	:	66
Loop read count	:	0
Star read count	:	2

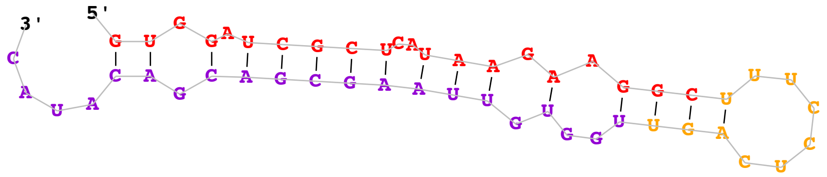

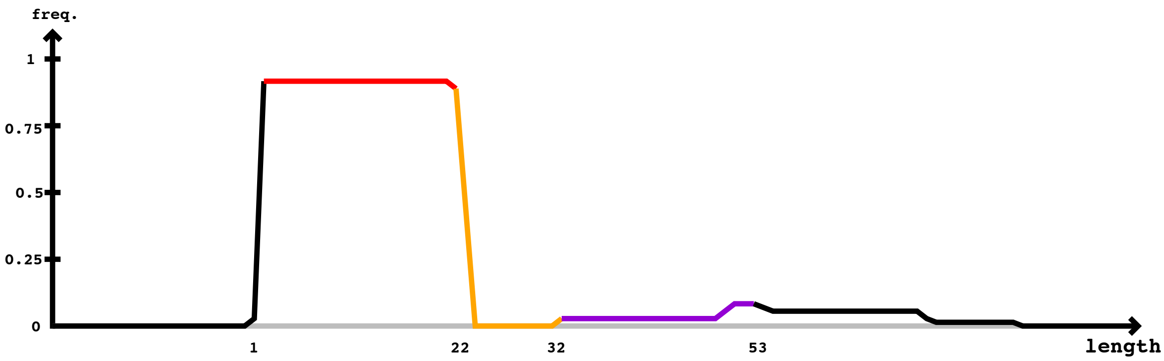

Mature Star

```
5'- gugauggguugggguaggggguggaucgcucauaagaaggcuuuccucaguugguguuaagcgacgacauacuugccugacuuggucguuauauucaucaucagagucggg   -3'    obs
    gugauggguugggguaggggguggaucgcucauaagaaggcuuuccucaguugguguuaagcgacgacauacuugccugacuuggucguuauauucaucaucagagucggg          exp
    (((((((((..(((((((..((.(.(((((..(((.(.((((......))))..).))))))))).))...)))))))(((...))).....)))))))))..........   reads   mm   sample
    ...................gguggaucgcucauaagaagg.......................................................................   2       0    seq
    ....................Nuggaucgcucauaagaaggc......................................................................   1       1    seq
    ....................guggaucgcucauaagaaggc......................................................................   63      0    seq
    ...................................................ugguguuaagcgacgacauac.......................................   1       0    seq
    ...................................................Ggguguuaagcgacgacauac.......................................   1       1    seq
    ....................................................................auacuugccugacuuggucgu......................   2       0    seq
    .....................................................................uacuugccugacuuggucguu.....................   1       0    seq
    .....................................................................uacuugccugacuuggucguuauauucauc............   1       0    seq
```

Provisional ID	:	ML0711_18222
Score total	:	28.9
Score for star read(s)	:	3.9
Score for read counts	:	27.1
Score for mfe	:	0.6
Score for randfold	:	-2.2
Score for cons. seed	:	-0.6
Total read count	:	65
Mature read count	:	58
Loop read count	:	5
Star read count	:	2

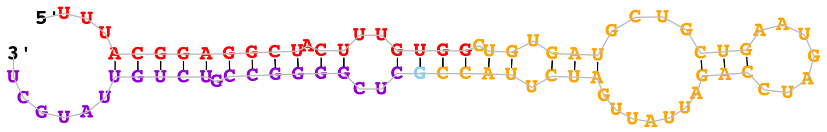

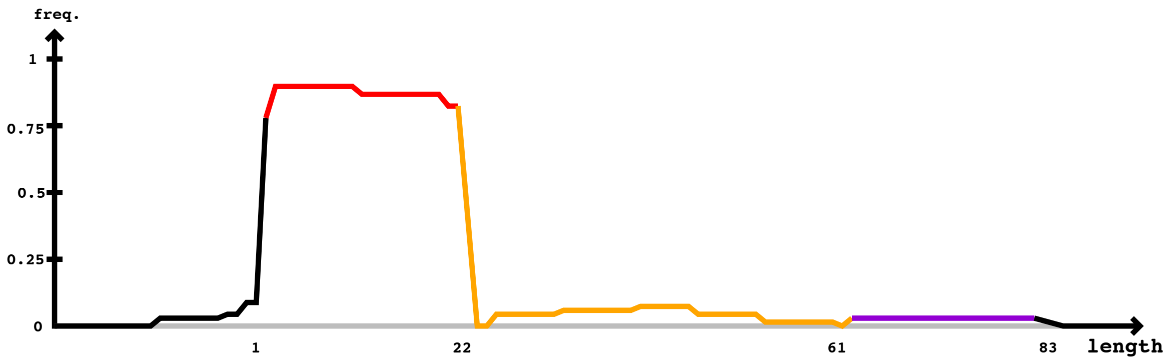


```
                       Mature                                                          Star
5'- cuuuacauauagucaagcaguuuacggaggcuacuuuguggcugugaugcugcugaaugauccagauuauugaucuuaccgcucggggccgucuguuaugcuugcgguggu   -3'    obs
    cuuuacauauagucaagcaguuuacggaggcuacuuuguggcugugaugcugcugaaugauccagauuauugaucuuaccgcucggggccgucuguuaugcuugcgguggu          exp
    .....(((...(.((((((....((((((((.((..((((.((.(((....(((.......))).......))).))))))..)))))).)))))..)))))))).)))..   reads   mm   sample
    .........Cagucaagcaguuuacggagg.................................................................................   2       1    seq
    ................gcaguuuacggaggcuacuuuguggc.....................................................................   1       0    seq
    .................aguuuacggaggcuacuuugu.........................................................................   3       0    seq
    ....................uuuacggaggcuacCuugugg......................................................................   1       1    seq
    ....................uuuaUggaggcuacuuugugg......................................................................   1       1    seq
    ....................uuuacggaggcuacuuugugg......................................................................   43      0    seq
    ....................Nuuacggaggcuacuuugugg......................................................................   1       1    seq
    ....................uuuacggaggAuacuuugugg......................................................................   1       1    seq
    .....................uuacggaggcAacuuuguggc.....................................................................   1       1    seq
    .....................uuacggaggcuacuuuguggc.....................................................................   7       0    seq
    ..........................................ugaugcugcugaaugauccag................................................   2       0    seq
    ..........................................ugaugcugcugaaugauccagauuauug.........................................   1       0    seq
    ...............................................gcugaaugauccagauuauug..........................................   1       0    seq
    .....................................................auccagauuauugaucuuacc.....................................   1       0    seq
    ..................................................................................cucAgggccgucuguuaugcu.........   2       1    seq
```

Provisional ID : ML1242_26756
Score total : 27.4
Score for star read(s) : 3.9
Score for read counts : 25.1
Score for mfe : -2.5
Score for randfold : 1.6
Score for cons. seed : -0.6
Total read count : 61
Mature read count : 44
Loop read count : 16
Star read count : 1

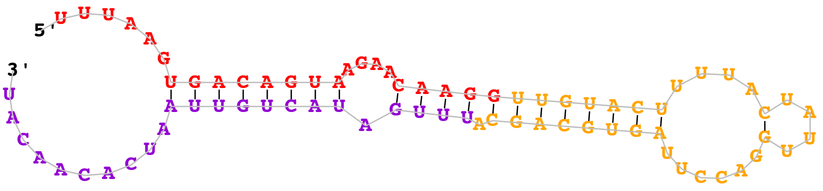

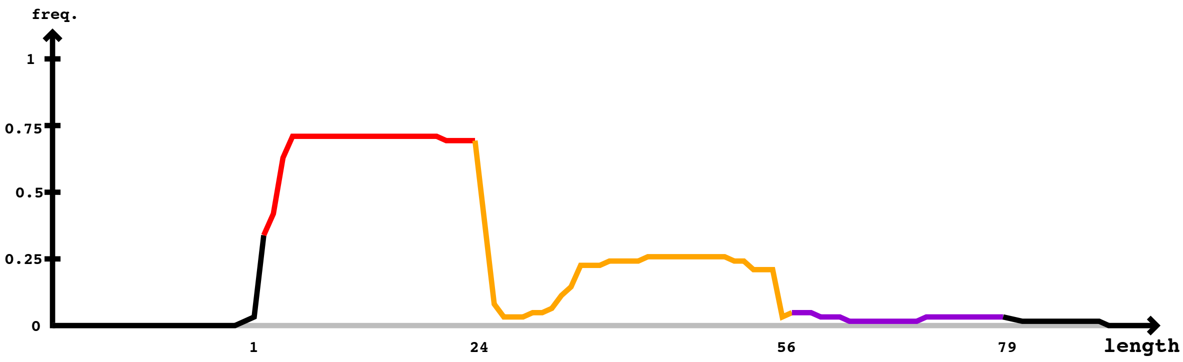


```
                          Mature                                             Star
5'- aucuacaaugacuuauuauguuuaagugacaguaagaacaagguuguacuuuuacuauuggaccuuagugcagcauuugauacuguuaaucacaacauacuuaucucgaaacu  -3'   obs
    aucuacaaugacuuauuauguuuaagugacaguaagaacaagguuguacuuuuacuauuggaccuuagugcagcauuugauacuguuaaucacaacauacuuaucucgaaacu        exp
    ........(((..((.((((((....((((((((....((((((((((((....(....)......)))))))).)))).)))))))).....))))))..))..))).....   reads   mm   sample
    ..................uguuuaagugacaguaagaac..........................................................................   1       0    seq
    ...................guuuaagugacaguaagaacaaggu.....................................................................   1       0    seq
    ....................uuuaagugacaguaagaacaagg......................................................................   17      0    seq
    ....................uuuaagugacaguaagaacaaggu.....................................................................   2       0    seq
    .....................uuaagugacaguaagaacaaggu.....................................................................   5       0    seq
    ......................uaagugacaguaagaacaagg......................................................................   9       0    seq
    ......................uaagugacaguaagUacaaggu.....................................................................   1       1    seq
    ......................uaagugacaguaagaacaagguu....................................................................   1       0    seq
    ......................uUagugacaguaagaacaagguu....................................................................   2       1    seq
    .......................aagugacaguaagaacaaggu.....................................................................   5       0    seq
    ...........................................uuguacuuuuacuauuggaccuuagugc..........................................   1       0    seq
    ...........................................uuguacuuuuacuauuggaccuuagugcagc.......................................   1       0    seq
    ................................................cuuuuacuauuggaccuuagu............................................   1       0    seq
    ..................................................uuuacuauuggaccuuagugcagcauuu...................................   1       0    seq
    ...................................................uuacuauuggaccuuagGgcagc.......................................   1       1    seq
    ...................................................uuacuauuggaccuuagugcagc.......................................   2       0    seq
    ....................................................uacuauuggaccuuagugcagc.......................................   1       0    seq
    ....................................................uacAauuggaccuuagugcagc.......................................   1       1    seq
    .....................................................acuauuggaccuuagugc..........................................   1       0    seq
    .....................................................acuauuggaccuuagugcagc.......................................   3       0    seq
    .....................................................Ucuauuggaccuuagugcagc.......................................   1       1    seq
    ........................................................auuggaccuuagugcagc.......................................   1       0    seq
    .............................................................gaccuuagugcagcauuugau...............................   1       0    seq
    ...........................................................................uuugauacuguGaaucacaacau...............   1       1    seq
    .........................................................................................ucacaacauacuuaucucg.....   1       0    seq
```

Provisional ID	:	ML0157_6135
Score total	:	23.8
Score for star read(s)	:	3.9
Score for read counts	:	20.5
Score for mfe	:	-1.3
Score for randfold	:	-2.2
Score for cons. seed	:	3
Total read count	:	52
Mature read count	:	40
Loop read count	:	11
Star read count	:	1

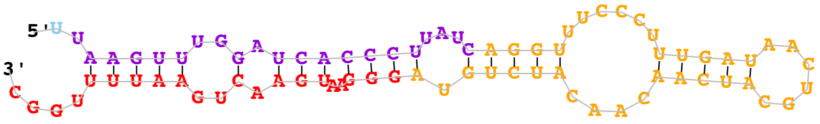

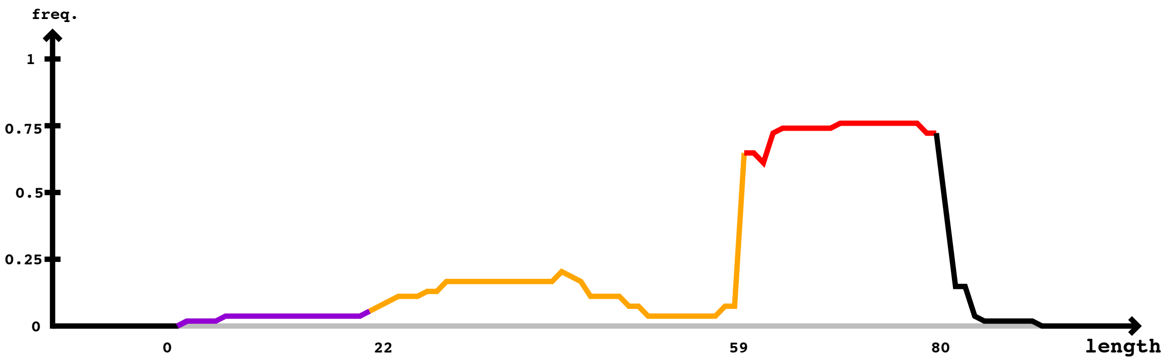


```
             Star                                                        Mature
5'- gucuuucugguuuaaguuuggaucacccuuaucagguuucccuuugauaacugcaucaacaacaucuguagggaaugaacugaauuuuggcgguugaaaugacccaaugua   -3'    obs
    gucuuucugguuuaaguuuggaucacccuuaucagguuucccuuugauaacugcaucaacaacaucuguagggaaugaacugaauuuuggcgguugaaaugacccaaugua          exp
    ((((((((.((..(((((..(.(((((((...(((((......(((((......)))))....))))).))))..))).)..)))))..)).)..)))).)))........    reads     mm     sample
    ............uaaguuuggaucacccuuauc..............................................................................    1         0      seq
    ................uuuggaucacccuuaucaggu..........................................................................    1         0      seq
    ...............................ucagguuucccuuugauaacu...........................................................    1         0      seq
    ................................cagguuucccuuugauaacug..........................................................    1         0      seq
    .................................agguuucccuuugauaacugc.........................................................    3         0      seq
    .....................................uucccuuugauaacugcauca.....................................................    2         0      seq
    .......................................cccuuugauaacugcaucaac...................................................    2         0      seq
    ...................................................ugcaucaacaacaucuguagg.......................................    2         0      seq
    ....................................................................uagggaUugaacugaauuuug......................    2         1      seq
    ......................................................................gggaaugaacugaauuuuggc....................    31        0      seq
    .........................................................................aaugaacugaauuuuggcggu.................    6         0      seq
    ..........................................................................augaacugaauuuuggcgguu................    1         0      seq
    ................................................................................ugaauuuuggcgguugaaaug..........    1         0      seq
```

Provisional ID	:	ML0467_13355
Score total	:	10.8
Score for star read(s)	:	3.9
Score for read counts	:	10.8
Score for mfe	:	-1
Score for randfold	:	-2.2
Score for cons. seed	:	-0.6
Total read count	:	33
Mature read count	:	32
Loop read count	:	0
Star read count	:	1

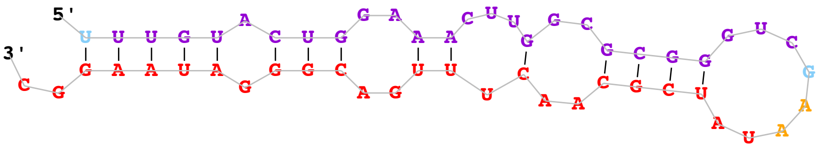

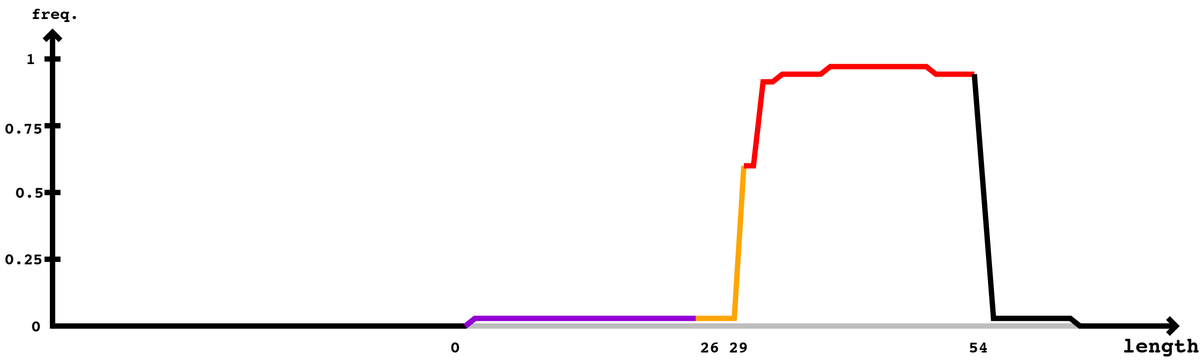

Star Mature

```
5'- auaucacaaagauuuaaaacuuggcagccgaaaagggaggcuuuguacuggaaacuuggcgcgggucgaauaucgcaacuuugacgggauaaggcuguugugaaggauuggaagu   -3'    obs
    auaucacaaagauuuaaaacuuggcagccgaaaagggaggcuuuguacuggaaacuuggcgcgggucgaauaucgcaacuuugacgggauaaggcuguugugaaggauuggaagu          exp
    ............(((((..(((.(((((..........((((((((.(((..((...(..((((........))))..).))..))).))))))))))))).)))..)))))...   reads   mm   sample
    ..........................................uuguacuggaaacuuggcgcgggucg................................................   1       0    seq
    ..................................................................Ugaauaucgcaacuuugacgggau.........................   1       1    seq
    ......................................................................uaucgcaGcuuugacgggauaaggc....................   1       1    seq
    ......................................................................uaucgcaacuuugaAgggauaaggc....................   1       1    seq
    ......................................................................uaucgcaacuuugacgggauaaggc....................   18      0    seq
    ........................................................................Gcgcaacuuugacgggauaaggc....................   1       1    seq
    ........................................................................ucgcaacuuugacgCgauaaggc....................   1       1    seq
    ........................................................................ucgcaacuuugacgggauaaggc....................   9       0    seq
    ..........................................................................gcaacuuugacgggauaaggc....................   1       0    seq
    ...............................................................................uuugacgggauaaggcuguugugaag..........   1       0    seq
```
