# Supplementary material for: MicroRNAs and essential components of the microRNA processing machinery are not encoded in the genome of the ctenophore Mnemiopsis leidyi
Source: BMC Genomics. 2012 Dec 20;13:714. doi: 10.1186/1471-2164-13-714 (PMC3563456; doi:10.1186/1471-2164-13-714)
Supplement: Additional file 3 — Dataset 2. contains a folder of output data files in plain text format related to the miRNA predictions (both canonical and mirtron) produced by the various programs described in the Methods. [file 1471-2164-13-714-S3.zip › Additional_Dataset_2/e_s2n_sample1_miRDeep2.html]

miRDeep2


| **miRDeep2** |  |

**Survey of miRDeep2 performance for score cut-offs -10 to 10**


miRDeep2 scorefor details on how the log-odds score is calculated, see Friedlander et al., Nature Biotechnology, 2008. | estimated signal-to-noisefor the given score cut-off, the signal-to-noise ratio is estimated as r = total miRNA hairpins reported / mean estimated false positive miRNA hairpins over 100 rounds of permuted controls. | excision gearingthis is the minimum read stack height required for excising a potential miRNA precursor from the genome in this analysis. || 10 0.7 5 | | |
| 9 0.7 5 | | |
| 8 0.7 5 | | |
| 7 0.6 5 | | |
| 6 0.6 5 | | |
| 5 1.1 5 | | |
| 4 1.6 5 | | |
| 3 1.2 5 | | |
| 2 0.9 5 | | |
| 1 0.8 5 | | |
| 0 0.7 5 | | |
| -1 0.6 5 | | |
| -2 0.5 5 | | |
| -3 0.5 5 | | |
| -4 0.6 5 | | |
| -5 0.7 5 | | |
| -6 0.7 5 | | |
| -7 0.7 5 | | |
| -8 0.8 5 | | |
| -9 0.8 5 | | |
| -10 0.8 5 | | |

  
  
