# Supplementary material for: MicroRNAs and essential components of the microRNA processing machinery are not encoded in the genome of the ctenophore Mnemiopsis leidyi
Source: BMC Genomics. 2012 Dec 20;13:714. doi: 10.1186/1471-2164-13-714 (PMC3563456; doi:10.1186/1471-2164-13-714)
Supplement: Additional file 4 — Figure S2. provides the prediction score histograms produced by the mirtron prediction method used [51]. [file 1471-2164-13-714-S4.pdf]

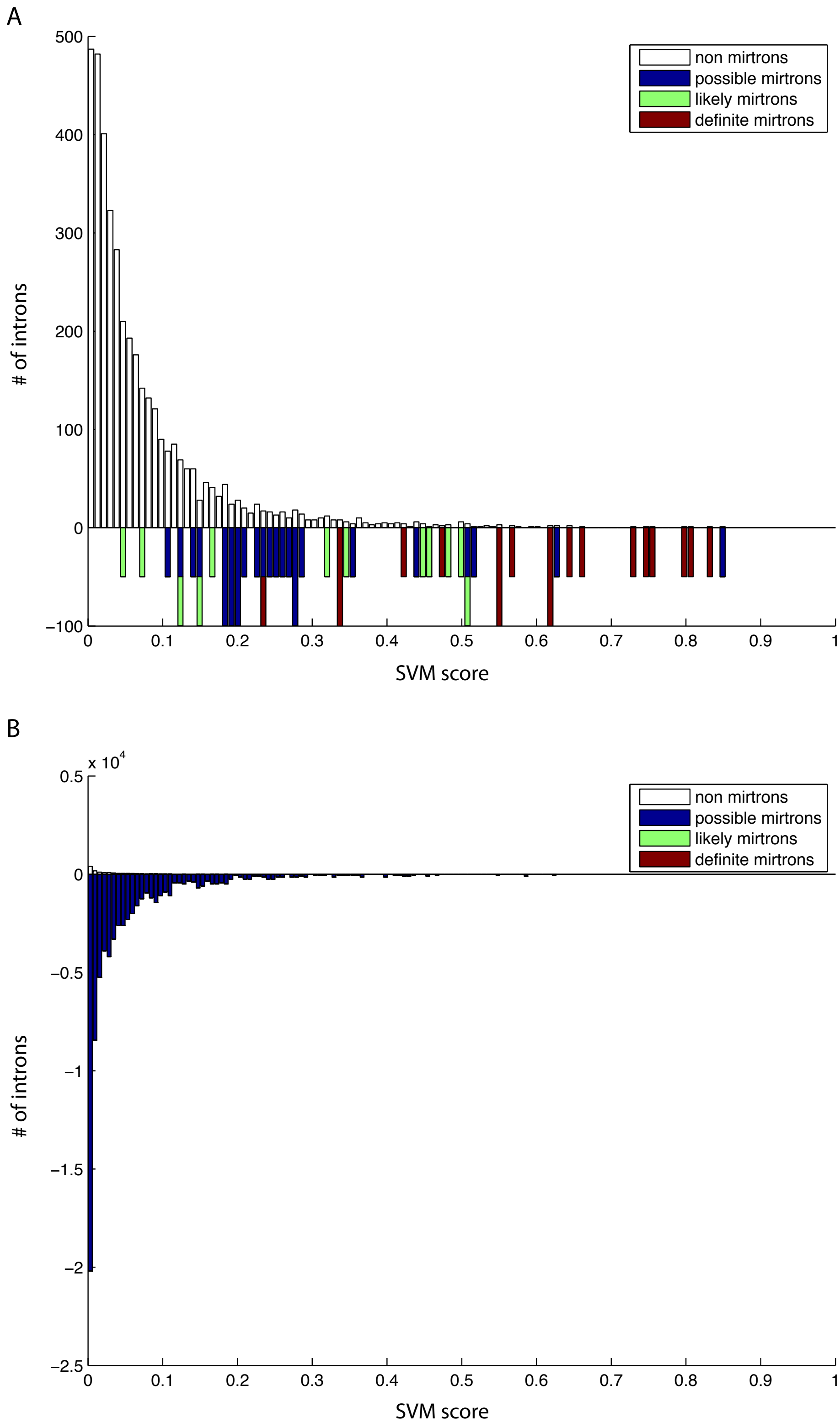


Additional Figure 2: Mirtron prediction scores histogram by SVM-method, Chung et al. 2011. **(A)** Histogram of *D. melanogaster* training data. **(B)** Histogram of *M. leidyi* predictions.
