# Supplementary material for: MicroRNAs and essential components of the microRNA processing machinery are not encoded in the genome of the ctenophore Mnemiopsis leidyi
Source: BMC Genomics. 2012 Dec 20;13:714. doi: 10.1186/1471-2164-13-714 (PMC3563456; doi:10.1186/1471-2164-13-714)
Supplement: Additional file 5 — Figure S3. shows the intron length distribution for Mnemiopsis leidyi. [file 1471-2164-13-714-S5.pdf]

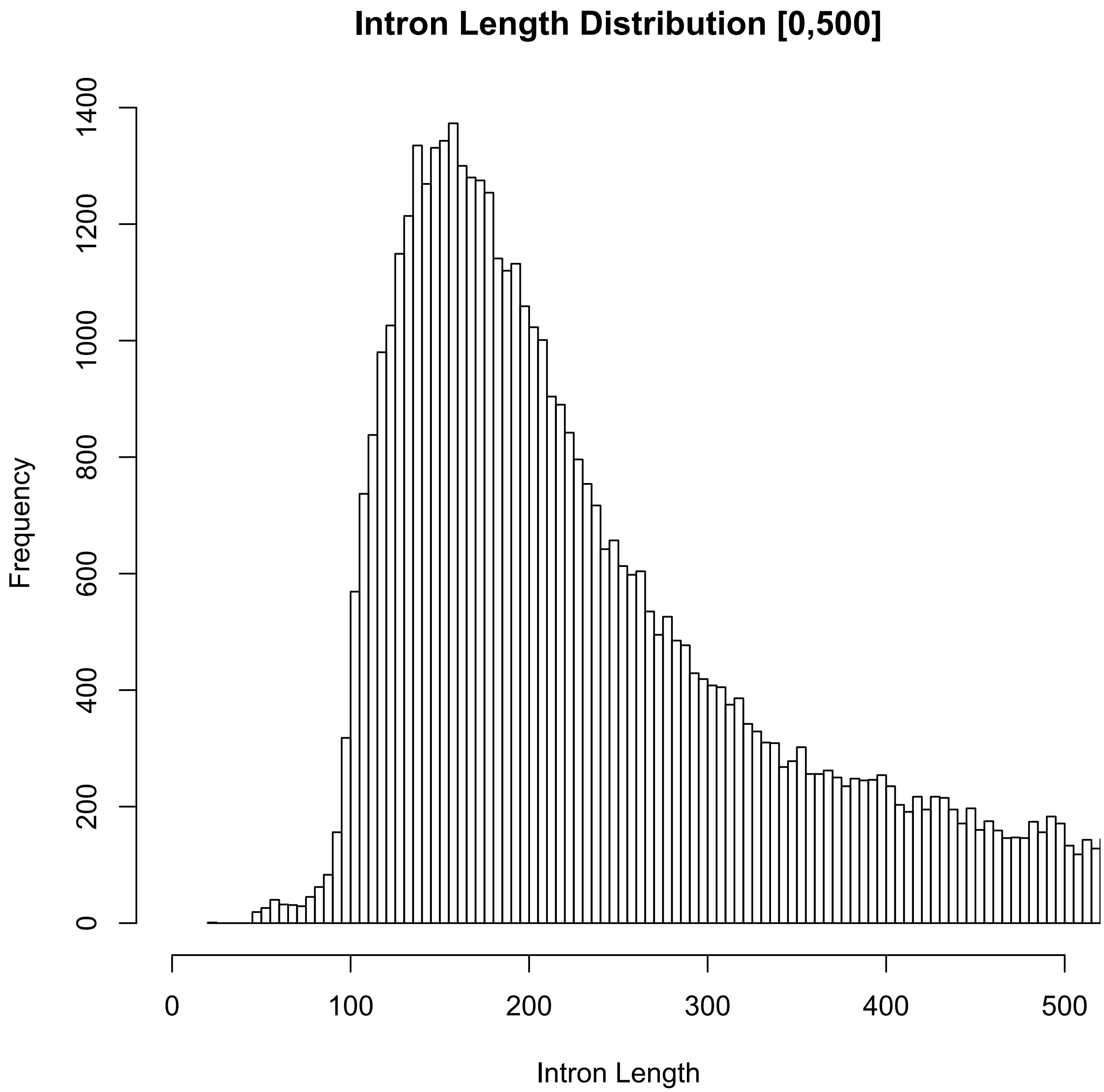

Additional Figure 3: Histogram of the intron length distribution over the *Mnemiopsis* genome. 3953 out of 69,333 introns are in the expected mirtron length range of $[50 - 120]$ nucleotides.
