# Supplementary material for: MicroRNAs and essential components of the microRNA processing machinery are not encoded in the genome of the ctenophore Mnemiopsis leidyi
Source: BMC Genomics. 2012 Dec 20;13:714. doi: 10.1186/1471-2164-13-714 (PMC3563456; doi:10.1186/1471-2164-13-714)
Supplement: Additional file 6 — Figures S4-S8. illustrate the top five mirtron preditions based on the criteria described in the Methods. [file 1471-2164-13-714-S6.zip › 2026021712724064_add6/2026021712724064_add10.pdf]

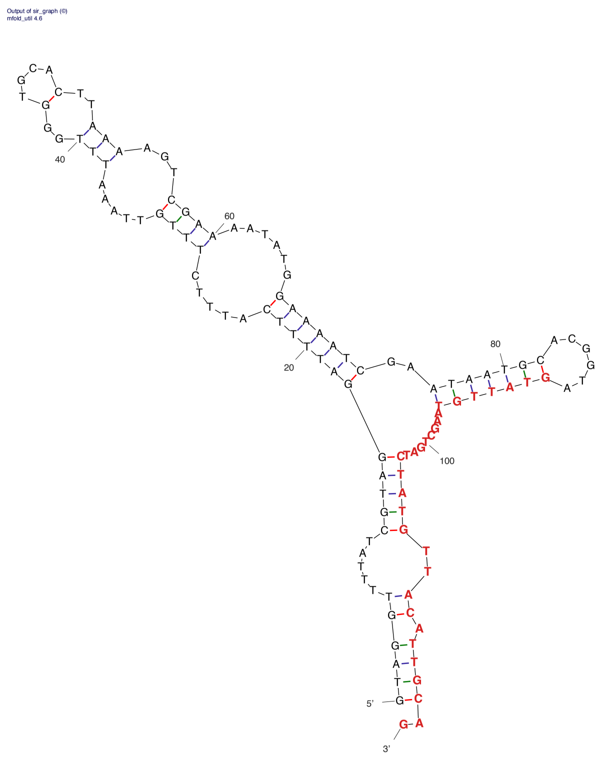


```
|GTAGGTTTTATCGTAGGATTTTCATTTCTTTGTTAAATTTGGGTGCACTTAAAAGTCGAAAATATGGAAAATCGAATAATGCACGGTAGTATTGTAAGCTGATCTATGTTACATTGCAG|
                                                                                          GTATTGTAAGCTGATCTATGTGACATTGC--|  3x2
                                                                                            ATTGAAAGCTGATCTATGTTACATTGCAG|  1x2
                                                                                          GTATTGTAAGCTGATCTATGTGACATTGCAG|  1x2
                                                                                                         TCTATGTTACATTGCAG|  1x1
                                                                                           TATTGAAAGCTGATCTATGTTACATTGCAG|  1x2
                                                                                                                   = 7 reads
```

| = Intron border
$\#x^1$ = # reads from sample 1
$\#x^2$ = # reads from sample 2

Additional Figure 7: Mirtron prediction, curated rank = 4. ML1417 226058..226176, + strand.
