# Supplementary material for: MicroRNAs and essential components of the microRNA processing machinery are not encoded in the genome of the ctenophore Mnemiopsis leidyi
Source: BMC Genomics. 2012 Dec 20;13:714. doi: 10.1186/1471-2164-13-714 (PMC3563456; doi:10.1186/1471-2164-13-714)
Supplement: Additional file 6 — Figures S4-S8. illustrate the top five mirtron preditions based on the criteria described in the Methods. [file 1471-2164-13-714-S6.zip › 2026021712724064_add6/2026021712724064_add11.pdf]

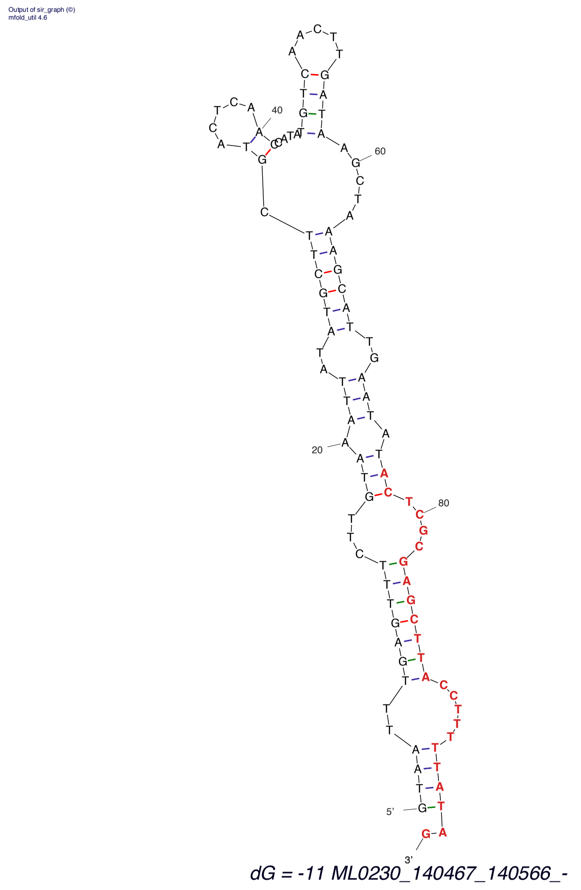


| = Intron border
#x[1] = # reads from sample 1
#x[2] = # reads from sample 2

|**GT**AATTTGAGTTTCTTGTAAATTATATGCTTCGTACTCAACCATATGTCAACTTGATAAGCTAAAGCATTGAATATACTCGCGAGCTTACCTTTTTAT**AG**|

ACTCGCGAGCTTACCTTTTTATAG $2x^2$

Additional Figure 8: Mirtron prediction, curated rank = 5. ML0230 140467..140566, - strand.
