# Supplementary material for: MicroRNAs and essential components of the microRNA processing machinery are not encoded in the genome of the ctenophore Mnemiopsis leidyi
Source: BMC Genomics. 2012 Dec 20;13:714. doi: 10.1186/1471-2164-13-714 (PMC3563456; doi:10.1186/1471-2164-13-714)
Supplement: Additional file 6 — Figures S4-S8. illustrate the top five mirtron preditions based on the criteria described in the Methods. [file 1471-2164-13-714-S6.zip › 2026021712724064_add6/2026021712724064_add7.pdf]

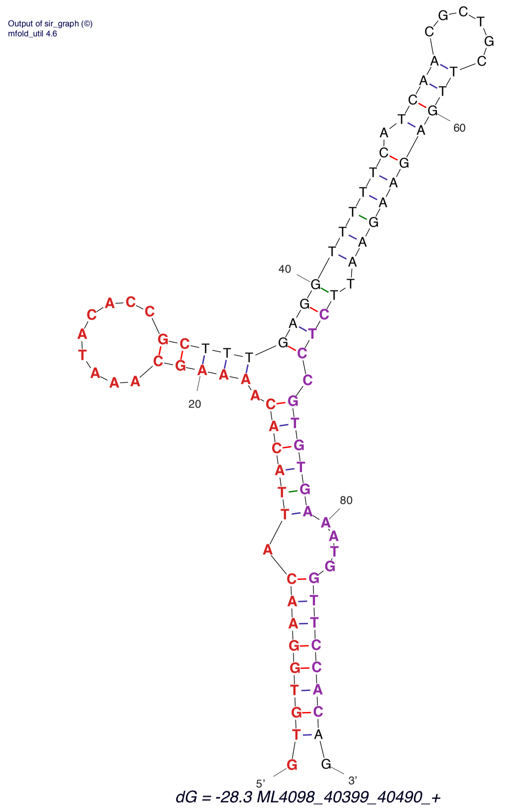

| = Intron border
#x¹ = # reads from sample 1
#x² = # reads from sample 2

```
        GAG|GTGTGGAACATTACACAAAAGCAAATACACCGCTTTGAGGTTTTTCATCAACGCTGCTTGAGAAGAATTCTCCGTGTGAAATGGTTCCACAG|AA

2x²     GAG|GTGTGGAACATTACACAAAAGC                                               CTCCGTGTGAAATGGTTCCAC--|     1x²
1x²      AG|GTGTGGAACATTACACAAAAGC
1x²         |--GTGGAACATTACACAAAAGCAAATACACC
1x²         |--GTGGAACATTACACAAAAGCAAATACACCGC
1x²         |---TGGAACATTACACAAAAGCAAATACAC
= 6 reads                                                                                                  = 1 read
```

Additional Figure 4: Mirtron prediction, curated rank = 1. ML4098 40399..40490, + strand.
