# Supplementary material for: MicroRNAs and essential components of the microRNA processing machinery are not encoded in the genome of the ctenophore Mnemiopsis leidyi
Source: BMC Genomics. 2012 Dec 20;13:714. doi: 10.1186/1471-2164-13-714 (PMC3563456; doi:10.1186/1471-2164-13-714)
Supplement: Additional file 6 — Figures S4-S8. illustrate the top five mirtron preditions based on the criteria described in the Methods. [file 1471-2164-13-714-S6.zip › 2026021712724064_add6/2026021712724064_add8.pdf]

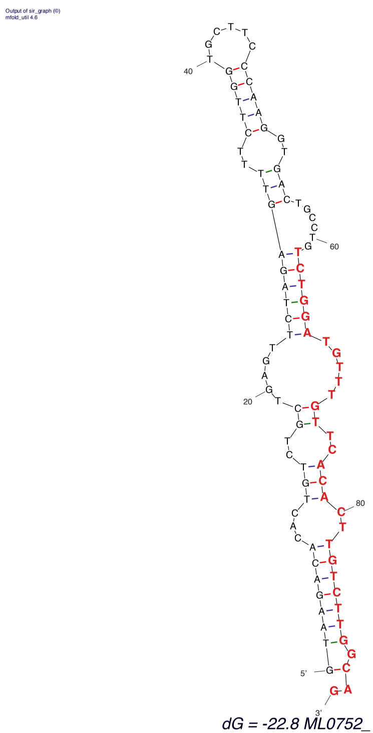

dG = -22.8 ML0752_19480_19571_-

| = Intron border
#x¹ = # reads from sample 1
#x² = # reads from sample 2

```
G|GTAAGACACACTGTCTGCTGAGTTCTAGAGTTTTCTTGGTGCTTCCCAAGGTGACTGCCTGTCTGGATGTTTGTTCACACTTGTCTTGGCAG|G

                                                                   TTTGTTCACACTTGTCTTGGC--|     5x2
                                                             TGGATGTTTGTTCACACTTGTCTTGGC--|     2x2
                                                                  GTTTGTTCACACTTGTCTTGG---|     2x2
                                                                  GTTTGTTCACACTTGTCTTGG---|     1x1
                                                         TATATGGATGTTTGTTCACACTTGTCTTGGC--|     1x2
                                                                 TGTTTGTTCACACTTGTCTTGGCA-|     1x2
                                                             TGGATGTTTGTTCAAACTTGTCTTGGC--|     1x2
                                                                      GTTCACACTTGTCTTGGCAG|G    1x2

                                                                                           = 14 reads
```

Additional Figure 5: Mirtron prediction, curated rank = 2. ML0752 19480..19571, - strand.
