# Supplementary material for: MicroRNAs and essential components of the microRNA processing machinery are not encoded in the genome of the ctenophore Mnemiopsis leidyi
Source: BMC Genomics. 2012 Dec 20;13:714. doi: 10.1186/1471-2164-13-714 (PMC3563456; doi:10.1186/1471-2164-13-714)
Supplement: Additional file 6 — Figures S4-S8. illustrate the top five mirtron preditions based on the criteria described in the Methods. [file 1471-2164-13-714-S6.zip › 2026021712724064_add6/2026021712724064_add9.pdf]

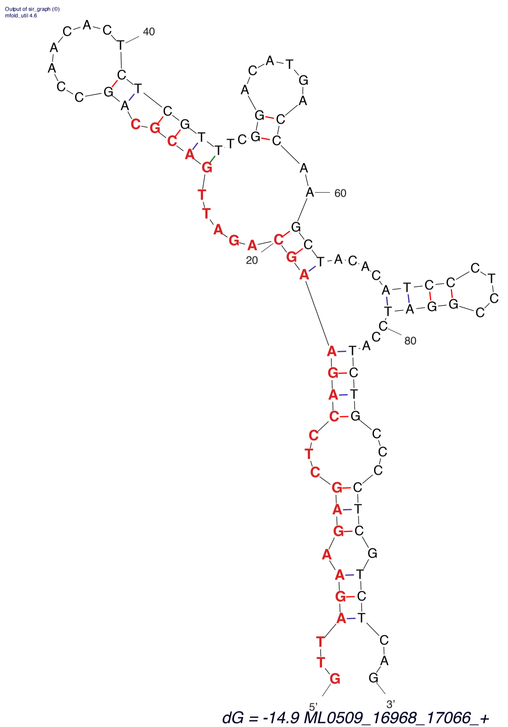


```
 |  = Intron border
#x¹ = # reads from sample 1
#x² = # reads from sample 2
```

```
       CA|GTTAGAAGAGCTCCAGAAGCAGATTGACGCAGCCAACACTCTCGTTTCGGACATGACCAAGCTACACATCCCTCCGGATCCATCTGCCCCTCGTCTCAG|

2x²      |--TAGAAGAGCTCCAGAAGCAGATTGAC
1x²      |-TTAGAAGAGCTCCAGAAGCAGATTGAC
1x²      |--TAGAAGAGCTCCAGAAGCAGATTGNC
1x²      |--TAGAAGAACTCCAGAAGCAGA
1x²      |GTTAGAAGAGCTCCAGAAG
1x²    CA|GTTAGAAGAGCTCCAGAAGCAGATTGATGC
1x²      |GTTAGAAGAGCTCCAGAAGCA
1x²      |--TAGAAGAGCTCCAGAAGCAGATTGACGC

= 9 reads
```

Additional Figure 6: Mirtron prediction, curated rank = 3. ML0509 16968..17066, + strand.
